# Ozonized Sunflower Oil: Standardization and Mechanisms of the Antimicrobial Effect

**DOI:** 10.3390/ijms26189156

**Published:** 2025-09-19

**Authors:** Matheus Henrique Vieira, Diogo Boreski, Bibiana Franzen Matte, Jean Lucas de Oliveira Arias, Celso Martins Júnior, Tais Maria Bauab, Sthefano Atique Gabriel, Chung Man Chin

**Affiliations:** 1 Laboratory for Drug Design (LAPDESF), School of Pharmaceutical Sciences, University of São Paulo State (UNESP), Araraquara 14800-903, Brazil; mh.vieira@unesp.br (M.H.V.); diogo.boreski@unesp.br (D.B.); celso.junior@unesp.br (C.M.J.); 2 Vitro Center, Rua da Várzea, 22, Jardim São Pedro, Porto Alegre 91040-600, Brazil; bibiana@nucleovitro.com; 3 School of Chemistry and Food, Federal University of Rio Grande (FURG), Av. Itália, Km 8, Carreiros Campus, Rio Grande 96203-900, Brazil; jeanarias@furg.br; 4 Laboratory of Microbiology, School of Pharmaceutical Sciences, University of São Paulo State (UNESP), Araraquara 14800-903, Brazil; 5 Advanced Research Center in Medicine (CEPAM), School of Medicine, Union of the Colleges of the Great Lakes (UNILAGO), Sao Jose do Rio Preto 15030-070, Brazil; sthefano@unilago.edu.br

**Keywords:** ozone, antibacterial, standardization, sunflower oil

## Abstract

Ozonized vegetable oils are gaining attention for their antimicrobial and therapeutic potential, yet the lack of standardized ozonation protocols and incomplete characterization of their chemical profiles hinder clinical translation. In this study, we standardized the ozonation process of sunflower oil and investigated the chemical evolution and antimicrobial efficacy of the resulting products. Ozonation proceeded through a classical three-step mechanism involving the formation of primary ozonides, their decomposition into carbonyl compounds and carbonyl oxides, and subsequent recombination into stable secondary ozonides capable of sustained ozone release with reduced toxicity. Time-course analysis at 100, 240, and 480 min revealed key reaction products, including the appearance of azelaic acid after 240 min, progressive depletion of linoleic acid, and the emergence of 2,5-furandione exclusively after 480 min—indicative of advanced oxidative processes. The formation of hydroperoxides and their secondary degradation into ketones, acids, and epoxides was also observed, with implications for both biological activity and sensory properties. Importantly, the ozonized oil demonstrated potent antimicrobial activity against *Staphylococcus aureus*, *Escherichia coli*, *Salmonella choleraesuis*, *Pseudomonas aeruginosa*, *Candida albicans*, and *Aspergillus brasiliensis*. These findings provide a comprehensive chemical and functional characterization of ozonized sunflower oil and support its development as a standardized antimicrobial agent for therapeutic use.

## 1. Introduction

Ozone (O_3_) is a highly reactive triatomic molecule naturally formed in the stratosphere through the photodissociation of molecular oxygen (O_2_) by ultraviolet (UV) radiation [1]. First synthesized in the laboratory by Christian Friedrich Schönbein in 1839 [2], ozone has since been recognized for its potent oxidative potential, which confers a dualistic role in biological systems—capable of inducing both therapeutic benefits and cytotoxic effects [3]. While systemic ozone therapy remains contentious and is not approved by regulatory agencies such as the U.S. Food and Drug Administration (FDA), topical ozone-based interventions—especially ozonized vegetable oils—have garnered increasing attention as alternative approaches to managing cutaneous infections and inflammatory disorders [4].

Ozonized sunflower oil (OSO) is synthesized by bubbling ozone gas through matrices rich in unsaturated fatty acids, primarily linoleic and oleic acids, which are abundant in sunflower oil. This process follows the Criegee mechanism, wherein ozone reacts with carbon–carbon double bonds in unsaturated lipids, leading to the formation of a complex mixture of oxidation products, including ozonides, peroxides, aldehydes, and dicarboxylic acids [5]. These reactive oxygen species are believed to contribute to both the antimicrobial properties and tissue-reparative effects of OSO formulations [5,6]

Mechanistically, these compounds disrupt microbial integrity through lipid peroxidation and protein denaturation. Additionally, some evidence suggests that OSO may modulate host cellular responses by influencing inflammatory signaling and oxidative stress pathways [7,8].

While ozone gas in its native form is highly reactive and thermodynamically unstable, its stability increases under specific aqueous conditions. Galdeano et al. [9] indicates that aqueous ozone is relatively stable at acidic pH ~3.0 and low temperatures, approximately 8 °C, with a half-life of up to 20 min under these parameters. In contrast, in ozonized oils, ozone does not remain in free form but instead participates in chemical reactions that produce more stable secondary products, such as ozonides, aldehydes, alcohols, and carboxylic acids, which are responsible for the oil’s prolonged biological activity [10].

However, the therapeutic application of OSO is constrained by critical gaps in standardization. Variables such as ozonation time, temperature, and oxygen concentration profoundly influence the chemical profile and biological activity of the final product. Moreover, prolonged or uncontrolled ozonation may result in the formation of toxic byproducts—such as formaldehyde, hydroperoxides, and epoxides, which raise safety concerns for clinical use [11,12,13]. Environmental factors like light, heat, and air exposure further contribute to batch-to-batch variability, undermining reproducibility and regulatory acceptance.

Amid the global crisis of antimicrobial resistance and the urgent need for alternative, non-antibiotic treatments, OSO presents a compelling therapeutic candidate. Its broad-spectrum efficacy against bacteria, fungi, and even certain viruses [7]—coupled with a low risk of resistance emergence due to its multimodal oxidative mechanism—positions it as a promising adjunct in dermatological care.

To address these challenges, the present study aimed to (1) establish a standardized ozonation protocol for sunflower oil under controlled physicochemical conditions; (2) characterize the chemical evolution of oxidation products over time using gas chromatography–mass spectrometry (GC-MS); (3) evaluate the antimicrobial efficacy of ozonized samples through agar diffusion, minimum inhibitory concentration (MIC), and time-kill assays; and (4) assess the cytotoxic profile of OSO in mammalian fibroblast cultures. Together, these objectives are designed to inform the rational development of a stable, bioactive, and clinically viable OSO formulation.

## 2. Results

To establish a standardized sample for this study, we compared OSO at different times of ozone exposure and evaluated antimicrobial activity. The sample ozonized for 100 min demonstrated no detectable antimicrobial activity. The 240 min sample produced modest activity, while the 480 min sample showed stronger results when compared to the positive control. Based on these findings, the OSO 480 min sample was selected for further analysis.

Analysis of the OSO 480 min revealed the presence of key oxidative derivatives formed from unsaturated native fatty acid chains of sunflower oil (SO). The control (non-ozonized) SO presented in its composition: 33.10% oleic acid, 47.13% linoleic acid, and 0.41% linolenic acid. Table 1 shows the GC-MS results by peak. The CG-MS spectrum is presented in the Supplementary Material. These values align with previous studies indicating that SO typically comprises ~85% unsaturated fatty acids (14–43% oleic and 44–75% linoleic acids) [14]. These findings are summarized in Table 1, showing the relative compound content in the 4 samples (SO, OSO-100 min, OSO-240 min, and OSO-480 min)

### 2.1. Acidity Index

**Figure 1:**
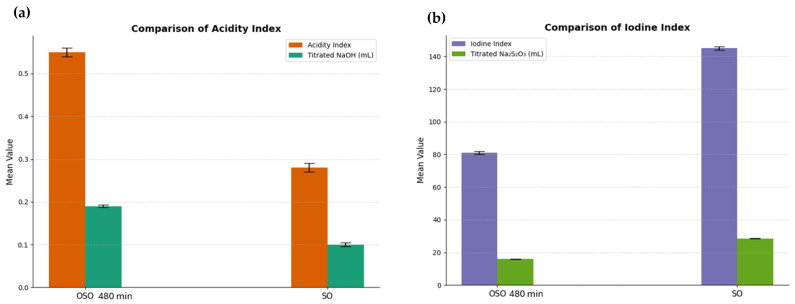
Acidity index (**a**) and iodine index (**b**) comparison of ozonized sunflower oil 480 min (OSO 480 min) with non-ozonized sunflower oil (SO).

The acidity index, which reflects the accumulation of free fatty acids and oxidative byproducts such as aldehydes and carboxylic acids, increased markedly from 1.2 mg KOH/g in the SO to 5.8 mg KOH/g after 480 min of ozonation, as shown in Figure 1. This elevation was expected since oxidative degradation of triglycerides by prolonged ozone exposure generates low-molecular-weight acidic compounds via oxidative cleavage of unsaturated chains.

### 2.2. Iodine Index

Iodine index decreased significantly from 110 g I_2_/100 g to 58 g I_2_/100 g, as shown in Figure 1. This reduction indicates the consumption of carbon–carbon double bonds and the oxidative saturation of polyunsaturated fatty acids. The depletion of iodine-reactive sites underscores the structural transformation of the oil into a more oxidized and chemically stable formulation, rich in secondary bioactive compounds.

### 2.3. Peroxide Index

**Figure 2:**
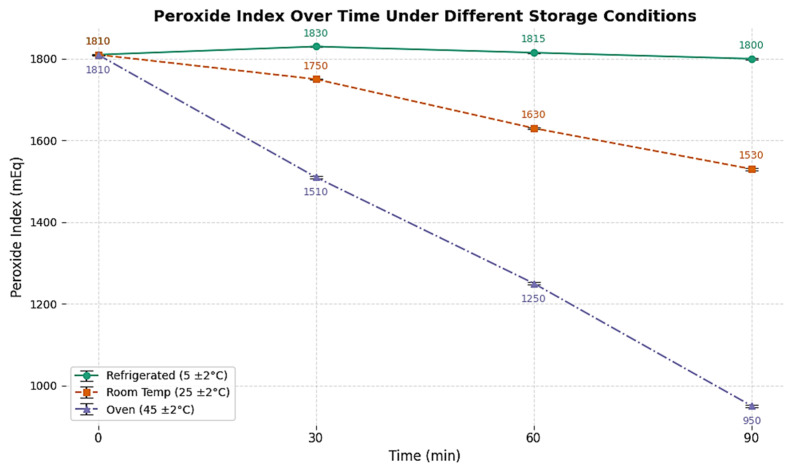
Peroxide index over time in different storage conditions of ozonized sunflower oil 480 min (OSO 480 min).

The peroxide index showed a progressive rise from 20 meq O_2_/kg in SO to 125 meq O_2_/kg after 480 min of ozonization in different storage conditions, as shown in Figure 2. This trend is indicative of sustained formation of hydroperoxides and ozonide intermediates, probably led by radical-mediated propagation mechanisms. While these species contribute to antimicrobial activity, they also raise concerns regarding sensory deterioration and potential toxicity if not properly managed.

### 2.4. Antimicrobial Activity

#### Agar Diffusion (Pour-Plate)

The agar diffusion (pour-plate) method was employed to preliminarily assess the antimicrobial activity of OSO. This technique involves seeding a standardized microbial suspension onto agar plates, followed by the application of test samples into wells or onto impregnated disks. The control was Oxoid™ Novobiocin Susceptibility disks. After incubation, the formation of inhibition zones around the wells serves as a direct indicator of antimicrobial efficacy.

In our study, the OSO-480 min demonstrated inhibition zones against all tested strains: *Staphylococcus aureus* ATCC 6538, *Escherichia coli* ATCC 8739, *Salmonella choleraesuis* ATCC 10718, *Pseudomonas aeruginosa* ATCC 9027, *Candida albicans* ATCC 10231, and *Aspergillus brasiliensis* ATCC 16404. These findings suggest the broad-spectrum antimicrobial potential of the OSO-480 min.

**Figure 3:**
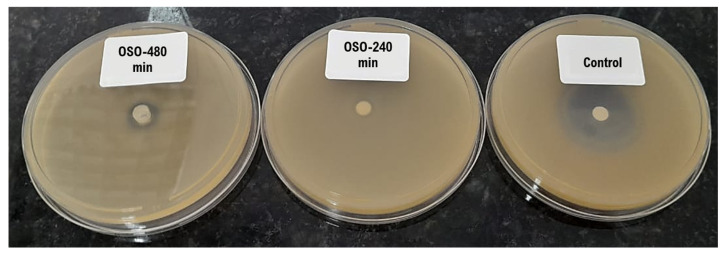
Agar diffusion plates comparison of OSO-480 min, OSO-240 min, and control (Oxoid™ Novobiocin disk).

In contrast, the OSO-100 min did not exhibit inhibition zones, indicating insufficient formation of antimicrobial oxidative products at early timepoints. The 240 min sample showed intermediate activity, with smaller halo diameters that reflect suboptimal yet progressing bioactivity. These differences are visually illustrated in Figure 3, where a marked increase in halo size correlates with longer ozonation durations, supporting the time-dependent generation of antimicrobial compounds. The microorganism used for pour-plate, presented in Figure 3, was *S. aureus*. Together, these results suggest that only extended ozonation (≥480 min) produces a chemical profile rich enough in ozonides and peroxidation products to effectively inhibit microbial growth in vitro.

### 2.5. Time-Kill

The time-kill assay was conducted to evaluate the dynamic antimicrobial efficacy of OSO-480 min over short contact intervals. This method quantifies microbial load reduction at defined timepoints, allowing for kinetic insights into the rate and extent of microbial inactivation.

The assessments demonstrated that OSO-480 min exerted rapid and potent antimicrobial activity against all tested microorganisms, achieving ≥3-log reductions within 2 min. Among Gram-negative species, *P. aeruginosa* and *E. coli* exhibited reductions of 4.10 and 3.54 log units, respectively, while *S. choleraesuis* showed the most pronounced response, with a 5.63-log reduction (>99.999%) after 2 min, as shown in Table 2.

The Gram-positive *S. aureus* demonstrated slightly lower susceptibility but still achieved a 3.63-log reduction within the same interval. Fungal strains were highly sensitive, with *A. brasiliensis* and *C. albicans* falling below the detection threshold (<10 CFU/mL) after 1–2 min, indicating a fungicidal effect. Collectively, these findings imply OSO-480 min as a broad-spectrum, fast-acting antimicrobial formulation capable of achieving potential antibacterial activity for further pre-clinical studies.

### 2.6. Minimum Inhibitory Concentration

The minimum inhibitory concentration assay (MIC) was performed to evaluate the antimicrobial efficacy of OSO-480 min.

The assessments revealed concentration-dependent inhibition of all tested microorganisms, as shown in Table 3. *S. aureus* showed complete growth inhibition at concentrations ≥ 12.5%, with regrowth observed at 6.25% and below. *E. coli* required a higher concentration, with inhibition at ≥50%, while growth persisted at 25% and lower. *A. brasiliensis* was inhibited at a concentration of ≥25% but showed growth at 12.5% and below. *C. albicans* exhibited a similar pattern to *S. aureus*, with inhibition at ≥12.5% and growth detected at 6.25% and lower. These findings demonstrate that OSO-480 min possesses potent antimicrobial activity against both bacterial and fungal strains, with Gram-positive bacteria and yeasts displaying higher sensitivity than Gram-negative bacteria and filamentous fungi.

### 2.7. Cytotoxicity and Cell Viability

Cytotoxicity analysis evaluated the hazardous potential of OSO-480 min to Balb.c 3T3 clone 31 fibroblast cells. The negative control was normalized to 100% cell viability, and the percentage viability for each sample and the positive control was calculated relative to the control. Samples were considered cytotoxic when they induced a reduction in cell viability of 30% or more.

The sample was dispersed in TSB by vigorous agitation, resulting in a heterogeneous suspension. Subsequently, the microbial inoculum solution, prepared in saline, was added under gentle stirring to ensure temporary dispersion of the sample and direct contact with the microorganisms during the MIC assay. No pre-solubilizing agent was added to avoid interference with the results.

**Figure 4:**
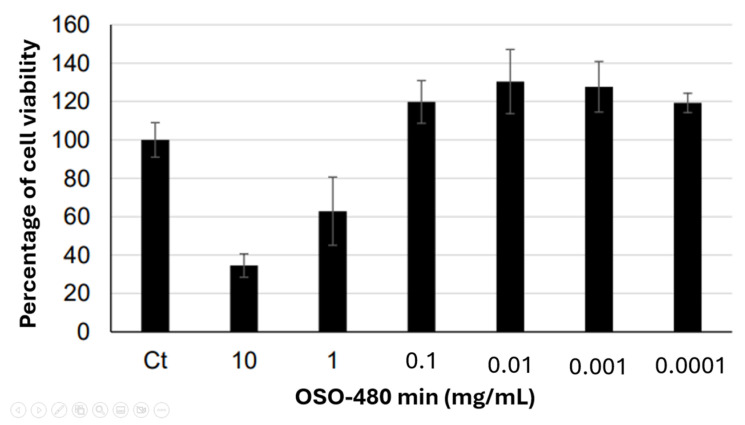
Percentage of cell viability following treatment with OSO-480 min.

OSO-480 min was solubilized in growth medium and applied to cell cultures at concentrations of 10, 1, and 0.1 mg/mL. Marked cytotoxicity was observed at 10 mg/mL and 1 mg/mL. At 0.1 mg/mL, no cytotoxic effect was detected, with cell viability above the cytotoxicity threshold. The positive control (100 μg/mL) resulted in cell viability of 14.3%. The cell viability data for all groups is presented in Figure 4. Cell viability was normalized to the untreated control (100%); values > 100% reflect treatment-induced proliferation or metabolic stimulation, while samples with ≤30% viability were classified as cytotoxic. Sodium lauryl sulfate (100 µg/mL) was used as the positive control.

## 3. Discussion

Ozone is a naturally occurring triatomic molecule found in the Earth’s stratosphere and known for its high oxidative capacity. This unique structure—comprising three oxygen atoms—has recently gained prominence for its associated antimicrobial and therapeutic effects. Ozone therapy can be applied in various forms, including ozonated hydrotherapy, ozonated oils, autohemotherapy, and other innovative delivery systems. Topical ozone applications have shown promise in treating numerous dermatological conditions, such as infectious dermatoses [15], chronic wounds [16], psoriasis [17], atopic dermatitis [18] and, diabetic foot ulcers [19,20]. Additionally, research suggests ozone therapy may support microbiome modulation and has potential in antitumor and anti-aging interventions [18].

Despite its therapeutic potential, ozone is an inherently unstable molecule able to generate reactive and potentially toxic species, including peroxides, hydroperoxides, formaldehyde, epoxides, and polymeric ozonides [21]. As such, the development of ozonation protocols must carefully control these byproducts to ensure both efficacy and safety. Sunflower and olive oils have been commonly selected as substrates in studies of ozonized vegetable oils due to their high degree of unsaturation, which facilitates efficient ozonation.

This study provides evidence for the antimicrobial potential of OSO through integrated chemical and biological assessments. Controlled ozonation produced significant physicochemical modifications—reflected by increased peroxide and acidity indices and a notable decrease in the iodine value—which are in line with Criegee’s mechanism for unsaturated fatty acid saturation and byproduct formation. While these chemical shifts support the formation of reactive compounds such as ozonides and aldehydes, the direct relationship between specific byproducts and antimicrobial activity remains inferential rather than demonstrated. It is also important to note that the formation of reactive oxygen species (ROS), though likely, was not quantified in this study, which limits our understanding of the precise pathways involved in microbial inactivation.

Moreover, the random covalent interactions proposed between these oxidative byproducts and microbial macromolecules (e.g., proteins, DNA) are well described and established in the literature [22,23]. As such, attributing the observed antimicrobial effects solely to these mechanisms may be premature. A more detailed molecular characterization—ideally coupled with targeted microbiological assays—would be necessary to unravel the contributions of each compound. Finally, the safety and selectivity of OSO against non-target cells or tissues remain unaddressed, raising questions about its broader applicability and potential side effects.

**Figure 5:**
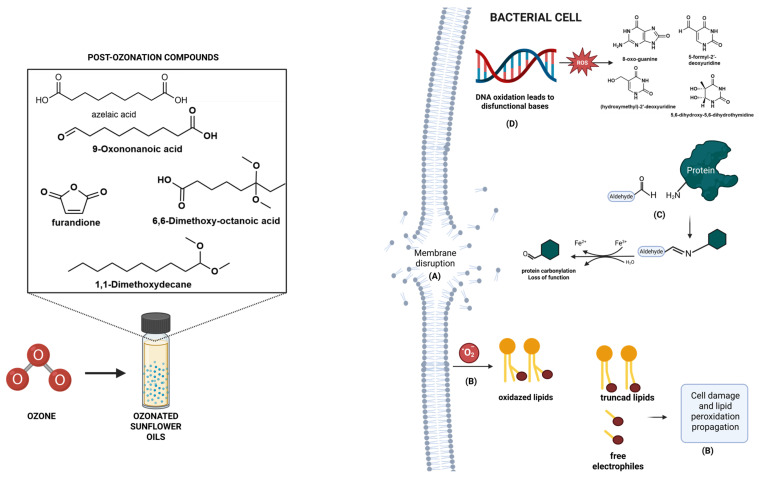
Post-ozonization compounds formed in sunflower oil such as -methoxy (6,6-dimethoxy-octanoic acid; 1,1-dimethoxydecane), carboxylic acid (azelaic acid), anhydride (furanedione) and, -formyl (9-oxononanoic acid) derivatives, in addition ozonide can lead to membrane disruption (**A**), through lipid peroxidation (**B**) and, cell and biomolecules damage such as protein carbonylation (**C**), DNA oxidation and formation of oxidated bases (**D**). Created in BioRender. Chin, C. (2025).

In addition, gas chromatography–mass spectrometry (GC-MS) analysis revealed the emergence of signature secondary oxidation products, including 2,5-furandione, and azelaic acid and the possible mechanisms of ozone byproducts, resulting in protein carbonylation with loss of protein functionality [24], DNA oxidation and dysfunctional nucleotides [25] and, lipid peroxidation that leads to truncated lipids and free radicals besides highly reactive electrophiles [26] as illustrated in Figure 5. The exact mechanism of action still lacks in-depth investigation.

**Figure 6:**
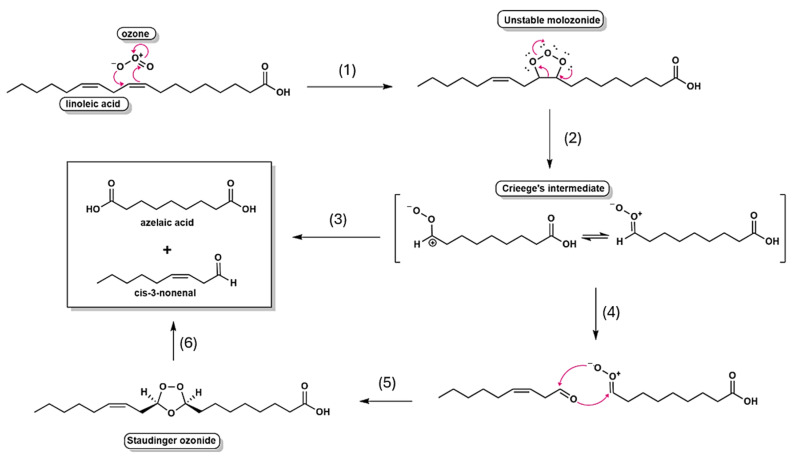
Mechanism of linoleic acid ozonolysis: (**1**) Addition of ozone to the double bond of linoleic acid, resulting in the formation of an unstable molozonide intermediate; (**2**) Rearrangement of the molozonide, leading to the formation of the highly reactive Criegee intermediate; (**3**) Oxidative cleavage of the Criegee intermediate, yielding the main products: azelaic acid and cis-3-nonenal; (**4**) Alternative rearrangements that produce cyclic ozonides (Staudinger ozonide) under specific conditions; (**5**) Formation of additional derivative products as a result of intermediate instability and influence of the chemical environment; (**6**) Major products generated from the ozonolysis of linoleic acid: azelaic acid (dicarboxylic acid) and cis-3-nonenal (unsaturated aldehyde). Pink arrow represents electron attack.

Regarding the mechanisms involving ozonolysis, the reactivity of ozone toward unsaturated fatty acids is determined by the degree of unsaturation, with oils rich in double bonds require extended ozonation times to achieve complete conversion. Thus, the kinetics of the process are intrinsically linked to the origin and fatty acid composition of the oil, leading to distinct reactivity profiles for olive oil, conventional sunflower oil, and high-oleic sunflower oil [7]. Mechanistic investigations, pioneered by Criegee, established that ozonolysis proceeds through a multistep sequence: the initial formation of unstable primary ozonides (1,2,3-trioxolanes), followed by their fragmentation into carbonyl compounds and reactive carbonyl oxides (Criegee’s intermediates), which subsequently recombine to yield more stable secondary ozonides (1,2,4-trioxolanes) [5]. As depicted in Figure 6, this mechanistic framework underscores the central role of transient intermediates in dictating both the efficiency and the selectivity of ozone addition to unsaturated substrates.

The delayed appearance of these bioactive compounds underscores the necessity of studies towards prolonged ozonation, aiming to verify the optimization of therapeutic efficacy. Importantly, this finding challenges the reliance on conventional quality indicators—such as the peroxide index—as sole proxies for bioactivity, advocating instead for direct molecular characterization. Furthermore, about 50 peaks were determined in the chromatogram (Supplementary Material), including some interesting compounds such as epoxides (Oxirane, hexadecyl-1,2-Epoxyoctadecane 2-Hexadecyloxirane), benzofuran derivatives ((3S,3aS,7aR)-3,6-dimethyl-2,3,3a,4,5,7a-hexahydro-1-benzofuran), clycoheptafuran derivatives (Ethanone, 1-(5,6,7,8-tetrahydro-2,8,8-trimethyl-4H-cyclohepta[b]furan-5-yl)), and benzoic esters derivatives (4-[(2,4-Dimethoxy-6-pentylbenzoyl)oxy]-2-methoxy-6-pentylbenzoic acid methyl ester). These findings suggest a load of compounds that can exert, itself or synergistically, the antimicrobial activity.

Microbiological assays, including agar diffusion, time-kill kinetics, and MIC, demonstrated that OSO ozonized for 480 min exhibits broad-spectrum efficacy. Significant inhibition was observed against Gram-positive bacteria (*S. aureus*), Gram-negative strains (*E. coli*, *S. choleraesuis*, *P. aeruginosa*), and opportunistic fungi (*C. albicans*, *A. brasiliensis*). Furthermore, although the inhibition zone observed for OSO 480 min appeared very small compared with the control (Figure 3), the pour-plate method is not suitable for evaluating oil-based samples in biological screenings. Due to the hydrophobic nature of OSO, the compound does not diffuse efficiently through the agar matrix, which results in an incomplete assessment of its antimicrobial potential. In contrast, the time-kill assay revealed a 3.63-log reduction in *S. aureus* after only 2 min of contact, indicating that direct interaction between OSO and the bacterial matrix is essential to achieve reliable antimicrobial activity. These results are consistent with [7] linking the antimicrobial action of ozonized oils to membrane disruption via lipid peroxidation and oxidative protein denaturation.

A particularly notable finding was the pronounced susceptibility of *C. albicans*, with growth inhibition achieved at OSO concentrations as low as 6.25%, whereas *E. coli* required concentrations of 50% for complete inhibition. This differential response suggests species-specific oxidative resistance, potentially mediated by cell wall composition and antioxidant defense systems. The data parallel findings by Slavinskienė et al. [27], who also noted variable MICs among microbial species and emphasized sunflower and linseed oils as superior carriers for ozone-derived bioactives.

Despite these promising results, the cytotoxicity profile raises caution. Fibroblast viability (Balb/c 3T3 line) was significantly compromised at concentrations between 1 and 10 µg/mL, aligning with studies by Colombo et al. [28] and Günaydin et al. [12] that highlight the therapeutic window of ozonized oils. This dose-dependent toxicity underscores the need for careful formulation and dilution to harness antimicrobial efficacy while minimizing adverse host effects.

Regarding the possible molecular mechanisms associated with OSO 480 min, the presence of alcohols, aldehydes, acetals, and methoxylated derivatives may exert antimicrobial activity through multiple pathways. Formaldehyde and glutaraldehyde, for example, are well-established agents used in the sterilization of surgical equipment, demonstrating broad-spectrum action [4,29]. In addition, ethanol and isopropanol—commonly present in alcohol-based sanitizers—act primarily through disruption of microbial cell membranes [30].

Duah Boakye et al. [31] demonstrated the antimicrobial mechanism of aliphatic α,β-unsaturated aldehydes, including (E)-2-hexenal, (E)-2-heptenal, (E)-2-octenal, (E)-2-nonenal, (E)-2-decenal, and (E,E)-2,4-decadienal. Their findings suggest that 2E-alkenals likely cause significant perturbation of the lipid fraction of plasma membranes and are capable of penetrating bacterial cells. Specifically, aldehydes and unsaturated structures can form covalent bonds with biological targets such as lipid membranes and essential proteins, thereby disrupting cellular integrity and function.

The antimicrobial mechanisms of action of other aldehydes, such as *o*-phthalaldehyde, are likely to involve interaction with the cytoplasmic membrane and increase in its permeability [28,29]. *o*-Phthalaldehyde also appears to kill spores by blocking the spore germination process. Although membrane functional proteins are generally supposed to be the potential targets toward which aldehydic antimicrobial agents are directed, other mechanisms of action/interaction can help explain their antimicrobial activity [31].

Clinically, OSO and related formulations showed a potential strategy for managing skin infections, chronic wounds, and fungal dermatosis. Their efficacy, multimodal oxidative mechanism, and low tendency for resistance make them valuable adjuncts in the fight against antimicrobial resistance. A randomized controlled clinical trial demonstrated that the topical application of ozonated oil significantly enhanced epithelial healing and gingival health following free gingival graft surgery [32]. Similarly, in patients with chronic periodontitis, the adjunctive use of ozonated olive oil with scaling and root planing led to notable improvements in both clinical and microbiological outcomes [32]. In a clinical trial by Campanati et al. [33], ozonated oil was compared with hyaluronic acid gel for the treatment of second-degree burns; both treatments were effective in promoting healing, but ozonated oil was more effective in reducing post-lesional hyperpigmentation. Furthermore, a 2023 systematic review of 13 clinical studies concluded that ozonated oil typically shortened healing times for oral lesions and was not associated with any adverse effects [34].

### 3.1. Conclusions

This study established a standardized ozonation protocol for sunflower oil and demonstrated its progressive chemical transformation into bioactive aldehydes, acids, and ozonides, accompanied by consistent changes in acidity, peroxide, and iodine indices. The 480 min ozonized oil exhibited potent, broad-spectrum antimicrobial activity against bacteria and fungi, with rapid microbicidal effects confirmed by time-kill assays. However, cytotoxicity at higher concentrations highlights the importance of dose optimization and formulation strategies to ensure safety. Together, these findings position ozonized sunflower oil as a promising topical antimicrobial candidate and provide a reproducible framework to guide future preclinical and clinical studies.

### 3.2. Limitations and Perspectives

A key limitation of this study was the use of a cellular model (Balb/c 3T3) that, although well-established for cytotoxicity screening, does not adequately simulate human dermal tissue or approximate the intended application of the OSO, which is targeted for skin infections. This limitation may have influenced the outcomes and limits the extrapolation of the findings to clinical scenarios. Furthermore, the assessment of minimum inhibitory concentration was affected by the hydrophobic nature of oil, impacting the results. Therefore, future studies should employ more representative models of human skin for both permeation and toxicity assessments, followed by in vivo investigations to more accurately evaluate the therapeutic potential and safety profile of OSO for dermatological use.

### 3.3. Disclaimer

Any potential therapeutic application of the procedures and findings described in this study must strictly comply with the applicable national and international medical-device regulations and directives. The data presented here are intended solely for scientific and research purposes and do not constitute clinical guidance or regulatory approval for medical use.

## 4. Materials and Methods

### 4.1. Ozonized Oil Preparation and Chemical Characterization

OSO was prepared using a PHILOZON^®^ MEDPLUS model ONE ozone generator, Nova Esperança, Sao Paulo, Brazil (ANVISA certified 25351.011611/2015-18), operating with medicinal-grade oxygen (Oxitab^®^ PHILOZON, Nova Esperança, Sao Paulo, Brazil) at a concentration of 60 μg/mL. The system was maintained at a pressure of 2.0 kgf/cm^2^ with a gas flow rate of 1 ozone was introduced into 100 mL of pharmaceutical-grade sunflower oil contained in a 250 mL amber glass vessel via a medical-grade silicone tube connected to a porous Acquap^®^ stone diffuser (Hengko technology Co., Ltd., Shenzhen, Guangdong, China). The ozonation process was conducted at three standardized durations: 100, 240, and 480 min to monitor progressive oxidative transformations.

Chemical profiling of the ozonated oils was performed using gas chromatography coupled with tandem mass spectrometry (GC-MS/MS) on a Shimadzu GC-2010 Plus system (Shimadzu Scientific Instruments, Columbia, MD, USA), equipped with a triple quadrupole mass spectrometer (TQ 8050; Shimadzu Scientific Instruments, Columbia, MD, USA), and an automatic sampler (Combipal AOC-6000; CTC Analytics AG, Zwingen, Switzerland). Separation was achieved using an Rtx^®^-5MS capillary column (30 m × 0.25 mm i.d., 0.25 μm film thickness; Restek Corporation, Bellefonte, PA, USA). The GC oven program initiated at 80 °C (held for 1 min), ramped at 10 °C/min to 180 °C, then at 7 °C/min to a final temperature of 330 °C, for a total run time of 32.43 min. The injector temperature was maintained at 250 °C, with a 1 μL injection volume and a split ratio of 1:10. Helium (99.99%) was used as the carrier gas at a constant flow rate of 1.30 mL/min. Ionization was carried out by electron impact (EI) at 70 eV, with the interface and ion source temperatures set to 280 °C and 230 °C, respectively. Data acquisition was conducted in full scan mode over an m/z range of 50–500, and analysis was performed using GCMS solution software (version 4.45 SP1, Shimadzu^®^ Scientific Instruments, Columbia, MD, USA).

Fatty acid profiling was conducted via alkaline hydrolysis. A 30 mg aliquot of oil was mixed with 500 μL of 0.1 mol/L potassium hydroxide and incubated in a water bath at 60 °C for 90 min. Following acidification with 1.5 mL of 1 mol/L sulfuric acid, the mixture was cooled to room temperature and extracted with 2 mL of hexane. The organic phase was separated and directly injected into the GC-MS/MS system.

To evaluate the unsaponifiable fraction, derivatization to fatty acid methyl esters (FAMEs) was performed. A 500 mg oil sample was saponified with 5 mL of 2 mol/L ethanolic potassium hydroxide in a water bath at 80 °C for 60 min. After cooling, 2 mL of distilled water and 8 mL of hexane were added. The mixture was vortexed for 2 min and centrifuged for 5 min. The resulting hexane layer containing the derivatized unsaponifiable components was injected into the GC-MS/MS for qualitative analysis.

### 4.2. Physicochemical Evaluation

#### 4.2.1. Acidity Index Evaluation

To assess the oxidative degradation of ozonized oil, the acidity index was determined via titration. A 2.0 g aliquot of each oil sample was homogenized with 25 mL of absolute ethanol for 1 min. Phenolphthalein was used as a pH indicator. Titration was performed using a standardized 0.1 mol/L sodium hydroxide (NaOH) solution, added dropwise under continuous stirring until a persistent pink coloration appeared and remained stable for at least 30 s. The acidity index, expressed in mg KOH/g of oil, was calculated using the following equation:

(1) $$\mathrm{Acidic}\;\mathrm{Index}=\frac{v\cdot f\cdot 5.61}{P}$$

*v* = volume of 0.1 M sodium hydroxide solution titrated (mL)

*f* = sodium hydroxide solution concentration

*P* = sample weight (g)

#### 4.2.2. Peroxide Index Evaluation

Peroxide value (PV), an indicator of primary lipid oxidation, was determined using iodometric titration. A 0.50 g aliquot of each oil sample was homogenized with 10 mL of freshly prepared acetic acid:chloroform solution (3:2, *v*/*v*) for 1 min. Following homogenization, 1 mL of potassium iodide (KI) solution (1.75 g/mL) was added, and the mixture was stirred for 1 min at ambient temperature. The reaction was then allowed to proceed for 30 min in a thermostatic water bath at 60 °C in the absence of light. After incubation, 25 mL of deionized water was added, and the mixture was vigorously shaken for 1 min. The liberated iodine was titrated with 0.1 N sodium thiosulfate solution under continuous stirring until the yellow coloration faded. At this point, 1 mL of freshly prepared 1% starch solution was added as an endpoint indicator. Titration continued until the blue coloration disappeared completely. A reagent blank was prepared and titrated in parallel under identical conditions to correct for background reactivity. The peroxide value was expressed in milliequivalents of active oxygen per kilogram of oil (meq O_2_/kg), calculated using the following formula:

(2) $$\mathrm{Peroxide}\;\mathrm{index}=\frac{\left(A-B\right)\cdot N\cdot f\cdot 1000}{P}$$

A = volume of 0.1 N sodium thiosulfate solution used in sample titration (mL)

B = volume of 0.1 N sodium thiosulfate solution used in the blank titration (mL)

N = normality of the sodium thiosulfate solution

f = factor of the sodium thiosulfate solution

P = sample weight (g)

After this, 25 mL of deionized water was added, and the mixture was homogenized for 1 min. The solution was titrated with 0.1 N sodium thiosulfate solution until the yellow color disappeared under constant stirring. Then, 1 mL of 1% starch indicator solution was added, and the titration continued until the blue color disappeared. A blank test was performed under the same titration conditions.

#### 4.2.3. Iodine Index Evaluation

The iodine value, an indicator of the degree of unsaturation in lipid molecules and a critical parameter in assessing susceptibility to oxidation, was determined using the Wijs method. A 0.25 g aliquot of each oil sample was dissolved in 10 mL of carbon tetrachloride (CCl_4_) under gentle mixing. Subsequently, 25 mL of freshly prepared Wijs solution—comprising 1.3% iodine in 75% glacial acetic acid with deionized water—was added. The reaction mixture was gently swirled using rotational movements to ensure homogeneity, then stored in the dark at room temperature for 30 min to allow halogenation of double bonds. Following the reaction period, 10 mL of 15% potassium iodide (KI) solution and 100 mL of deionized water were added to the flask. The liberated iodine was titrated with standardized 0.1 mol/L sodium thiosulfate solution until a pale-yellow coloration persisted. At this point, 2 mL of 1% starch indicator solution was added, and titration continued until the blue color fully disappeared, indicating the endpoint. A reagent blank, prepared without the oil sample, was analyzed in parallel to correct for background iodine reactivity. The iodine value was calculated according to the following equation:

$$\mathrm{Iodine}\;\mathrm{index}=\frac{\left(VB-VA\right)\cdot M\cdot 12.68}{P}$$

### 4.3. Strains

The certified strains of *Staphylococcus aureus* ATCC 6538, *Escherichia coli* ATCC 8739, *Salmonela choleraesuis* ATCC 10708, *Pseudomonas aeruginosa* ATCC 9027, *Candida albicans* ATCC 10231, *Aspergillus brasiliensis* ATCC 16404, and *Malassezia furfur* ATCC 14521 were employed in this study.

### 4.4. Agar Diffusion–Pour Plate

The antimicrobial efficacy of ozonized sunflower oil was evaluated against representative Gram-positive and Gram-negative bacteria (*Staphylococcus aureus*, *Escherichia coli*), as well as fungal strains (*Candida albicans* and *Aspergillus brasiliensis*), all obtained from certified microbial collections. Bacterial strains were cultured in tryptic soy broth (TSB) and incubated at 35 ± 2 °C for 24 h. Fungal strains were cultured in Sabouraud dextrose broth and incubated at 25 ± 2 °C for 5 days. Following incubation, each microbial suspension was adjusted to a turbidity equivalent to 0.5 McFarland standard, and serial 10-fold dilutions were performed up to 10^7^ in sterile phosphate-buffered saline (PBS). For viable count analysis, 1.0 mL aliquots from each dilution were mixed with molten culture media maintained at 45 ± 2 °C. Tryptic soy agar (TSA) was used for bacteria, and Sabouraud dextrose agar (SDA) for fungi. The mixtures were poured into sterile Petri dishes, allowed to solidify, and then incubated in an inverted position. Plates were incubated for 48 h at 35 ± 2 °C for bacterial strains and at 25 ± 2 °C for fungal strains. Microbial growth was quantified by colony-forming unit (CFU) counts, and antimicrobial activity was inferred by comparing treated versus untreated control plates. According to The Brazilian pharmacopeia, 6th edition, 2019, The United States pharmacopeia, 45th ed., 2024, and Jongensen et al. [35].

### 4.5. Time-Kill Evaluation

The bactericidal and fungicidal kinetics of ozonized sunflower oil were assessed using a time-kill assay. A 1 mL aliquot of standardized microbial suspension—containing 10^8^ to 10^9^ CFU/mL for bacterial strains and 10^6^ to 10^7^ CFU/mL for yeast strains—was inoculated into 49 mL of OSO. For inoculum control, an equivalent volume of each microbial suspension was added to 49 mL of sterile distilled water. Serial 10-fold dilutions were performed, and dilutions of 10^−3^ to 10^−6^ were plated in duplicate. At designated contact times of 30 and 60 s, 100 µL aliquots were withdrawn from the OSO-microbial suspensions, immediately transferred to tubes containing 8.8 mL of neutralizing broth and 1.1 mL of sterile water, and vigorously mixed to halt the oxidative action of the oil.

After the final timepoint, tubes were incubated at 35 ± 2 °C for 5 min. Serial decimal dilutions were again performed, and aliquots were plated on appropriate agar media. Bacterial samples were incubated on tryptic soy agar (TSA) at 35 ± 2 °C for 48 h, while fungal samples were incubated on Sabouraud dextrose agar (SDA) at 25 ± 2 °C for 5 days. Colony-forming units (CFUs) were enumerated to determine microbial viability at each timepoint. The evaluation method employed following [36].

### 4.6. Minimum Inhibitory Concentration Evaluation

Minimum inhibitory concentration testing was conducted to determine the lowest concentration of OSO capable of inhibiting visible microbial growth. From the initial microbial suspensions, 1 mL was transferred into 10 mL of 0.85% sterile saline and serially diluted to 10^−3^, 10^−4^, and 10^−5^. Aliquots of 1 mL from each dilution were placed into sterile Petri dishes, followed by the addition of 25 mL of molten, water bath-maintained agar media. Tryptic soy agar (TSA) was used for bacterial strains, and Sabouraud dextrose agar (SDA) for fungal strains. After solidification, TSA plates were incubated at 35 ± 2 °C for 48 h and SDA plates at 25 ± 2 °C for 5 days. Colony counts were used to assess microbial inhibition at each concentration. To rule out any inherent antimicrobial activity of the vehicle, preserved (non-ozonized) sunflower oil (OG) was tested independently and confirmed to have no inhibitory effect. MIC testing of OSO was subsequently conducted using serial dilutions in OG at the following concentrations: 50%, 25%, 12.5%, 6.25%, 3.125%, and 1.56% (*v*/*v*). The lowest concentration showing complete inhibition of visible colony growth was recorded as the MIC. The analysis was carried out according to ISO 20776-1:2019 [37]

### 4.7. Cytotoxicity Assessment

The cytotoxic potential of ozonized sunflower oil was evaluated using the Balb/c 3T3 clone 31 fibroblast cell line (Rio de Janeiro Cell Bank, batch 001242, Rio de Janeiro, Brazil). Cells were cultured in Dulbecco’s Modified Eagle’s Medium (DMEM), supplemented with 10% fetal bovine serum (FBS), 1% penicillin-streptomycin, and maintained at 37 °C in a humidified atmosphere with 5% CO_2_. Following thawing and stabilization, cells were seeded into 96-well plates at a density of 1 × 10^4^ cells/well and incubated for 24 h under standard culture conditions. OSO was diluted in serum-free DMEM to final concentrations of 10, 1, 0.1, 0.001, and 0.0001 mg/mL. Negative controls received only supplemented culture medium, while positive controls were treated with sodium lauryl sulfate (SLS) 100 µg/mL diluted in supplemented medium. Cell viability was assessed using the Neutral Red Uptake (NRU) assay, which measures the ability of viable cells to incorporate the neutral red dye into their lysosomes. After exposure to the test compounds, cells were incubated with neutral red solution, followed by dye extraction; the absorbance was measured spectrophotometrically at 540 nm as an indicator of cell viability.

Each well received 100 μL of the test solution or control and was incubated for an additional 24 h at 37 °C and 5% CO_2_. After incubation, wells were gently washed with deionized water, and cell viability was assessed using the neutral red uptake assay. A 100 μL aliquot of 0.05 mg/mL neutral red solution was added per well, followed by 3 h of incubation under the same conditions. After removal of the dye and cell washing, the incorporated dye was solubilized, and optical density was measured at 540 nm using a microplate reader. All according to ISO 993-5:2009 [38]. All experiments were performed in quadruplicate, and results were expressed as percentage viability relative to the negative control group.

## Figures and Tables

**Table 1:** Chemical components identified in CG-MS analysis. All values are given as the relative percentage (%) of total components.

Compound	SO (%)	100 min (%)	240 min (%)	480 min (%)
	Native fatty acids			
Palmitic acid	10.81	9.77	9.84	9.91
Stearic acid	6.12	10.01	9.90	9.52
Oleic acid	33.10	23.69	25.92	10.84
Linoleic acid	47.13	40.53	31.15	5.12
Behenic acid	1.06	2.19	2.10	1.57
Lauric acid	ND	0.83	0.81	1.40
Arachidic acid	ND	0.99	1.01	0.68
Myristic acid	ND	0.40	ND	0.63
Lignoceric acid	ND	0.58	0.57	0.43
	Ozonation-derived compounds			
Nonanoic acid, 9-oxo-		1.27	2.72	7.31
Azelaic acid		2.67	3.85	8.19
Nonanoic acid, methyl ester		0.67	0.93	5.70
2-Nonenal, (E)-		ND	0.30	1.77
Dimethyl acetal nonanal		0.80	0.98	19.64
Dimethyl acetal decanal		2.11	2.61	7.92
2-oxo-9-dodecenoate methyl ester		0.36	1.06	1.10
5,13-Docosadienoate methyl ester		2.23	4.02	2.60
3-Nonenoic acid		0.90	1.62	ND
Caprylic acid		ND	0.12	0.46
9-Decen-2-ol		ND	ND	3.65
Octanoic acid, 6,6-dimethoxy		ND	ND	1.11
2-Octanol acetate		ND	ND	0.45

ND: Not Detected.

**Table 2:** Reduction in microorganisms following exposure to an antimicrobial agent at different time intervals. The reduction factor represents the efficacy of the antimicrobial agent in the tested microorganisms.

Microorganism	Time 0	0.5 min (CFU)	1 min (CFU)	2 min (CFU)	%RF—0.5 min	%RF—1 min	%RF—2 min
*P. aeruginosa* ATCC 9027	1.2 × 10^7^	3.2 × 10^3^	1.5 × 10^3^	9.5 × 10^2^	>99.9%	>99.9%	>99.9%
*E. coli* ATCC 8739	1.9 × 10^7^	1.1 × 10^5^	8.5 × 10^4^	5.5 × 10^3^	>99%	>99%	>99.9%
*S. aureus* ATCC 6538	1.5 × 10^7^	2.4 × 10^4^	1.6 × 10^4^	3.5 × 10^3^	>99%	>99%	>99.9%
*S. choleraesuis* ATCC 10718	6.0 × 10^7^	3.5 × 10^4^	9.5 × 10^2^	1.4 × 10^2^	>99.9%	>99.99%	>99.999%
*A. brasiliensis* ATCC 16404	5.0 × 10^5^	<10	<10	-	>99.9%	>99.99%	-
*C. albicans* ATCC 10231	2.7 × 10^5^	4.2 × 10^2^	<10	-	>99%	>99%	-

CFU = colony forming unity.

**Table 3:** Minimum inhibitory concentration of ozonized sunflower oil is 480 min. Data is shown as a percentage (%) of microdilution.

Microorganism	100%	50%	25%	12.5%	6.25%	3.12%	1.56%	Control
*Staphylococcus aureus* ATCC 6538	-	-	-	-	+	+	+	-
*Escherichia coli* ATCC 8739	-	-	+	+	+	+	+	-
*Aspergillus brasiliensis* ATCC 16404	-	-	-	+	+	+	+	-
*Candida albicans* ATCC 10231	-	-	-	-	+	+	+	-

(+): Growing microorganisms in the described concentration; (-): Inhibition of microorganisms in the described concentration.

## Data Availability

The original contributions presented in the study are included in the article. All other information can be requested by the corresponding author.

## Supplementary Materials

The following supporting information can be downloaded at: https://www.mdpi.com/article/10.3390/ijms26189156/s1.

## Abbreviations

The following abbreviations are used in this manuscript:

OSO	Ozonized sunflower oil
SO	(Non-ozonized) sunflower oil (control group)
O_3_	Ozone
UV	Ultraviolet
FDA	Food and Drug Administration
GC-MS	Gas chromatography–mass spectrometry
GC-MS/MS	Gas chromatography coupled with tandem mass spectrometry
CFU	Colony-forming unit
MIC	Minimum inhibitory concentration
PBS	Phosphate-buffered saline
TSB	Tryptic soy broth
TSA	Tryptic soy agar
SDA	Sabouraud dextrose agar
FBS	Fetal bovine serum
DMEM	Dulbecco’s Modified Eagle’s Medium
SLS	Sodium lauryl sulfate
ROS	Reactive oxygen species
FAMEs	Fatty acid methyl esters
PV	Peroxide value
RF	Reduction factor
CCl_4_	Carbon tetrachloride
KI	Potassium iodide
NaOH	Sodium hydroxide
m/z	Mass-to-charge ratio
