# Supplementary material for: Ozonized Sunflower Oil: Standardization and Mechanisms of the Antimicrobial Effect
Source: Int J Mol Sci. 2025 Sep 19;26(18):9156. doi: 10.3390/ijms26189156 (PMC12470628; doi:10.3390/ijms26189156)

Centro Integrado de Análises - CIA-FURG
Laboratório de Cromatografia Gasosa - GC-MS/MS

Visite o nosso site - www.cia.furg.br

Relatório de análise - GC-MS/MS

Sample Information

Analyzed by	: Jean Arias
Analyzed	: 12/10/2023 12:36:50
Sample Type	: Unknown
Level #	: 1
Sample Name	: 11102023_OVgirassol_100min_MI
Sample ID	:
Rack	: Rack 2
IS Amount	: [1]=1
Sample Amount	: 1
Dilution Factor	: 1
Vial #	: 13
Injection Volume	: 1.00
Data File	: C:\GCMSsolution\Data\Óleos - ácidos graxos\11102023_amostras_óleos\11102023_OVgirassol_100min_MI.qgd
Org Data File	: C:\GCMSsolution\Data\Óleos - ácidos graxos\11102023_amostras_óleos\11102023_OVgirassol_100min_MI.qgd
Method File	: C:\GCMSsolution\Data\Óleos - ácidos graxos\Matéria insaponificável - scan.qgm
Org Method File	: C:\GCMSsolution\Data\Óleos - ácidos graxos\Matéria insaponificável - scan.qgm
Report File	:
Tuning File	: C:\GCMSsolution\System\Tune1\10102023_tuning.qgt
Modified by	: Jean Arias
Modified	: 18/10/2023 14:22:24

Method

[Comment]

===== Analytical Line 1 =====

[GC-2010]

Column Oven Temp.	:80.0 °C
Injection Temp.	:250.00 °C
Injection Mode	:Split
Flow Control Mode	:Linear Velocity
Pressure	:88.5 kPa
Total Flow	:17.3 mL/min
Column Flow	:1.30 mL/min
Linear Velocity	:42.0 cm/sec
Purge Flow	:3.0 mL/min
Split Ratio	:10.0
High Pressure Injection	:ON
High Press. Inj. Pressure	:300.0 kPa
High Press. Inj. Time	:2.30 min
Carrier Gas Saver	:OFF
Splitter Hold	:OFF

Oven Temp. Program

Rate	Temperature(°C)	Hold Time(min)
-	80.0	1.00
10.00	180.0	0.00
7.00	330.0	0.00

< Ready Check Heat Unit >

Column Oven	: Yes
SPL1	: Yes
MS	: Yes

< Ready Check Detector(FTD/BID) >

< Ready Check Baseline Drift >

< Ready Check Injection Flow >

SPL1 Carrier	: Yes
SPL1 Purge	: Yes

< Ready Check APC Flow >

< Ready Check Detector APC Flow >

External Wait	:No
Equilibrium Time	:3.0 min

[GC Program]

[GCMS-TQ8050]

IonSourceTemp	:230.00 °C

Interface Temp.	:280.00 °C
Solvent Cut Time	:4.00 min
Detector Gain Mode	:Relative to the Tuning Result
Detector Gain	:1.03 kV +0.00 kV
Threshold	:0
Acquire Data without Using CID Gas(Q3Scan)	:ON

[MS Table]
--Group 1 - Event 1--

Compound Name	:
Start Time	:4.50min
End Time	:32.40min
Acq. Mode	:Q3 Scan
Event Time	:0.200sec
Scan Speed	:2500
Start m/z	:50.00
End m/z	:500.00
Q1 Resolution	: -
Q3 Resolution	: -

Sample Inlet Unit :GC

[MS Program]
Use MS Program :OFF

Peak Report TIC

Peak#	R.Time	Area	Area%	Name	IR
1	8.375	2502415	0.57	Cyclooctyl alcohol	
2	8.869	33496797	7.58	2-Octenal, 2-butyl-	
3	10.127	2073770	0.47	2-Nonenal, 2-pentyl-	
4	10.182	1814424	0.41	2-Octenal, 2-butyl-	
5	11.386	1715973	0.39	2-Nonenal, 2-pentyl-	
6	12.221	3231688	0.73	2-Propylcyclohexanol	
7	12.284	3266455	0.74	2-Propylcyclohexanol	
8	12.590	45915721	10.39	2-Nonenal, 2-pentyl-	
9	12.711	43797030	9.91	2-Octenal, 2-butyl-	
10	13.813	2259732	0.51	2-Nonenal, 2-pentyl-	
11	13.874	2303549	0.52	2-Nonenal, 2-pentyl-	
12	15.082	3205435	0.73	2-Nonenal, 2-pentyl-	
13	16.039	3909909	0.88	Pentadecanal-	
14	16.269	6688170	1.51	n-Hexadecanoic acid	
15	16.353	49378854	11.17	2-Nonenal, 2-pentyl-	
16	16.571	2579416	0.58	Cyclodecasiloxane, eicosamethyl-	
17	16.643	2504074	0.57	Octanoic acid, undec-2-enyl ester	
18	16.779	2613124	0.59	Heneicosane	
19	16.903	2757139	0.62	7,9-Di-tert-butyl-1-oxaspiro(4,5)deca-6,9-diene-2,8-dion	
20	17.843	1848941	0.42	1,2,3,3a,4a,5,6,7,8,9,9a,9b-Dodecahydrocyclopenta[def	
21	18.025	3486892	0.79	2-Methylhexacosane	
22	18.064	3178014	0.72	2,4,6-Triisopropylphenetole	
23	18.250	2773607	0.63	Phytol	
24	18.493	26944889	6.09	9,12-Octadecadienoic acid (Z,Z)-	
25	18.555	32787194	7.42	9-Octadecenoic acid, (E)-	
26	18.791	6009840	1.36	Octadecanoic acid	
27	19.250	2675705	0.61	Eicosane	
28	20.366	3123905	0.71	2H-1-benzopyran-6-ol, 3,4-dihydro-2,2-dimethyl-4-(1-me	
29	20.449	2432904	0.55	Silane, trichlorodecyl-	
30	21.297	2571302	0.58	Tetracyclo[6.1.0.0(2,4).0(5,7)]nonane, 3,3,6,6,9,9-hexae	
31	21.428	4411375	1.00	2,4-Di-t-butyladamantane-2,4-diol	
32	21.515	2379918	0.54	2H-1-benzopyran-6-ol, 3,4-dihydro-2,2-dimethyl-4-(1-me	
33	21.968	2099640	0.47	Benzoic acid, 4-(4-pentylcyclohexyl)-, 4'-cyano[1,1'-biph	
34	23.120	2239301	0.51	4(1H)-Phenanthrenone, 7-(acetyloxy)-2,3,4a,9,10,10a-h	
35	23.591	2064263	0.47	2H-1-benzopyran-6-ol, 3,4-dihydro-2,2-dimethyl-4-(1-me	
36	23.798	1860282	0.42	Pentacosane	
37	24.375	1699979	0.38	(6aS,10aS)-9-(Hydroxymethyl)-6,6-dimethyl-3-(2-methyl	
38	24.767	1791374	0.41	Silane, methylvinyl(hept-4-yloxy)decyloxy-	
39	26.049	2267623	0.51	Podocarpa-8,11,13-triene-1,3-dione, 13-isopropyl-12-me	
40	26.831	2028494	0.46	Dotriacontane	
41	28.964	3719890	0.84	Stigmasta-3,5-diene	
42	30.178	12043841	2.72	Campesterol	
43	30.459	5544113	1.25	Stigmasterol	
44	30.774	11008695	2.49	Obtusifoliol	
45	30.887	1995732	0.45	Dodecanoic acid, 1,2,3-propanetriyl ester	
46	30.977	55805280	12.62	.gamma.-Sitosterol	
47	31.463	9503220	2.15	Stigmast-7-en-3-ol, (3.beta.,5.alpha.,24S)-	
48	31.639	2783513	0.63	9,19-Cyclolanost-24-en-3-ol, (3.beta.)-	
49	31.781	2145182	0.49	24-Norursa-3,12-diene	
50	32.159	10864785	2.46	17.beta.-Methyl-18-nor-17-isopregna-4,13-dien-16.beta.	
		442103368	100.00		

Chromatogram 11102023_OVgirassol_100min_MI C:\GCMSsolution\Data\Óleos - ácidos graxos\11102023_amostras_óleos\11102023_OVgirassol_100min_MI.qgd

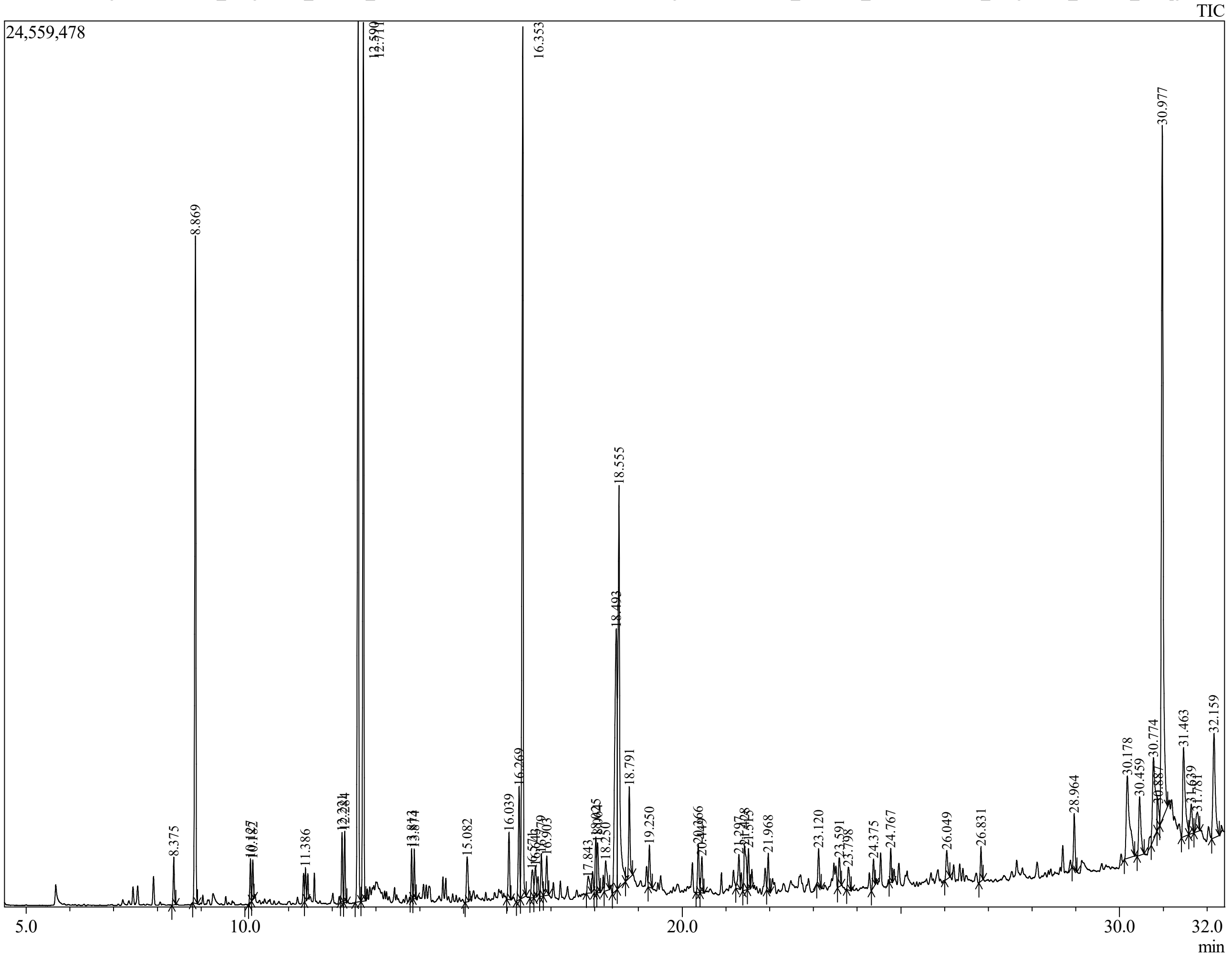

Library

<< Target >>
Line#:1   R.Time:8.373(Scan#:1163) MassPeaks:283
RawMode:Averaged 8.370-8.377(1162-1164) BasePeak:57.05(230945)
BG Mode:Calc. from Peak Group 1 - Event 1 Q3 Scan

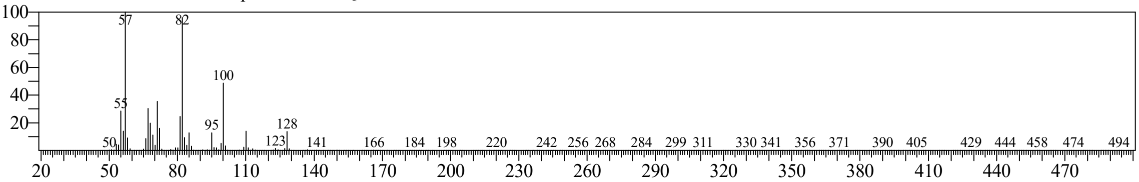

Hit#:1 Entry:6249 Library:NIST17s.lib
SI:86 Formula:C8H16O CAS:696-71-9 MolWeight:128 RetIndex:1147
CompName:Cyclooctyl alcohol $$ Cyclooctanol $$

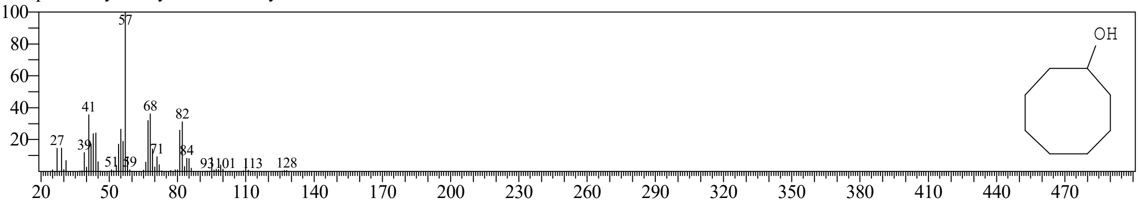

<< Target >>
Line#:2 R.Time:8.870(Scan#:1312) MassPeaks:317
RawMode:Averaged 8.867-8.873(1311-1313) BasePeak:55.05(1587688)
BG Mode:Calc. from Peak Group 1 - Event 1 Q3 Scan

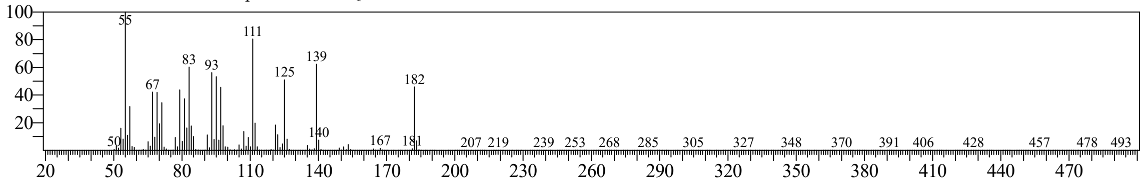

Hit#:1 Entry:18037 Library:NIST17s.lib
SI:95 Formula:C12H22O CAS:13019-16-4 MolWeight:182 RetIndex:1388
CompName:2-Octenal, 2-butyl- $$ 2-Butyl-2-octenal $$ 2-n-Butyloct-2-enal $$

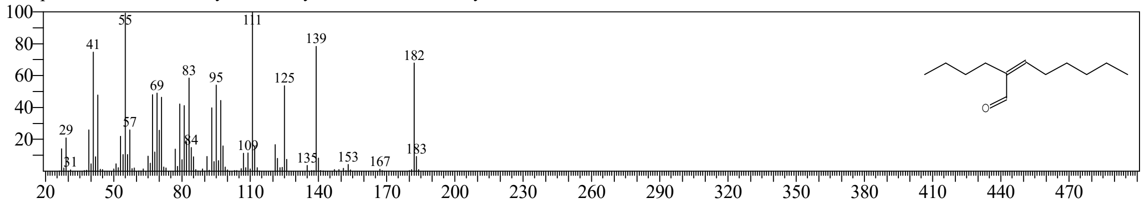

<< Target >>
Line#:3   R.Time:10.127(Scan#:1689) MassPeaks:260
RawMode:Averaged 10.123-10.130(1688-1690) BasePeak:55.05(87229)
BG Mode:Calc. from Peak Group 1 - Event 1 Q3 Scan

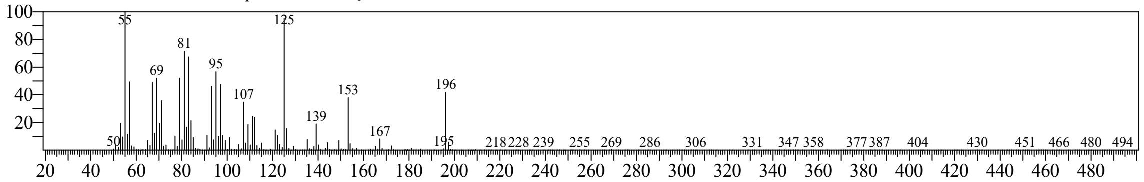

Hit#:1 Entry:58137 Library:NIST17-1.lib
SI:85 Formula:C14H26O CAS:3021-89-4 MolWeight:210 RetIndex:1586
CompName:2-Nonenal, 2-pentyl- $$ 2-Amylnon-2-enal $$ 2-Pentyl-2-nonenal $$ 2-Pentylnon-2-enal $$

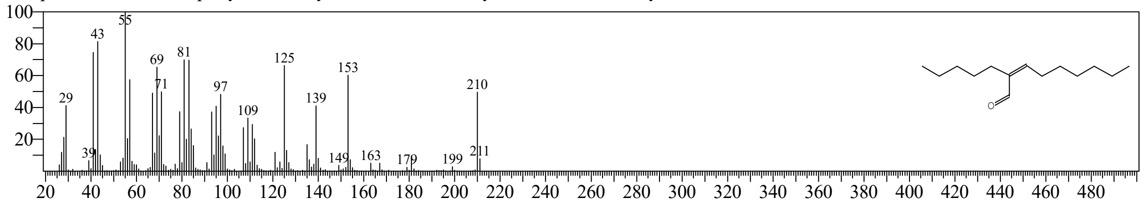

<< Target >>
Line#:4 R.Time:10.180(Scan#:1705) MassPeaks:252
RawMode:Averaged 10.177-10.183(1704-1706) BasePeak:55.05(91197)
BG Mode:Calc. from Peak Group 1 - Event 1 Q3 Scan

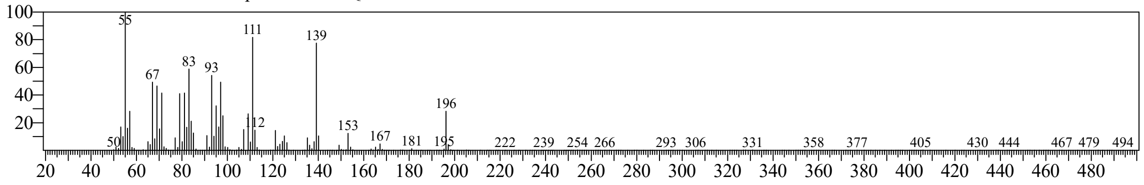

Hit#:1 Entry:18037 Library:NIST17s.lib
SI:88 Formula:C12H22O CAS:13019-16-4 MolWeight:182 RetIndex:1388
CompName:2-Octenal, 2-butyl- $$ 2-Butyl-2-octenal $$ 2-n-Butyloct-2-enal $$

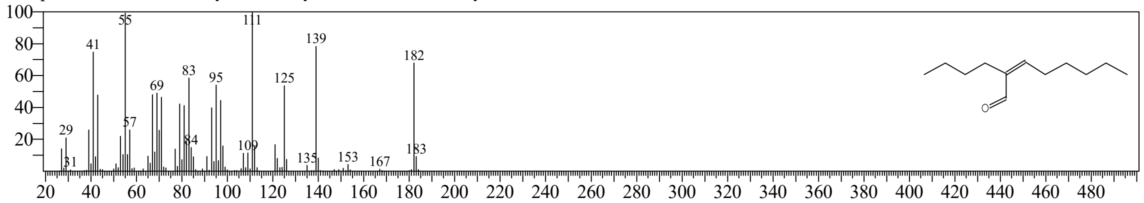

<< Target >>
Line#:5 R.Time:11.387(Scan#:2067) MassPeaks:214
RawMode:Averaged 11.383-11.390(2066-2068) BasePeak:55.05(49411)
BG Mode:Calc. from Peak Group 1 - Event 1 Q3 Scan

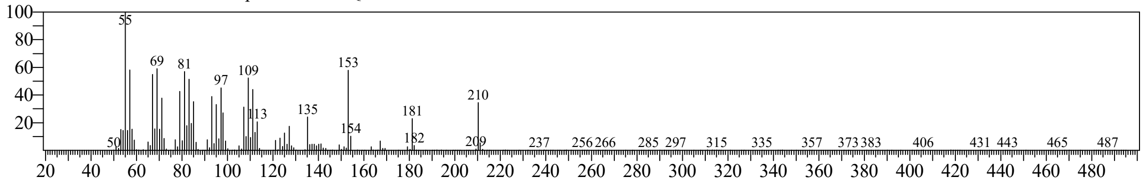

Hit#:1 Entry:58137 Library:NIST17-1.lib
SI:86 Formula:C14H26O CAS:3021-89-4 MolWeight:210 RetIndex:1586
CompName:2-Nonenal, 2-pentyl- $$ 2-Amylnon-2-enal $$ 2-Pentyl-2-nonenal $$ 2-Pentylnon-2-enal $$

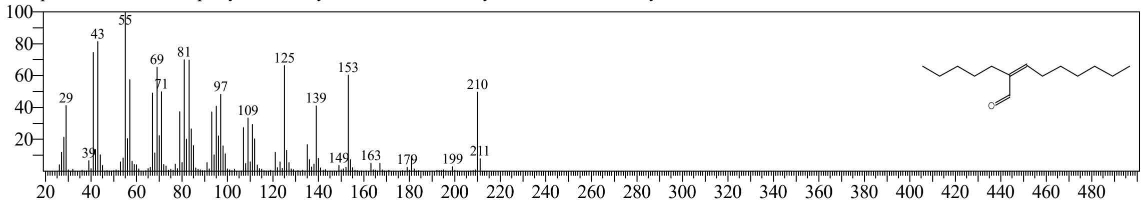

<< Target >>
Line#:6 R.Time:12.220(Scan#:2317) MassPeaks:298
RawMode:Averaged 12.217-12.223(2316-2318) BasePeak:82.05(209147)
BG Mode:Calc. from Peak Group 1 - Event 1 Q3 Scan

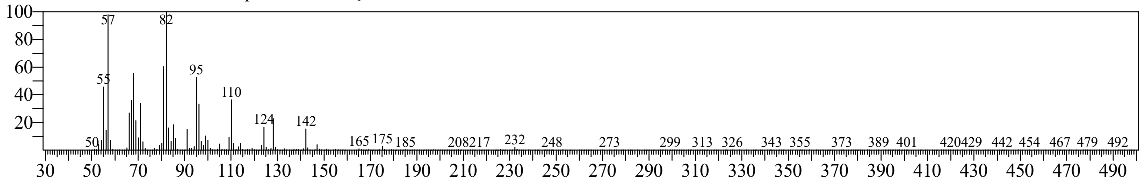

Hit#:1 Entry:9184 Library:NIST17s.lib
SI:86 Formula:C9H18O CAS:90676-25-8 MolWeight:142 RetIndex:1167
CompName:2-Propylcyclohexanol

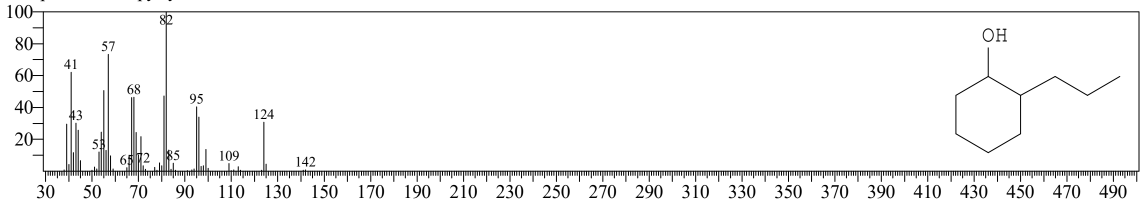

<< Target >>
Line#:7 R.Time:12.283(Scan#:2336) MassPeaks:267
RawMode:Averaged 12.280-12.287(2335-2337) BasePeak:82.05(356411)
BG Mode:Calc. from Peak Group 1 - Event 1 Q3 Scan

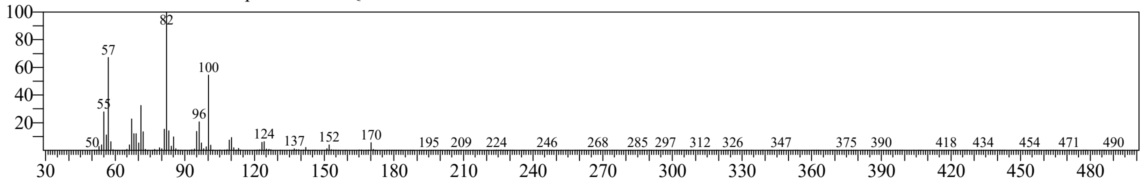

Hit#:1 Entry:9184 Library:NIST17s.lib
SI:82 Formula:C9H18O CAS:90676-25-8 MolWeight:142 RetIndex:1167
CompName:2-Propylcyclohexanol

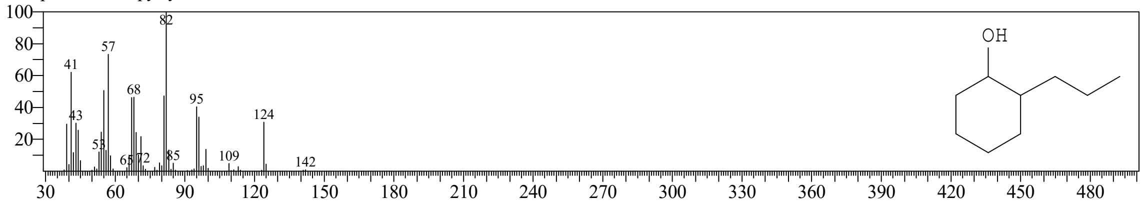

<< Target >>
Line#:8 R.Time:12.590(Scan#:2428) MassPeaks:375
RawMode:Averaged 12.587-12.593(2427-2429) BasePeak:55.05(1411595)
BG Mode:Calc. from Peak Group 1 - Event 1 Q3 Scan

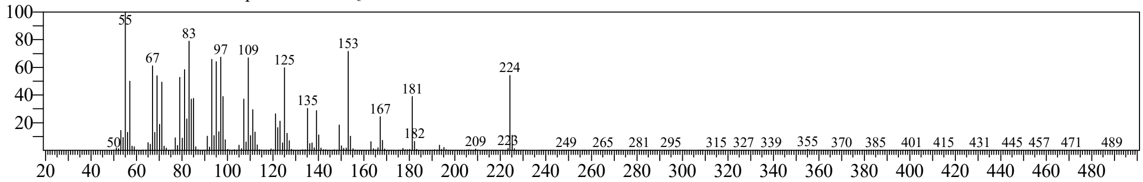

Hit#:1 Entry:58137 Library:NIST17-1.lib
SI:84 Formula:C14H26O CAS:3021-89-4 MolWeight:210 RetIndex:1586
CompName:2-Nonenal, 2-pentyl- $$ 2-Amylnon-2-enal $$ 2-Pentyl-2-nonenal $$ 2-Pentylnon-2-enal $$

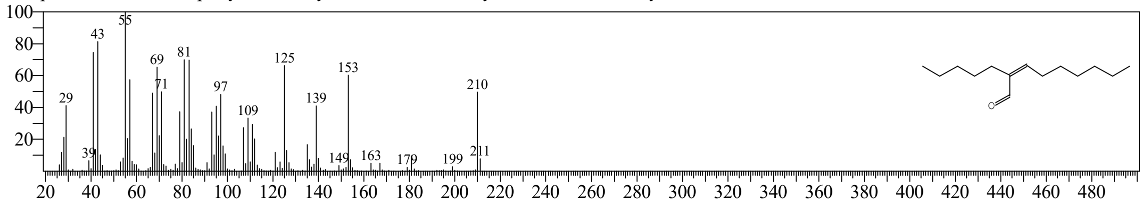

<< Target >>
Line#:9 R.Time:12.710(Scan#:2464) MassPeaks:335
RawMode:Averaged 12.707-12.713(2463-2465) BasePeak:111.05(1738015)
BG Mode:Calc. from Peak Group 1 - Event 1 Q3 Scan

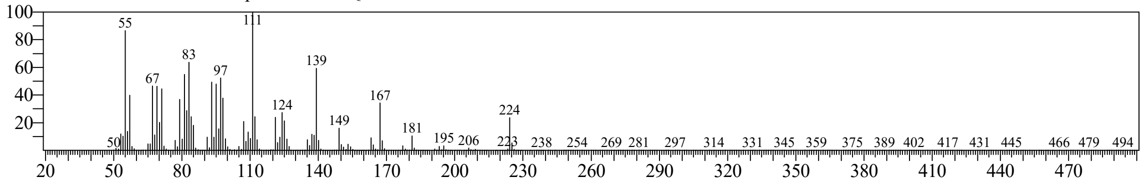

Hit#:1 Entry:18037 Library:NIST17s.lib
SI:83 Formula:C12H22O CAS:13019-16-4 MolWeight:182 RetIndex:1388
CompName:2-Octenal, 2-butyl- $$ 2-Butyl-2-octenal $$ 2-n-Butyloct-2-enal $$

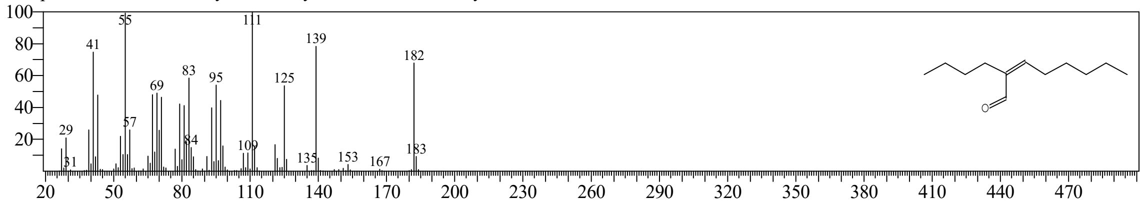

<< Target >>
Line#:10   R.Time:13.813(Scan#:2795) MassPeaks:261
RawMode:Averaged 13.810-13.817(2794-2796) BasePeak:55.05(84010)
BG Mode:Calc. from Peak Group 1 - Event 1 Q3 Scan

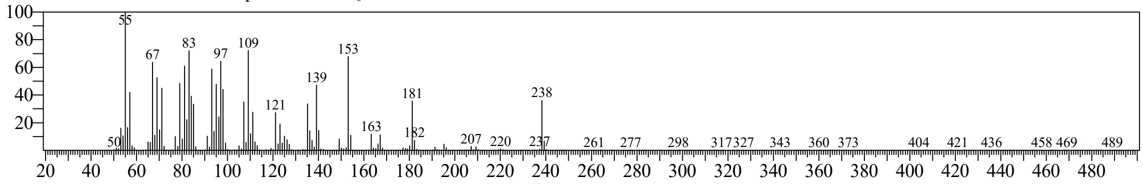

Hit#:1 Entry:58137 Library:NIST17-1.lib
SI:85 Formula:C14H26O CAS:3021-89-4 MolWeight:210 RetIndex:1586
CompName:2-Nonenal, 2-pentyl- $$ 2-Amylnon-2-enal $$ 2-Pentyl-2-nonenal $$ 2-Pentylnon-2-enal $$

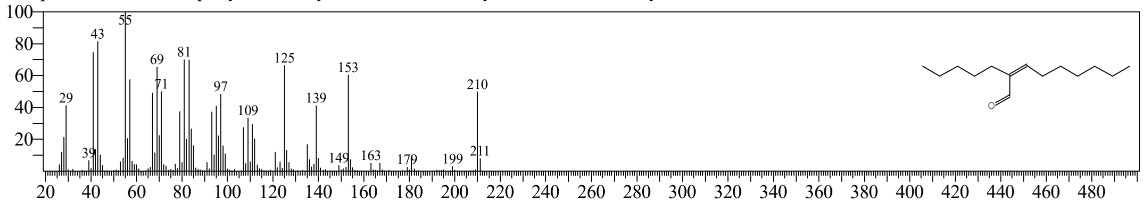

<< Target >>
Line#:11   R.Time:13.873(Scan#:2813) MassPeaks:302
RawMode:Averaged 13.870-13.877(2812-2814) BasePeak:55.05(82166)
BG Mode:Calc. from Peak Group 1 - Event 1 Q3 Scan

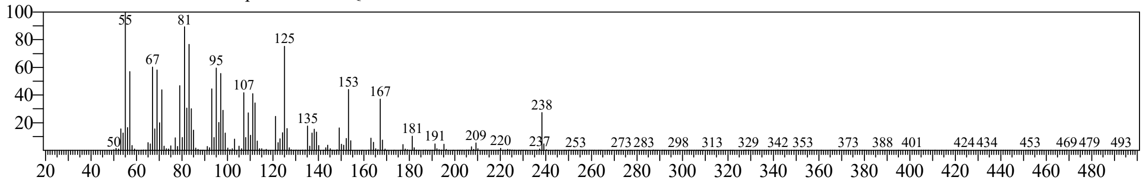

Hit#:1 Entry:58137 Library:NIST17-1.lib
SI:85 Formula:C14H26O CAS:3021-89-4 MolWeight:210 RetIndex:1586
CompName:2-Nonenal, 2-pentyl- $$ 2-Amylnon-2-enal $$ 2-Pentyl-2-nonenal $$ 2-Pentylnon-2-enal $$

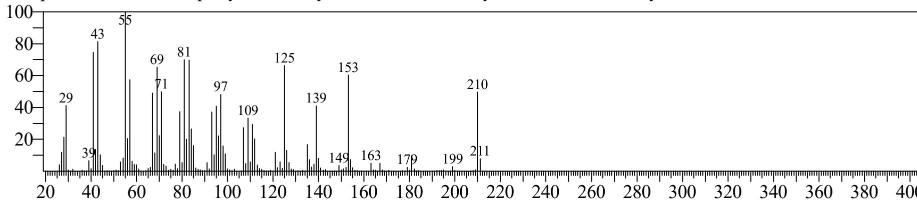

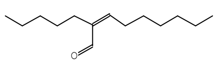

<< Target >>
Line#:12 R.Time:15.083(Scan#:3176) MassPeaks:323
RawMode:Averaged 15.080-15.087(3175-3177) BasePeak:55.05(66345)
BG Mode:Calc. from Peak Group 1 - Event 1 Q3 Scan

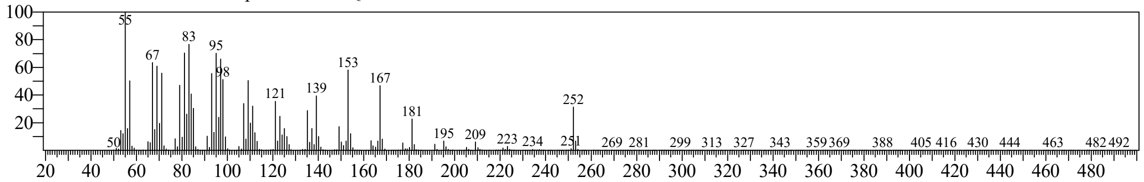

Hit#:1 Entry:58137 Library:NIST17-1.lib
SI:84 Formula:C14H26O CAS:3021-89-4 MolWeight:210 RetIndex:1586
CompName:2-Nonenal, 2-pentyl- $$ 2-Amylnon-2-enal $$ 2-Pentyl-2-nonenal $$ 2-Pentylnon-2-enal $$

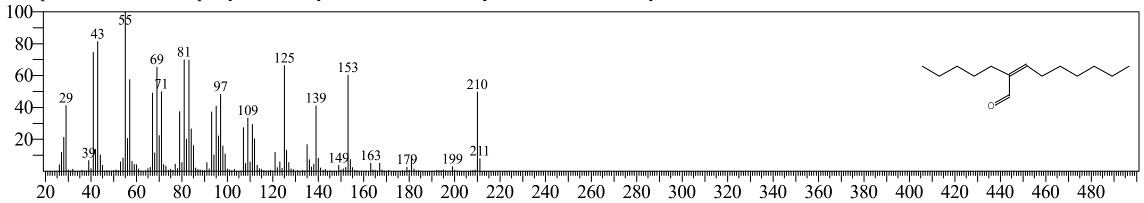

<< Target >>
Line#:13 R.Time:16.040(Scan#:3463) MassPeaks:289
RawMode:Averaged 16.037-16.043(3462-3464) BasePeak:82.05(251196)
BG Mode:Calc. from Peak Group 1 - Event 1 Q3 Scan

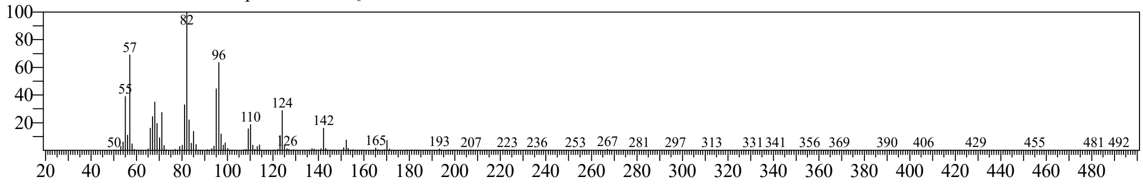

Hit#:1 Entry:25974 Library:NIST17s.lib
SI:88 Formula:C15H30O CAS:2765-11-9 MolWeight:226 RetIndex:1701
CompName:Pentadecanal- $$ 1-Pentadecanal $$ n-Pentadecanal $$

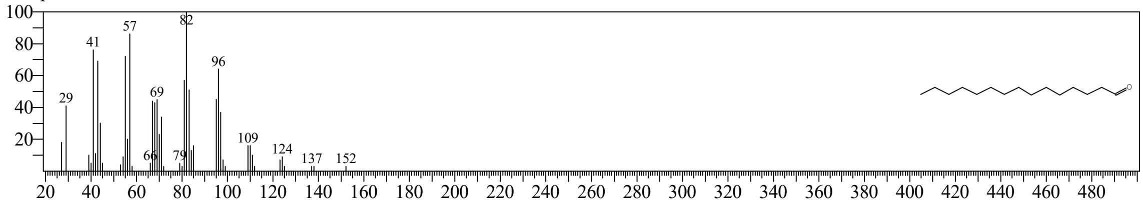

<< Target >>
Line#:14   R.Time:16.270(Scan#:3532) MassPeaks:337
RawMode:Averaged 16.267-16.273(3531-3533) BasePeak:73.05(229626)
BG Mode:Calc. from Peak Group 1 - Event 1 Q3 Scan

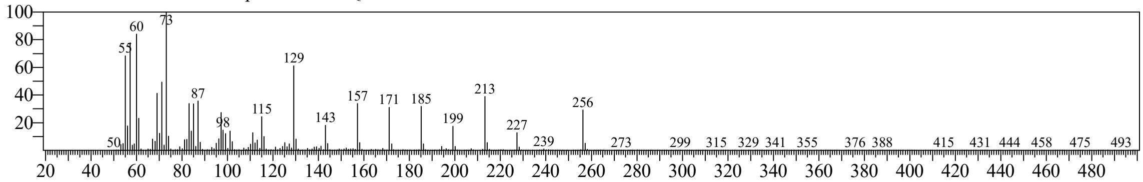

Hit#:1 Entry:29350 Library:NIST17s.lib
SI:94 Formula:C16H32O2 CAS:57-10-3 MolWeight:256 RetIndex:1968
CompName:n-Hexadecanoic acid $$ Hexadecanoic acid $$ n-Hexadecoic acid $$ Palmitic acid $$ Pentadecanecarboxylic acid $$ 1-Pentadecanecarboxylic

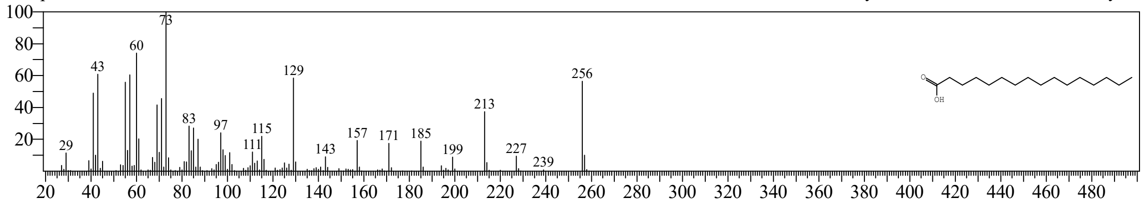

<< Target >>
Line#:15   R.Time:16.353(Scan#:3557) MassPeaks:319
RawMode:Averaged 16.350-16.357(3556-3558) BasePeak:55.05(1157921)
BG Mode:Calc. from Peak Group 1 - Event 1 Q3 Scan

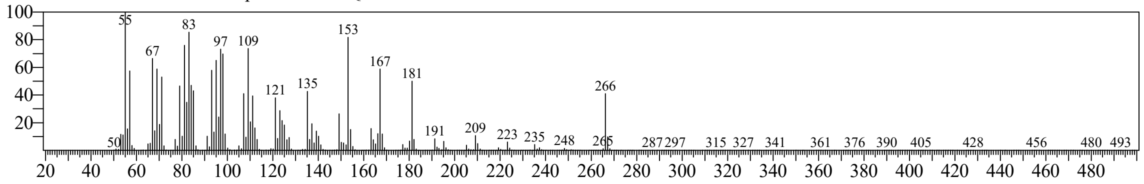

Hit#:1 Entry:58137 Library:NIST17-1.lib
SI:79 Formula:C14H26O CAS:3021-89-4 MolWeight:210 RetIndex:1586
CompName:2-Nonenal, 2-pentyl- $$ 2-Amylnon-2-enal $$ 2-Pentyl-2-nonenal $$ 2-Pentylnon-2-enal $$

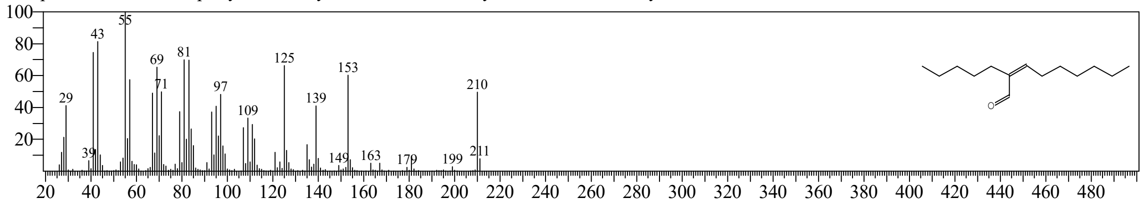

<< Target >>
Line#:16 R.Time:16.570(Scan#:3622) MassPeaks:328
RawMode:Averaged 16.567-16.573(3621-3623) BasePeak:281.05(50042)
BG Mode:Calc. from Peak Group 1 - Event 1 Q3 Scan

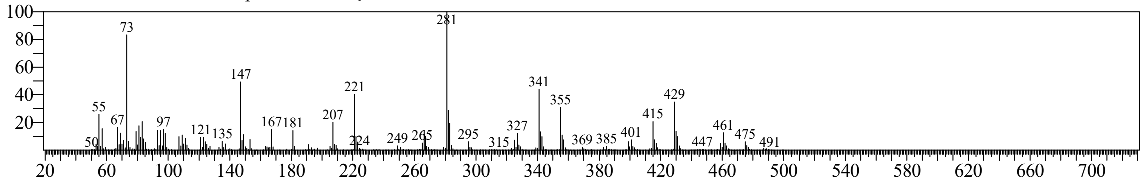

Hit#:1 Entry:9236 Library:NIST17-2.lib
SI:65 Formula:C20H60O10Si10 CAS:18772-36-6 MolWeight:740 RetIndex:2067
CompName:Cyclodecasiloxane, eicosamethyl- $$ 2,2,4,4,6,6,8,8,10,10,12,12,14,14,16,16,18,18,20,20-Icosamethylcyclodecasiloxane # $$ Eicosamethyl-cy

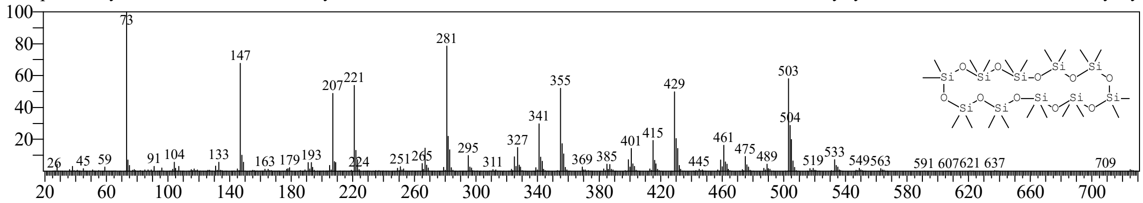

<< Target >>
Line#:17 R.Time:16.643(Scan#:3644) MassPeaks:267
RawMode:Averaged 16.640-16.647(3643-3645) BasePeak:169.10(72519)
BG Mode:Calc. from Peak Group 1 - Event 1 Q3 Scan

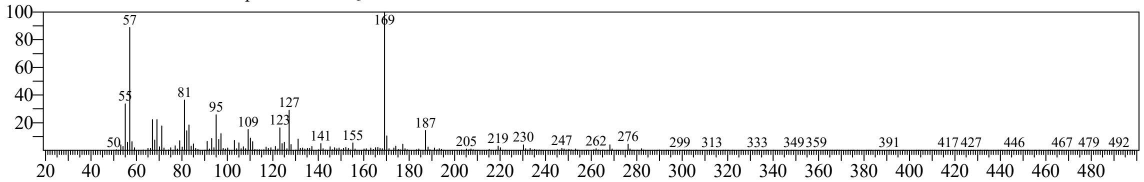

Hit#:1 Entry:139697 Library:NIST17-1.lib
SI:72 Formula:C19H36O2 CAS:0-00-0 MolWeight:296 RetIndex:2085
CompName:Octanoic acid, undec-2-enyl ester

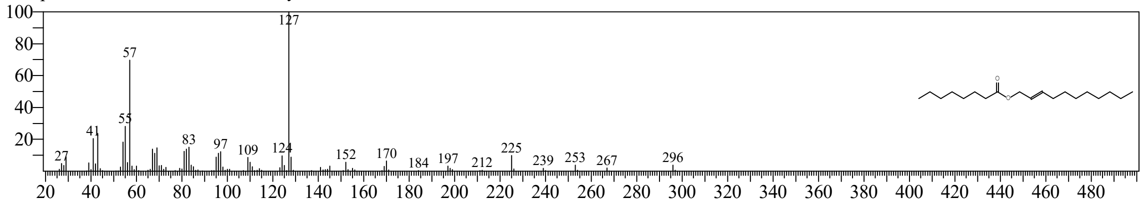

<< Target >>
Line#:18   R.Time:16.780(Scan#:3685) MassPeaks:254
RawMode:Averaged 16.777-16.783(3684-3686) BasePeak:57.10(219056)
BG Mode:Calc. from Peak Group 1 - Event 1 Q3 Scan

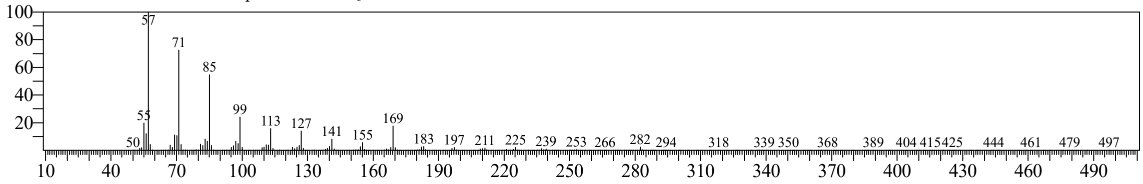

Hit#:1 Entry:32774 Library:NIST17s.lib
SI:95 Formula:C21H44 CAS:629-94-7 MolWeight:296 RetIndex:2109
CompName:Heneicosane $$ n-Heneicosane $$ Henicosane # $$

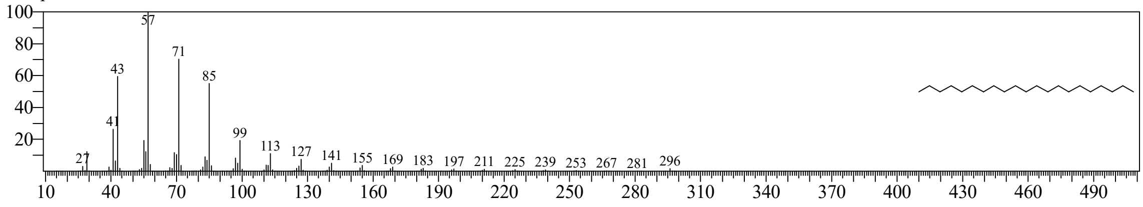

<< Target >>
Line#:19 R.Time:16.903(Scan#:3722) MassPeaks:303
RawMode:Averaged 16.900-16.907(3721-3723) BasePeak:57.10(151051)
BG Mode:Calc. from Peak Group 1 - Event 1 Q3 Scan

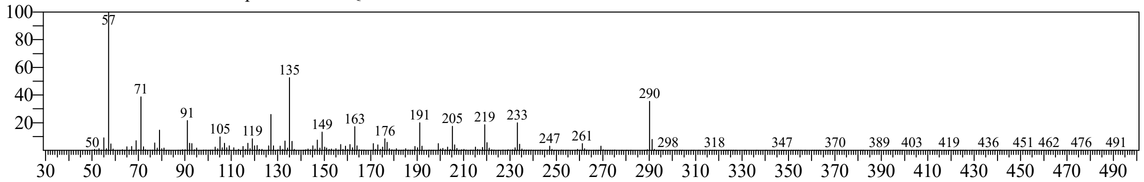

Hit#:1 Entry:31113 Library:NIST17s.lib
SI:62 Formula:C17H24O3 CAS:82304-66-3 MolWeight:276 RetIndex:2081
CompName:7,9-Di-tert-butyl-1-oxaspiro(4,5)deca-6,9-diene-2,8-dione $$ 1-Oxa-spiro[4.5]deca-6,9-diene-2,8-dione, 7,9-di-tert-butyl- $$ 7,9-Di-tert-butyl-1

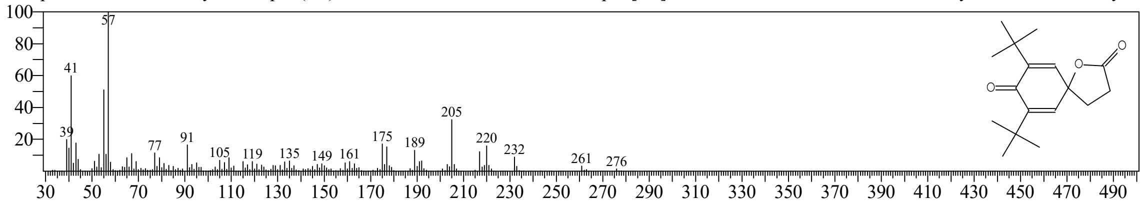

<< Target >>
Line#:20   R.Time:17.843(Scan#:4004) MassPeaks:286
RawMode:Averaged 17.840-17.847(4003-4005) BasePeak:131.05(37994)
BG Mode:Calc. from Peak Group 1 - Event 1 Q3 Scan

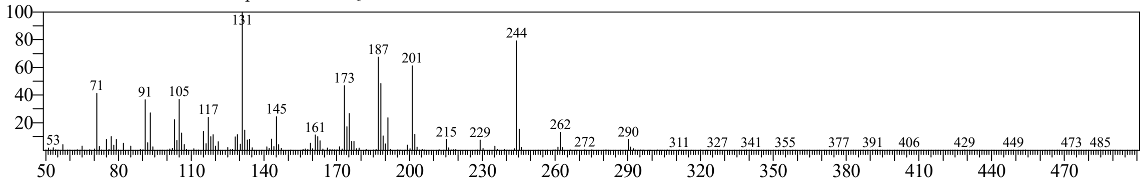

Hit#:1 Entry:51194 Library:NIST17-1.lib
SI:65 Formula:C15H22 CAS:0-00-0 MolWeight:202 RetIndex:1421
CompName:1,2,3,3a,4a,5,6,7,8,9,9a,9b-Dodecahydrocyclopenta[def]phenanthrene $$ 2,3,3a,4,4a,5,6,7,7a,8,9,9c-Dodecahydro-1H-cyclopenta[def]phenanth

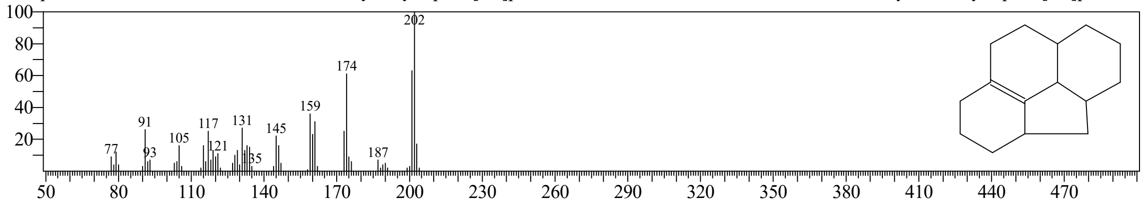

<< Target >>
Line#:21 R.Time:18.023(Scan#:4058) MassPeaks:244
RawMode:Averaged 18.020-18.027(4057-4059) BasePeak:57.10(112239)
BG Mode:Calc. from Peak Group 1 - Event 1 Q3 Scan

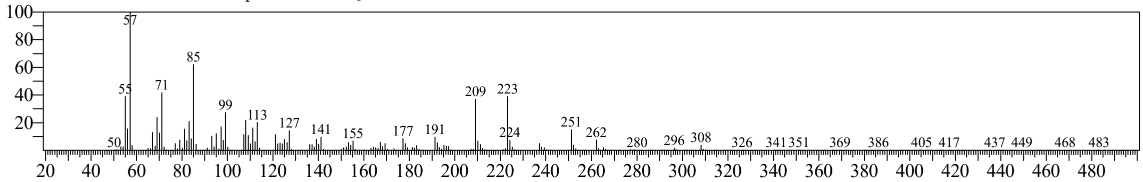

Hit#:1 Entry:215841 Library:NIST17-1.lib
SI:74 Formula:C27H56 CAS:1561-02-0 MolWeight:380 RetIndex:2641
CompName:2-Methylhexacosane $$ Hexacosane, 2-methyl- $$ 2-Methyl-n-hexacosane $$

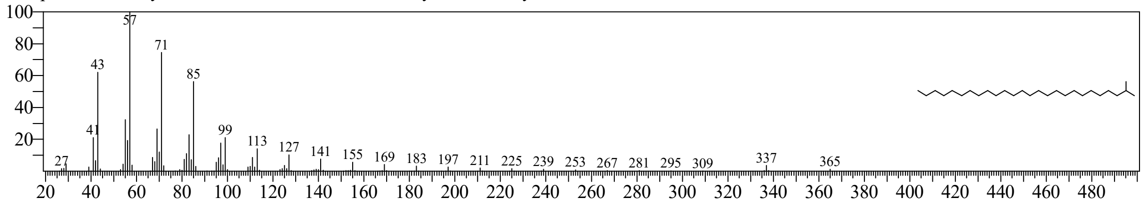

<< Target >>
Line#:22   R.Time:18.063(Scan#:4070) MassPeaks:271
RawMode:Averaged 18.060-18.067(4069-4071) BasePeak:71.10(104713)
BG Mode:Calc. from Peak Group 1 - Event 1 Q3 Scan

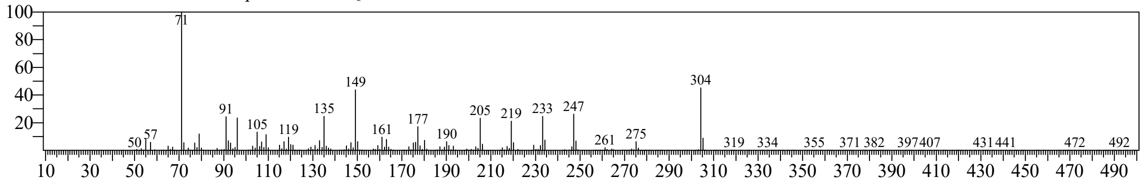

Hit#:1 Entry:92363 Library:NIST17-1.lib
SI:57 Formula:C17H28O CAS:32246-45-0 MolWeight:248 RetIndex:1713
CompName:2,4,6-Triisopropylphenetole $$ 2-Ethoxy-1,3,5-triisopropylbenzene # $$

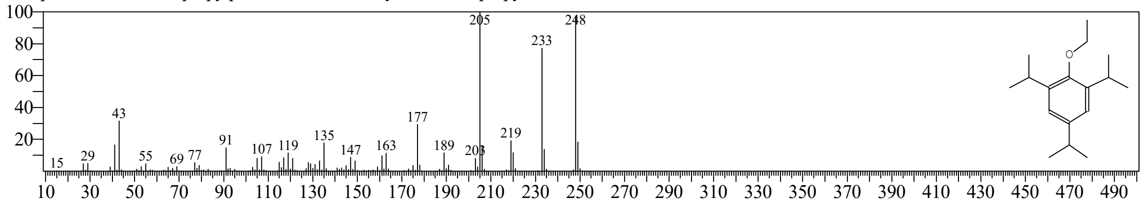

<< Target >>
Line#:23   R.Time:18.250(Scan#:4126) MassPeaks:246
RawMode:Averaged 18.247-18.253(4125-4127) BasePeak:71.05(158577)
BG Mode:Calc. from Peak Group 1 - Event 1 Q3 Scan

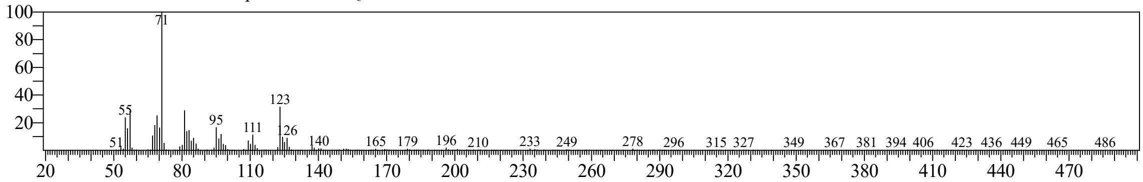

Hit#:1 Entry:139764 Library:NIST17-1.lib
SI:96 Formula:C20H40O CAS:150-86-7 MolWeight:296 RetIndex:2045
CompName:Phytol $$ 2-Hexadecen-1-ol, 3,7,11,15-tetramethyl-, [R-[R*,R*-(E)]]- $$ trans-Phytol $$ 3,7,11,15-Tetramethyl-2-hexadecen-1-ol-, (2E,7R,11F

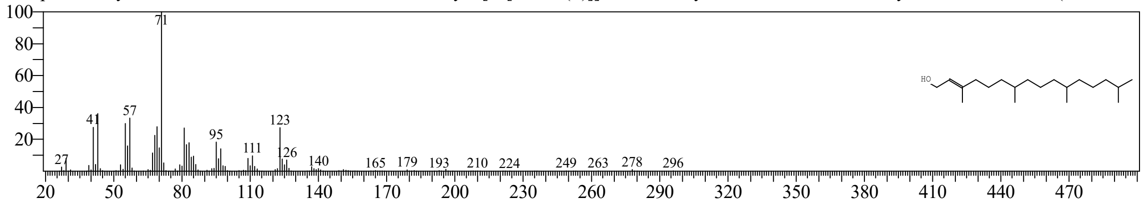

<< Target >>
Line#:24 R.Time:18.493(Scan#:4199) MassPeaks:335
RawMode:Averaged 18.490-18.497(4198-4200) BasePeak:67.05(378910)
BG Mode:Calc. from Peak Group 1 - Event 1 Q3 Scan

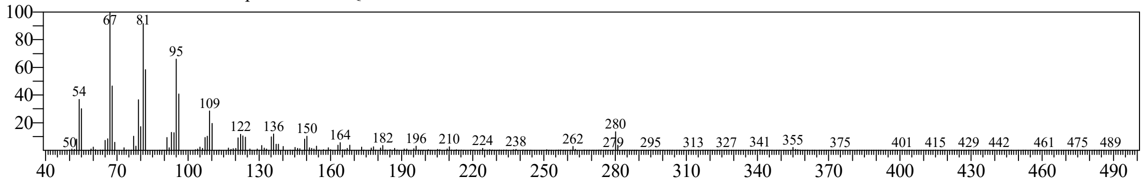

Hit#:1 Entry:31427 Library:NIST17s.lib
SI:90 Formula:C18H32O2 CAS:60-33-3 MolWeight:280 RetIndex:2183
CompName:9,12-Octadecadienoic acid (Z,Z)- $$ cis-9,cis-12-Octadecadienoic acid $$ cis,cis-Linoleic acid $$ Grape seed oil $$ Linoleic $$ Linoleic acid $

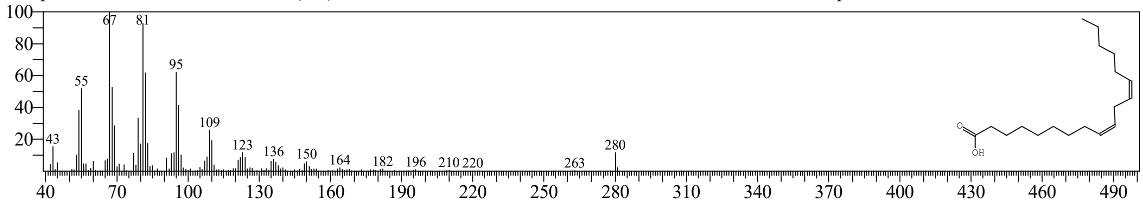

<< Target >>
Line#:25 R.Time:18.557(Scan#:4218) MassPeaks:356
RawMode:Averaged 18.553-18.560(4217-4219) BasePeak:55.05(540417)
BG Mode:Calc. from Peak Group 1 - Event 1 Q3 Scan

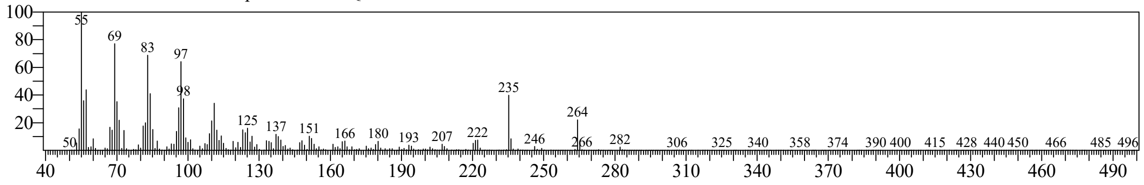

Hit#:1 Entry:31601 Library:NIST17s.lib
SI:90 Formula:C18H34O2 CAS:112-79-8 MolWeight:282 RetIndex:2175
CompName:9-Octadecenoic acid, (E)- $$ trans-.delta.(sup 9)-Octadecenoic acid $$ trans-.delta.9-Octadecenoic acid $$ trans-Octadec-9-enoic acid $$ trans-

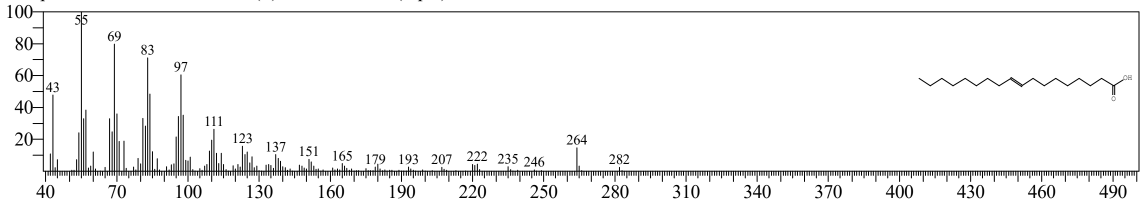

<< Target >>
Line#:26   R.Time:18.790(Scan#:4288) MassPeaks:337
RawMode:Averaged 18.787-18.793(4287-4289) BasePeak:73.05(142503)
BG Mode:Calc. from Peak Group 1 - Event 1 Q3 Scan

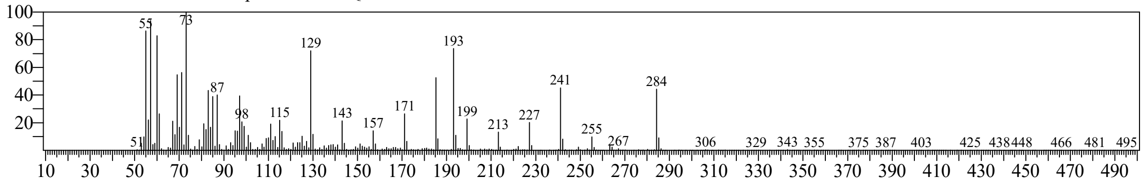

Hit#:1 Entry:127688 Library:NIST17-1.lib
SI:86 Formula:C18H36O2 CAS:57-11-4 MolWeight:284 RetIndex:2167
CompName:Octadecanoic acid $$ Stearic acid $$ n-Octadecanoic acid $$ Humko Industrene R $$ Hydrofol Acid 150 $$ Hystrene S-97 $$ Hystrene T-70 $

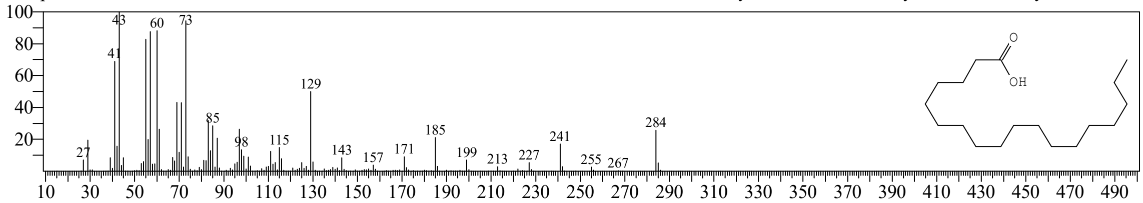

<< Target >>
Line#:27   R.Time:19.250(Scan#:4426) MassPeaks:227
RawMode:Averaged 19.247-19.253(4425-4427) BasePeak:57.05(209964)
BG Mode:Calc. from Peak Group 1 - Event 1 Q3 Scan

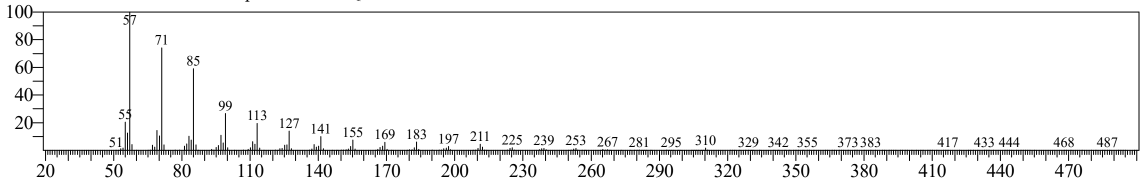

Hit#:1 Entry:125569 Library:NIST17-1.lib
SI:94 Formula:C20H42 CAS:112-95-8 MolWeight:282 RetIndex:2009
CompName:Eicosane $$ n-Eicosane $$ Icosane # $$ n-Icosane $$

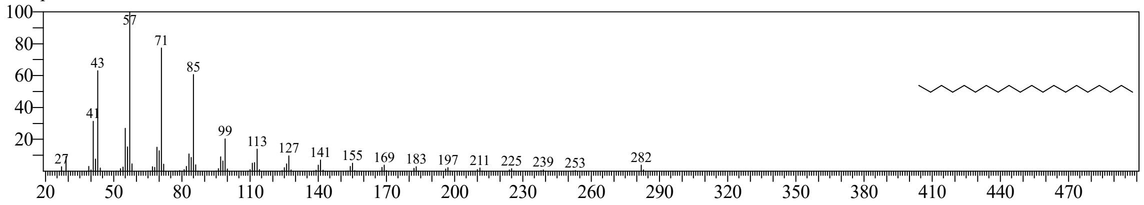

<< Target >>
Line#:28 R.Time:20.367(Scan#:4761) MassPeaks:316
RawMode:Averaged 20.363-20.370(4760-4762) BasePeak:57.10(229648)
BG Mode:Calc. from Peak Group 1 - Event 1 Q3 Scan

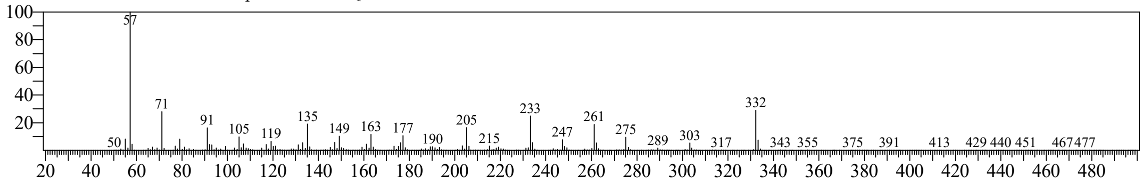

Hit#:1 Entry:176347 Library:NIST17-1.lib
SI:64 Formula:C22H36O2 CAS:0-00-0 MolWeight:332 RetIndex:2346
CompName:2H-1-benzopyran-6-ol, 3,4-dihydro-2,2-dimethyl-4-(1-methylethyl)-7-(1,1,3,3-tetramethylbutyl)- $$ 2,2-dimethyl-4-(propan-2-yl)-7-(2,4,4-trime

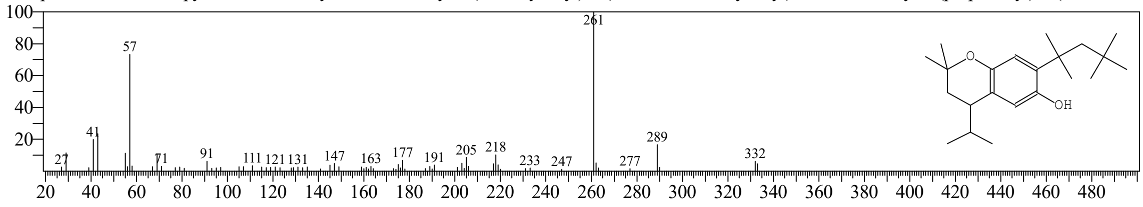

<< Target >>
Line#:29 R.Time:20.450(Scan#:4786) MassPeaks:329
RawMode:Averaged 20.447-20.453(4785-4787) BasePeak:57.10(160136)
BG Mode:Calc. from Peak Group 1 - Event 1 Q3 Scan

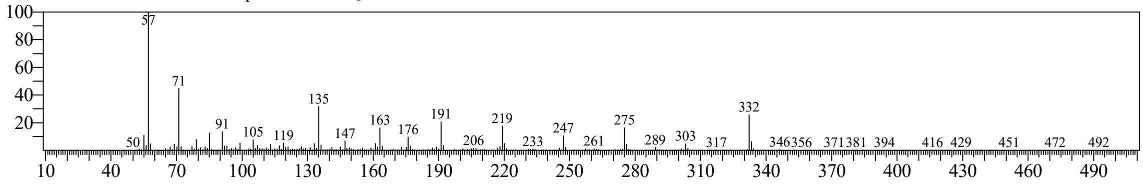

Hit#:1 Entry:116875 Library:NIST17-1.lib
SI:66 Formula:C10H21Cl3Si CAS:13829-21-5 MolWeight:274 RetIndex:1454
CompName:Silane, trichlorodecyl- $$ Decyltrichlorosilane $$ Trichloro(n-decyl)silane $$ n-Decyltrichlorosilane $$ Trichloro(decyl)silane # $$

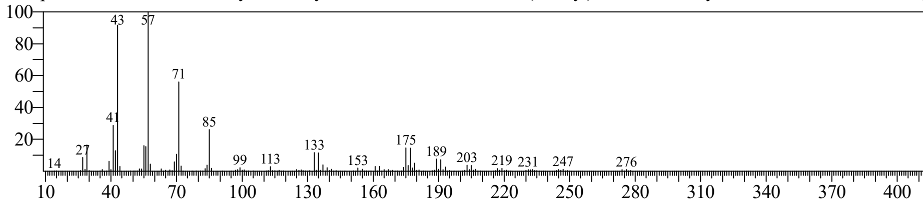

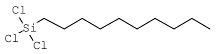

<< Target >>
Line#:30   R.Time:21.297(Scan#:5040) MassPeaks:289
RawMode:Averaged 21.293-21.300(5039-5041) BasePeak:273.15(62173)
BG Mode:Calc. from Peak Group 1 - Event 1 Q3 Scan

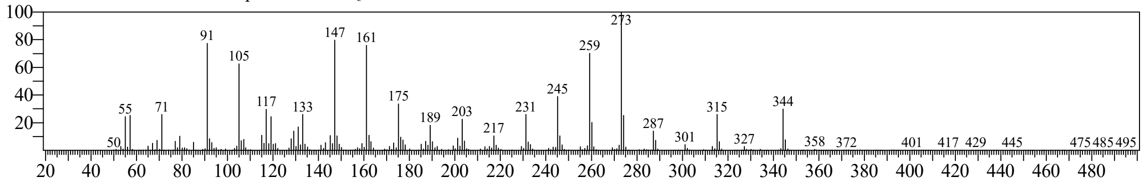

Hit#:1 Entry:131786 Library:NIST17-1.lib
SI:67 Formula:C21H36 CAS:78578-98-0 MolWeight:288 RetIndex:1663
CompName:Tetracyclo[6.1.0.0(2,4).0(5,7)]nonane, 3,3,6,6,9,9-hexaethyl-, cis,cis,trans-

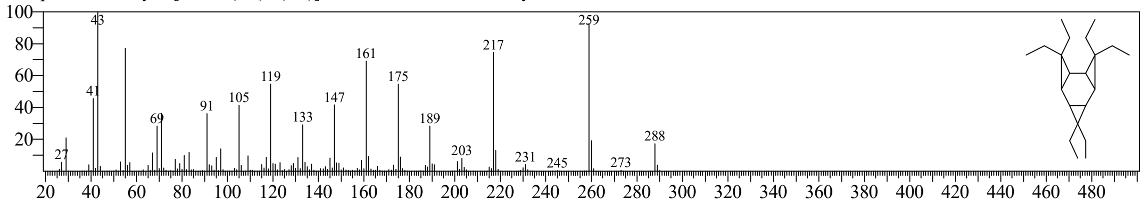

<< Target >>
Line#:31 R.Time:21.430(Scan#:5080) MassPeaks:310
RawMode:Averaged 21.427-21.433(5079-5081) BasePeak:71.10(111844)
BG Mode:Calc. from Peak Group 1 - Event 1 Q3 Scan

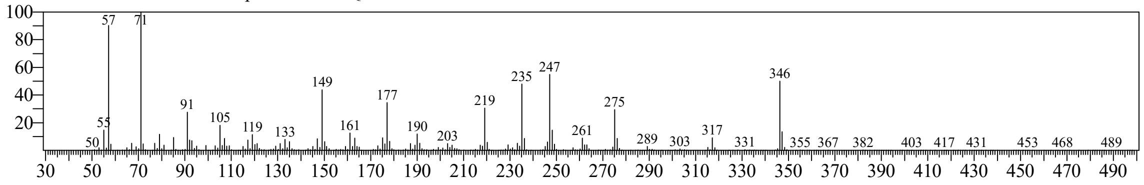

Hit#:1 Entry:123318 Library:NIST17-1.lib
SI:55 Formula:C18H32O2 CAS:132156-48-0 MolWeight:280 RetIndex:1731
CompName:2,4-Di-t-butyladamantane-2,4-diol $$ 2,4-Ditert-butyl-2,4-adamantanediol # $$

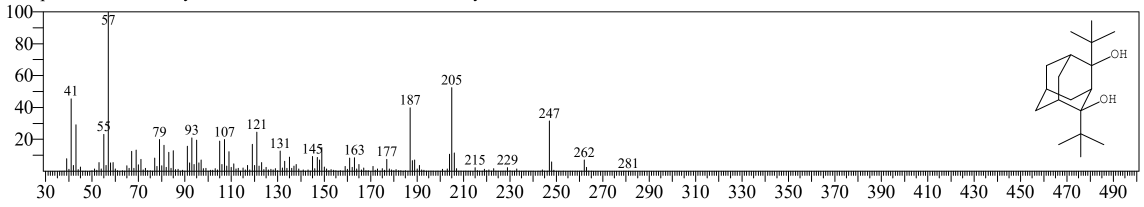

<< Target >>
Line#:32 R.Time:21.517(Scan#:5106) MassPeaks:283
RawMode:Averaged 21.513-21.520(5105-5107) BasePeak:71.10(130308)
BG Mode:Calc. from Peak Group 1 - Event 1 Q3 Scan

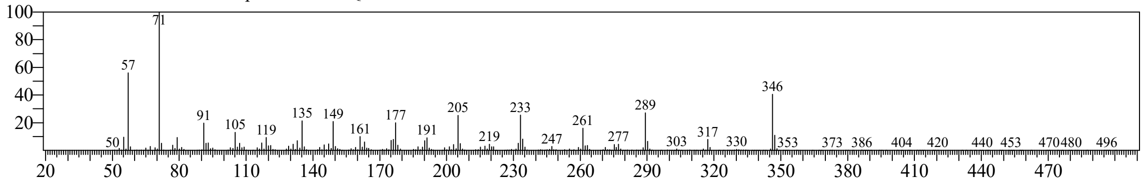

Hit#:1 Entry:176347 Library:NIST17-1.lib
SI:59 Formula:C22H36O2 CAS:0-00-0 MolWeight:332 RetIndex:2346
CompName:2H-1-benzopyran-6-ol, 3,4-dihydro-2,2-dimethyl-4-(1-methylethyl)-7-(1,1,3,3-tetramethylbutyl)- $$ 2,2-dimethyl-4-(propan-2-yl)-7-(2,4,4-trime

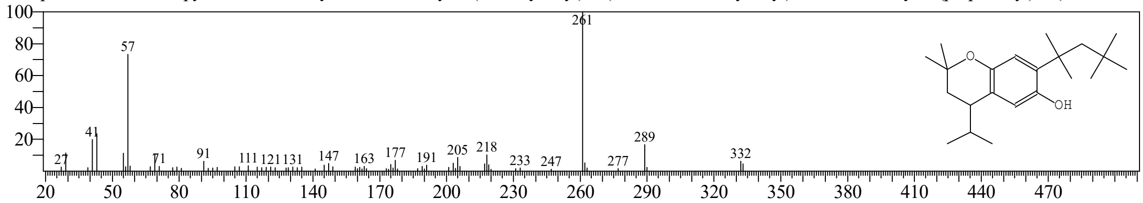

<< Target >>
Line#:33   R.Time:21.967(Scan#:5241) MassPeaks:267
RawMode:Averaged 21.963-21.970(5240-5242) BasePeak:257.15(444437)
BG Mode:Calc. from Peak Group 1 - Event 1 Q3 Scan

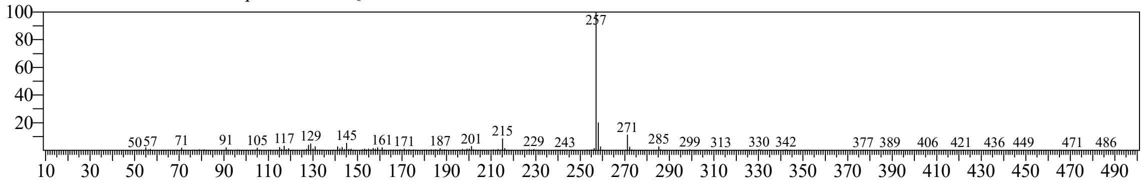

Hit#:1 Entry:247258 Library:NIST17-1.lib
SI:78 Formula:C31H33NO2 CAS:82406-82-4 MolWeight:451 RetIndex:3731
CompName:Benzoic acid, 4-(4-pentylcyclohexyl)-, 4'-cyano[1,1'-biphenyl]-4-yl ester $$ 4'-Cyano[1,1'-biphenyl]-4-yl 4-(4-pentylcyclohexyl)benzoate # $$

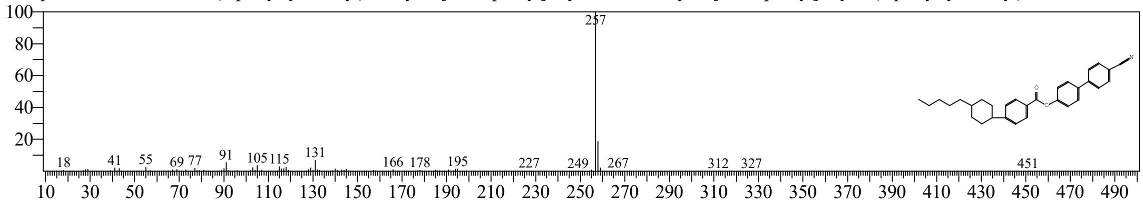

<< Target >>
Line#:34 R.Time:23.120(Scan#:5587) MassPeaks:344
RawMode:Averaged 23.117-23.123(5586-5588) BasePeak:267.20(52793)
BG Mode:Calc. from Peak Group 1 - Event 1 Q3 Scan

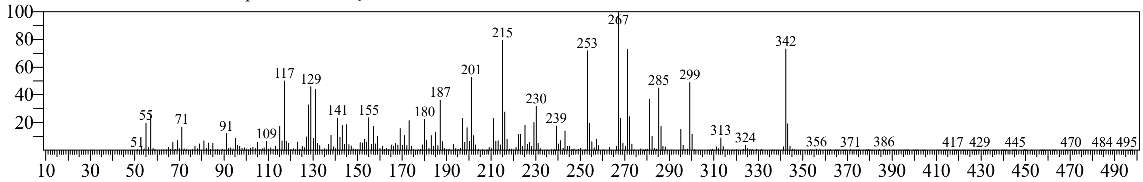

Hit#:1 Entry:186037 Library:NIST17-1.lib
SI:52 Formula:C22H30O3 CAS:57397-35-0 MolWeight:342 RetIndex:2555
CompName:4(1H)-Phenanthrenone, 7-(acetyloxy)-2,3,4a,9,10,10a-hexahydro-1,1,4a-trimethyl-8-(1-methylethyl)-, (4aS-trans)- $$ 14-Isopropyl-1-oxopodoc

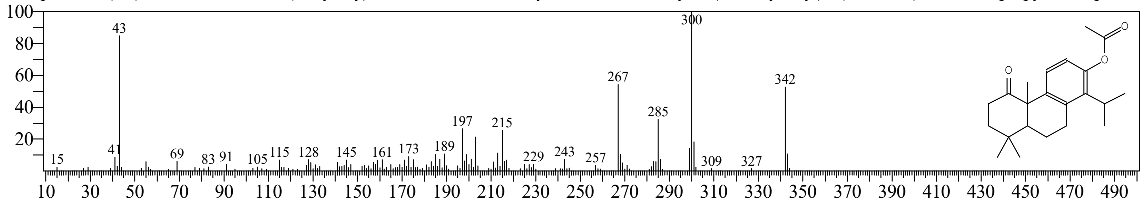

<< Target >>
Line#:35   R.Time:23.590(Scan#:5728) MassPeaks:278
RawMode:Averaged 23.587-23.593(5727-5729) BasePeak:57.10(166800)
BG Mode:Calc. from Peak Group 1 - Event 1 Q3 Scan

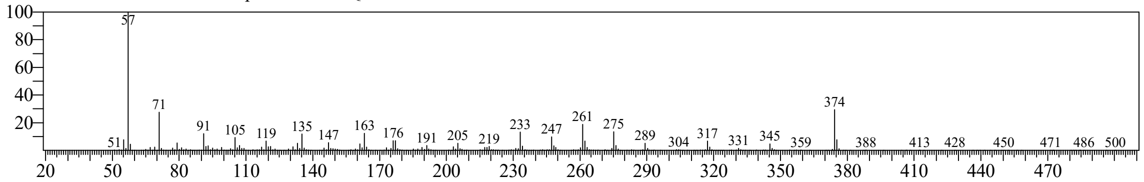

Hit#:1 Entry:176347 Library:NIST17-1.lib
SI:63 Formula:C22H36O2 CAS:0-00-0 MolWeight:332 RetIndex:2346
CompName:2H-1-benzopyran-6-ol, 3,4-dihydro-2,2-dimethyl-4-(1-methylethyl)-7-(1,1,3,3-tetramethylbutyl)- $$ 2,2-dimethyl-4-(propan-2-yl)-7-(2,4,4-trime

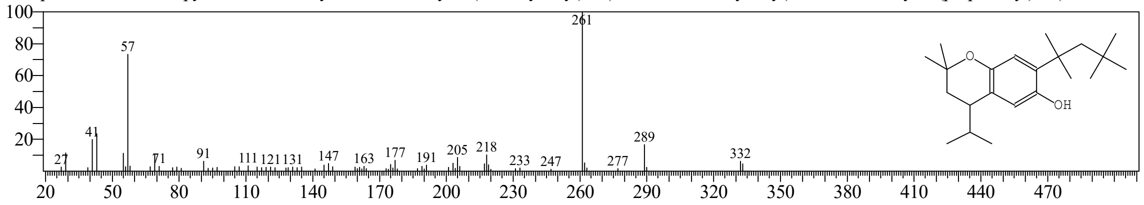

<< Target >>
Line#:36 R.Time:23.797(Scan#:5790) MassPeaks:324
RawMode:Averaged 23.793-23.800(5789-5791) BasePeak:299.20(107098)
BG Mode:Calc. from Peak Group 1 - Event 1 Q3 Scan

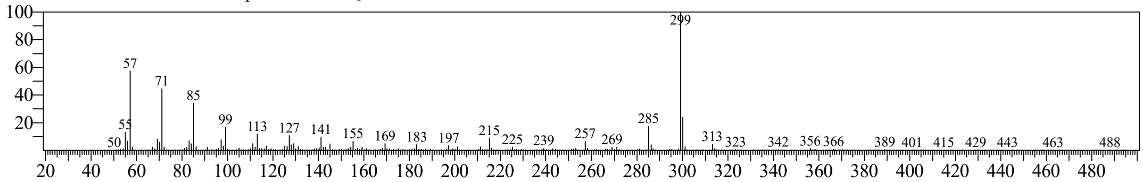

Hit#:1 Entry:194678 Library:NIST17-1.lib
SI:69 Formula:C25H52 CAS:629-99-2 MolWeight:352 RetIndex:2506
CompName:Pentacosane $$ n-Pentacosane $$

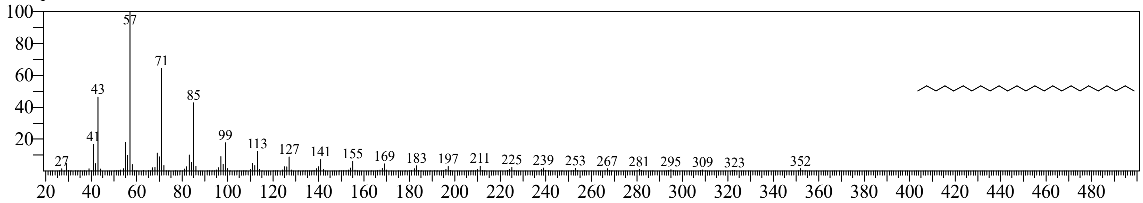

<< Target >>
Line#:37   R.Time:24.373(Scan#:5963) MassPeaks:349
RawMode:Averaged 24.370-24.377(5962-5964) BasePeak:315.20(41601)
BG Mode:Calc. from Peak Group 1 - Event 1 Q3 Scan

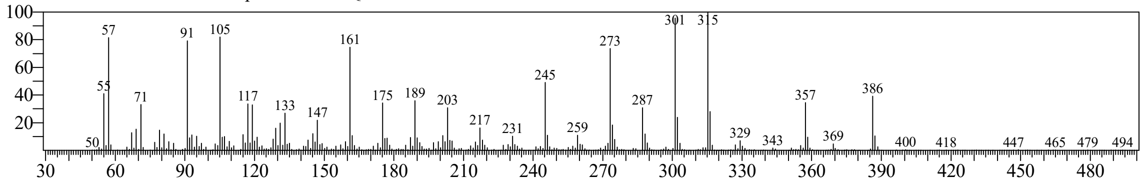

Hit#:1 Entry:37237 Library:NIST17s.lib
SI:60 Formula:C25H38O3 CAS:112924-45-5 MolWeight:386 RetIndex:3031
CompName:(6aS,10aS)-9-(Hydroxymethyl)-6,6-dimethyl-3-(2-methyloctan-2-yl)-6a,7,10,10a-tetrahydrobenzo[c]chromen-1-ol $$ HU-211 $$

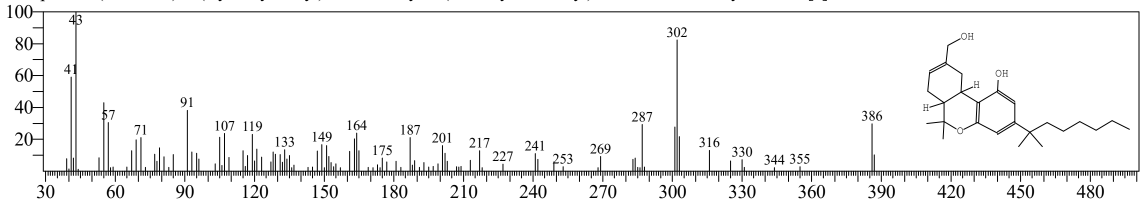

<< Target >>
Line#:38 R.Time:24.767(Scan#:6081) MassPeaks:312
RawMode:Averaged 24.763-24.770(6080-6082) BasePeak:299.20(385593)
BG Mode:Calc. from Peak Group 1 - Event 1 Q3 Scan

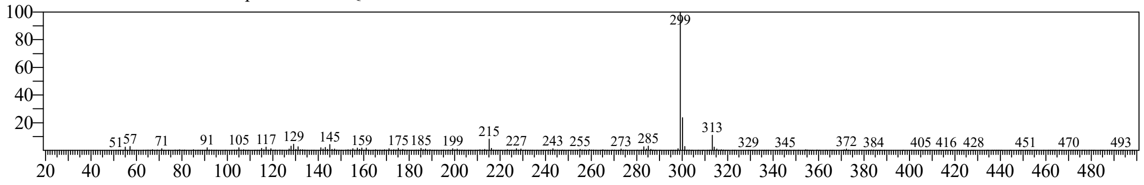

Hit#:1 Entry:185866 Library:NIST17-1.lib
SI:74 Formula:C20H42O2Si CAS:0-00-0 MolWeight:342 RetIndex:1997
CompName:Silane, methylvinyl(hept-4-yloxy)decyloxy-

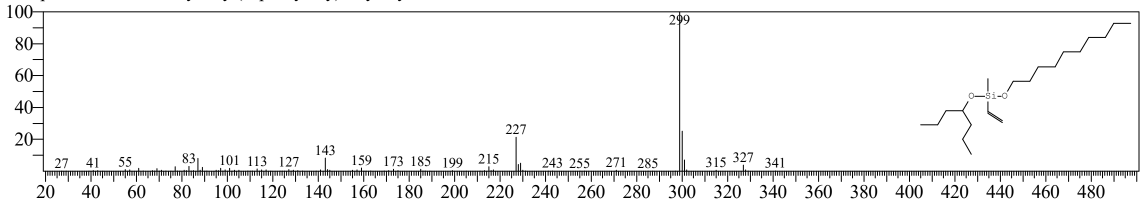

<< Target >>
Line#:39   R.Time:26.050(Scan#:6466) MassPeaks:353
RawMode:Averaged 26.047-26.053(6465-6467) BasePeak:215.15(36558)
BG Mode:Calc. from Peak Group 1 - Event 1 Q3 Scan

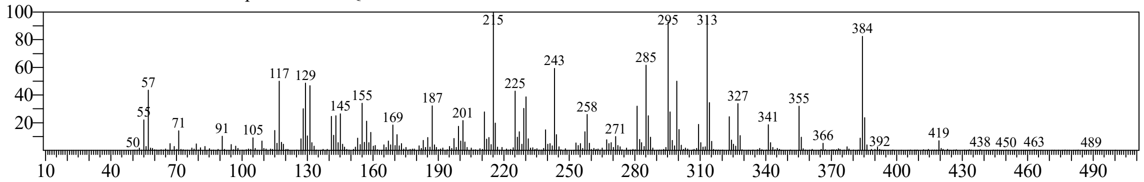

Hit#:1 Entry:172386 Library:NIST17-1.lib
SI:50 Formula:C21H28O3 CAS:18326-18-6 MolWeight:328 RetIndex:2536
CompName:Podocarpa-8,11,13-triene-1,3-dione, 13-isopropyl-12-methoxy- $$ 5.Xi.-Podocarpa-8,11,13-triene-1,3-dione, 13-isopropyl-12-methoxy- $$ 12-I

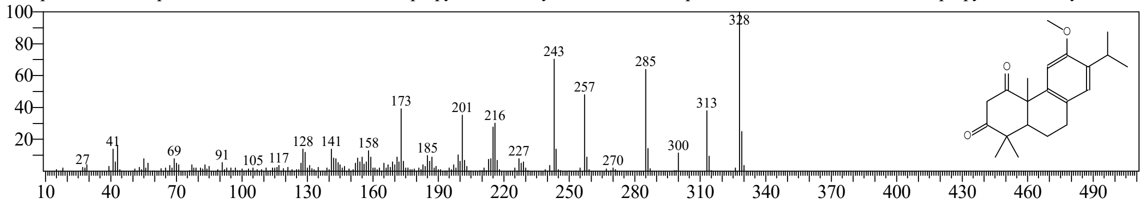

<< Target >>
Line#:40   R.Time:26.830(Scan#:6700) MassPeaks:333
RawMode:Averaged 26.827-26.833(6699-6701) BasePeak:57.10(145350)
BG Mode:Calc. from Peak Group 1 - Event 1 Q3 Scan

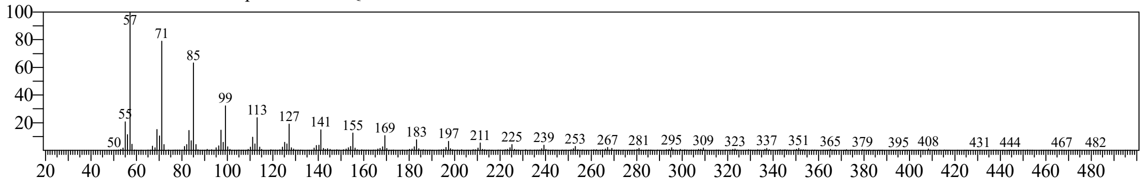

Hit#:1 Entry:38391 Library:NIST17s.lib
SI:95 Formula:C32H66 CAS:544-85-4 MolWeight:450 RetIndex:3202
CompName:Dotriacontane $$ n-Dotriacontane $$ Bicetyl $$

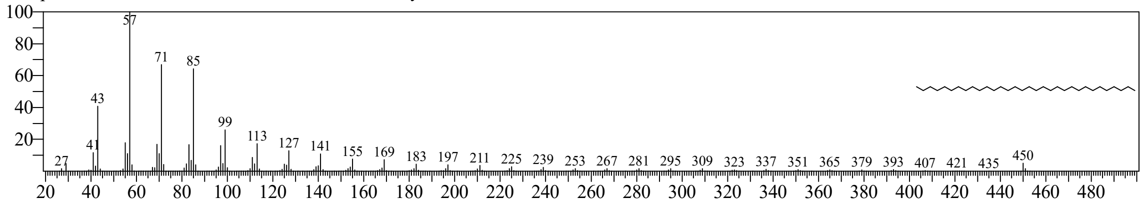

<< Target >>
Line#:41 R.Time:28.963(Scan#:7340) MassPeaks:343
RawMode:Averaged 28.960-28.967(7339-7341) BasePeak:396.30(96825)
BG Mode:Calc. from Peak Group 1 - Event 1 Q3 Scan

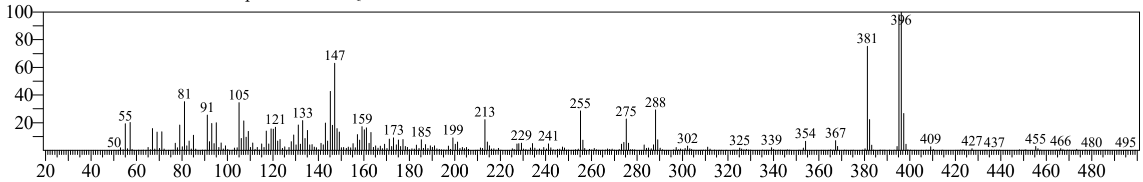

Hit#:1 Entry:37555 Library:NIST17s.lib
SI:83 Formula:C29H48 CAS:4970-37-0 MolWeight:396 RetIndex:2525
CompName:Stigmasta-3,5-diene $$ (8S,9S,10R,13R,14S,17R)-17-((2R)-5-Ethyl-6-methylheptan-2-yl)-10,13-dimethyl-2,7,8,9,10,11,12,13,14,15,16,17-doc

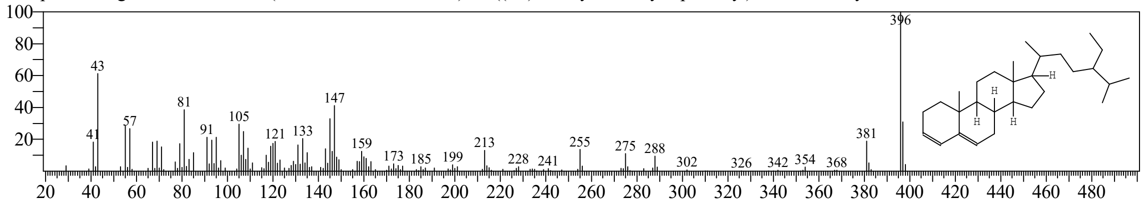

<< Target >>
Line#:42   R.Time:30.177(Scan#:7704) MassPeaks:400
RawMode:Averaged 30.173-30.180(7703-7705) BasePeak:315.25(58908)
BG Mode:Calc. from Peak Group 1 - Event 1 Q3 Scan

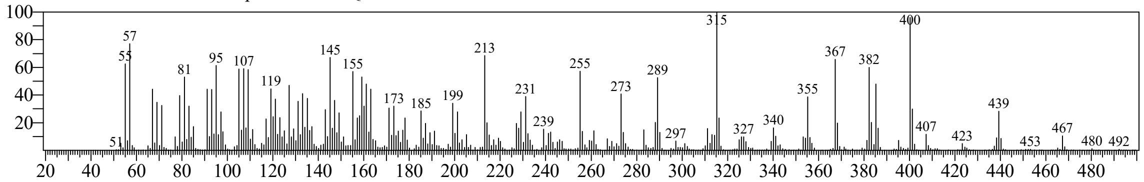

Hit#:1 Entry:37658 Library:NIST17s.lib
SI:81 Formula:C28H48O CAS:474-62-4 MolWeight:400 RetIndex:2632
CompName:Campesterol $$ Ergost-5-en-3-ol, (3.beta.,24R)- $$ Ergost-5-en-3.beta.-ol, (24R)- $$ (24R)-5-Ergosten-3.beta.-ol $$ Campesterin $$ 24.alpha.-

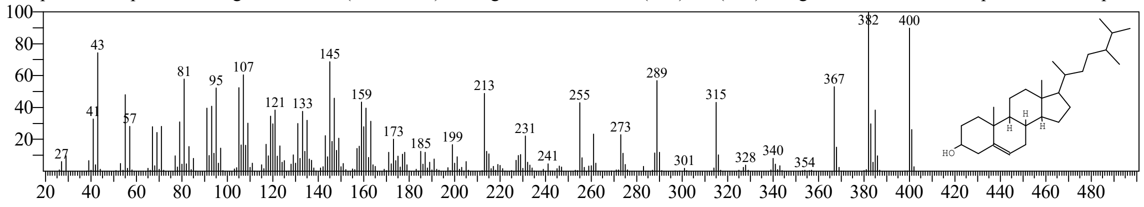

<< Target >>
Line#:43 R.Time:30.460(Scan#:7789) MassPeaks:362
RawMode:Averaged 30.457-30.463(7788-7790) BasePeak:55.05(53194)
BG Mode:Calc. from Peak Group 1 - Event 1 Q3 Scan

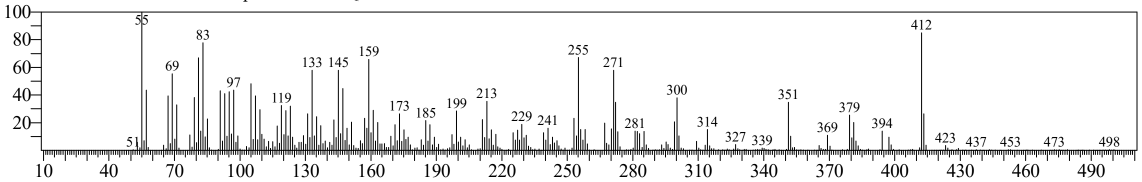

Hit#:1 Entry:37859 Library:NIST17s.lib
SI:82 Formula:C29H48O CAS:83-48-7 MolWeight:412 RetIndex:2739
CompName:Stigmasterol $$ Stigmasta-5,22-dien-3-ol, (3.beta.,22E)- $$ Stigmasta-5,22-dien-3.beta.-ol $$ .beta.-Stigmasterol $$ (24S)-5,22-Stigmastadien-

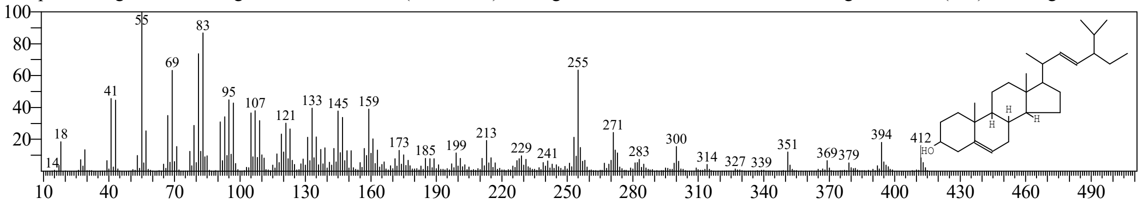

<< Target >>
Line#:44 R.Time:30.773(Scan#:7883) MassPeaks:366
RawMode:Averaged 30.770-30.777(7882-7884) BasePeak:411.30(108788)
BG Mode:Calc. from Peak Group 1 - Event 1 Q3 Scan

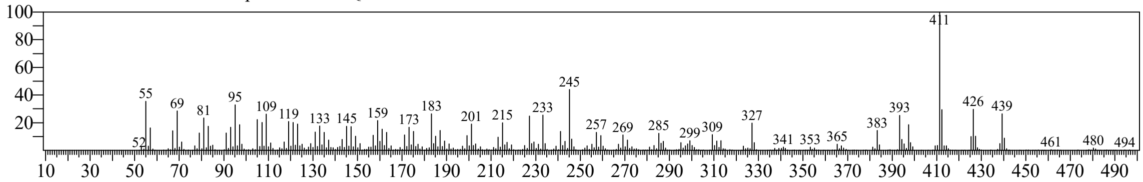

Hit#:1 Entry:239409 Library:NIST17-1.lib
SI:83 Formula:C30H50O CAS:16910-32-0 MolWeight:426 RetIndex:2826
CompName:Obtusifoliol $$ Ergosta-8,24(28)-dien-3-ol, 4,14-dimethyl-, (3.beta.,4.alpha.,5.alpha.)- $$ 5.alpha.-Ergosta-8,24(28)-dien-3.beta.-ol, 4.alpha.,14-

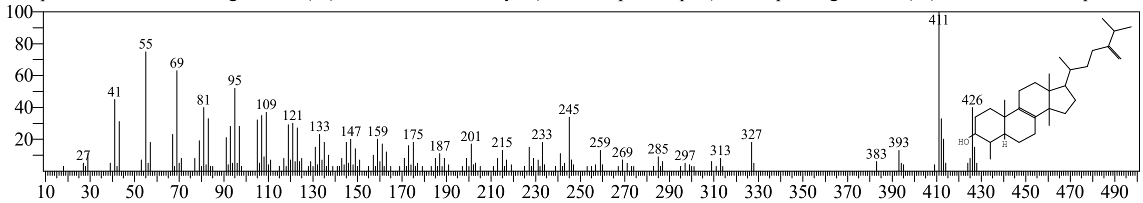

<< Target >>
Line#:45   R.Time:30.887(Scan#:7917) MassPeaks:250
RawMode:Averaged 30.883-30.890(7916-7918) BasePeak:183.15(8717)
BG Mode:Calc. from Peak Group 1 - Event 1 Q3 Scan

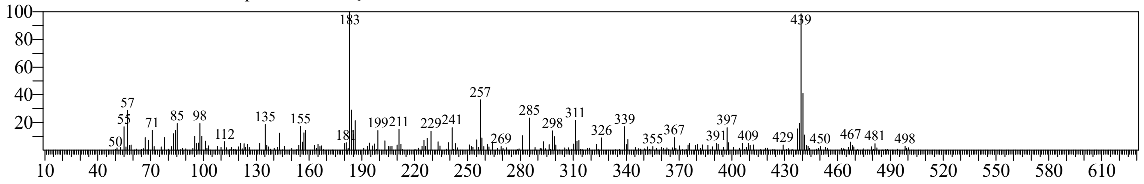

Hit#:1 Entry:7830 Library:NIST17-2.lib
SI:65 Formula:C39H74O6 CAS:538-24-9 MolWeight:638 RetIndex:4336
CompName:Dodecanoic acid, 1,2,3-propanetriyl ester $$ Laurin, tri- $$ Glycerol trilaurate $$ Glyceryl tridodecanoate $$ Glyceryl trilaurate $$ Lauric acid t

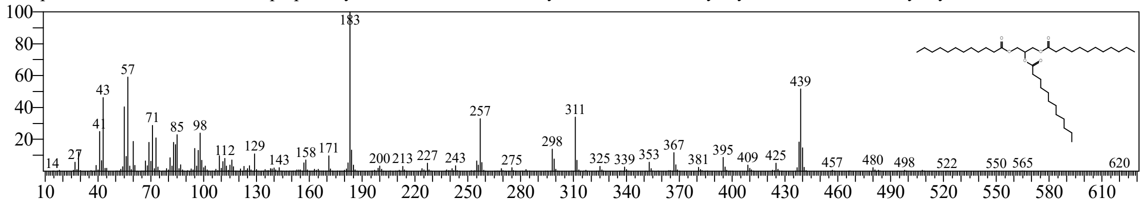

<< Target >>
Line#:46 R.Time:30.977(Scan#:7944) MassPeaks:408
RawMode:Averaged 30.973-30.980(7943-7945) BasePeak:414.35(672254)
BG Mode:Calc. from Peak Group 1 - Event 1 Q3 Scan

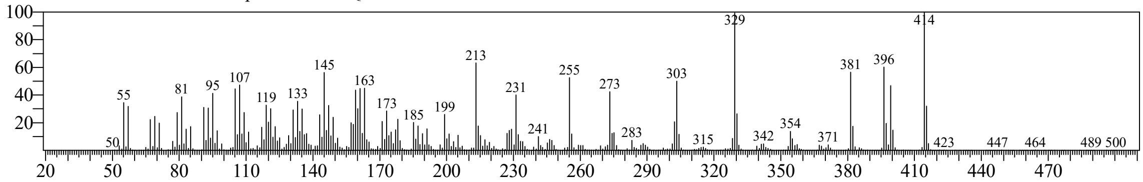

Hit#:1 Entry:37913 Library:NIST17s.lib
SI:86 Formula:C29H50O CAS:83-47-6 MolWeight:414 RetIndex:2731
CompName:.gamma.-Sitosterol $$ Stigmast-5-en-3-ol, (3.beta.,24S)- $$ Stigmast-5-en-3.beta.-ol, (24S)- $$ Clionasterol $$ Fucosterol, .beta.-dihydro- $$ 2

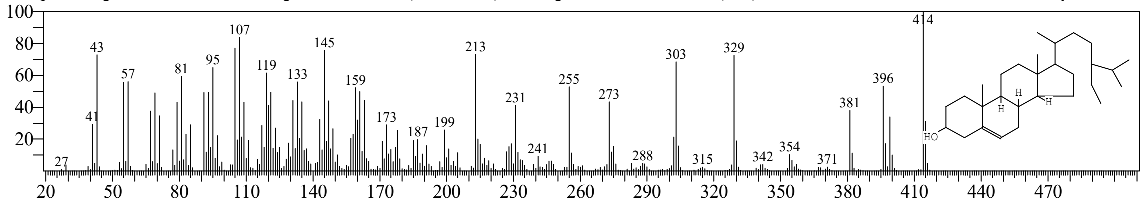

<< Target >>
Line#:47   R.Time:31.463(Scan#:8090) MassPeaks:340
RawMode:Averaged 31.460-31.467(8089-8091) BasePeak:414.35(149733)
BG Mode:Calc. from Peak Group 1 - Event 1 Q3 Scan

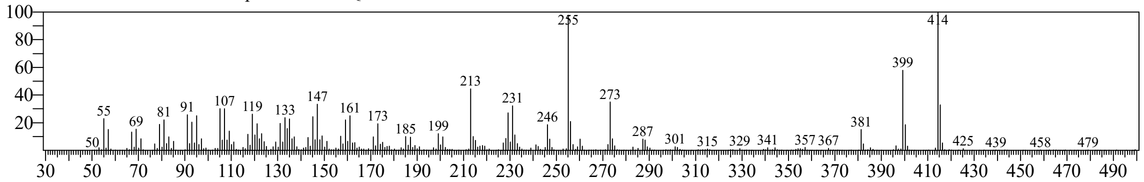

Hit#:1 Entry:234573 Library:NIST17-1.lib
SI:85 Formula:C29H50O CAS:18525-35-4 MolWeight:414 RetIndex:2731
CompName:Stigmast-7-en-3-ol, (3.beta.,5.alpha.,24S)- $$ 5.alpha.-Stigmast-7-en-3.beta.-ol, (24S)- $$ .delta.7-Chondrillastenol $$ Chondrillast-7-enol $$ 2[illegible]

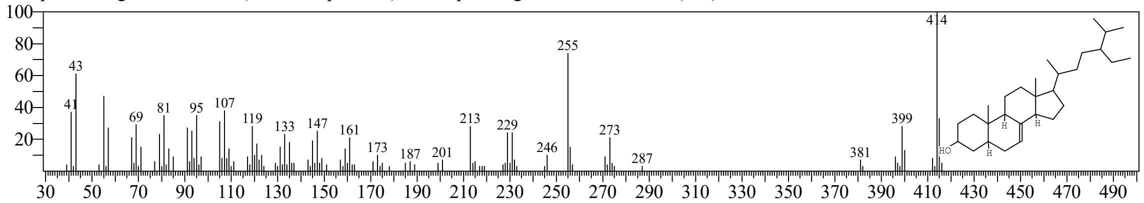

<< Target >>
Line#:48   R.Time:31.640(Scan#:8143) MassPeaks:313
RawMode:Averaged 31.637-31.643(8142-8144) BasePeak:271.20(34598)
BG Mode:Calc. from Peak Group 1 - Event 1 Q3 Scan

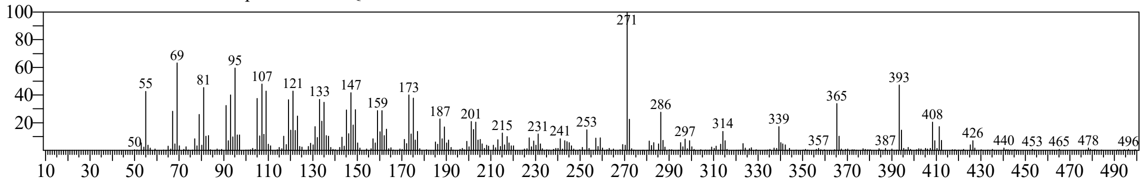

Hit#:1 Entry:38104 Library:NIST17s.lib
SI:84 Formula:C30H50O CAS:469-38-5 MolWeight:426 RetIndex:2816
CompName:9,19-Cyclolanost-24-en-3-ol, (3.beta.)- $$ 9,19-Cyclo-9.beta.-lanost-24-en-3.beta.-ol $$ Cycloartenol $$ Handianol $$ 1-(1,5-Dimethyl-4-hexer

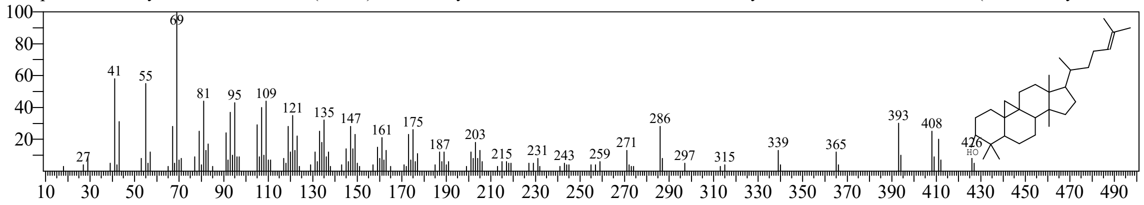

<< Target >>
Line#:49   R.Time:31.780(Scan#:8185) MassPeaks:307
RawMode:Averaged 31.777-31.783(8184-8186) BasePeak:218.20(46209)
BG Mode:Calc. from Peak Group 1 - Event 1 Q3 Scan

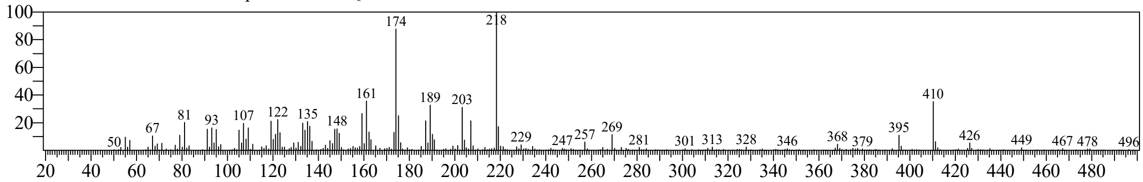

Hit#:1 Entry:224429 Library:NIST17-1.lib
SI:76 Formula:C29H46 CAS:201358-25-0 MolWeight:394 RetIndex:2622
CompName:24-Norursa-3,12-diene $$ (1S,2R,4aR,6aS,6bR,12aS,12bR,14bR)-1,2,4a,6a,6b,9,12a-Heptamethyl-1,2,3,4,4a,5,6,6a,6b,7,8,8a,11,12,12a,12b,1

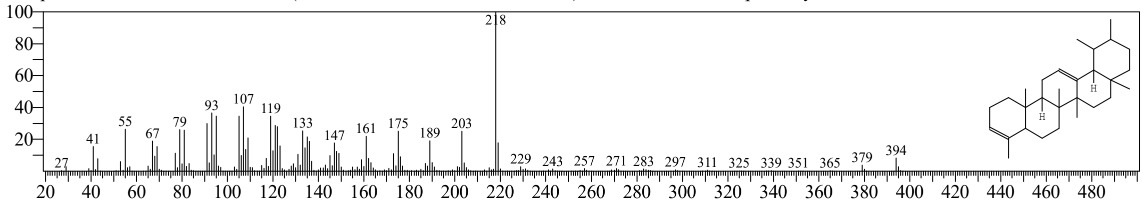

<< Target >>
Line#:50   R.Time:32.160(Scan#:8299) MassPeaks:395
RawMode:Averaged 32.157-32.163(8298-8300) BasePeak:285.20(332242)
BG Mode:Calc. from Peak Group 1 - Event 1 Q3 Scan

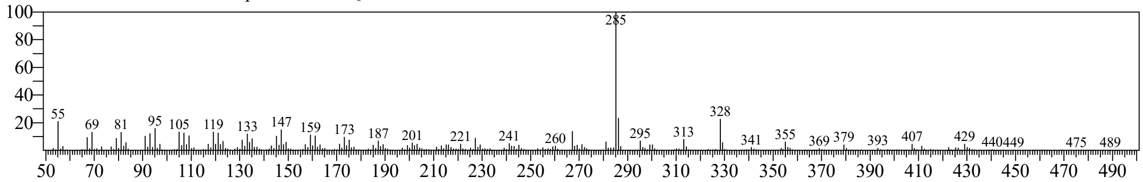

Hit#:1 Entry:172372 Library:NIST17-1.lib
SI:70 Formula:C21H28O3 CAS:23925-68-0 MolWeight:328 RetIndex:2474
CompName:17.beta.-Methyl-18-nor-17-isopregna-4,13-dien-16.beta.-ol-3,20-dione $$ 17-Acetyl-16-hydroxy-10,17-dimethylgona-4,13-dien-3-one # $$

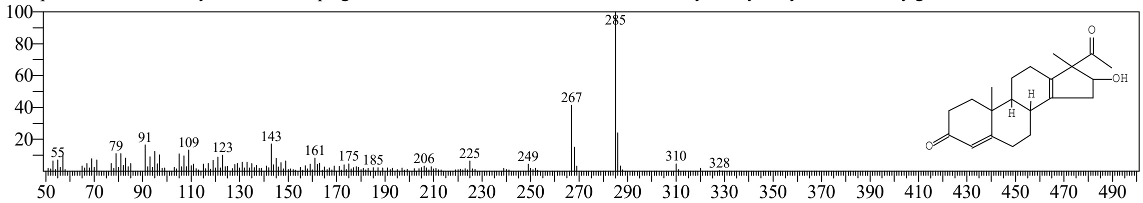

Supplement: Supplementary file 1 [file ijms-26-09156-s001.zip › MS OSO 100min.pdf]
