# Supplementary material for: Ozonized Sunflower Oil: Standardization and Mechanisms of the Antimicrobial Effect
Source: Int J Mol Sci. 2025 Sep 19;26(18):9156. doi: 10.3390/ijms26189156 (PMC12470628; doi:10.3390/ijms26189156)

Relatório de análise - GC-MS/MS

Sample Information

Analyzed by	: Jean Arias
Analyzed	: 12/10/2023 13:18:01
Sample Type	: Unknown
Level #	: 1
Sample Name	: 11102023_OVgirassol_240min_MI
Sample ID	:
Rack	: Rack 2
IS Amount	: [1]=1
Sample Amount	: 1
Dilution Factor	: 1
Vial #	: 14
Injection Volume	: 1.00
Data File	: C:\GCMSsolution\Data\Óleos - ácidos graxos\11102023_amostras_óleos\11102023_OVgirassol_240min_MI.qgd
Org Data File	: C:\GCMSsolution\Data\Óleos - ácidos graxos\11102023_amostras_óleos\11102023_OVgirassol_240min_MI.qgd
Method File	: C:\GCMSsolution\Data\Óleos - ácidos graxos\Matéria insaponificável - scan.qgm
Org Method File	: C:\GCMSsolution\Data\Óleos - ácidos graxos\Matéria insaponificável - scan.qgm
Report File	:
Tuning File	: C:\GCMSsolution\System\Tune1\10102023_tuning.qgt
Modified by	: Jean Arias
Modified	: 18/10/2023 14:23:11

Method

[Comment]

===== Analytical Line 1 =====

[GC-2010]

Column Oven Temp.	:80.0 °C
Injection Temp.	:250.00 °C
Injection Mode	:Split
Flow Control Mode	:Linear Velocity
Pressure	:88.5 kPa
Total Flow	:17.3 mL/min
Column Flow	:1.30 mL/min
Linear Velocity	:42.0 cm/sec
Purge Flow	:3.0 mL/min
Split Ratio	:10.0
High Pressure Injection	:ON
High Press. Inj. Pressure	:300.0 kPa
High Press. Inj. Time	:2.30 min
Carrier Gas Saver	:OFF
Splitter Hold	:OFF

Oven Temp. Program

Rate	Temperature(°C)	Hold Time(min)
-	80.0	1.00
10.00	180.0	0.00
7.00	330.0	0.00

< Ready Check Heat Unit >
Column Oven : Yes
SPL1 : Yes
MS : Yes
< Ready Check Detector(FTD/BID) >
< Ready Check Baseline Drift >
< Ready Check Injection Flow >
SPL1 Carrier : Yes
SPL1 Purge : Yes
< Ready Check APC Flow >
< Ready Check Detector APC Flow >
External Wait :No
Equilibrium Time :3.0 min

[GC Program]

[GCMS-TQ8050]
IonSourceTemp :230.00 °C

Interface Temp. :280.00 °C
Solvent Cut Time :4.00 min
Detector Gain Mode :Relative to the Tuning Result
Detector Gain :1.03 kV +0.00 kV
Threshold :0
Acquire Data without Using CID Gas(Q3Scan) :ON

[MS Table]
--Group 1 - Event 1--
Compound Name :
Start Time :4.50min
End Time :32.40min
Acq. Mode :Q3 Scan
Event Time :0.200sec
Scan Speed :2500
Start m/z :50.00
End m/z :500.00
Q1 Resolution : -
Q3 Resolution : -

Sample Inlet Unit :GC

[MS Program]
Use MS Program :OFF

Peak Report TIC

Peak#	R.Time	Area	Area%	Name	IR
1	8.375	3508123	0.62	Cyclooctyl alcohol	
2	8.873	52772698	9.35	2-Octenal, 2-butyl-	
3	10.126	3056442	0.54	2-Nonenal, 2-pentyl-	
4	10.182	2872746	0.51	2-Octenal, 2-butyl-	
5	11.388	2478143	0.44	2-Nonenal, 2-pentyl-	
6	12.223	5676880	1.01	2-Propylcyclohexanol	
7	12.286	4914933	0.87	2-Propylcyclohexanol	
8	12.600	73023258	12.94	2-Nonenal, 2-pentyl-	
9	12.720	70277131	12.45	2-Octenal, 2-butyl-	
10	13.015	2450741	0.43	Tetrapentacontane, 1,54-dibromo-	
11	13.421	2570074	0.46	Dill ether	
12	13.815	3201747	0.57	2-Nonenal, 2-pentyl-	
13	13.878	4164529	0.74	2-Nonenal, 2-pentyl-	
14	15.087	4231878	0.75	2-Nonenal, 2-pentyl-	
15	16.040	6269630	1.11	Pentadecanal-	
16	16.274	6551594	1.16	n-Hexadecanoic acid	
17	16.363	80473747	14.26	2-Nonenal, 2-pentyl-	
18	16.586	2407420	0.43	2-Dodecen-1-yl(-)succinic anhydride	
19	16.636	3506687	0.62	10-Undecen-1-ol, 2-methoxy-	
20	16.904	3254159	0.58	7,9-Di-tert-butyl-1-oxaspiro(4,5)deca-6,9-diene-2,8-dion	
21	17.823	2818101	0.50	2,3-naphthalenedicarboxaldehyde, 5,6,7,8-tetrahydro-5,	
22	17.951	2275735	0.40	2-Dodecen-1-yl(-)succinic anhydride	
23	18.020	3129605	0.55	n-Nonenylsuccinic anhydride	
24	18.065	5305623	0.94	Ethanone, 1-(5,6,7,8-tetrahydro-2,8,8-trimethyl-4H-cyclo	
25	18.240	3326557	0.59	Phytol	
26	18.473	14806291	2.62	9,12-Octadecadienoic acid (Z,Z)-	
27	18.542	24659798	4.37	Oleic Acid	
28	18.786	5359327	0.95	Octadecanoic acid	
29	20.368	4690831	0.83	2H-1-benzopyran-6-ol, 3,4-dihydro-2,2-dimethyl-4-(1-me	
30	20.452	3222559	0.57	Silane, trichlorodecyl-	
31	21.172	2613540	0.46	14,15,16-Trinor-8.xi.-labdan-6.beta.-ol, 8,13-epoxy-	
32	21.298	4030013	0.71	Tetracyclo[6.1.0.0(2,4).0(5,7)]nonane, 3,3,6,6,9,9-hexae	
33	21.426	5175275	0.92	1,4-benzenediamine, 2-methyl-N4,N4-dioctyl-	
34	21.463	2714089	0.48	Cyclohexane, 1,3-didecyl-	
35	21.517	3992474	0.71	2H-1-benzopyran-6-ol, 3,4-dihydro-2,2-dimethyl-4-(1-me	
36	21.967	3451176	0.61	Benzoic acid, 4-(4-pentylcyclohexyl)-, 4'-cyano[1,1'-biph	
37	23.120	3180072	0.56	4(1H)-Phenanthrenone, 7-(acetyloxy)-2,3,4a,9,10,10a-h	
38	23.595	4138420	0.73	2H-1-benzopyran-6-ol, 3,4-dihydro-2,2-dimethyl-4-(1-me	
39	23.802	2587997	0.46	Succinic acid, 2-(2-chlorophenoxy)ethyl undecyl ester	
40	24.376	2664250	0.47	(6aS,10aS)-9-(Hydroxymethyl)-6,6-dimethyl-3-(2-methyl	
41	24.539	3138547	0.56	2H-1-benzopyran-6-ol, 3,4-dihydro-2,2-dimethyl-4-(1-me	
42	24.770	3035419	0.54	Silane, methylvinyl(hept-4-yloxy)decyloxy-	
43	26.047	2675351	0.47	2,3-Hexadienoic acid, 2-ethyl-4-phenyl-, ethyl ester	
44	28.964	3147789	0.56	.beta.-Sitosterol, propionate	
45	30.166	7099403	1.26	Campesterol	
46	30.449	6777375	1.20	Stigmasterol	
47	30.767	6221394	1.10	Obtusifoliol	
48	30.975	68660429	12.17	.gamma.-Sitosterol	
49	31.458	13951439	2.47	Stigmast-7-en-3-ol, (3.beta.,5.alpha.,24S)-	
50	32.150	7876276	1.40	Pregnan-17,21-diol-9,11-epoxy-3,20-dione, acetate	
		564387715	100.00		

Chromatogram 11102023_OVgirassol_240min_MI C:\GCMSsolution\Data\Óleos - ácidos graxos\11102023_amostras_óleos\11102023_OVgirassol_240min_MI.qgd

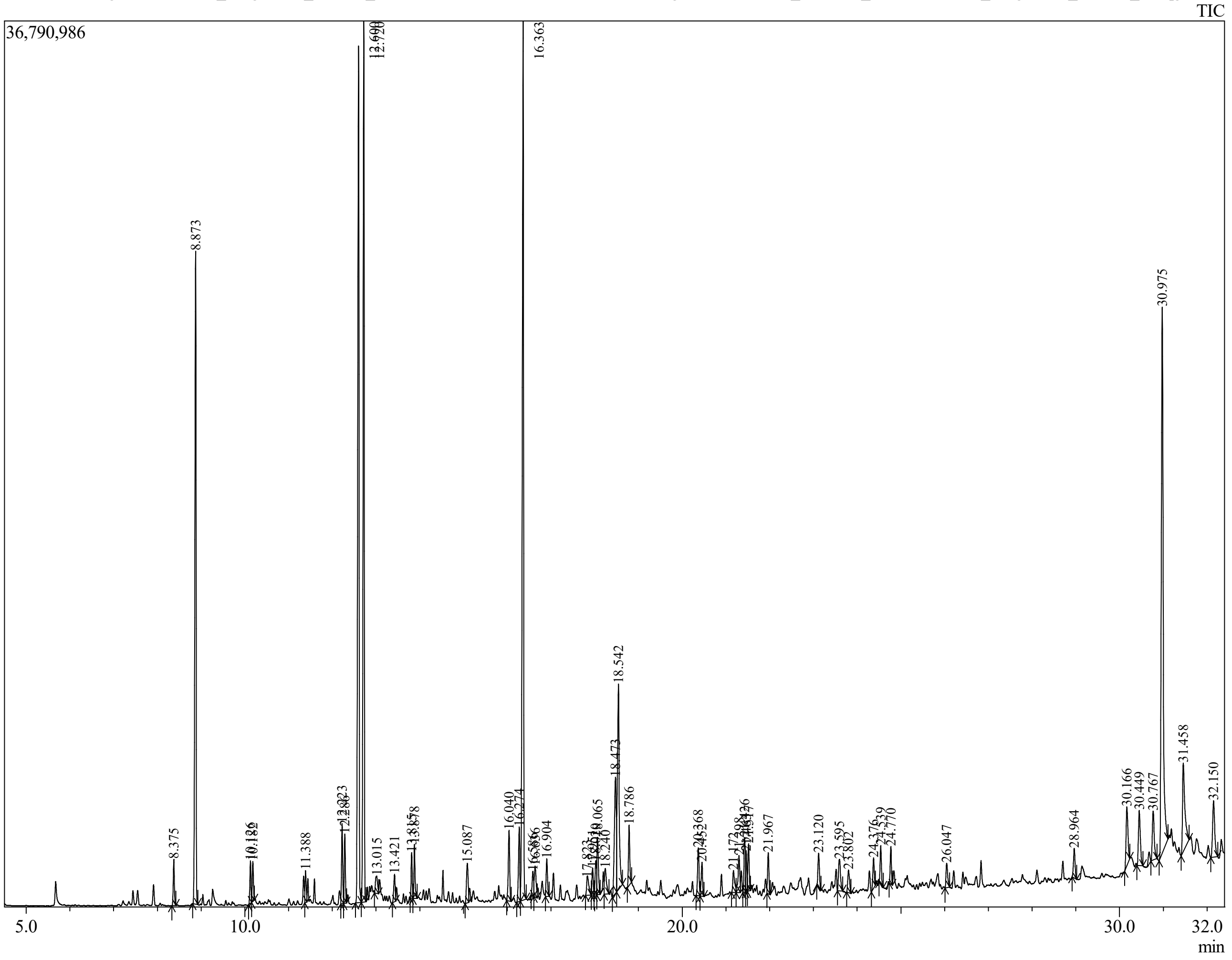

Library

<< Target >>
Line#:1   R.Time:8.377(Scan#:1164) MassPeaks:254
RawMode:Averaged 8.373-8.380(1163-1165) BasePeak:57.05(336421)
BG Mode:Calc. from Peak Group 1 - Event 1 Q3 Scan

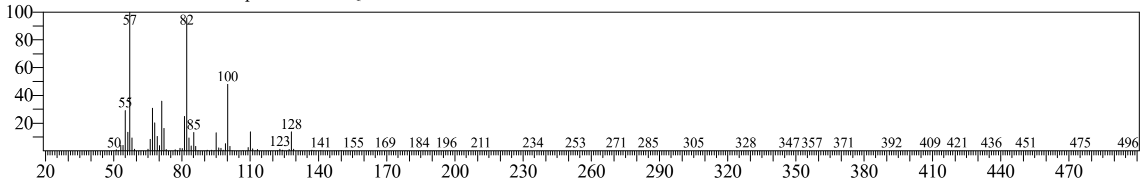

Hit#:1 Entry:6249 Library:NIST17s.lib
SI:86 Formula:C8H16O CAS:696-71-9 MolWeight:128 RetIndex:1147
CompName:Cyclooctyl alcohol $$ Cyclooctanol $$

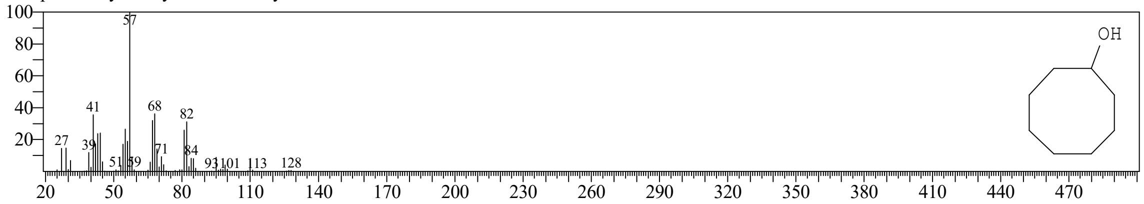

<< Target >>
Line#:2 R.Time:8.873(Scan#:1313) MassPeaks:293
RawMode:Averaged 8.870-8.877(1312-1314) BasePeak:55.05(2254006)
BG Mode:Calc. from Peak Group 1 - Event 1 Q3 Scan

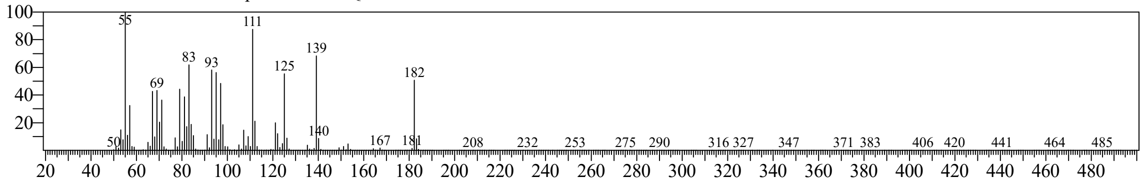

Hit#:1 Entry:18037 Library:NIST17s.lib
SI:95 Formula:C12H22O CAS:13019-16-4 MolWeight:182 RetIndex:1388
CompName:2-Octenal, 2-butyl- $$ 2-Butyl-2-octenal $$ 2-n-Butyloct-2-enal $$

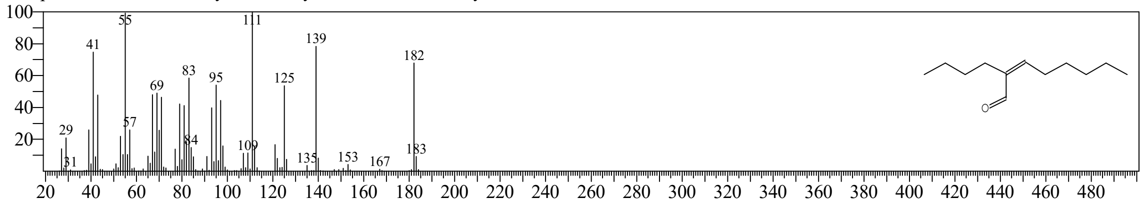

<< Target >>
Line#:3 R.Time:10.127(Scan#:1689) MassPeaks:288
RawMode:Averaged 10.123-10.130(1688-1690) BasePeak:55.05(124912)
BG Mode:Calc. from Peak Group 1 - Event 1 Q3 Scan

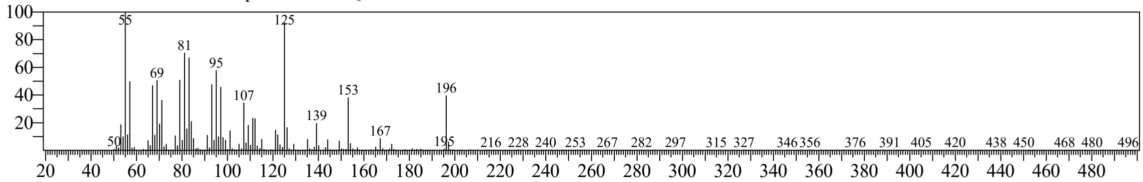

Hit#:1 Entry:58137 Library:NIST17-1.lib
SI:84 Formula:C14H26O CAS:3021-89-4 MolWeight:210 RetIndex:1586
CompName:2-Nonenal, 2-pentyl- $$ 2-Amylnon-2-enal $$ 2-Pentyl-2-nonenal $$ 2-Pentylnon-2-enal $$

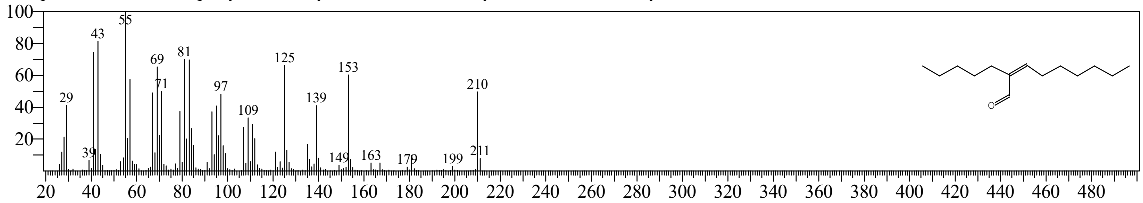

<< Target >>
Line#:4 R.Time:10.183(Scan#:1706) MassPeaks:288
RawMode:Averaged 10.180-10.187(1705-1707) BasePeak:55.05(135233)
BG Mode:Calc. from Peak Group 1 - Event 1 Q3 Scan

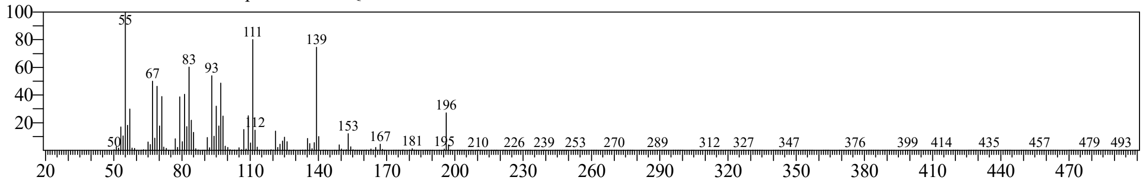

Hit#:1 Entry:18037 Library:NIST17s.lib
SI:88 Formula:C12H22O CAS:13019-16-4 MolWeight:182 RetIndex:1388
CompName:2-Octenal, 2-butyl- $$ 2-Butyl-2-octenal $$ 2-n-Butyloct-2-enal $$

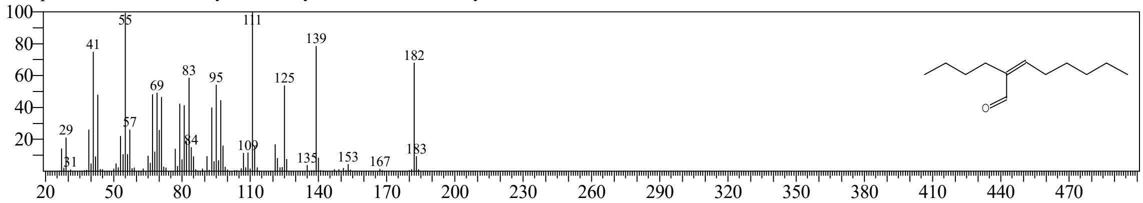

<< Target >>
Line#:5 R.Time:11.387(Scan#:2067) MassPeaks:281
RawMode:Averaged 11.383-11.390(2066-2068) BasePeak:55.05(70915)
BG Mode:Calc. from Peak Group 1 - Event 1 Q3 Scan

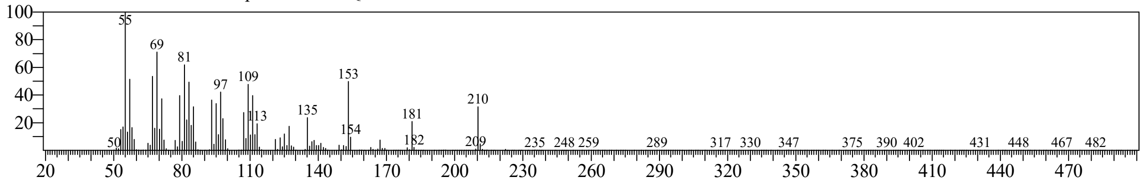

Hit#:1 Entry:58137 Library:NIST17-1.lib
SI:86 Formula:C14H26O CAS:3021-89-4 MolWeight:210 RetIndex:1586
CompName:2-Nonenal, 2-pentyl- $$ 2-Amylnon-2-enal $$ 2-Pentyl-2-nonenal $$ 2-Pentylnon-2-enal $$

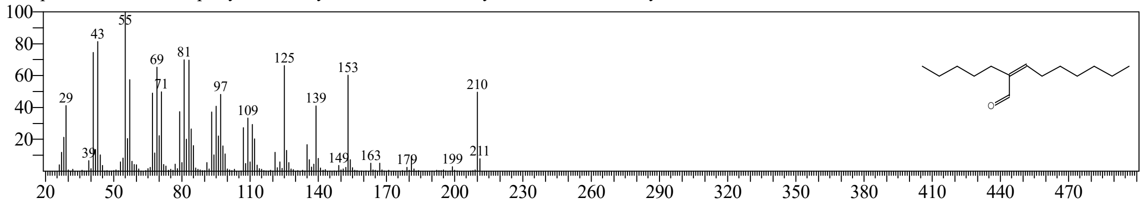

<< Target >>
Line#:6   R.Time:12.223(Scan#:2318) MassPeaks:291
RawMode:Averaged 12.220-12.227(2317-2319) BasePeak:82.05(302483)
BG Mode:Calc. from Peak Group 1 - Event 1 Q3 Scan

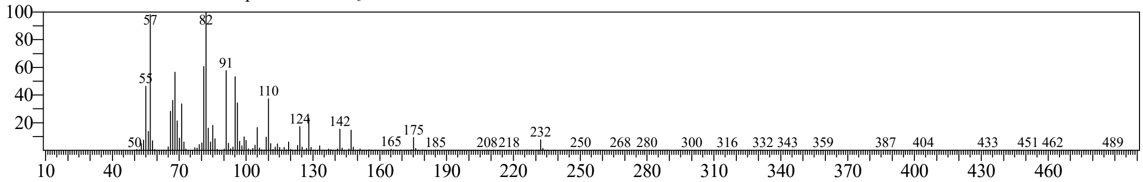

Hit#:1 Entry:13077 Library:NIST17-1.lib
SI:81 Formula:C9H18O CAS:90676-25-8 MolWeight:142 RetIndex:1167
CompName:2-Propylcyclohexanol

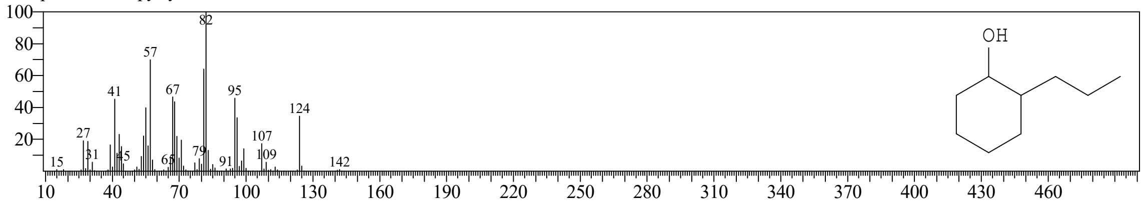

<< Target >>
Line#:7 R.Time:12.287(Scan#:2337) MassPeaks:238
RawMode:Averaged 12.283-12.290(2336-2338) BasePeak:82.05(518028)
BG Mode:Calc. from Peak Group 1 - Event 1 Q3 Scan

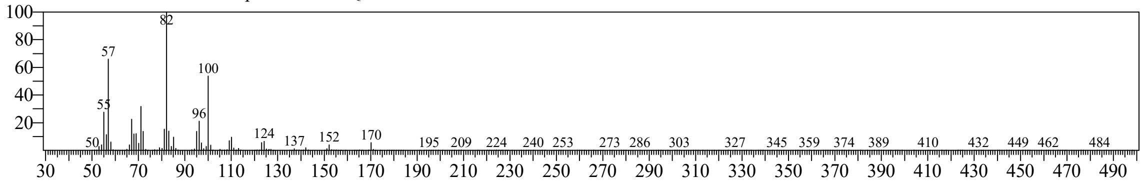

Hit#:1 Entry:9184 Library:NIST17s.lib
SI:82 Formula:C9H18O CAS:90676-25-8 MolWeight:142 RetIndex:1167
CompName:2-Propylcyclohexanol

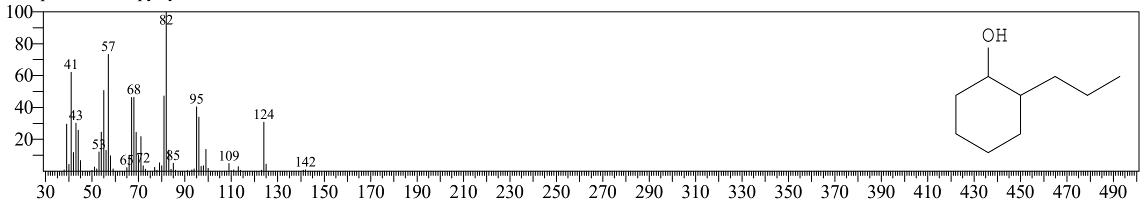

<< Target >>
Line#:8 R.Time:12.600(Scan#:2431) MassPeaks:300
RawMode:Averaged 12.597-12.603(2430-2432) BasePeak:55.05(1935580)
BG Mode:Calc. from Peak Group 1 - Event 1 Q3 Scan

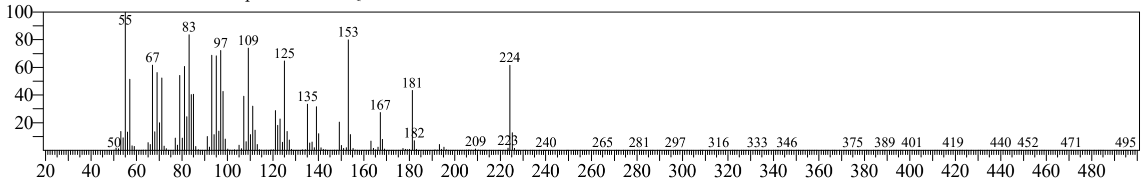

Hit#:1 Entry:58137 Library:NIST17-1.lib
SI:83 Formula:C14H26O CAS:3021-89-4 MolWeight:210 RetIndex:1586
CompName:2-Nonenal, 2-pentyl- $$ 2-Amylnon-2-enal $$ 2-Pentyl-2-nonenal $$ 2-Pentylnon-2-enal $$

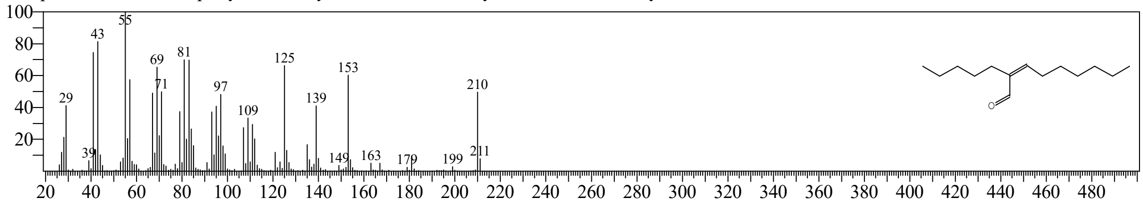

<< Target >>
Line#:9 R.Time:12.720(Scan#:2467) MassPeaks:289
RawMode:Averaged 12.717-12.723(2466-2468) BasePeak:111.05(2666951)
BG Mode:Calc. from Peak Group 1 - Event 1 Q3 Scan

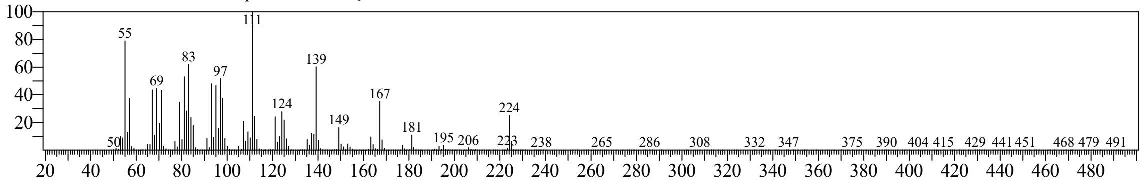

Hit#:1 Entry:18037 Library:NIST17s.lib
SI:82 Formula:C12H22O CAS:13019-16-4 MolWeight:182 RetIndex:1388
CompName:2-Octenal, 2-butyl- $$ 2-Butyl-2-octenal $$ 2-n-Butyloct-2-enal $$

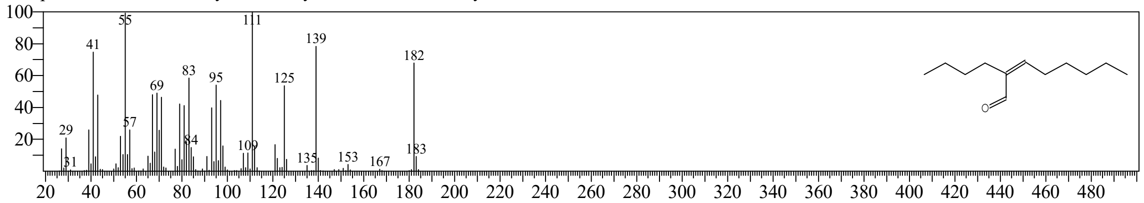

<< Target >>
Line#:10 R.Time:13.013(Scan#:2555) MassPeaks:278
RawMode:Averaged 13.010-13.017(2554-2556) BasePeak:57.05(69532)
BG Mode:Calc. from Peak Group 1 - Event 1 Q3 Scan

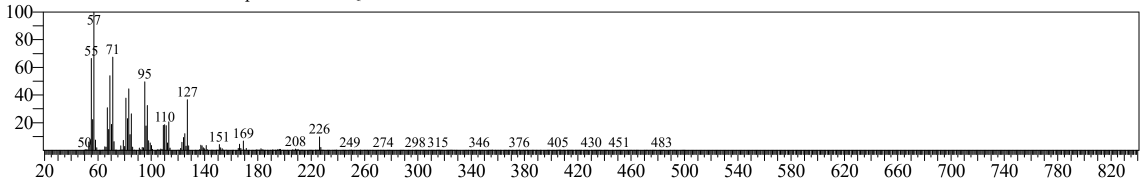

Hit#:1 Entry:9801 Library:NIST17-2.lib
SI:87 Formula:C54H108Br2 CAS:0-00-0 MolWeight:914 RetIndex:5981
CompName:Tetrapentacontane, 1,54-dibromo- $$ 1,54-Dibromotetrapentacontane # $$

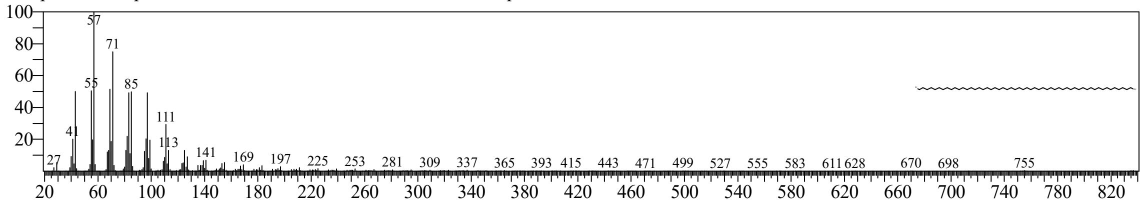

<< Target >>
Line#:11   R.Time:13.420(Scan#:2677) MassPeaks:283
RawMode:Averaged 13.417-13.423(2676-2678) BasePeak:137.10(310168)
BG Mode:Calc. from Peak Group 1 - Event 1 Q3 Scan

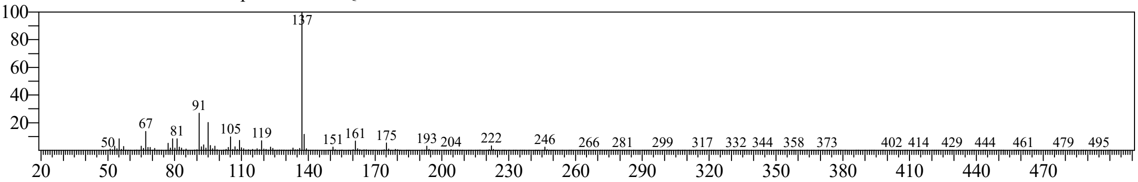

Hit#:1 Entry:17655 Library:NIST17-1.lib
SI:81 Formula:C10H16O CAS:74410-10-9 MolWeight:152 RetIndex:1103
CompName:Dill ether $$ (3S,3aS,7aR)-3,6-Dimethyl-2,3,3a,4,5,7a-hexahydrobenzofuran $$ Benzofuran, 2,3,3a,4,5,7a-hexahydro-3,6-dimethyl-, (3S,3aS,7a

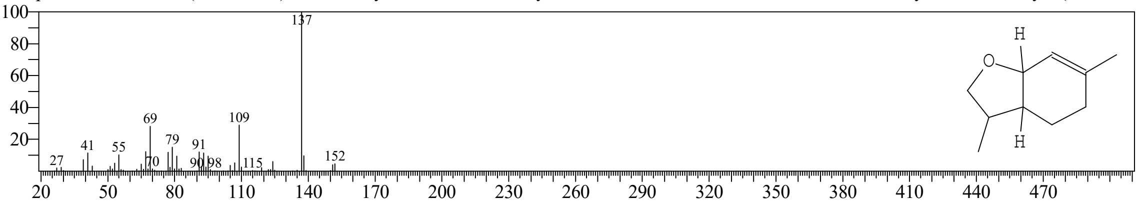

<< Target >>
Line#:12   R.Time:13.813(Scan#:2795) MassPeaks:250
RawMode:Averaged 13.810-13.817(2794-2796) BasePeak:55.05(119025)
BG Mode:Calc. from Peak Group 1 - Event 1 Q3 Scan

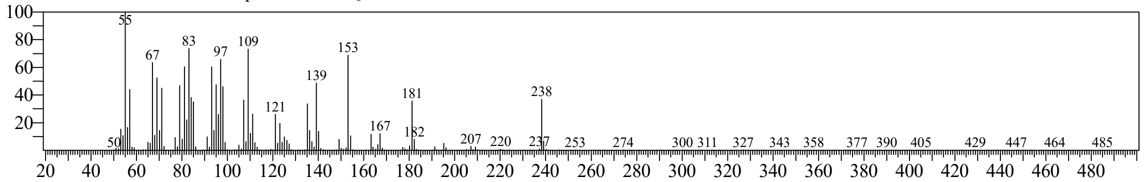

Hit#:1 Entry:58137 Library:NIST17-1.lib
SI:84 Formula:C14H26O CAS:3021-89-4 MolWeight:210 RetIndex:1586
CompName:2-Nonenal, 2-pentyl- $$ 2-Amylnon-2-enal $$ 2-Pentyl-2-nonenal $$ 2-Pentylnon-2-enal $$

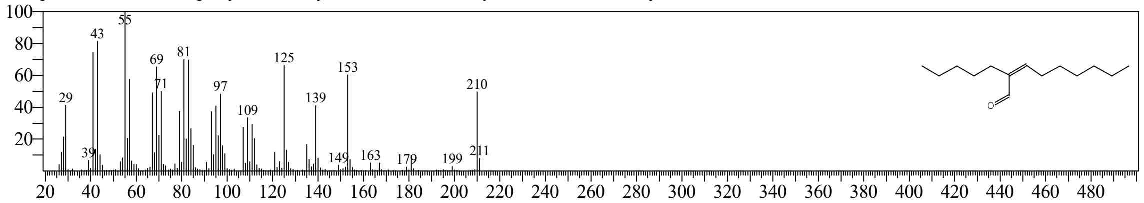

<< Target >>
Line#:13   R.Time:13.877(Scan#:2814) MassPeaks:285
RawMode:Averaged 13.873-13.880(2813-2815) BasePeak:55.05(127869)
BG Mode:Calc. from Peak Group 1 - Event 1 Q3 Scan

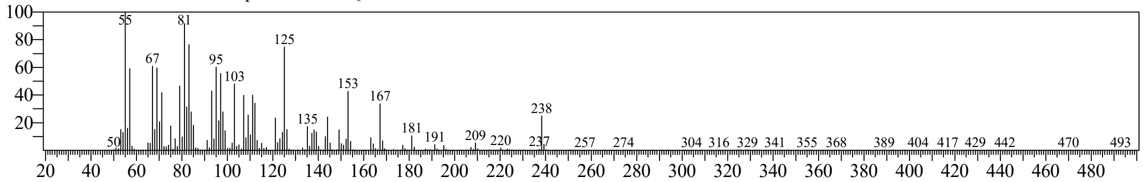

Hit#:1 Entry:58137 Library:NIST17-1.lib
SI:83 Formula:C14H26O CAS:3021-89-4 MolWeight:210 RetIndex:1586
CompName:2-Nonenal, 2-pentyl- $$ 2-Amylnon-2-enal $$ 2-Pentyl-2-nonenal $$ 2-Pentylnon-2-enal $$

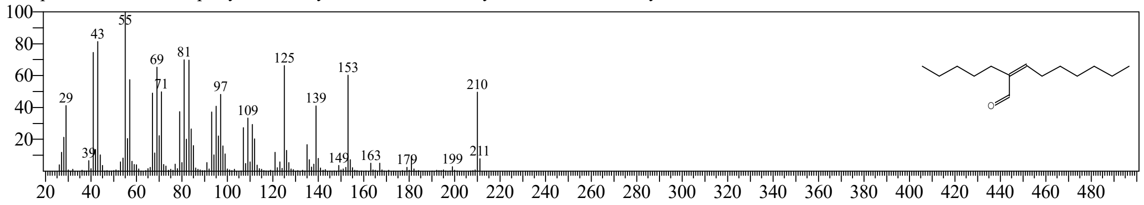

<< Target >>
Line#:14 R.Time:15.087(Scan#:3177) MassPeaks:283
RawMode:Averaged 15.083-15.090(3176-3178) BasePeak:55.05(89640)
BG Mode:Calc. from Peak Group 1 - Event 1 Q3 Scan

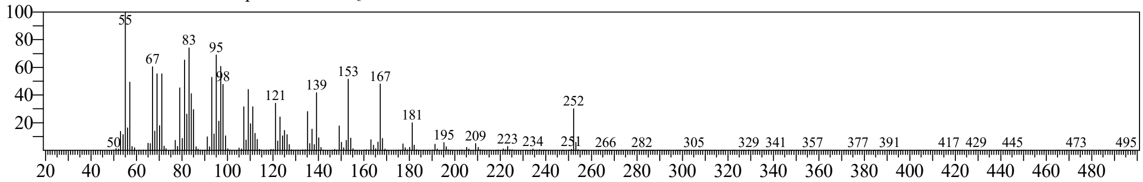

Hit#:1 Entry:58137 Library:NIST17-1.lib
SI:84 Formula:C14H26O CAS:3021-89-4 MolWeight:210 RetIndex:1586
CompName:2-Nonenal, 2-pentyl- $$ 2-Amylnon-2-enal $$ 2-Pentyl-2-nonenal $$ 2-Pentylnon-2-enal $$

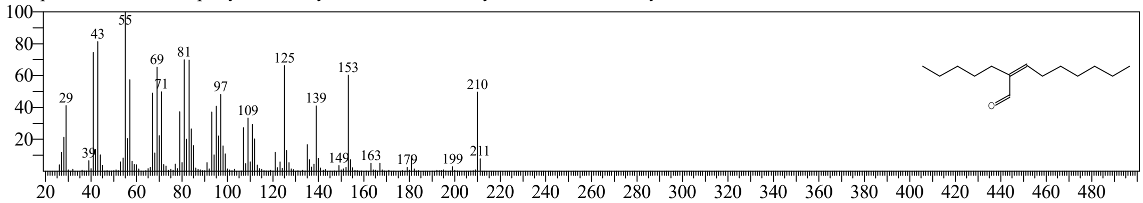

<< Target >>
Line#:15   R.Time:16.040(Scan#:3463) MassPeaks:313
RawMode:Averaged 16.037-16.043(3462-3464) BasePeak:82.05(395800)
BG Mode:Calc. from Peak Group 1 - Event 1 Q3 Scan

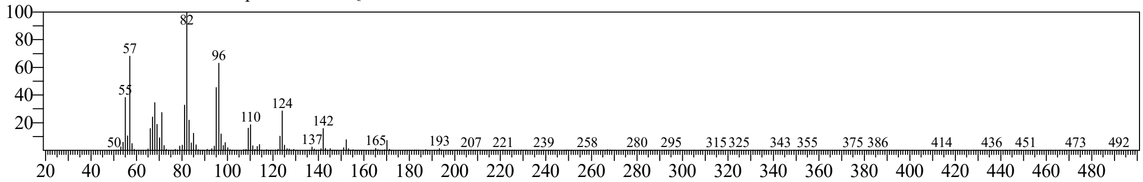

Hit#:1 Entry:25974 Library:NIST17s.lib
SI:87 Formula:C15H30O CAS:2765-11-9 MolWeight:226 RetIndex:1701
CompName:Pentadecanal- $$ 1-Pentadecanal $$ n-Pentadecanal $$

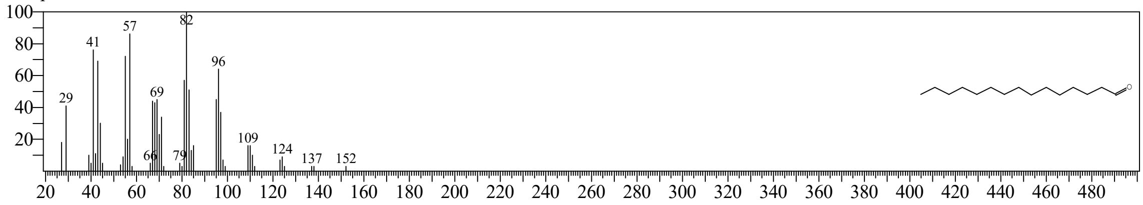

<< Target >>
Line#:16   R.Time:16.273(Scan#:3533) MassPeaks:337
RawMode:Averaged 16.270-16.277(3532-3534) BasePeak:73.05(218560)
BG Mode:Calc. from Peak Group 1 - Event 1 Q3 Scan

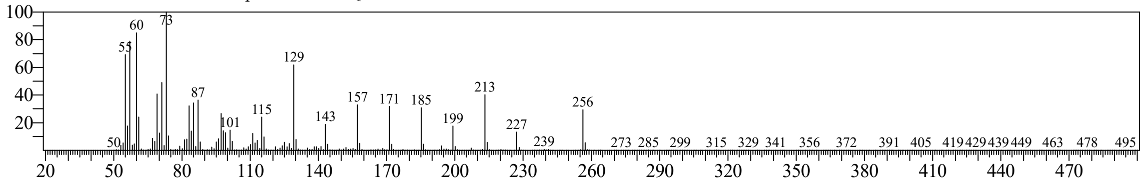

Hit#:1 Entry:29350 Library:NIST17s.lib
SI:93 Formula:C16H32O2 CAS:57-10-3 MolWeight:256 RetIndex:1968
CompName:n-Hexadecanoic acid $$ Hexadecanoic acid $$ n-Hexadecoic acid $$ Palmitic acid $$ Pentadecanecarboxylic acid $$ 1-Pentadecanecarboxylic

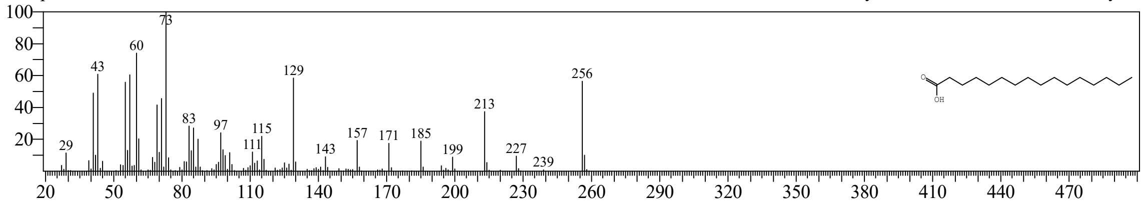

<< Target >>
Line#:17 R.Time:16.363(Scan#:3560) MassPeaks:317
RawMode:Averaged 16.360-16.367(3559-3561) BasePeak:55.05(1621551)
BG Mode:Calc. from Peak Group 1 - Event 1 Q3 Scan

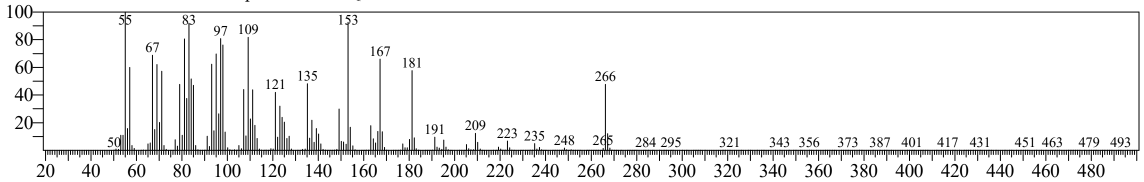

Hit#:1 Entry:58137 Library:NIST17-1.lib
SI:78 Formula:C14H26O CAS:3021-89-4 MolWeight:210 RetIndex:1586
CompName:2-Nonenal, 2-pentyl- $$ 2-Amylnon-2-enal $$ 2-Pentyl-2-nonenal $$ 2-Pentylnon-2-enal $$

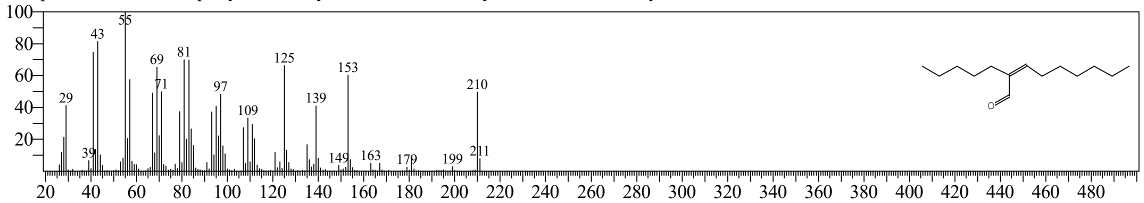

<< Target >>
Line#:18 R.Time:16.587(Scan#:3627) MassPeaks:302
RawMode:Averaged 16.583-16.590(3626-3628) BasePeak:225.15(45738)
BG Mode:Calc. from Peak Group 1 - Event 1 Q3 Scan

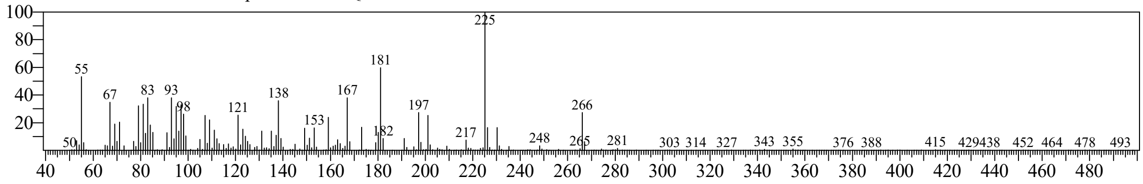

Hit#:1 Entry:30199 Library:NIST17s.lib
SI:65 Formula:C16H26O3 CAS:19780-11-1 MolWeight:266 RetIndex:2159
CompName:2-Dodecen-1-yl(-)succinic anhydride $$ 2,5-Furandione, 3-dodecenyl- $$ n-Dodecenylsuccinic anhydride $$ Dodecenyl succinic anhydride $$ 2

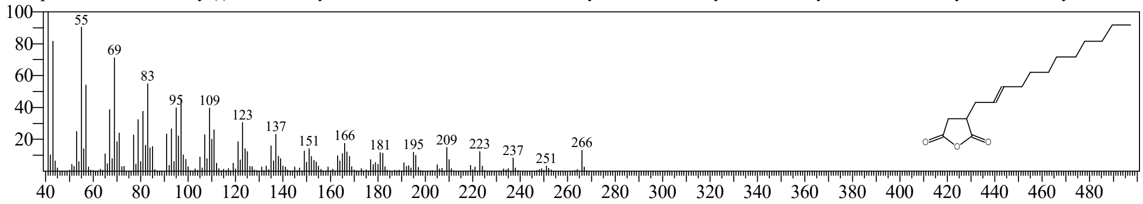

<< Target >>
Line#:19 R.Time:16.637(Scan#:3642) MassPeaks:286
RawMode:Averaged 16.633-16.640(3641-3643) BasePeak:169.10(94246)
BG Mode:Calc. from Peak Group 1 - Event 1 Q3 Scan

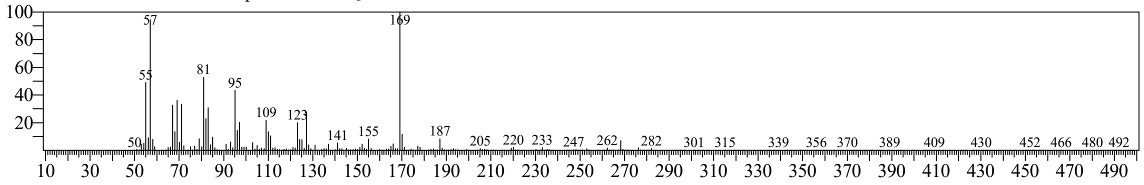

Hit#:1 Entry:49626 Library:NIST17-1.lib
SI:76 Formula:C12H24O2 CAS:54889-62-2 MolWeight:200 RetIndex:1459
CompName:10-Undecen-1-ol, 2-methoxy- $$ 2-Methoxy-10-undecen-1-ol # $$

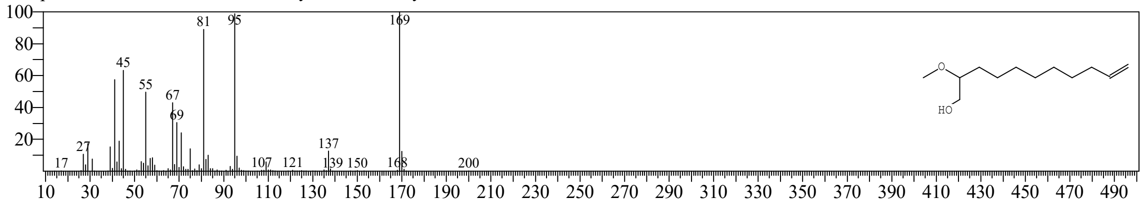

<< Target >>
Line#:20 R.Time:16.903(Scan#:3722) MassPeaks:311
RawMode:Averaged 16.900-16.907(3721-3723) BasePeak:57.05(208504)
BG Mode:Calc. from Peak Group 1 - Event 1 Q3 Scan

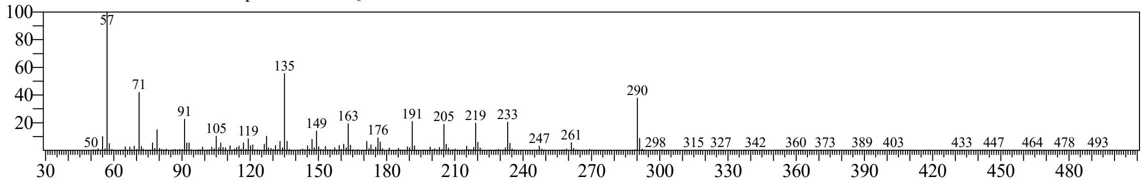

Hit#:1 Entry:31113 Library:NIST17s.lib
SI:61 Formula:C17H24O3 CAS:82304-66-3 MolWeight:276 RetIndex:2081
CompName:7,9-Di-tert-butyl-1-oxaspiro(4,5)deca-6,9-diene-2,8-dione $$ 1-Oxa-spiro[4.5]deca-6,9-diene-2,8-dione, 7,9-di-tert-butyl- $$ 7,9-Di-tert-butyl-1

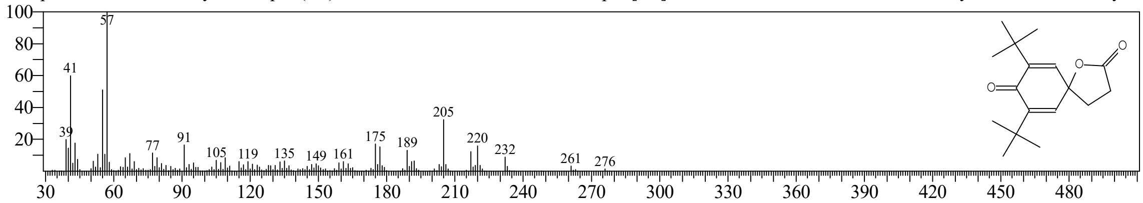

<< Target >>
Line#:21   R.Time:17.823(Scan#:3998) MassPeaks:269
RawMode:Averaged 17.820-17.827(3997-3999) BasePeak:131.10(70918)
BG Mode:Calc. from Peak Group 1 - Event 1 Q3 Scan

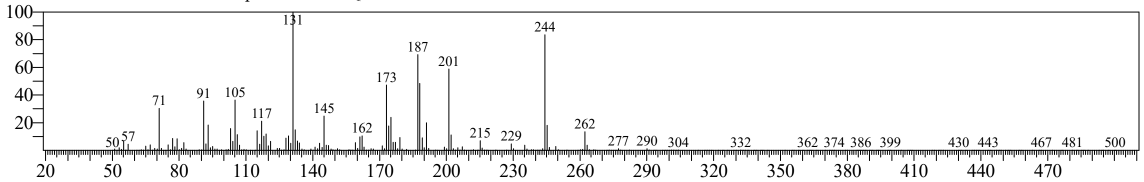

Hit#:1 Entry:88604 Library:NIST17-1.lib
SI:66 Formula:C16H20O2 CAS:0-00-0 MolWeight:244 RetIndex:2039
CompName:2,3-naphthalenedicarboxaldehyde, 5,6,7,8-tetrahydro-5,5,8,8-tetramethyl- $$ 5,5,8,8-tetramethyl-5,6,7,8-tetrahydronaphthalene-2,3-dicarbaldeh

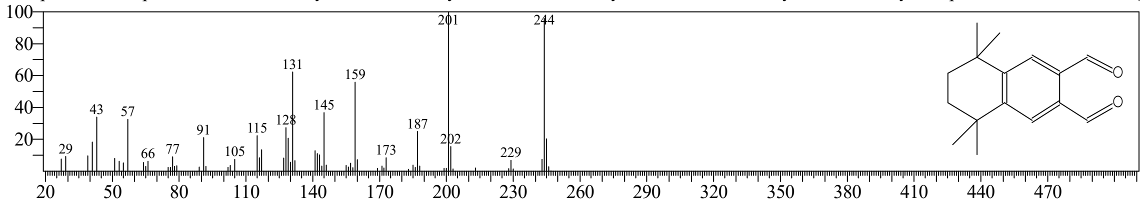

<< Target >>
Line#:22 R.Time:17.950(Scan#:4036) MassPeaks:299
RawMode:Averaged 17.947-17.953(4035-4037) BasePeak:223.20(61897)
BG Mode:Calc. from Peak Group 1 - Event 1 Q3 Scan

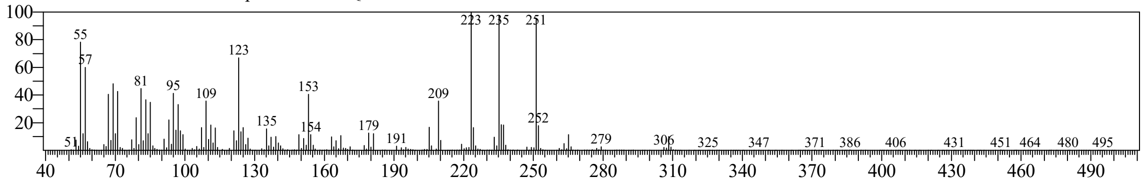

Hit#:1 Entry:30199 Library:NIST17s.lib
SI:68 Formula:C16H26O3 CAS:19780-11-1 MolWeight:266 RetIndex:2159
CompName:2-Dodecen-1-yl(-)succinic anhydride $$ 2,5-Furandione, 3-dodecenyl- $$ n-Dodecenylsuccinic anhydride $$ Dodecenyl succinic anhydride $$ 2

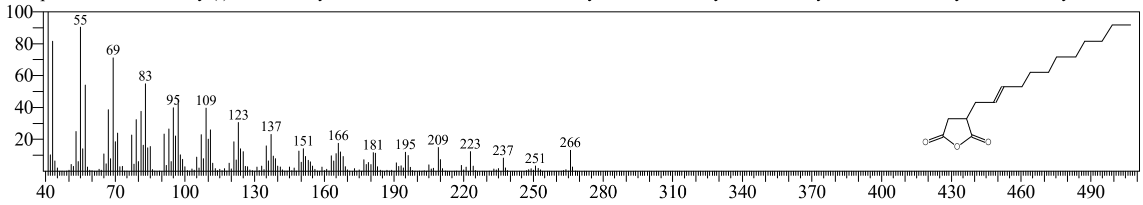

<< Target >>
Line#:23 R.Time:18.020(Scan#:4057) MassPeaks:231
RawMode:Averaged 18.017-18.023(4056-4058) BasePeak:223.20(76011)
BG Mode:Calc. from Peak Group 1 - Event 1 Q3 Scan

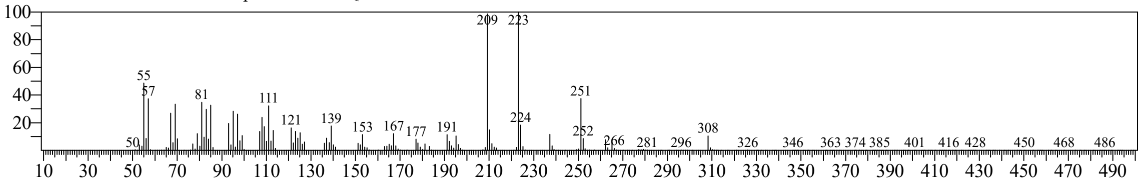

Hit#:1 Entry:70287 Library:NIST17-1.lib
SI:65 Formula:C13H20O3 CAS:28928-97-4 MolWeight:224 RetIndex:1861
CompName:n-Nonenylsuccinic anhydride $$ Nonenylsuccinic anhydride $$ 2,5-Furandione, dihydro-3-(nonenyl)- $$ 3-[(3E)-3-Nonenyl]dihydro-2,5-furand

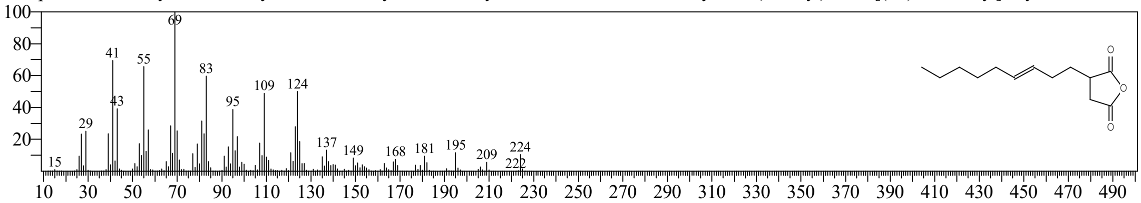

<< Target >>
Line#:24   R.Time:18.067(Scan#:4071) MassPeaks:279
RawMode:Averaged 18.063-18.070(4070-4072) BasePeak:71.10(255576)
BG Mode:Calc. from Peak Group 1 - Event 1 Q3 Scan

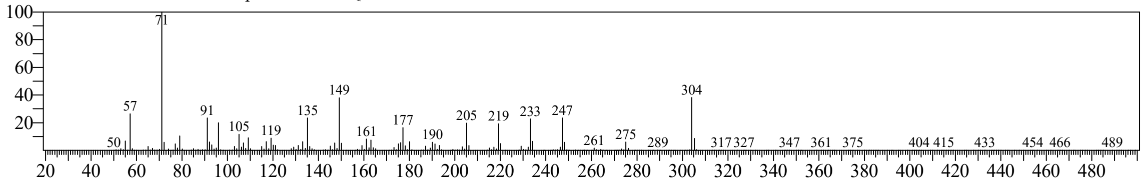

Hit#:1 Entry:66534 Library:NIST17-1.lib
SI:58 Formula:C14H20O2 CAS:71596-88-8 MolWeight:220 RetIndex:1632
CompName:Ethanone, 1-(5,6,7,8-tetrahydro-2,8,8-trimethyl-4H-cyclohepta[b]furan-5-yl)- $$ 1-(2,8,8-Trimethyl-5,6,7,8-tetrahydro-4H-cyclohepta[b]furan-5

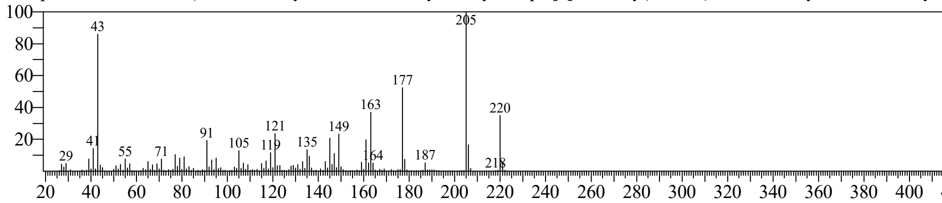

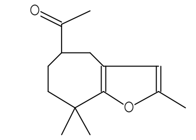

<< Target >>
Line#:25 R.Time:18.240(Scan#:4123) MassPeaks:220
RawMode:Averaged 18.237-18.243(4122-4124) BasePeak:71.05(176398)
BG Mode:Calc. from Peak Group 1 - Event 1 Q3 Scan

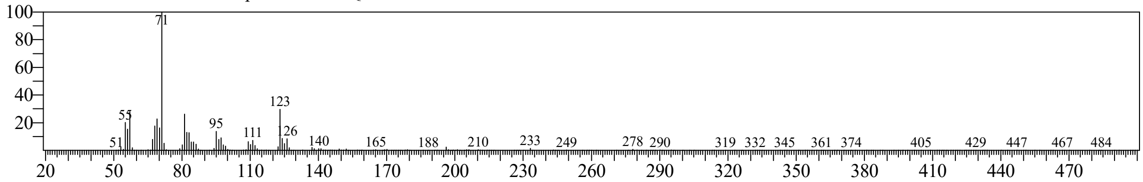

Hit#:1 Entry:32766 Library:NIST17s.lib
SI:96 Formula:C20H40O CAS:150-86-7 MolWeight:296 RetIndex:2045
CompName:Phytol $$ 2-Hexadecen-1-ol, 3,7,11,15-tetramethyl-, [R-[R*,R*-(E)]]- $$ trans-Phytol $$ 3,7,11,15-Tetramethyl-2-hexadecen-1-ol-, (2E,7R,11F

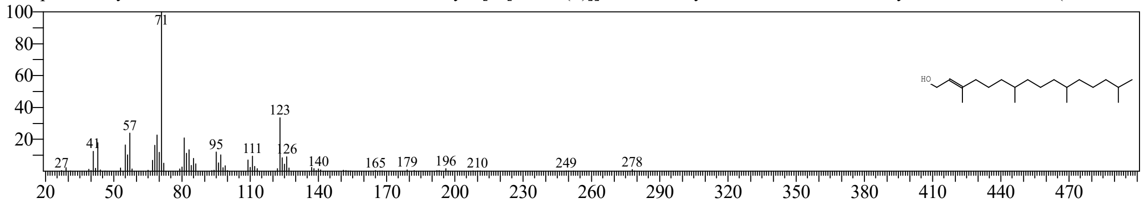

<< Target >>
Line#:26   R.Time:18.473(Scan#:4193) MassPeaks:318
RawMode:Averaged 18.470-18.477(4192-4194) BasePeak:67.05(294141)
BG Mode:Calc. from Peak Group 1 - Event 1 Q3 Scan

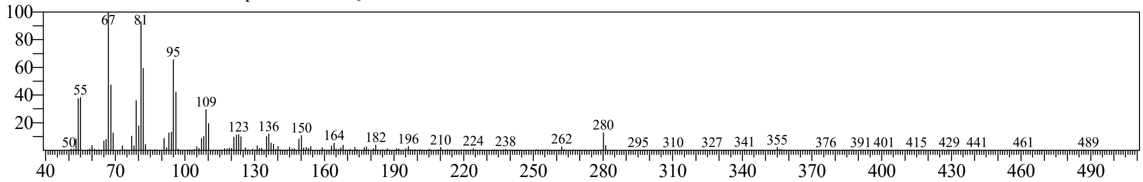

Hit#:1 Entry:31427 Library:NIST17s.lib
SI:93 Formula:C18H32O2 CAS:60-33-3 MolWeight:280 RetIndex:2183
CompName:9,12-Octadecadienoic acid (Z,Z)- $$ cis-9,cis-12-Octadecadienoic acid $$ cis,cis-Linoleic acid $$ Grape seed oil $$ Linoleic $$ Linoleic acid $

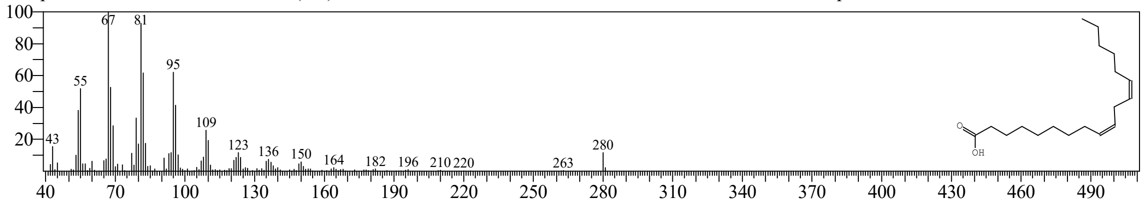

<< Target >>
Line#:27   R.Time:18.543(Scan#:4214) MassPeaks:351
RawMode:Averaged 18.540-18.547(4213-4215) BasePeak:55.05(494967)
BG Mode:Calc. from Peak Group 1 - Event 1 Q3 Scan

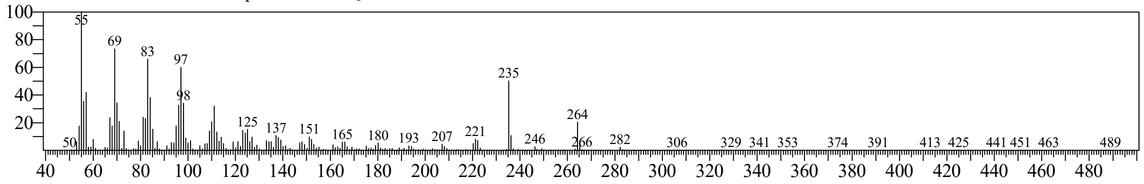

Hit#:1 Entry:31602 Library:NIST17s.lib
SI:91 Formula:C18H34O2 CAS:112-80-1 MolWeight:282 RetIndex:2175
CompName:Oleic Acid $$ 9-Octadecenoic acid (Z)- $$ .DELTA.9-cis-Oleic acid $$ cis-Oleic Acid $$ cis-9-Octadecenoic Acid $$ Emersol 211 $$ Emersol [illegible]

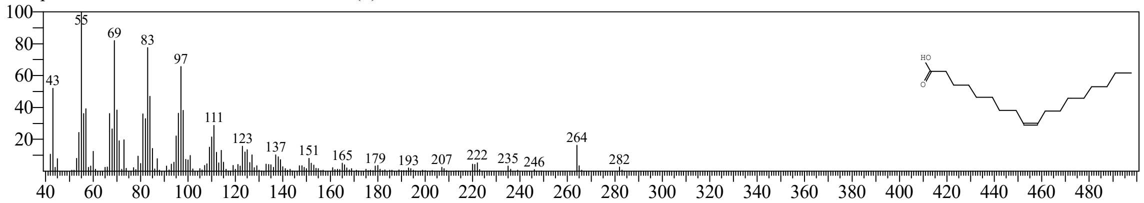

<< Target >>
Line#:28   R.Time:18.787(Scan#:4287) MassPeaks:328
RawMode:Averaged 18.783-18.790(4286-4288) BasePeak:193.15(220884)
BG Mode:Calc. from Peak Group 1 - Event 1 Q3 Scan

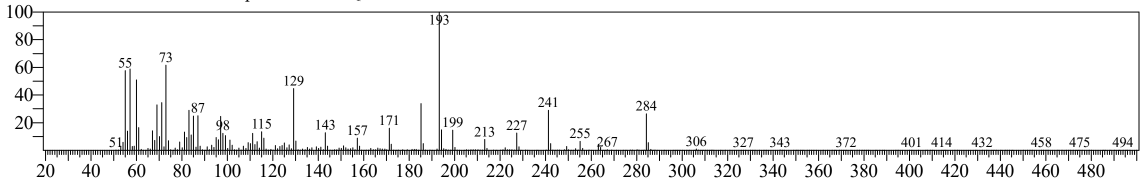

Hit#:1 Entry:31781 Library:NIST17s.lib
SI:86 Formula:C18H36O2 CAS:57-11-4 MolWeight:284 RetIndex:2167
CompName:Octadecanoic acid $$ Stearic acid $$ n-Octadecanoic acid $$ Humko Industrene R $$ Hydrofol Acid 150 $$ Hystrene S-97 $$ Hystrene T-70 $

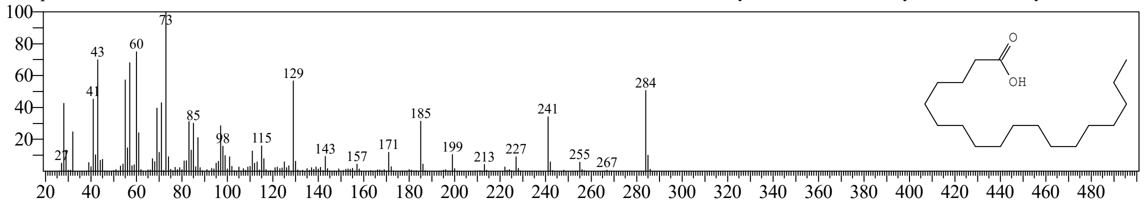

<< Target >>
Line#:29   R.Time:20.370(Scan#:4762) MassPeaks:297
RawMode:Averaged 20.367-20.373(4761-4763) BasePeak:57.10(349963)
BG Mode:Calc. from Peak Group 1 - Event 1 Q3 Scan

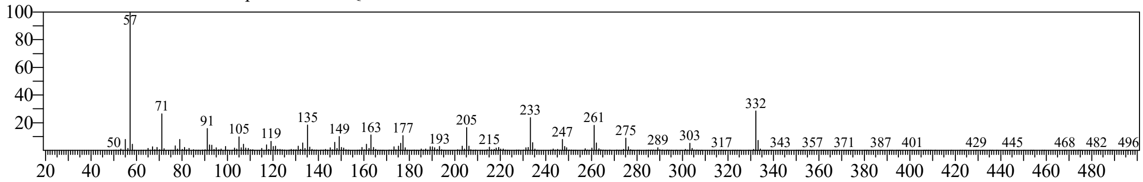

Hit#:1 Entry:176347 Library:NIST17-1.lib
SI:64 Formula:C22H36O2 CAS:0-00-0 MolWeight:332 RetIndex:2346
CompName:2H-1-benzopyran-6-ol, 3,4-dihydro-2,2-dimethyl-4-(1-methylethyl)-7-(1,1,3,3-tetramethylbutyl)- $$ 2,2-dimethyl-4-(propan-2-yl)-7-(2,4,4-trime

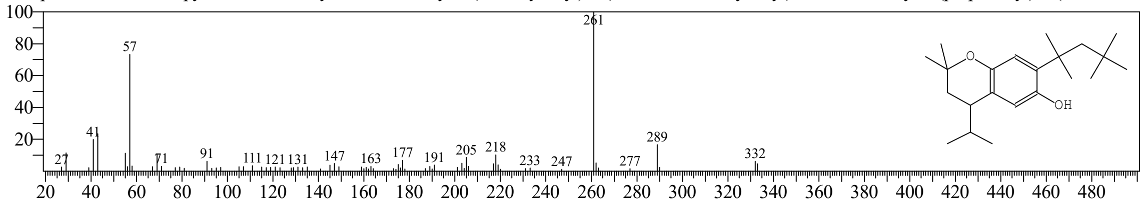

<< Target >>
Line#:30 R.Time:20.450(Scan#:4786) MassPeaks:315
RawMode:Averaged 20.447-20.453(4785-4787) BasePeak:57.10(226204)
BG Mode:Calc. from Peak Group 1 - Event 1 Q3 Scan

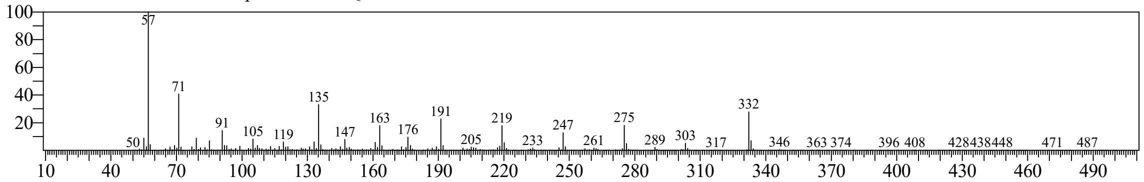

Hit#:1 Entry:116875 Library:NIST17-1.lib
SI:64 Formula:C10H21Cl3Si CAS:13829-21-5 MolWeight:274 RetIndex:1454
CompName:Silane, trichlorodecyl- $$ Decyltrichlorosilane $$ Trichloro(n-decyl)silane $$ n-Decyltrichlorosilane $$ Trichloro(decyl)silane # $$

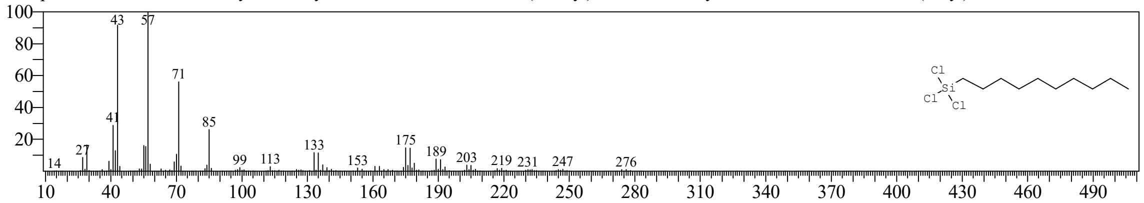

<< Target >>
Line#:31   R.Time:21.170(Scan#:5002) MassPeaks:291
RawMode:Averaged 21.167-21.173(5001-5003) BasePeak:251.20(88362)
BG Mode:Calc. from Peak Group 1 - Event 1 Q3 Scan

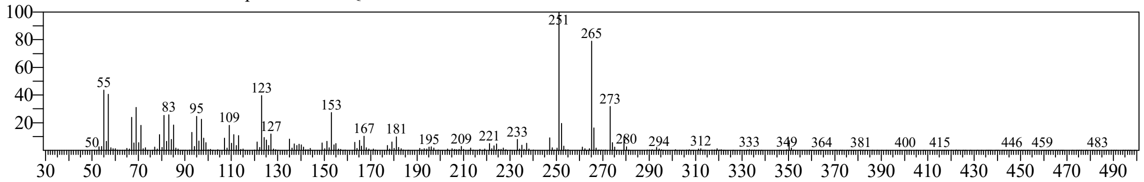

Hit#:1 Entry:109477 Library:NIST17-1.lib
SI:72 Formula:C17H30O2 CAS:18671-36-8 MolWeight:266 RetIndex:1942
CompName:14,15,16-Trinor-8.xi.-labdan-6.beta.-ol, 8,13-epoxy- $$ 4a,7,7,10a-Tetramethyldodecahydro-1H-benzo[f]chromen-6-ol # $$

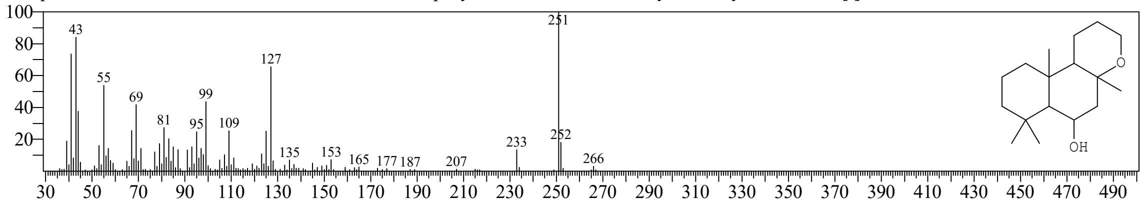

<< Target >>
Line#:32 R.Time:21.297(Scan#:5040) MassPeaks:281
RawMode:Averaged 21.293-21.300(5039-5041) BasePeak:273.15(99085)
BG Mode:Calc. from Peak Group 1 - Event 1 Q3 Scan

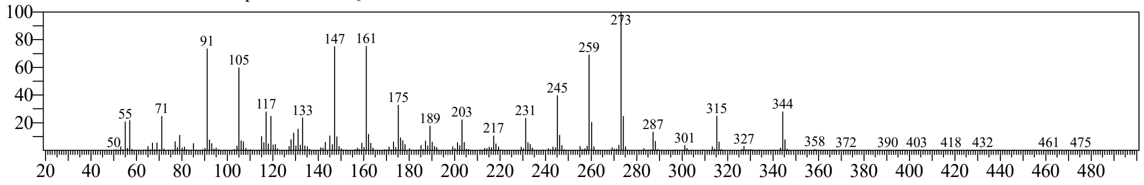

Hit#:1 Entry:131786 Library:NIST17-1.lib
SI:67 Formula:C21H36 CAS:78578-98-0 MolWeight:288 RetIndex:1663
CompName:Tetracyclo[6.1.0.0(2,4).0(5,7)]nonane, 3,3,6,6,9,9-hexaethyl-, cis,cis,trans-

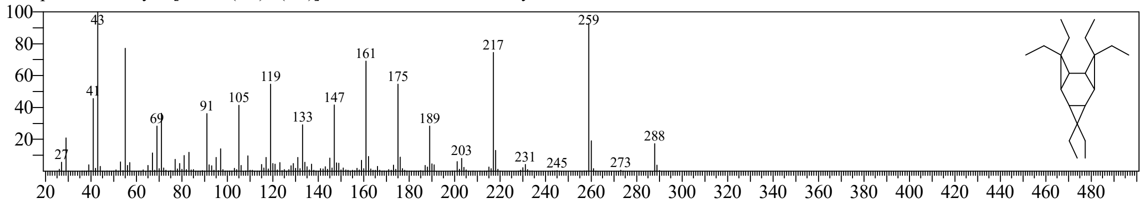

<< Target >>
Line#:33 R.Time:21.427(Scan#:5079) MassPeaks:259
RawMode:Averaged 21.423-21.430(5078-5080) BasePeak:71.10(194509)
BG Mode:Calc. from Peak Group 1 - Event 1 Q3 Scan

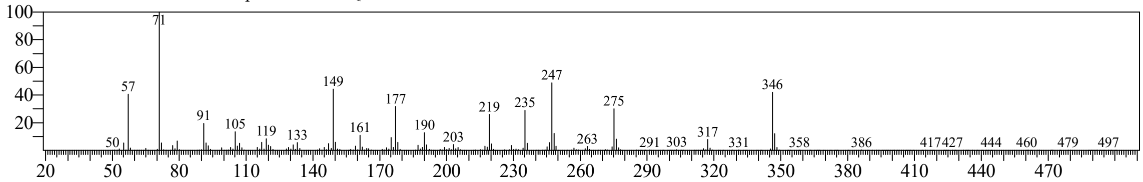

Hit#:1 Entry:189727 Library:NIST17-1.lib
SI:53 Formula:C23H42N2 CAS:0-00-0 MolWeight:346 RetIndex:2759
CompName:1,4-benzenediamine, 2-methyl-N4,N4-dioctyl- $$ 2-methyl-N4,N4-dioctylbenzene-1,4-diamine $$

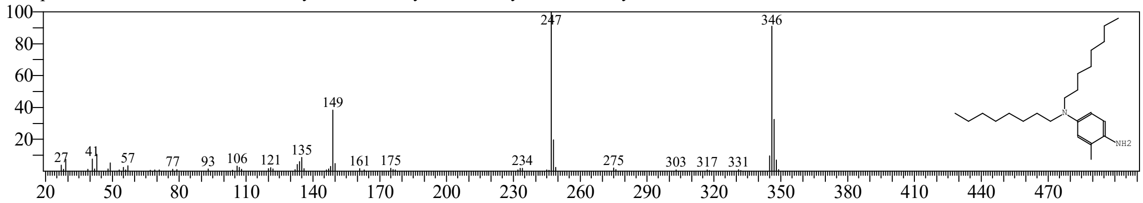

<< Target >>
Line#:34   R.Time:21.463(Scan#:5090) MassPeaks:283
RawMode:Averaged 21.460-21.467(5089-5091) BasePeak:251.20(44296)
BG Mode:Calc. from Peak Group 1 - Event 1 Q3 Scan

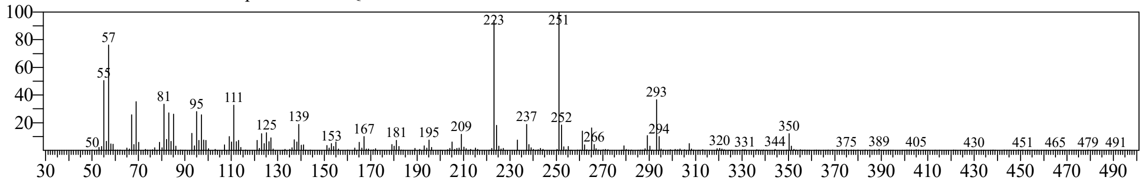

Hit#:1 Entry:204223 Library:NIST17-1.lib
SI:66 Formula:C26H52 CAS:55334-19-5 MolWeight:364 RetIndex:2631
CompName:Cyclohexane, 1,3-didecyl- $$ 1,3-Di-n-decylcyclohexane $$ 1,3-Didecylcyclohexane # $$

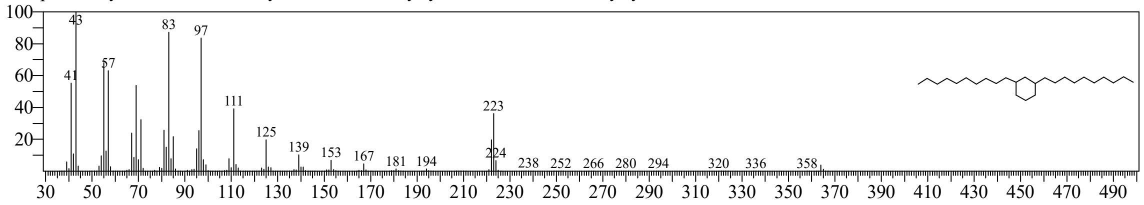

<< Target >>
Line#:35 R.Time:21.517(Scan#:5106) MassPeaks:299
RawMode:Averaged 21.513-21.520(5105-5107) BasePeak:71.10(233899)
BG Mode:Calc. from Peak Group 1 - Event 1 Q3 Scan

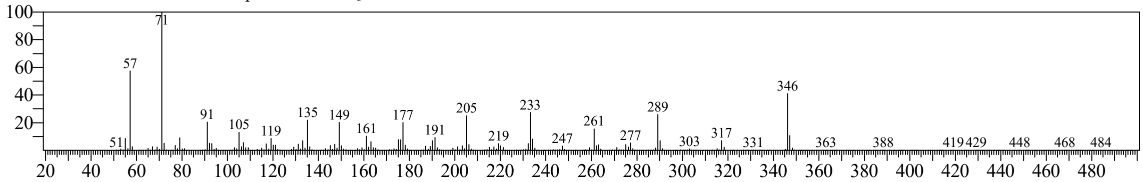

Hit#:1 Entry:176347 Library:NIST17-1.lib
SI:58 Formula:C22H36O2 CAS:0-00-0 MolWeight:332 RetIndex:2346
CompName:2H-1-benzopyran-6-ol, 3,4-dihydro-2,2-dimethyl-4-(1-methylethyl)-7-(1,1,3,3-tetramethylbutyl)- $$ 2,2-dimethyl-4-(propan-2-yl)-7-(2,4,4-trime

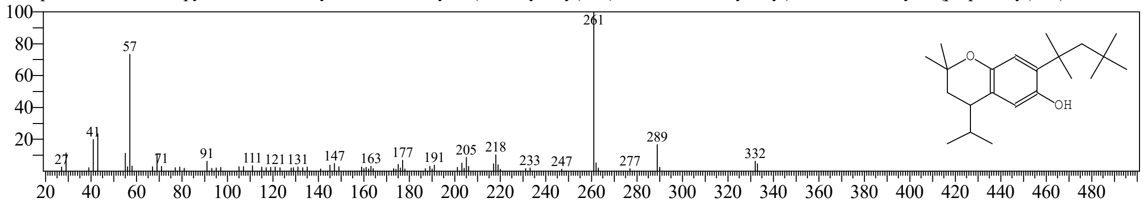

<< Target >>
Line#:36   R.Time:21.967(Scan#:5241) MassPeaks:270
RawMode:Averaged 21.963-21.970(5240-5242) BasePeak:257.15(712371)
BG Mode:Calc. from Peak Group 1 - Event 1 Q3 Scan

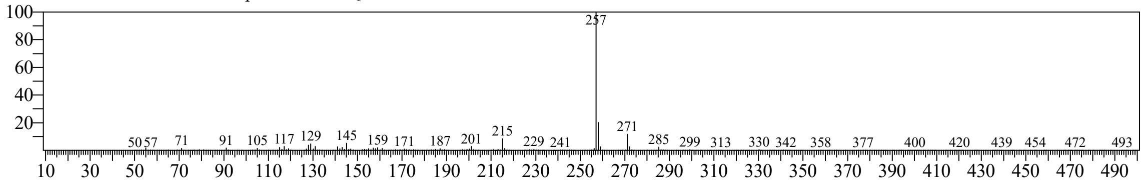

Hit#:1 Entry:247258 Library:NIST17-1.lib
SI:77 Formula:C31H33NO2 CAS:82406-82-4 MolWeight:451 RetIndex:3731
CompName:Benzoic acid, 4-(4-pentylcyclohexyl)-, 4'-cyano[1,1'-biphenyl]-4-yl ester $$ 4'-Cyano[1,1'-biphenyl]-4-yl 4-(4-pentylcyclohexyl)benzoate # $$

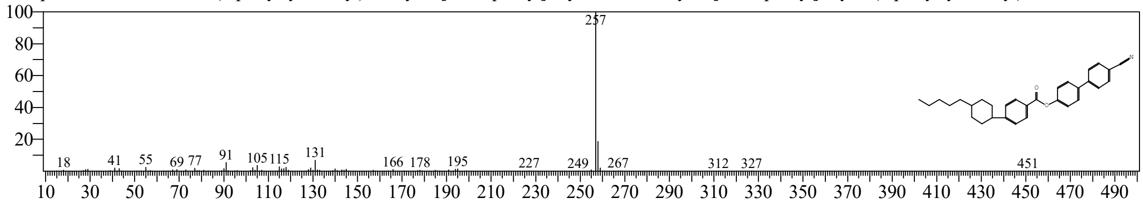

<< Target >>
Line#:37   R.Time:23.120(Scan#:5587) MassPeaks:348
RawMode:Averaged 23.117-23.123(5586-5588) BasePeak:267.20(103380)
BG Mode:Calc. from Peak Group 1 - Event 1 Q3 Scan

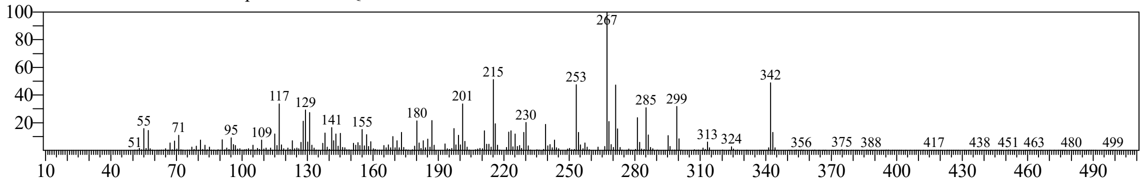

Hit#:1 Entry:186037 Library:NIST17-1.lib
SI:55 Formula:C22H30O3 CAS:57397-35-0 MolWeight:342 RetIndex:2555
CompName:4(1H)-Phenanthrenone, 7-(acetyloxy)-2,3,4a,9,10,10a-hexahydro-1,1,4a-trimethyl-8-(1-methylethyl)-, (4aS-trans)- $$ 14-Isopropyl-1-oxopodoc

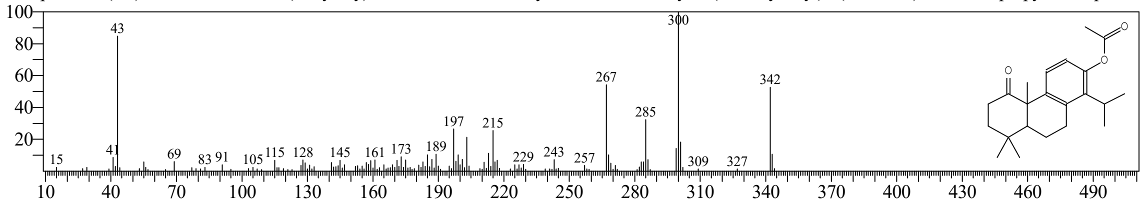

<< Target >>
Line#:38   R.Time:23.597(Scan#:5730) MassPeaks:360
RawMode:Averaged 23.593-23.600(5729-5731) BasePeak:57.10(254664)
BG Mode:Calc. from Peak Group 1 - Event 1 Q3 Scan

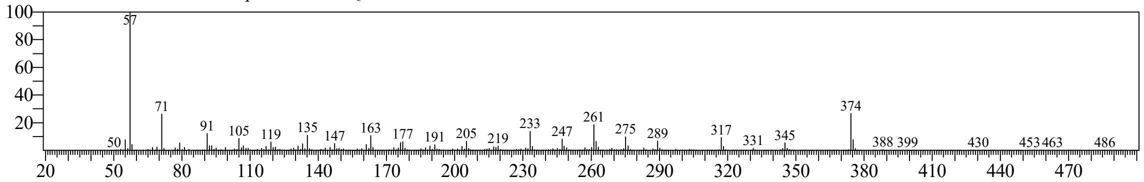

Hit#:1 Entry:176347 Library:NIST17-1.lib
SI:64 Formula:C22H36O2 CAS:0-00-0 MolWeight:332 RetIndex:2346
CompName:2H-1-benzopyran-6-ol, 3,4-dihydro-2,2-dimethyl-4-(1-methylethyl)-7-(1,1,3,3-tetramethylbutyl)- $$ 2,2-dimethyl-4-(propan-2-yl)-7-(2,4,4-trime

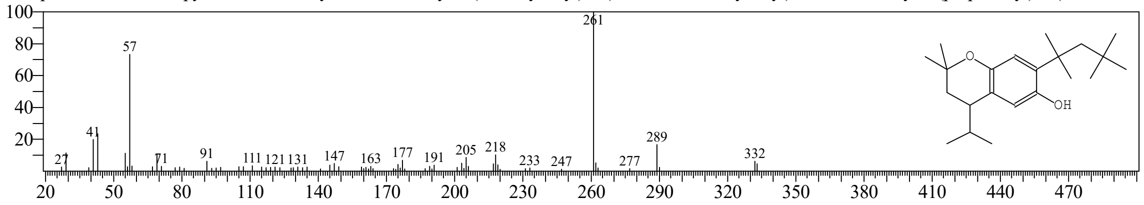

<< Target >>
Line#:39 R.Time:23.803(Scan#:5792) MassPeaks:335
RawMode:Averaged 23.800-23.807(5791-5793) BasePeak:299.20(208335)
BG Mode:Calc. from Peak Group 1 - Event 1 Q3 Scan

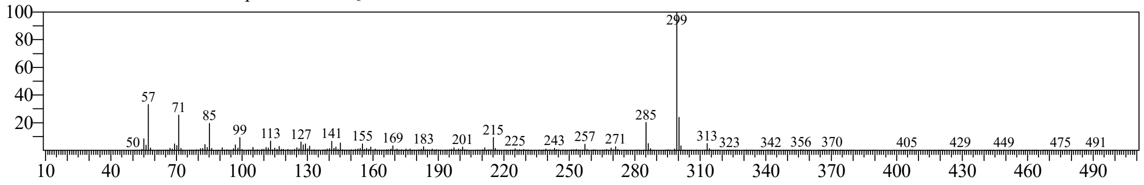

Hit#:1 Entry:238978 Library:NIST17-1.lib
SI:69 Formula:C23H35ClO5 CAS:0-00-0 MolWeight:426 RetIndex:2974
CompName:Succinic acid, 2-(2-chlorophenoxy)ethyl undecyl ester

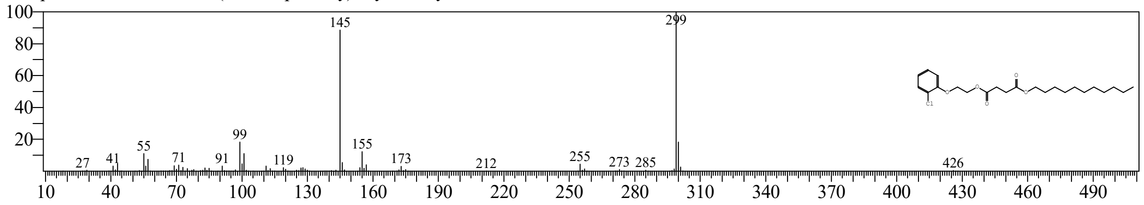

<< Target >>
Line#:40 R.Time:24.377(Scan#:5964) MassPeaks:348
RawMode:Averaged 24.373-24.380(5963-5965) BasePeak:315.25(68164)
BG Mode:Calc. from Peak Group 1 - Event 1 Q3 Scan

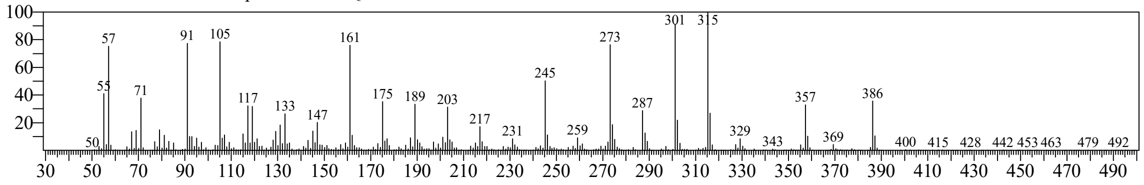

Hit#:1 Entry:37237 Library:NIST17s.lib
SI:59 Formula:C25H38O3 CAS:112924-45-5 MolWeight:386 RetIndex:3031
CompName:(6aS,10aS)-9-(Hydroxymethyl)-6,6-dimethyl-3-(2-methyloctan-2-yl)-6a,7,10,10a-tetrahydrobenzo[c]chromen-1-ol $$ HU-211 $$

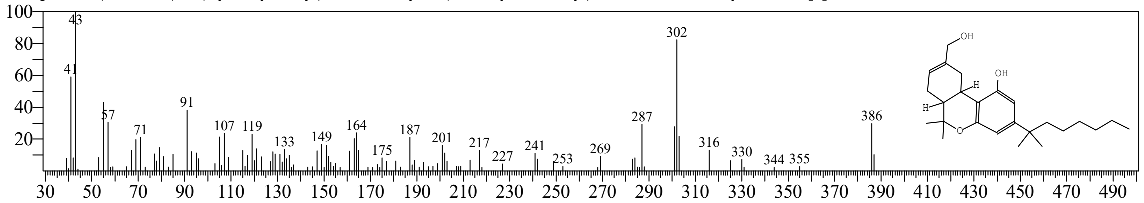

<< Target >>
Line#:41 R.Time:24.540(Scan#:6013) MassPeaks:338
RawMode:Averaged 24.537-24.543(6012-6014) BasePeak:71.10(178077)
BG Mode:Calc. from Peak Group 1 - Event 1 Q3 Scan

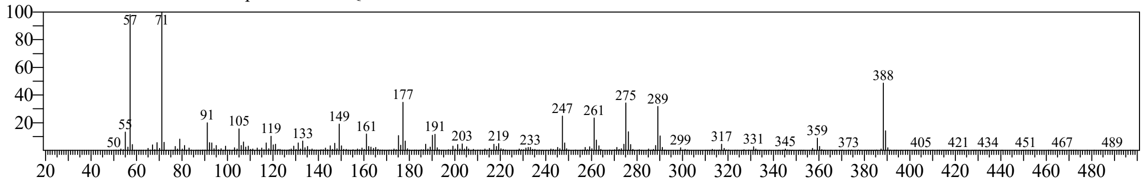

Hit#:1 Entry:176347 Library:NIST17-1.lib
SI:57 Formula:C22H36O2 CAS:0-00-0 MolWeight:332 RetIndex:2346
CompName:2H-1-benzopyran-6-ol, 3,4-dihydro-2,2-dimethyl-4-(1-methylethyl)-7-(1,1,3,3-tetramethylbutyl)- $$ 2,2-dimethyl-4-(propan-2-yl)-7-(2,4,4-trime

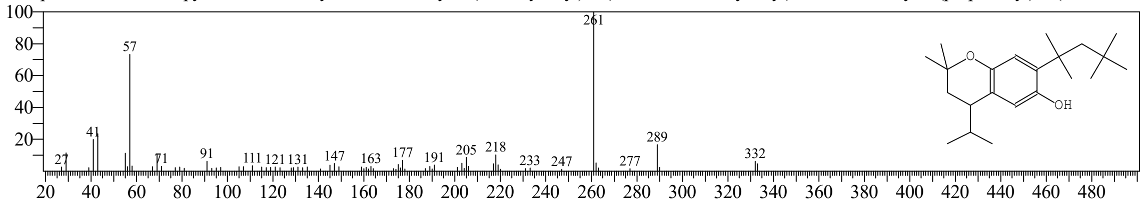

<< Target >>
Line#:42 R.Time:24.770(Scan#:6082) MassPeaks:298
RawMode:Averaged 24.767-24.773(6081-6083) BasePeak:299.20(659985)
BG Mode:Calc. from Peak Group 1 - Event 1 Q3 Scan

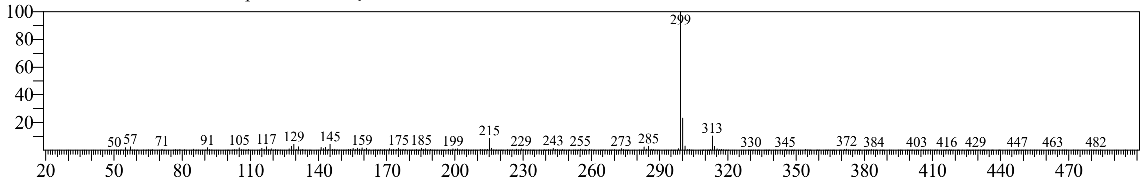

Hit#:1 Entry:185866 Library:NIST17-1.lib
SI:74 Formula:C20H42O2Si CAS:0-00-0 MolWeight:342 RetIndex:1997
CompName:Silane, methylvinyl(hept-4-yloxy)decyloxy-

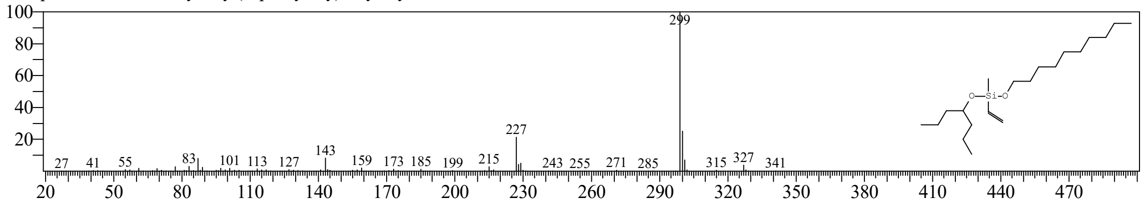

<< Target >>
Line#:43   R.Time:26.047(Scan#:6465) MassPeaks:349
RawMode:Averaged 26.043-26.050(6464-6466) BasePeak:215.15(49348)
BG Mode:Calc. from Peak Group 1 - Event 1 Q3 Scan

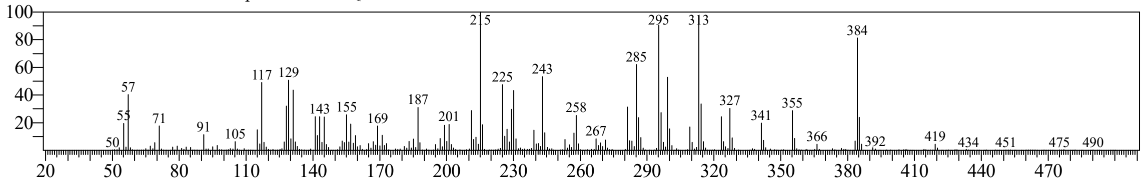

Hit#:1 Entry:88610 Library:NIST17-1.lib
SI:45 Formula:C16H20O2 CAS:38701-10-9 MolWeight:244 RetIndex:0
CompName:2,3-Hexadienoic acid, 2-ethyl-4-phenyl-, ethyl ester $$ Ethyl 2-ethyl-4-phenyl-2,3-hexadienoate # $$

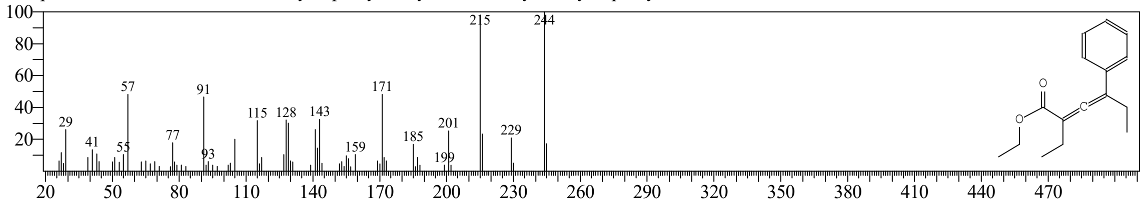

<< Target >>
Line#:44   R.Time:28.963(Scan#:7340) MassPeaks:361
RawMode:Averaged 28.960-28.967(7339-7341) BasePeak:395.30(152921)
BG Mode:Calc. from Peak Group 1 - Event 1 Q3 Scan

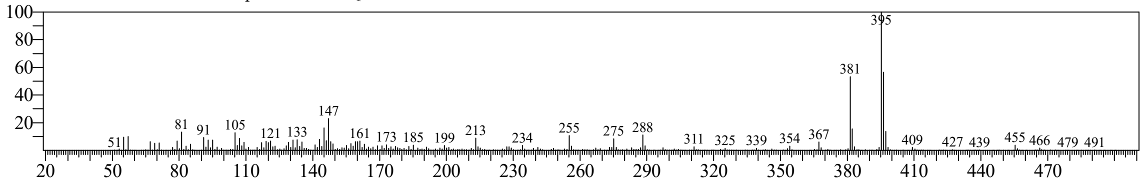

Hit#:1 Entry:252280 Library:NIST17-1.lib
SI:77 Formula:C32H54O2 CAS:0-00-0 MolWeight:470 RetIndex:2970
CompName:.beta.-Sitosterol, propionate

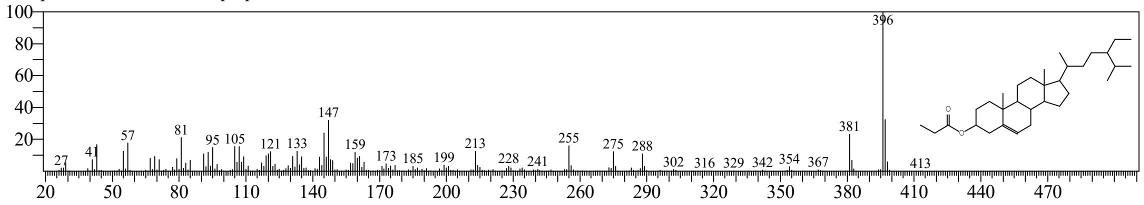

<< Target >>
Line#:45 R.Time:30.167(Scan#:7701) MassPeaks:367
RawMode:Averaged 30.163-30.170(7700-7702) BasePeak:315.25(76902)
BG Mode:Calc. from Peak Group 1 - Event 1 Q3 Scan

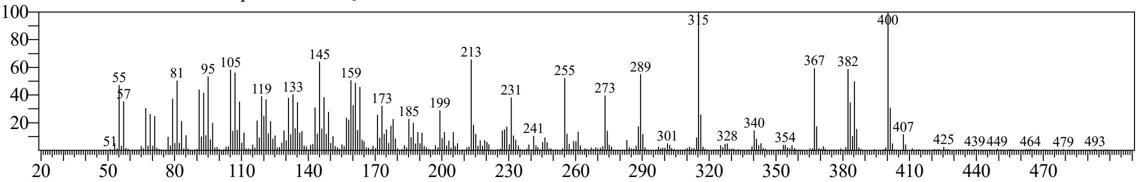

Hit#:1 Entry:37658 Library:NIST17s.lib
SI:90 Formula:C28H48O CAS:474-62-4 MolWeight:400 RetIndex:2632
CompName:Campesterol $$ Ergost-5-en-3-ol, (3.beta.,24R)- $$ Ergost-5-en-3.beta.-ol, (24R)- $$ (24R)-5-Ergosten-3.beta.-ol $$ Campesterin $$ 24.alpha.-

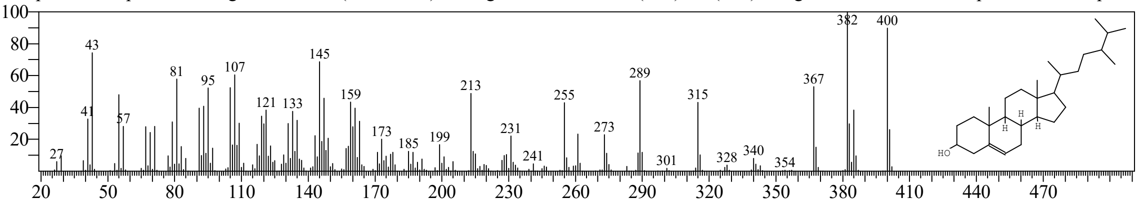

<< Target >>
Line#:46   R.Time:30.450(Scan#:7786) MassPeaks:384
RawMode:Averaged 30.447-30.453(7785-7787) BasePeak:55.05(72368)
BG Mode:Calc. from Peak Group 1 - Event 1 Q3 Scan

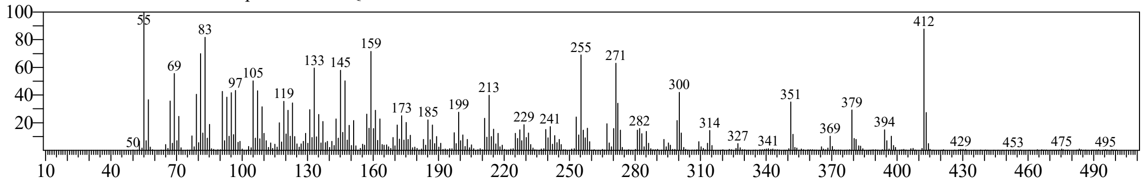

Hit#:1 Entry:37859 Library:NIST17s.lib
SI:82 Formula:C29H48O CAS:83-48-7 MolWeight:412 RetIndex:2739
CompName:Stigmasterol $$ Stigmasta-5,22-dien-3-ol, (3.beta.,22E)- $$ Stigmasta-5,22-dien-3.beta.-ol $$ .beta.-Stigmasterol $$ (24S)-5,22-Stigmastadien-

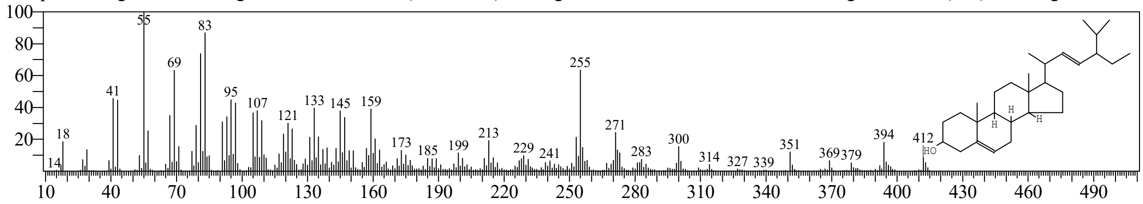

<< Target >>
Line#:47   R.Time:30.767(Scan#:7881) MassPeaks:358
RawMode:Averaged 30.763-30.770(7880-7882) BasePeak:411.30(135560)
BG Mode:Calc. from Peak Group 1 - Event 1 Q3 Scan

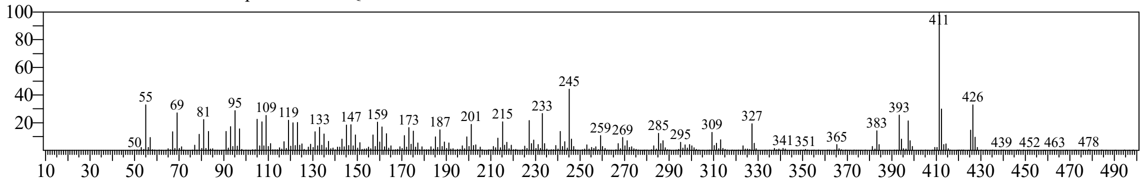

Hit#:1 Entry:239409 Library:NIST17-1.lib
SI:85 Formula:C30H50O CAS:16910-32-0 MolWeight:426 RetIndex:2826
CompName:Obtusifoliol $$ Ergosta-8,24(28)-dien-3-ol, 4,14-dimethyl-, (3.beta.,4.alpha.,5.alpha.)- $$ 5.alpha.-Ergosta-8,24(28)-dien-3.beta.-ol, 4.alpha.,14-

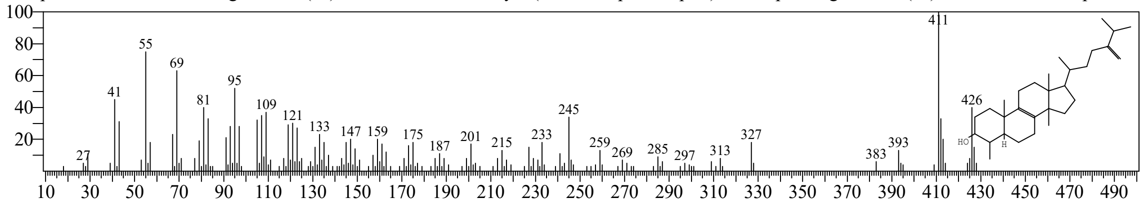

<< Target >>
Line#:48   R.Time:30.973(Scan#:7943) MassPeaks:436
RawMode:Averaged 30.970-30.977(7942-7944) BasePeak:414.35(795162)
BG Mode:Calc. from Peak Group 1 - Event 1 Q3 Scan

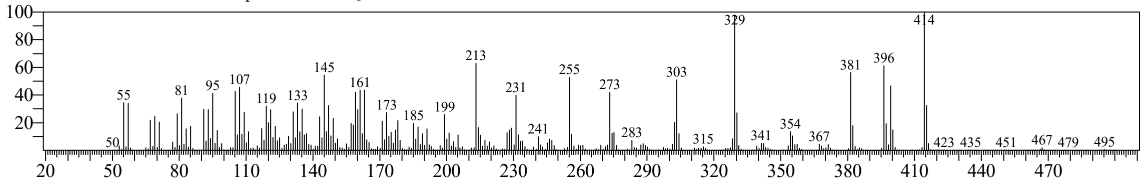

Hit#:1 Entry:37913 Library:NIST17s.lib
SI:87 Formula:C29H50O CAS:83-47-6 MolWeight:414 RetIndex:2731
CompName:.gamma.-Sitosterol $$ Stigmast-5-en-3-ol, (3.beta.,24S)- $$ Stigmast-5-en-3.beta.-ol, (24S)- $$ Clionasterol $$ Fucosterol, .beta.-dihydro- $$ 2

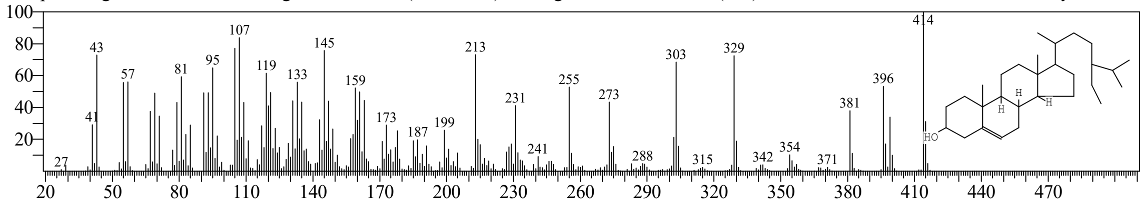

<< Target >>
Line#:49   R.Time:31.457(Scan#:8088) MassPeaks:378
RawMode:Averaged 31.453-31.460(8087-8089) BasePeak:414.35(211833)
BG Mode:Calc. from Peak Group 1 - Event 1 Q3 Scan

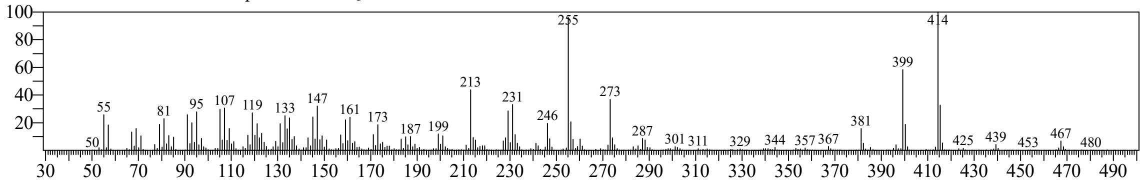

Hit#:1 Entry:234573 Library:NIST17-1.lib
SI:87 Formula:C29H50O CAS:18525-35-4 MolWeight:414 RetIndex:2731
CompName:Stigmast-7-en-3-ol, (3.beta.,5.alpha.,24S)- $$ 5.alpha.-Stigmast-7-en-3.beta.-ol, (24S)- $$ .delta.7-Chondrillastenol $$ Chondrillast-7-enol $$ 2[illegible]

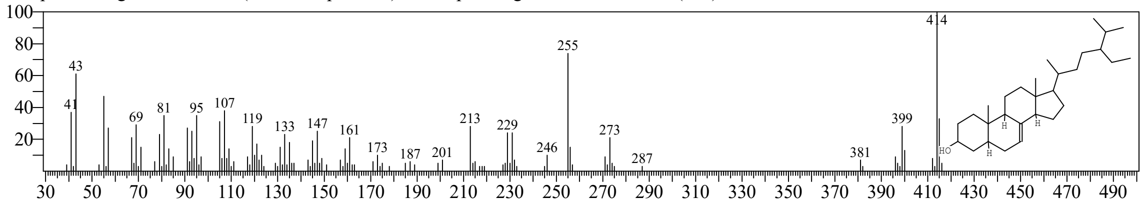

<< Target >>
Line#:50 R.Time:32.150(Scan#:8296) MassPeaks:378
RawMode:Averaged 32.147-32.153(8295-8297) BasePeak:285.20(226752)
BG Mode:Calc. from Peak Group 1 - Event 1 Q3 Scan

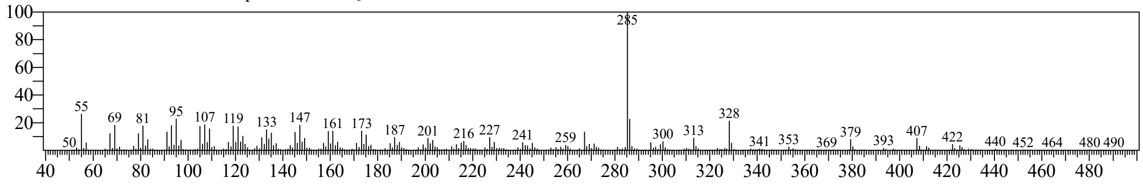

Hit#:1 Entry:229688 Library:NIST17-1.lib
SI:71 Formula:C23H32O6 CAS:0-00-0 MolWeight:404 RetIndex:2777
CompName:Pregnan-17,21-diol-9,11-epoxy-3,20-dione, acetate $$ 2-(7-Hydroxy-4a,6a-dimethyl-2-oxohexadecahydrocyclopenta[7,8]phenanthro[4b,5-b]ox

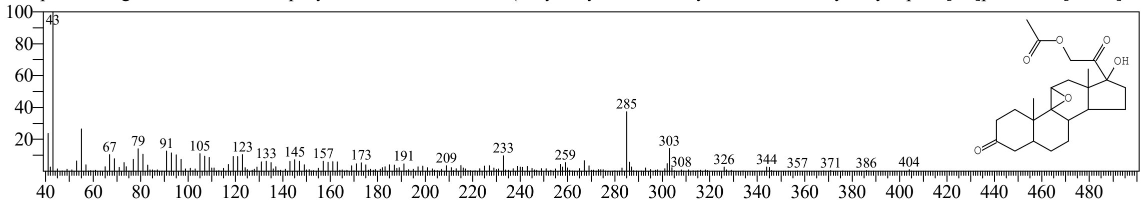

Supplement: Supplementary file 1 [file ijms-26-09156-s001.zip › MS OSO 240min.pdf]
