# Supplementary material for: Ozonized Sunflower Oil: Standardization and Mechanisms of the Antimicrobial Effect
Source: Int J Mol Sci. 2025 Sep 19;26(18):9156. doi: 10.3390/ijms26189156 (PMC12470628; doi:10.3390/ijms26189156)

Centro Integrado de Análises - CIA-FURG
Laboratório de Cromatografia Gasosa - GC-MS/MS
Visite o nosso site - www.cia.furg.br

Relatório de análise - GC-MS/MS

Sample Information

Analyzed by	: Jean Arias
Analyzed	: 12/10/2023 13:59:08
Sample Type	: Unknown
Level #	: 1
Sample Name	: 11102023_OVgirassol_480min_MI
Sample ID	:
Rack	: Rack 2
IS Amount	: [1]=1
Sample Amount	: 1
Dilution Factor	: 1
Vial #	: 15
Injection Volume	: 1.00
Data File	: C:\GCMSsolution\Data\Óleos - ácidos graxos\11102023_amostras_óleos\11102023_OVgirassol_480min_MI.qgd
Org Data File	: C:\GCMSsolution\Data\Óleos - ácidos graxos\11102023_amostras_óleos\11102023_OVgirassol_480min_MI.qgd
Method File	: C:\GCMSsolution\Data\Óleos - ácidos graxos\Matéria insaponificável - scan.qgm
Org Method File	: C:\GCMSsolution\Data\Óleos - ácidos graxos\Matéria insaponificável - scan.qgm
Report File	:
Tuning File	: C:\GCMSsolution\System\Tune1\10102023_tuning.qgt
Modified by	: Jean Arias
Modified	: 18/10/2023 14:40:37

Method

[Comment]

===== Analytical Line 1 =====

[GC-2010]

Column Oven Temp.	:80.0 °C
Injection Temp.	:250.00 °C
Injection Mode	:Split
Flow Control Mode	:Linear Velocity
Pressure	:88.5 kPa
Total Flow	:17.3 mL/min
Column Flow	:1.30 mL/min
Linear Velocity	:42.0 cm/sec
Purge Flow	:3.0 mL/min
Split Ratio	:10.0
High Pressure Injection	:ON
High Press. Inj. Pressure	:300.0 kPa
High Press. Inj. Time	:2.30 min
Carrier Gas Saver	:OFF
Splitter Hold	:OFF

Oven Temp. Program

Rate	Temperature(°C)	Hold Time(min)
-	80.0	1.00
10.00	180.0	0.00
7.00	330.0	0.00

< Ready Check Heat Unit >
Column Oven : Yes
SPL1 : Yes
MS : Yes
< Ready Check Detector(FTD/BID) >
< Ready Check Baseline Drift >
< Ready Check Injection Flow >
SPL1 Carrier : Yes
SPL1 Purge : Yes
< Ready Check APC Flow >
< Ready Check Detector APC Flow >
External Wait :No
Equilibrium Time :3.0 min

[GC Program]

[GCMS-TQ8050]
IonSourceTemp :230.00 °C

Interface Temp.	:280.00 °C
Solvent Cut Time	:4.00 min
Detector Gain Mode	:Relative to the Tuning Result
Detector Gain	:1.03 kV +0.00 kV
Threshold	:0
Acquire Data without Using CID Gas(Q3Scan)	:ON

[MS Table]
--Group 1 - Event 1--

Compound Name	:
Start Time	:4.50min
End Time	:32.40min
Acq. Mode	:Q3 Scan
Event Time	:0.200sec
Scan Speed	:2500
Start m/z	:50.00
End m/z	:500.00
Q1 Resolution	: -
Q3 Resolution	: -

Sample Inlet Unit :GC

[MS Program]
Use MS Program :OFF

Peak Report TIC

Peak#	R.Time	Area	Area%	Name	IR
1	5.673	3516171	0.43	1-Nonanol	
2	8.376	4197129	0.52	Cyclooctyl alcohol	
3	8.887	105343439	12.94	2-Octenal, 2-butyl-	
4	9.258	3041986	0.37	Octadecanal, 2-bromo-	
5	10.126	4714736	0.58	2-Nonenal, 2-pentyl-	
6	10.182	3954117	0.49	2-Octenal, 2-butyl-	
7	11.345	3102810	0.38	2-Octenal, 2-butyl-	
8	11.388	4194471	0.52	2-Nonenal, 2-pentyl-	
9	12.162	3633249	0.45	4,5-Heptadien-2-one, 3,3,6-trimethyl-	
10	12.226	7429140	0.91	2-Propylcyclohexanol	
11	12.288	6047190	0.74	2-Propylcyclohexanol	
12	12.616	138698056	17.03	2-Nonenal, 2-pentyl-	
13	12.742	133976435	16.45	2-Octenal, 2-butyl-	
14	12.894	3946949	0.48	Oxirane, hexadecyl-	
15	13.016	6009239	0.74	Oxirane, hexadecyl-	
16	13.423	4875184	0.60	Dill ether	
17	13.814	4124564	0.51	2-Nonenal, 2-pentyl-	
18	13.877	5644155	0.69	2-Nonenal, 2-pentyl-	
19	14.530	3338316	0.41	2-Pentanone, 4-cyclohexylidene-3,3-diethyl-	
20	15.089	6604651	0.81	2-Nonenal, 2-pentyl-	
21	15.145	2952339	0.36	2-Amino-4-benzylthiomethyl-6-piperidino-1,3,5-triazine	
22	15.807	3398089	0.42	2H-1-Benzopyran, 3,4,4a,5,6,8a-hexahydro-2,5,5,8a-tet	
23	16.043	8464491	1.04	Pentadecanal-	
24	16.283	6619750	0.81	n-Hexadecanoic acid	
25	16.384	153141924	18.80	2,5-Furandione, 3-dodecyl-	
26	16.580	3923708	0.48	2,5-Furandione, 3-dodecyl-	
27	16.622	4539764	0.56	Oxirane, hexadecyl-	
28	16.909	5038810	0.62	2,4,5-Trimethylaniline, N-trimethylacetyl-	
29	17.056	4047953	0.50	2H-1-Benzopyran, 3,4,4a,5,6,8a-hexahydro-2,5,5,8a-tet	
30	17.955	3255471	0.40	Silane, chlorodiethylnonyloxy-	
31	18.023	4184140	0.51	Cyclohexadecadiene-1,6-dione, 3,4-diethyl-, cis-	
32	18.070	6257068	0.77	Ethanone, 1-(5,6,7,8-tetrahydro-2,8,8-trimethyl-4H-cyclo	
33	18.509	5207816	0.64	9-Octadecenoic acid, (E)-	
34	18.560	6254893	0.77	Benzoic acid, 4-[(2,4-dimethoxy-6-pentylbenzoyl)oxy]-2-	
35	18.779	7494518	0.92	Octadecanoic acid	
36	20.369	8429905	1.04	2H-1-benzopyran-6-ol, 3,4-dihydro-2,2-dimethyl-4-(1-me	
37	20.454	5296056	0.65	2,4,5-Trimethylaniline, N-trimethylacetyl-	
38	21.430	9840572	1.21	9-t-Butyl-4-iodo-2,2-dimethyladamantane	
39	21.516	4571462	0.56	7,9-Di-tert-butyl-1-oxaspiro(4,5)deca-6,9-diene-2,8-dion	
40	21.909	3656860	0.45	Benzoic acid, 4-[(2,4-dimethoxy-6-pentylbenzoyl)oxy]-2-	
41	23.517	2938914	0.36	7,9-Di-tert-butyl-1-oxaspiro(4,5)deca-6,9-diene-2,8-dion	
42	23.592	6023258	0.74	2H-1-benzopyran-6-ol, 3,4-dihydro-2,2-dimethyl-4-(1-me	
43	24.282	3036350	0.37	i-Propyl 11,12-methylene-octadecanoate	
44	24.541	4233569	0.52	2H-1-benzopyran-6-ol, 3,4-dihydro-2,2-dimethyl-4-(1-me	
45	30.168	5790967	0.71	Campesterol	
46	30.448	5596058	0.69	Stigmasterol	
47	30.971	55827797	6.86	.gamma.-Sitosterol	
48	31.455	6889155	0.85	Stigmast-7-en-3-ol, (3.beta.,5.alpha.,24S)-	
49	32.144	5233343	0.64	9,19-Cyclolanostan-3-ol, 24-methylene-, (3.beta.)-	
50	32.327	5865494	0.72	1.alpha.-Methyl-5.alpha.-androstan-3.alpha.,17.beta.-di	
		814402481	100.00		

Chromatogram 11102023_OVgirassol_480min_MI C:\GCMSsolution\Data\Óleos - ácidos graxos\11102023_amostras_óleos\11102023_OVgirassol_480min_MI.qgd

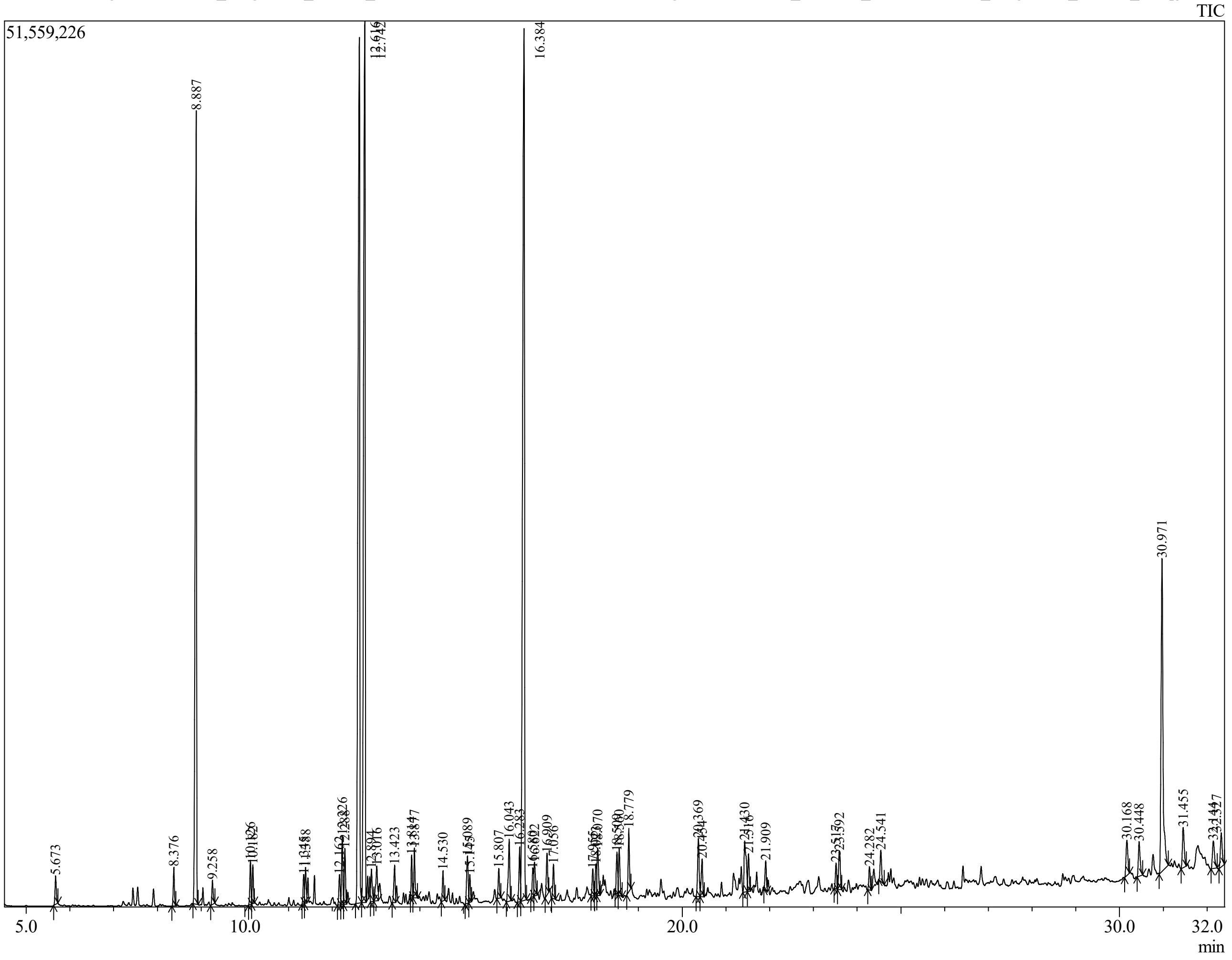

Library

<< Target >>
Line#:1 R.Time:5.673(Scan#:353) MassPeaks:256
RawMode:Averaged 5.670-5.677(352-354) BasePeak:56.05(299826)
BG Mode:Calc. from Peak Group 1 - Event 1 Q3 Scan

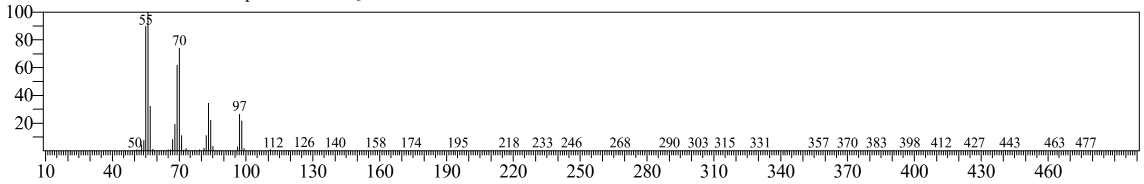

Hit#:1 Entry:9604 Library:NIST17s.lib
SI:98 Formula:C9H20O CAS:143-08-8 MolWeight:144 RetIndex:1159
CompName:1-Nonanol $$ Nonyl alcohol $$ n-Nonyl alcohol $$ Octyl carbinol $$ Pelargonic alcohol $$ Alcohol C-9 $$ Nonan-1-ol $$ Nonanol-(1) $$ n-N

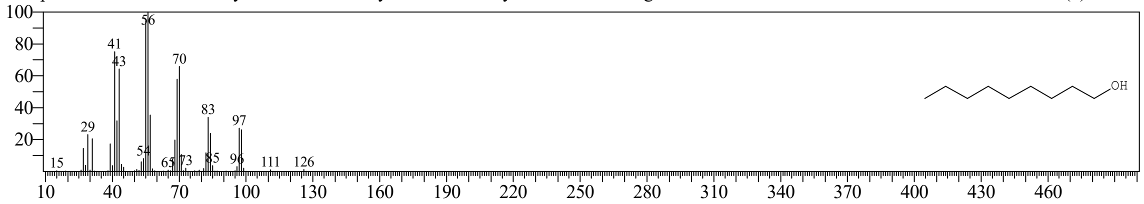

<< Target >>
Line#:2   R.Time:8.377(Scan#:1164) MassPeaks:259
RawMode:Averaged 8.373-8.380(1163-1165) BasePeak:57.05(397204)
BG Mode:Calc. from Peak Group 1 - Event 1 Q3 Scan

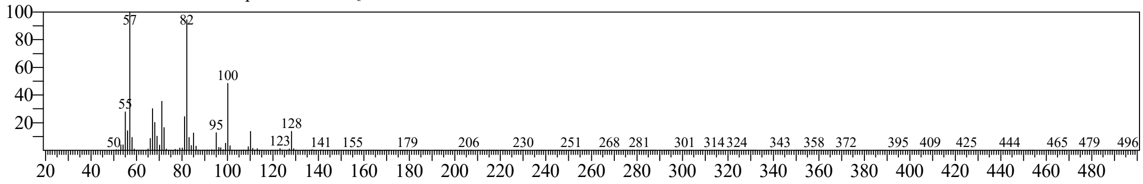

Hit#:1 Entry:6249 Library:NIST17s.lib
SI:86 Formula:C8H16O CAS:696-71-9 MolWeight:128 RetIndex:1147
CompName:Cyclooctyl alcohol $$ Cyclooctanol $$

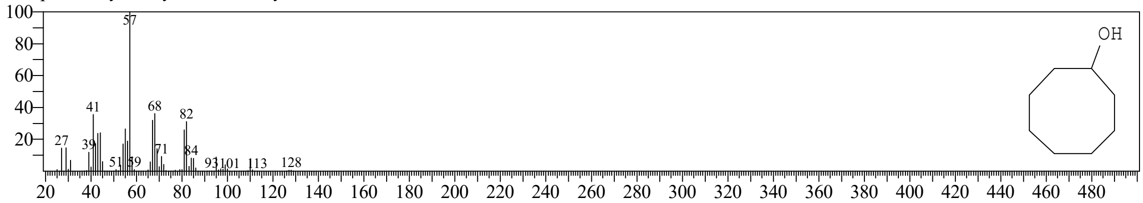

<< Target >>
Line#:3 R.Time:8.887(Scan#:1317) MassPeaks:321
RawMode:Averaged 8.883-8.890(1316-1318) BasePeak:111.05(3537015)
BG Mode:Calc. from Peak Group 1 - Event 1 Q3 Scan

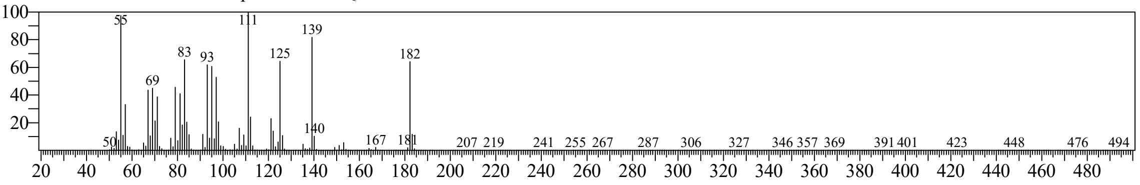

Hit#:1 Entry:18037 Library:NIST17s.lib
SI:95 Formula:C12H22O CAS:13019-16-4 MolWeight:182 RetIndex:1388
CompName:2-Octenal, 2-butyl- $$ 2-Butyl-2-octenal $$ 2-n-Butyloct-2-enal $$

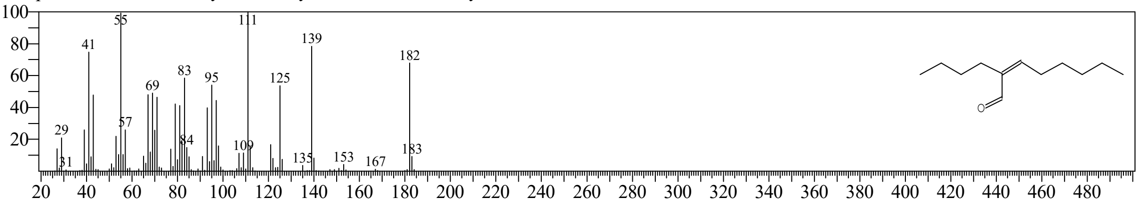

<< Target >>
Line#:4 R.Time:9.260(Scan#:1429) MassPeaks:262
RawMode:Averaged 9.257-9.263(1428-1430) BasePeak:57.05(175567)
BG Mode:Calc. from Peak Group 1 - Event 1 Q3 Scan

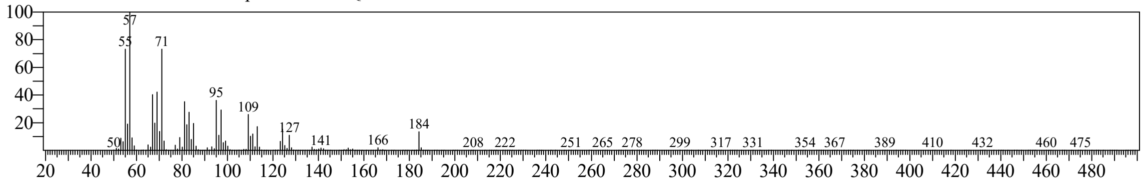

Hit#:1 Entry:189193 Library:NIST17-1.lib
SI:87 Formula:C18H35BrO CAS:56599-95-2 MolWeight:346 RetIndex:2231
CompName:Octadecanal, 2-bromo- $$ 2-Bromooctadecanal # $$

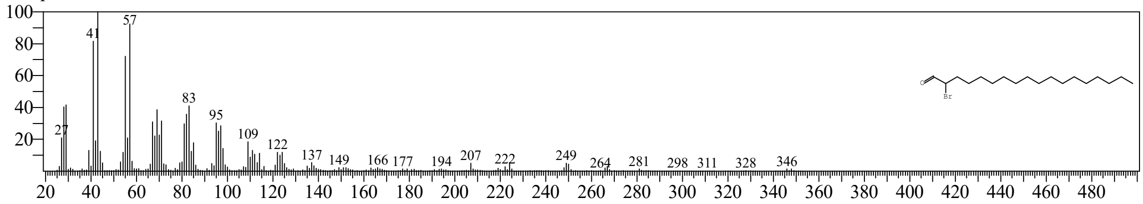

<< Target >>
Line#:5   R.Time:10.127(Scan#:1689) MassPeaks:270
RawMode:Averaged 10.123-10.130(1688-1690) BasePeak:55.05(171291)
BG Mode:Calc. from Peak Group 1 - Event 1 Q3 Scan

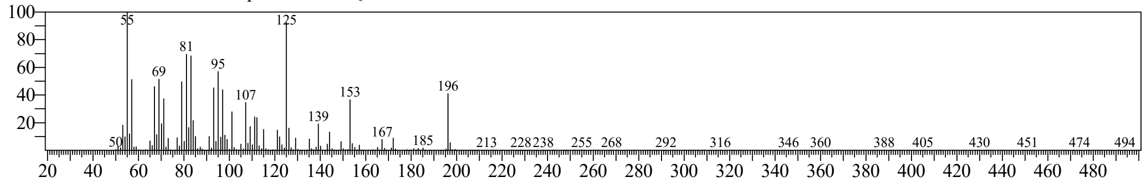

Hit#:1 Entry:58137 Library:NIST17-1.lib
SI:83 Formula:C14H26O CAS:3021-89-4 MolWeight:210 RetIndex:1586
CompName:2-Nonenal, 2-pentyl- $$ 2-Amylnon-2-enal $$ 2-Pentyl-2-nonenal $$ 2-Pentylnon-2-enal $$

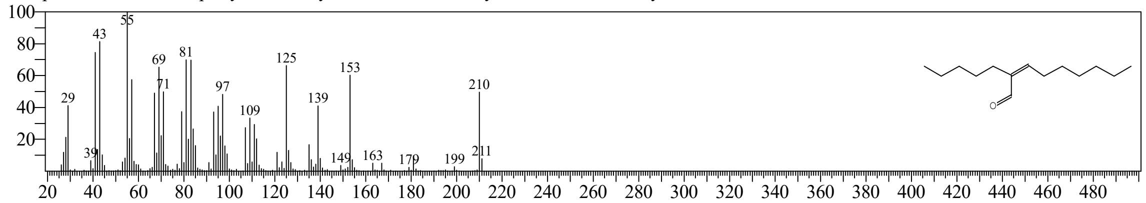

<< Target >>
Line#:6 R.Time:10.183(Scan#:1706) MassPeaks:283
RawMode:Averaged 10.180-10.187(1705-1707) BasePeak:55.05(174721)
BG Mode:Calc. from Peak Group 1 - Event 1 Q3 Scan

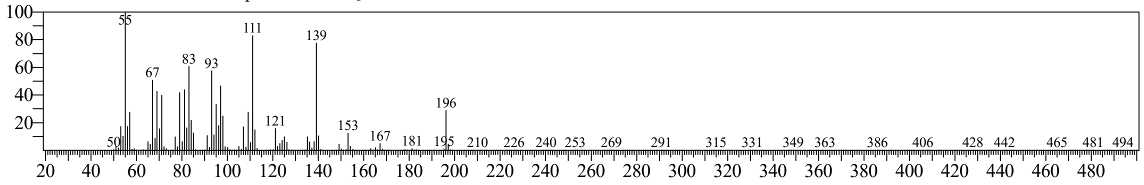

Hit#:1 Entry:18037 Library:NIST17s.lib
SI:88 Formula:C12H22O CAS:13019-16-4 MolWeight:182 RetIndex:1388
CompName:2-Octenal, 2-butyl- $$ 2-Butyl-2-octenal $$ 2-n-Butyloct-2-enal $$

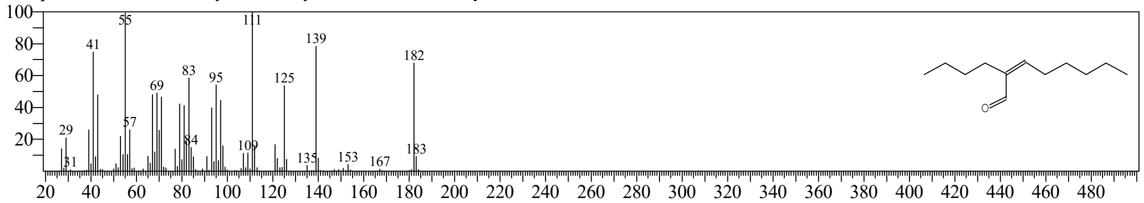

<< Target >>
Line#:7 R.Time:11.343(Scan#:2054) MassPeaks:286
RawMode:Averaged 11.340-11.347(2053-2055) BasePeak:95.05(75800)
BG Mode:Calc. from Peak Group 1 - Event 1 Q3 Scan

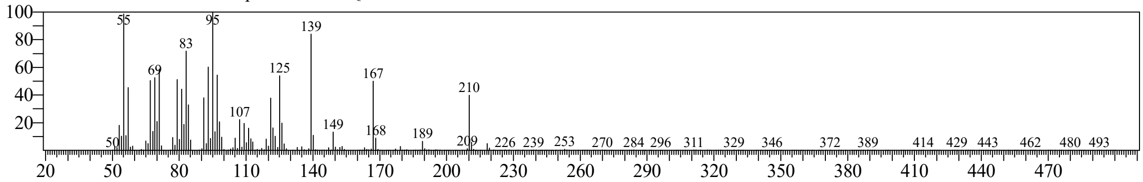

Hit#:1 Entry:18037 Library:NIST17s.lib
SI:82 Formula:C12H22O CAS:13019-16-4 MolWeight:182 RetIndex:1388
CompName:2-Octenal, 2-butyl- $$ 2-Butyl-2-octenal $$ 2-n-Butyloct-2-enal $$

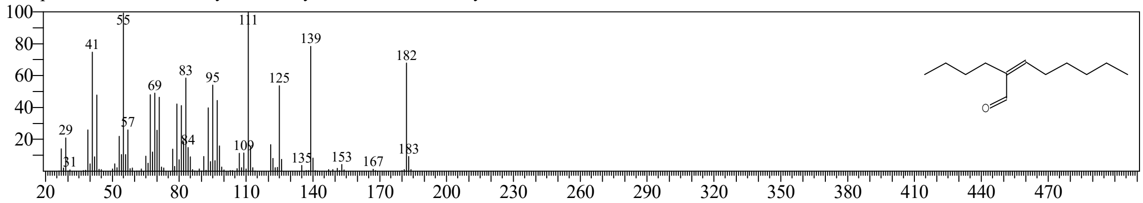

<< Target >>
Line#:8 R.Time:11.390(Scan#:2068) MassPeaks:258
RawMode:Averaged 11.387-11.393(2067-2069) BasePeak:55.05(124400)
BG Mode:Calc. from Peak Group 1 - Event 1 Q3 Scan

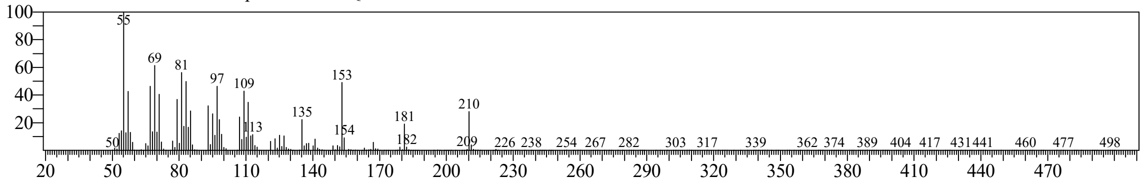

Hit#:1 Entry:58137 Library:NIST17-1.lib
SI:87 Formula:C14H26O CAS:3021-89-4 MolWeight:210 RetIndex:1586
CompName:2-Nonenal, 2-pentyl- $$ 2-Amylnon-2-enal $$ 2-Pentyl-2-nonenal $$ 2-Pentylnon-2-enal $$

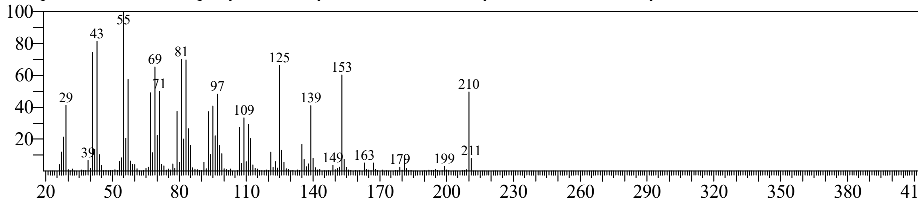

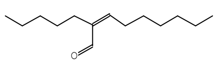

<< Target >>
Line#:9 R.Time:12.163(Scan#:2300) MassPeaks:262
RawMode:Averaged 12.160-12.167(2299-2301) BasePeak:137.10(549474)
BG Mode:Calc. from Peak Group 1 - Event 1 Q3 Scan

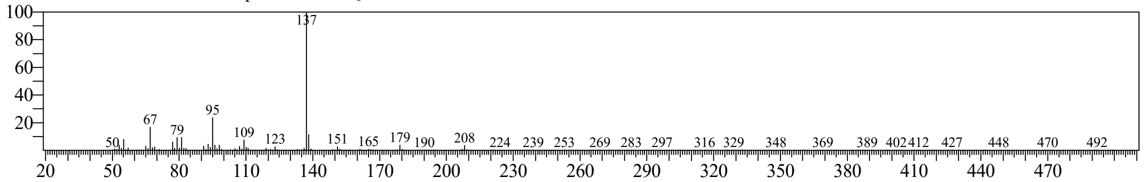

Hit#:1 Entry:17653 Library:NIST17-1.lib
SI:86 Formula:C10H16O CAS:81250-41-1 MolWeight:152 RetIndex:0
CompName:4,5-Heptadien-2-one, 3,3,6-trimethyl- $$ 3,3,6-Trimethyl-4,5-heptadien-2-one # $$

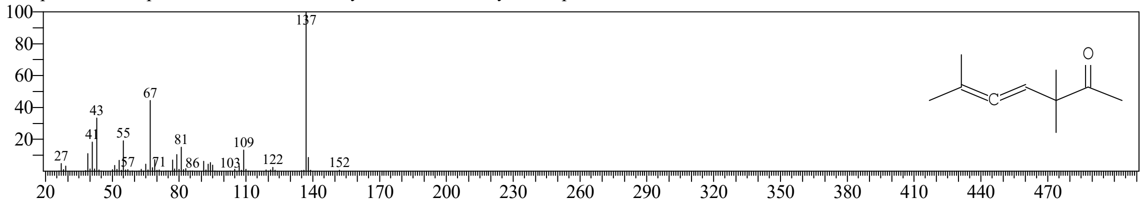

<< Target >>
Line#:10 R.Time:12.227(Scan#:2319) MassPeaks:283
RawMode:Averaged 12.223-12.230(2318-2320) BasePeak:82.05(350740)
BG Mode:Calc. from Peak Group 1 - Event 1 Q3 Scan

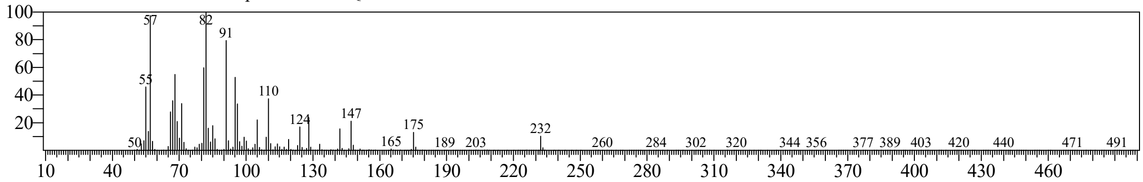

Hit#:1 Entry:13077 Library:NIST17-1.lib
SI:79 Formula:C9H18O CAS:90676-25-8 MolWeight:142 RetIndex:1167
CompName:2-Propylcyclohexanol

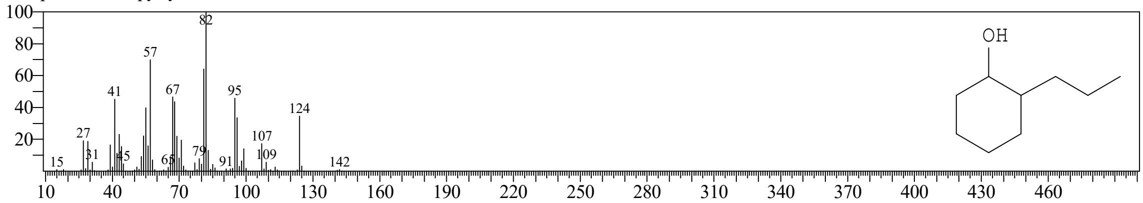

<< Target >>
Line#:11   R.Time:12.287(Scan#:2337) MassPeaks:257
RawMode:Averaged 12.283-12.290(2336-2338) BasePeak:82.05(570653)
BG Mode:Calc. from Peak Group 1 - Event 1 Q3 Scan

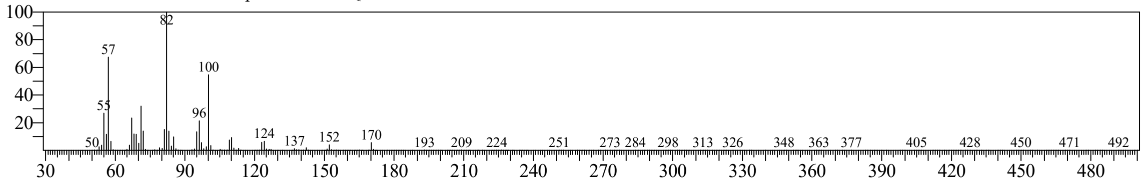

Hit#:1 Entry:9184 Library:NIST17s.lib
SI:82 Formula:C9H18O CAS:90676-25-8 MolWeight:142 RetIndex:1167
CompName:2-Propylcyclohexanol

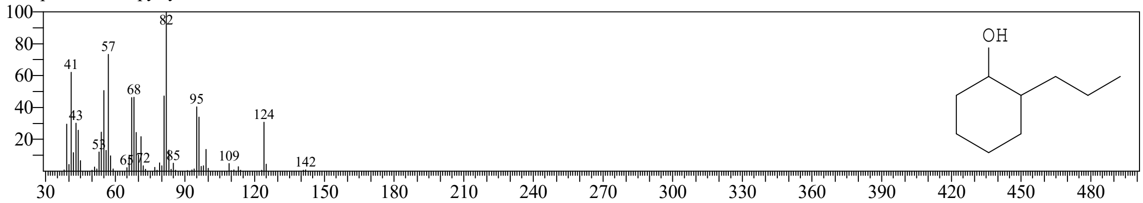

<< Target >>
Line#:12 R.Time:12.617(Scan#:2436) MassPeaks:299
RawMode:Averaged 12.613-12.620(2435-2437) BasePeak:55.05(2528485)
BG Mode:Calc. from Peak Group 1 - Event 1 Q3 Scan

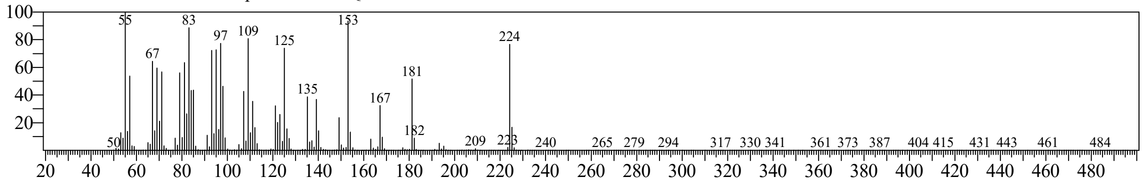

Hit#:1 Entry:58137 Library:NIST17-1.lib
SI:82 Formula:C14H26O CAS:3021-89-4 MolWeight:210 RetIndex:1586
CompName:2-Nonenal, 2-pentyl- $$ 2-Amylnon-2-enal $$ 2-Pentyl-2-nonenal $$ 2-Pentylnon-2-enal $$

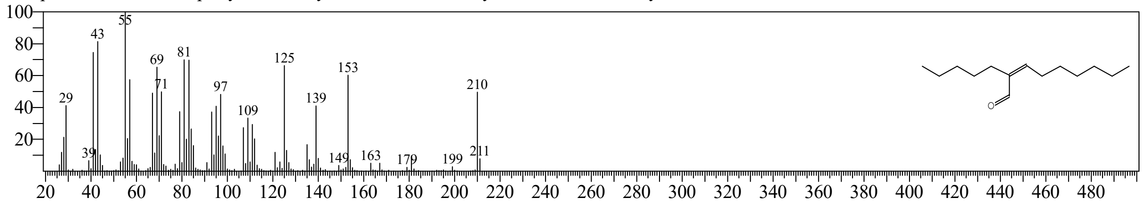

<< Target >>
Line#:13   R.Time:12.743(Scan#:2474) MassPeaks:309
RawMode:Averaged 12.740-12.747(2473-2475) BasePeak:111.05(3899879)
BG Mode:Calc. from Peak Group 1 - Event 1 Q3 Scan

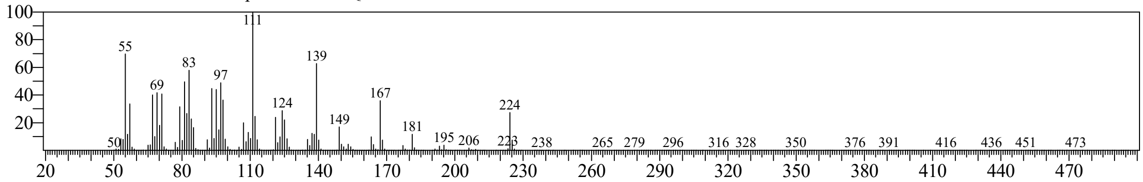

Hit#:1 Entry:18037 Library:NIST17s.lib
SI:81 Formula:C12H22O CAS:13019-16-4 MolWeight:182 RetIndex:1388
CompName:2-Octenal, 2-butyl- $$ 2-Butyl-2-octenal $$ 2-n-Butyloct-2-enal $$

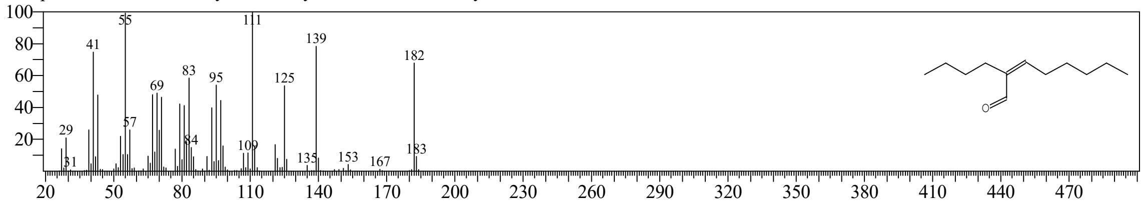

<< Target >>
Line#:14   R.Time:12.893(Scan#:2519) MassPeaks:235
RawMode:Averaged 12.890-12.897(2518-2520) BasePeak:57.05(120143)
BG Mode:Calc. from Peak Group 1 - Event 1 Q3 Scan

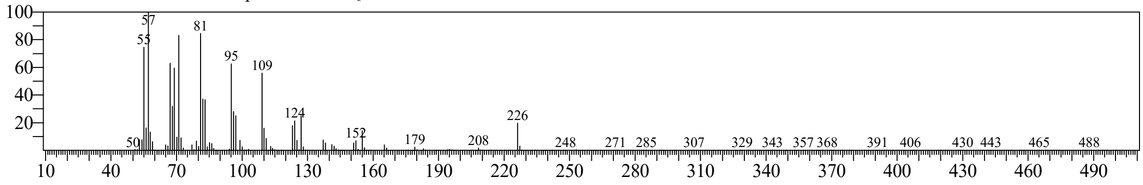

Hit#:1 Entry:111649 Library:NIST17-1.lib
SI:85 Formula:C18H36O CAS:7390-81-0 MolWeight:268 RetIndex:1901
CompName:Oxirane, hexadecyl- $$ 1,2-Epoxyoctadecane $$ 2-Hexadecyloxirane # $$

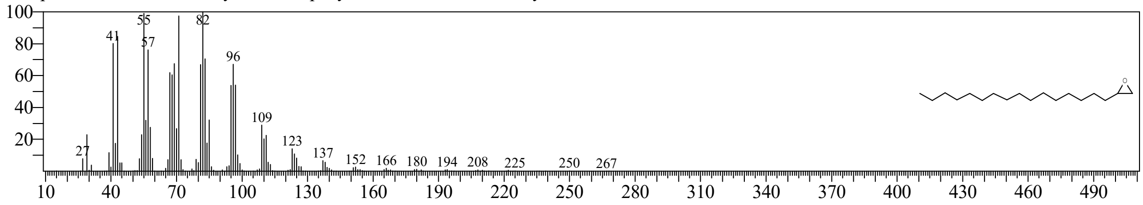

<< Target >>
Line#:15 R.Time:13.017(Scan#:2556) MassPeaks:260
RawMode:Averaged 13.013-13.020(2555-2557) BasePeak:57.05(152774)
BG Mode:Calc. from Peak Group 1 - Event 1 Q3 Scan

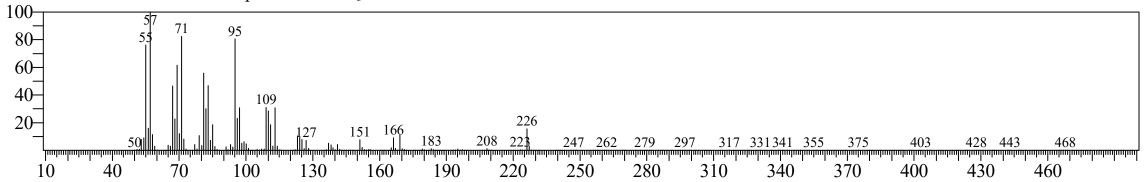

Hit#:1 Entry:111649 Library:NIST17-1.lib
SI:86 Formula:C18H36O CAS:7390-81-0 MolWeight:268 RetIndex:1901
CompName:Oxirane, hexadecyl- $$ 1,2-Epoxyoctadecane $$ 2-Hexadecyloxirane # $$

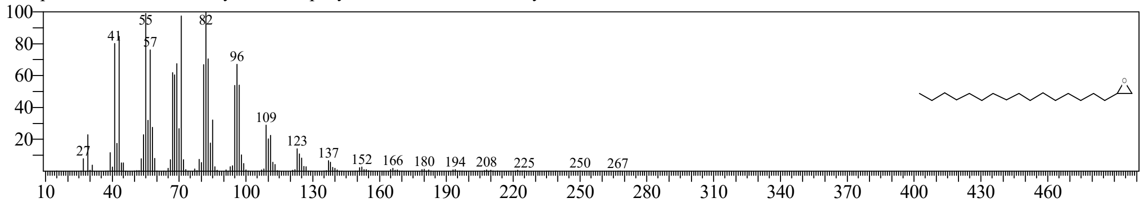

<< Target >>
Line#:16 R.Time:13.423(Scan#:2678) MassPeaks:276
RawMode:Averaged 13.420-13.427(2677-2679) BasePeak:137.10(627253)
BG Mode:Calc. from Peak Group 1 - Event 1 Q3 Scan

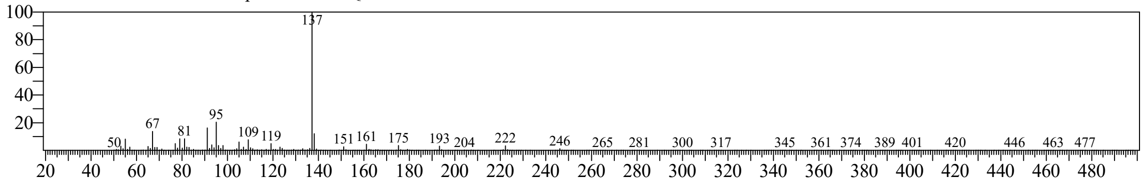

Hit#:1 Entry:17655 Library:NIST17-1.lib
SI:83 Formula:C10H16O CAS:74410-10-9 MolWeight:152 RetIndex:1103
CompName:Dill ether $$ (3S,3aS,7aR)-3,6-Dimethyl-2,3,3a,4,5,7a-hexahydrobenzofuran $$ Benzofuran, 2,3,3a,4,5,7a-hexahydro-3,6-dimethyl-, (3S,3aS,7

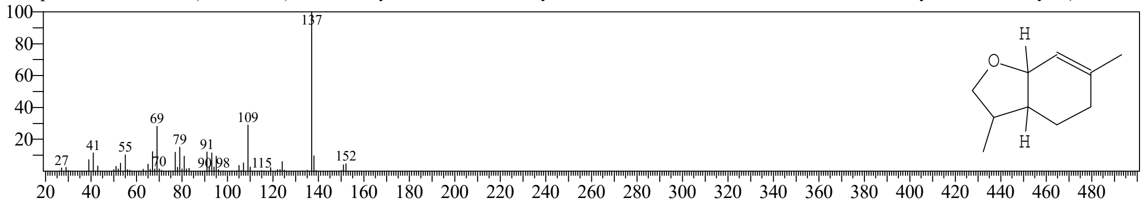

<< Target >>
Line#:17 R.Time:13.813(Scan#:2795) MassPeaks:257
RawMode:Averaged 13.810-13.817(2794-2796) BasePeak:55.05(155605)
BG Mode:Calc. from Peak Group 1 - Event 1 Q3 Scan

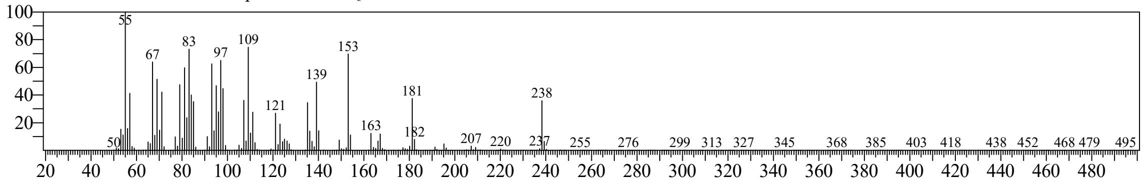

Hit#:1 Entry:58137 Library:NIST17-1.lib
SI:83 Formula:C14H26O CAS:3021-89-4 MolWeight:210 RetIndex:1586
CompName:2-Nonenal, 2-pentyl- $$ 2-Amylnon-2-enal $$ 2-Pentyl-2-nonenal $$ 2-Pentylnon-2-enal $$

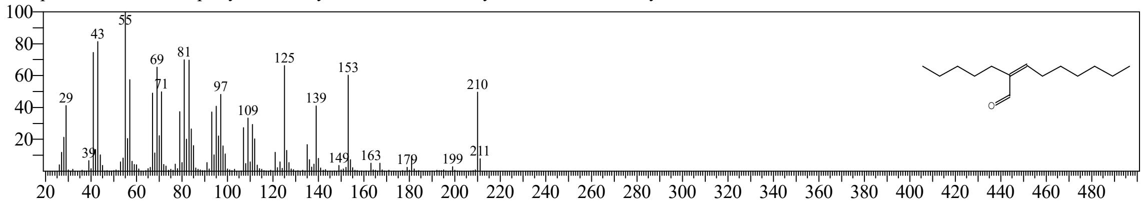

<< Target >>
Line#:18   R.Time:13.877(Scan#:2814) MassPeaks:289
RawMode:Averaged 13.873-13.880(2813-2815) BasePeak:55.05(161052)
BG Mode:Calc. from Peak Group 1 - Event 1 Q3 Scan

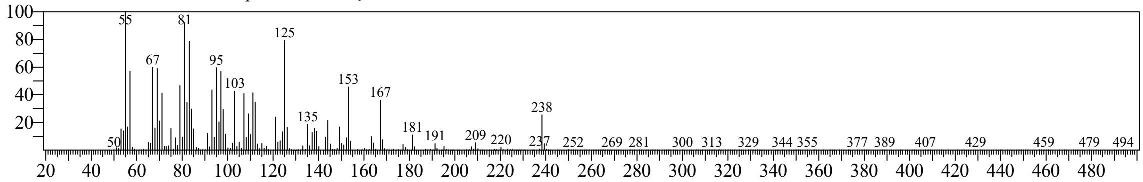

Hit#:1 Entry:58137 Library:NIST17-1.lib
SI:82 Formula:C14H26O CAS:3021-89-4 MolWeight:210 RetIndex:1586
CompName:2-Nonenal, 2-pentyl- $$ 2-Amylnon-2-enal $$ 2-Pentyl-2-nonenal $$ 2-Pentylnon-2-enal $$

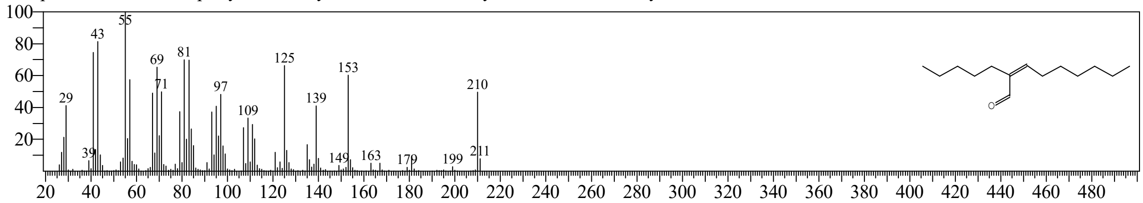

<< Target >>
Line#:19 R.Time:14.530(Scan#:3010) MassPeaks:312
RawMode:Averaged 14.527-14.533(3009-3011) BasePeak:193.15(277349)
BG Mode:Calc. from Peak Group 1 - Event 1 Q3 Scan

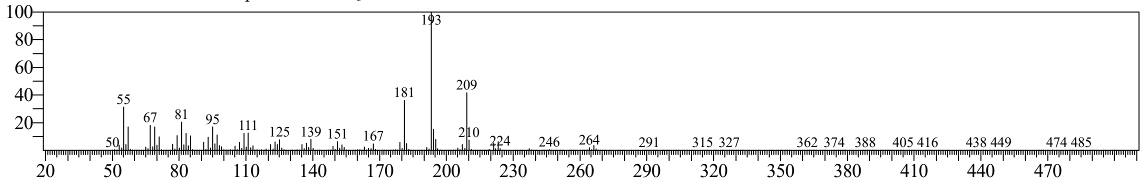

Hit#:1 Entry:68784 Library:NIST17-1.lib
SI:76 Formula:C15H26O CAS:313253-65-5 MolWeight:222 RetIndex:1628
CompName:2-Pentanone, 4-cyclohexylidene-3,3-diethyl- $$ 4-Cyclohexylidene-3,3-diethyl-2-pentanone # $$

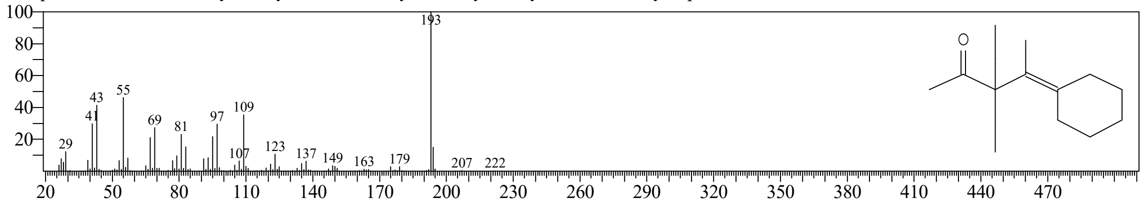

<< Target >>
Line#:20 R.Time:15.090(Scan#:3178) MassPeaks:300
RawMode:Averaged 15.087-15.093(3177-3179) BasePeak:55.05(140469)
BG Mode:Calc. from Peak Group 1 - Event 1 Q3 Scan

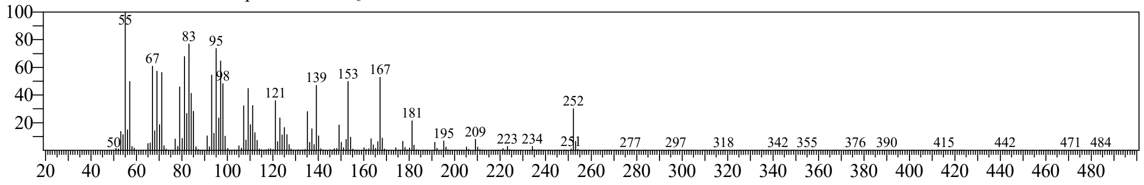

Hit#:1 Entry:58137 Library:NIST17-1.lib
SI:84 Formula:C14H26O CAS:3021-89-4 MolWeight:210 RetIndex:1586
CompName:2-Nonenal, 2-pentyl- $$ 2-Amylnon-2-enal $$ 2-Pentyl-2-nonenal $$ 2-Pentylnon-2-enal $$

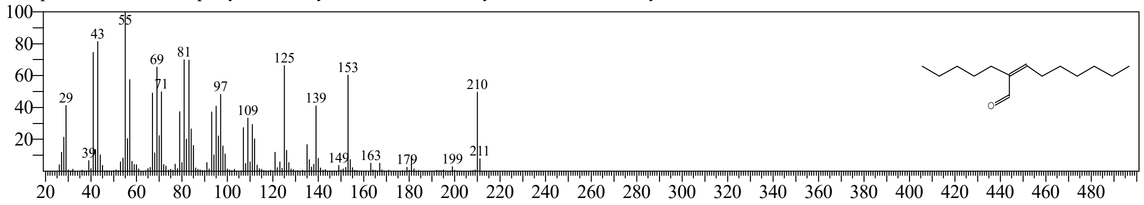

<< Target >>
Line#:21   R.Time:15.143(Scan#:3194) MassPeaks:302
RawMode:Averaged 15.140-15.147(3193-3195) BasePeak:193.15(688020)
BG Mode:Calc. from Peak Group 1 - Event 1 Q3 Scan

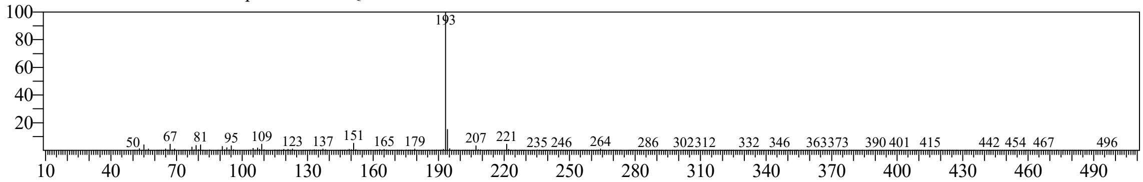

Hit#:1 Entry:158810 Library:NIST17-1.lib
SI:80 Formula:C16H21N5S CAS:22362-19-2 MolWeight:315 RetIndex:2836
CompName:2-Amino-4-benzylthiomethyl-6-piperidino-1,3,5-triazine $$ 4-[(Benzylsulfanyl)methyl]-6-(1-piperidinyl)-1,3,5-triazin-2-ylamine # $$

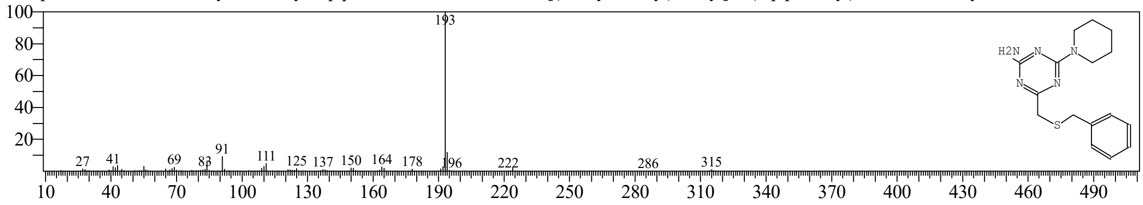

<< Target >>
Line#:22 R.Time:15.807(Scan#:3393) MassPeaks:244
RawMode:Averaged 15.803-15.810(3392-3394) BasePeak:179.10(515745)
BG Mode:Calc. from Peak Group 1 - Event 1 Q3 Scan

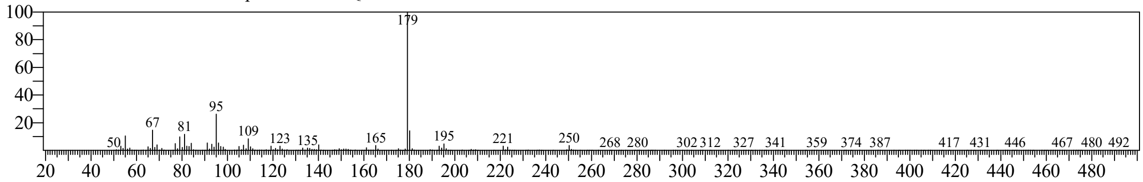

Hit#:1 Entry:20394 Library:NIST17s.lib
SI:79 Formula:C13H22O CAS:41678-32-4 MolWeight:194 RetIndex:1342
CompName:2H-1-Benzopyran, 3,4,4a,5,6,8a-hexahydro-2,5,5,8a-tetramethyl-, (2.alpha.,4a.alpha.,8a.alpha.)- $$ (2.alpha.,4a.alpha.,8a.alpha.)-3,4,4a,5,6,8a-I

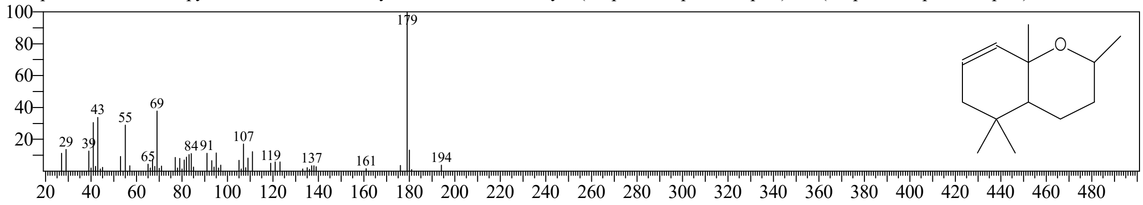

<< Target >>
Line#:23   R.Time:16.043(Scan#:3464) MassPeaks:271
RawMode:Averaged 16.040-16.047(3463-3465) BasePeak:82.05(478609)
BG Mode:Calc. from Peak Group 1 - Event 1 Q3 Scan

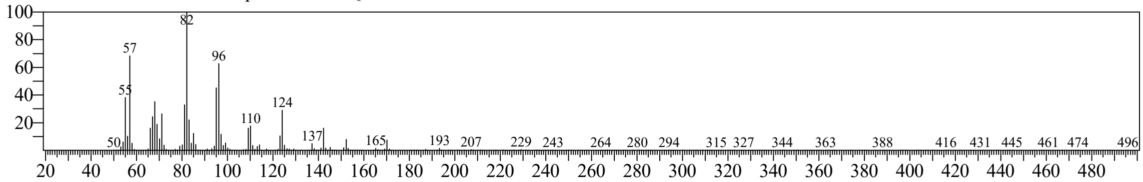

Hit#:1 Entry:25974 Library:NIST17s.lib
SI:87 Formula:C15H30O CAS:2765-11-9 MolWeight:226 RetIndex:1701
CompName:Pentadecanal- $$ 1-Pentadecanal $$ n-Pentadecanal $$

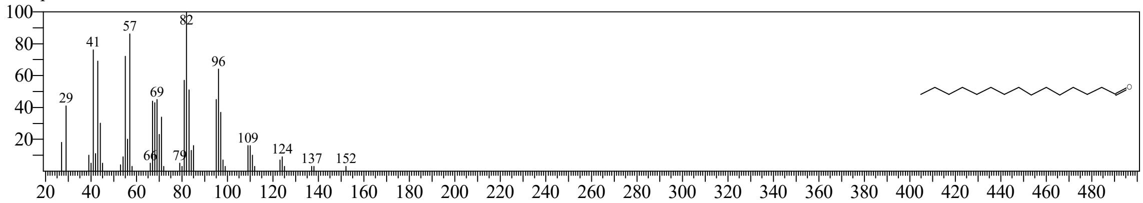

<< Target >>
Line#:24   R.Time:16.283(Scan#:3536) MassPeaks:285
RawMode:Averaged 16.280-16.287(3535-3537) BasePeak:73.00(214876)
BG Mode:Calc. from Peak Group 1 - Event 1 Q3 Scan

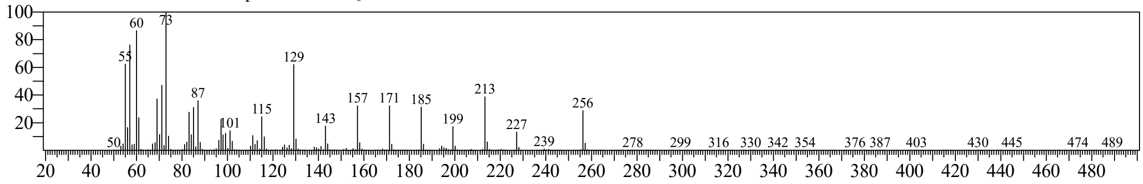

Hit#:1 Entry:29350 Library:NIST17s.lib
SI:93 Formula:C16H32O2 CAS:57-10-3 MolWeight:256 RetIndex:1968
CompName:n-Hexadecanoic acid $$ Hexadecanoic acid $$ n-Hexadecoic acid $$ Palmitic acid $$ Pentadecanecarboxylic acid $$ 1-Pentadecanecarboxylic

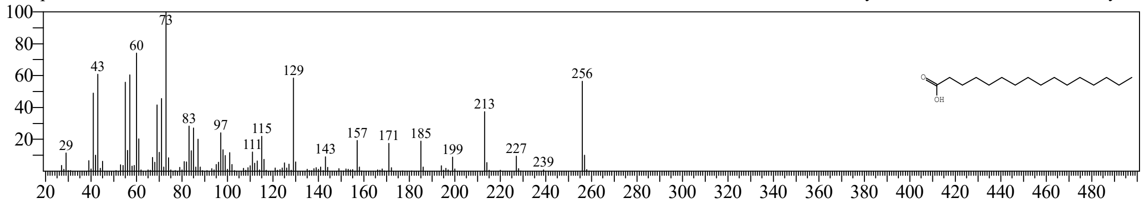

<< Target >>
Line#:25   R.Time:16.383(Scan#:3566) MassPeaks:310
RawMode:Averaged 16.380-16.387(3565-3567) BasePeak:153.10(2246682)
BG Mode:Calc. from Peak Group 1 - Event 1 Q3 Scan

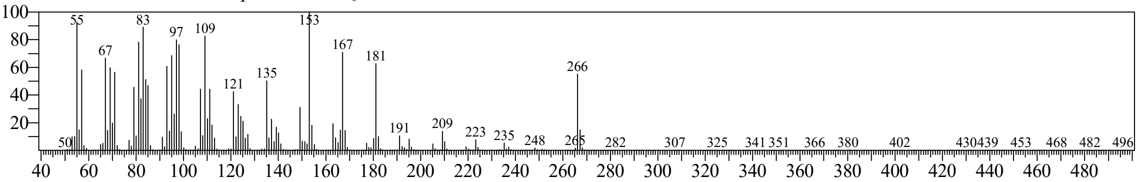

Hit#:1 Entry:109302 Library:NIST17-1.lib
SI:77 Formula:C16H26O3 CAS:59426-46-9 MolWeight:266 RetIndex:2161
CompName:2,5-Furandione, 3-dodecyl- $$ 3-Dodecyl-2,5-furandione # $$

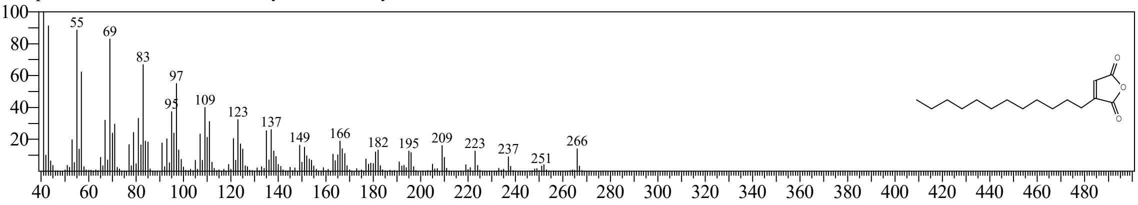

<< Target >>
Line#:26 R.Time:16.580(Scan#:3625) MassPeaks:278
RawMode:Averaged 16.577-16.583(3624-3626) BasePeak:181.15(39108)
BG Mode:Calc. from Peak Group 1 - Event 1 Q3 Scan

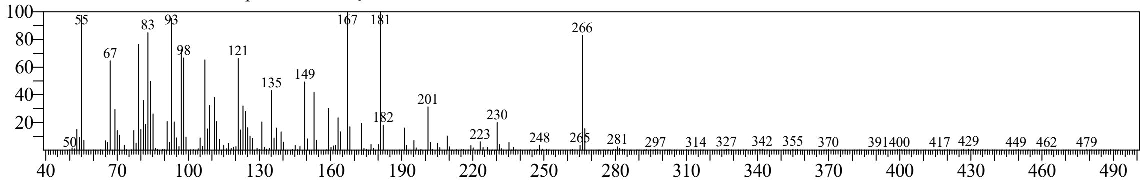

Hit#:1 Entry:109302 Library:NIST17-1.lib
SI:68 Formula:C16H26O3 CAS:59426-46-9 MolWeight:266 RetIndex:2161
CompName:2,5-Furandione, 3-dodecyl- $$ 3-Dodecyl-2,5-furandione # $$

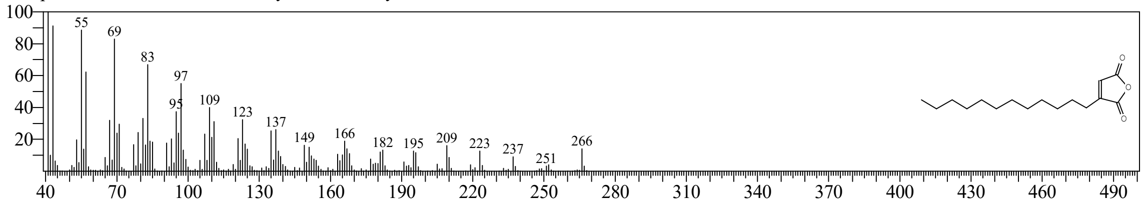

<< Target >>
Line#:27 R.Time:16.623(Scan#:3638) MassPeaks:232
RawMode:Averaged 16.620-16.627(3637-3639) BasePeak:57.05(83111)
BG Mode:Calc. from Peak Group 1 - Event 1 Q3 Scan

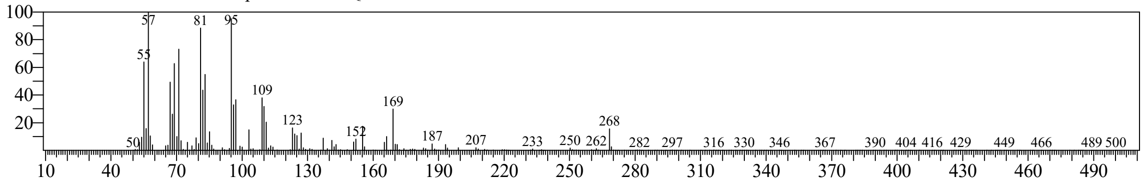

Hit#:1 Entry:111649 Library:NIST17-1.lib
SI:82 Formula:C18H36O CAS:7390-81-0 MolWeight:268 RetIndex:1901
CompName:Oxirane, hexadecyl- $$ 1,2-Epoxyoctadecane $$ 2-Hexadecyloxirane # $$

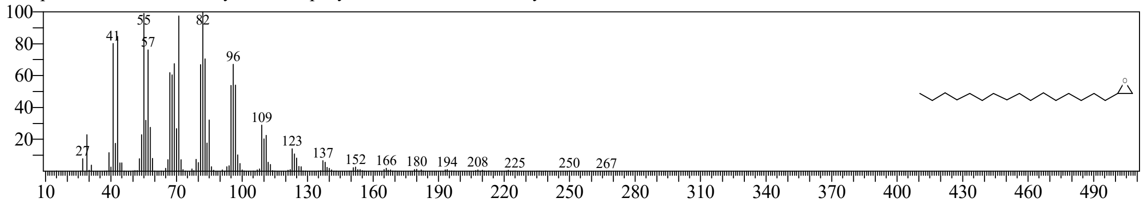

<< Target >>
Line#:28 R.Time:16.910(Scan#:3724) MassPeaks:326
RawMode:Averaged 16.907-16.913(3723-3725) BasePeak:57.10(349754)
BG Mode:Calc. from Peak Group 1 - Event 1 Q3 Scan

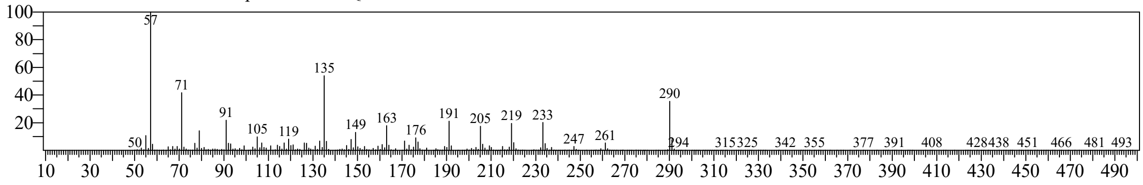

Hit#:1 Entry:65438 Library:NIST17-1.lib
SI:61 Formula:C14H21NO CAS:0-00-0 MolWeight:219 RetIndex:1847
CompName:2,4,5-Trimethylaniline, N-trimethylacetyl-

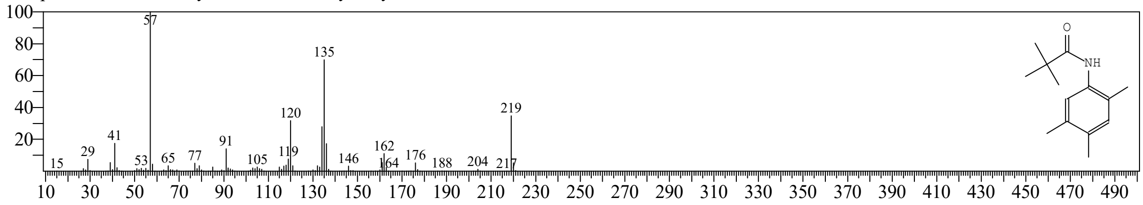

<< Target >>
Line#:29   R.Time:17.057(Scan#:3768) MassPeaks:306
RawMode:Averaged 17.053-17.060(3767-3769) BasePeak:179.10(610648)
BG Mode:Calc. from Peak Group 1 - Event 1 Q3 Scan

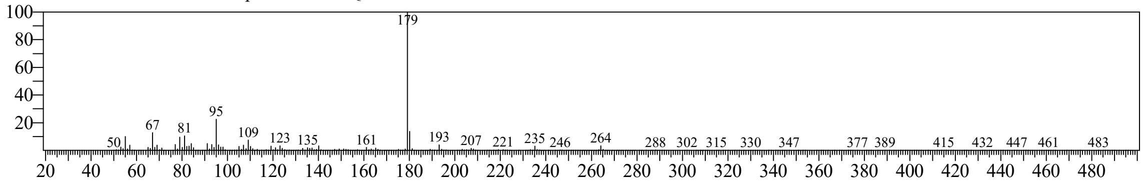

Hit#:1 Entry:20394 Library:NIST17s.lib
SI:81 Formula:C13H22O CAS:41678-32-4 MolWeight:194 RetIndex:1342
CompName:2H-1-Benzopyran, 3,4,4a,5,6,8a-hexahydro-2,5,5,8a-tetramethyl-, (2.alpha.,4a.alpha.,8a.alpha.)- $$ (2.alpha.,4a.alpha.,8a.alpha.)-3,4,4a,5,6,8a-I

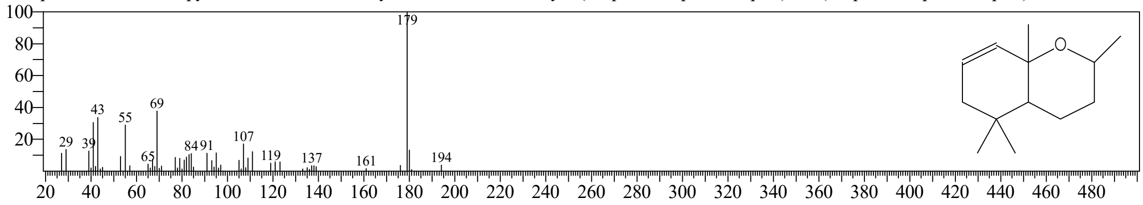

<< Target >>
Line#:30 R.Time:17.953(Scan#:4037) MassPeaks:316
RawMode:Averaged 17.950-17.957(4036-4038) BasePeak:235.20(165064)
BG Mode:Calc. from Peak Group 1 - Event 1 Q3 Scan

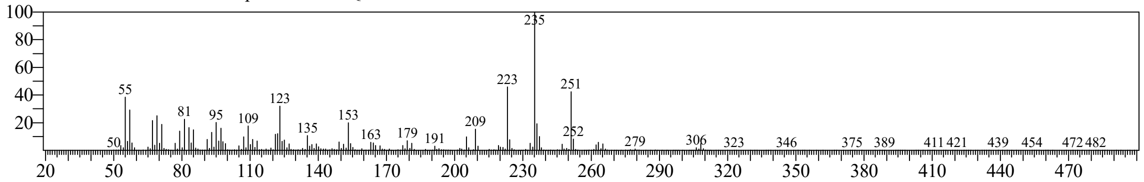

Hit#:1 Entry:107064 Library:NIST17-1.lib
SI:65 Formula:C13H29ClOSi CAS:0-00-0 MolWeight:264 RetIndex:1476
CompName:Silane, chlorodiethylnonyloxy-

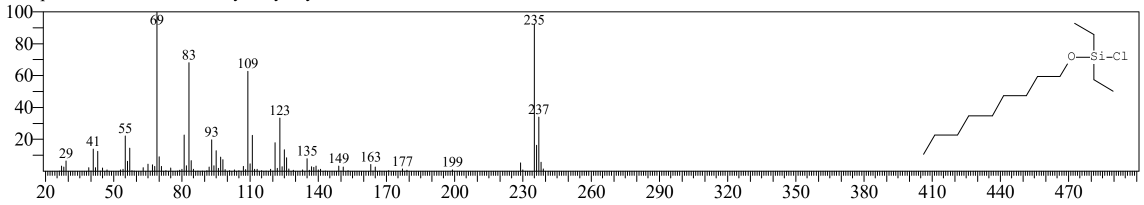

<< Target >>
Line#:31   R.Time:18.023(Scan#:4058) MassPeaks:232
RawMode:Averaged 18.020-18.027(4057-4059) BasePeak:223.20(98097)
BG Mode:Calc. from Peak Group 1 - Event 1 Q3 Scan

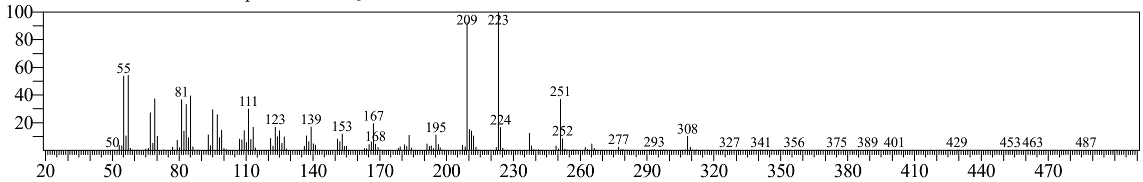

Hit#:1 Entry:151972 Library:NIST17-1.lib
SI:67 Formula:C20H36O2 CAS:15583-49-0 MolWeight:308 RetIndex:2582
CompName:Cyclohexadecadiene-1,6-dione, 3,4-diethyl-, cis- $$ 3,4-Diethyl-1,6-cyclohexadecanedione # $$

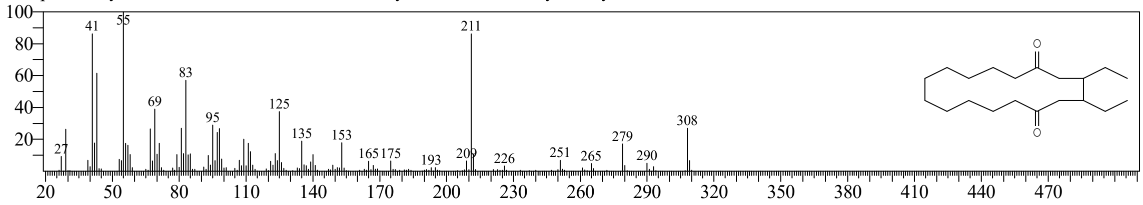

<< Target >>
Line#:32 R.Time:18.070(Scan#:4072) MassPeaks:270
RawMode:Averaged 18.067-18.073(4071-4073) BasePeak:71.10(269639)
BG Mode:Calc. from Peak Group 1 - Event 1 Q3 Scan

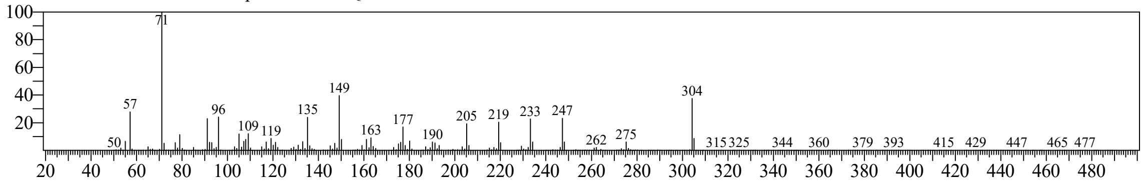

Hit#:1 Entry:66534 Library:NIST17-1.lib
SI:57 Formula:C14H20O2 CAS:71596-88-8 MolWeight:220 RetIndex:1632
CompName:Ethanone, 1-(5,6,7,8-tetrahydro-2,8,8-trimethyl-4H-cyclohepta[b]furan-5-yl)- $$ 1-(2,8,8-Trimethyl-5,6,7,8-tetrahydro-4H-cyclohepta[b]furan-5

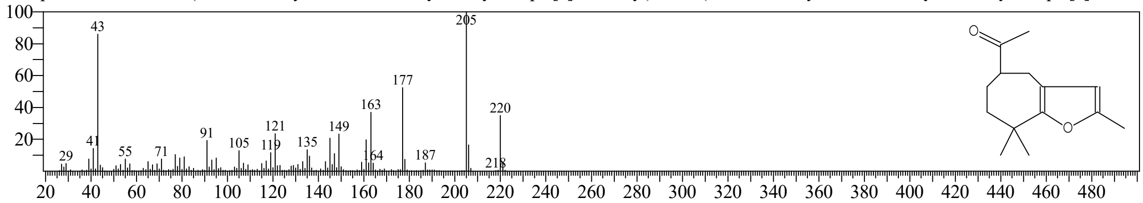

<< Target >>
Line#:33   R.Time:18.510(Scan#:4204) MassPeaks:298
RawMode:Averaged 18.507-18.513(4203-4205) BasePeak:55.05(158513)
BG Mode:Calc. from Peak Group 1 - Event 1 Q3 Scan

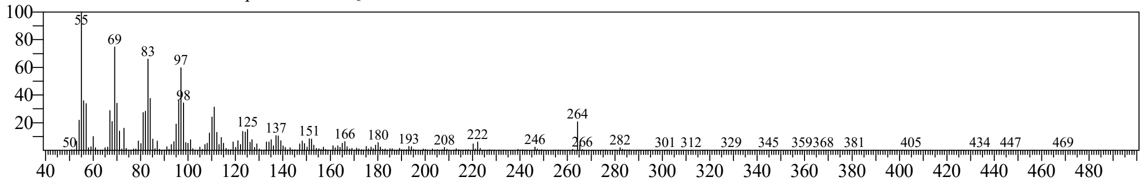

Hit#:1 Entry:31601 Library:NIST17s.lib
SI:94 Formula:C18H34O2 CAS:112-79-8 MolWeight:282 RetIndex:2175
CompName:9-Octadecenoic acid, (E)- $$ trans-.delta.(sup 9)-Octadecenoic acid $$ trans-.delta.9-Octadecenoic acid $$ trans-Octadec-9-enoic acid $$ trans-

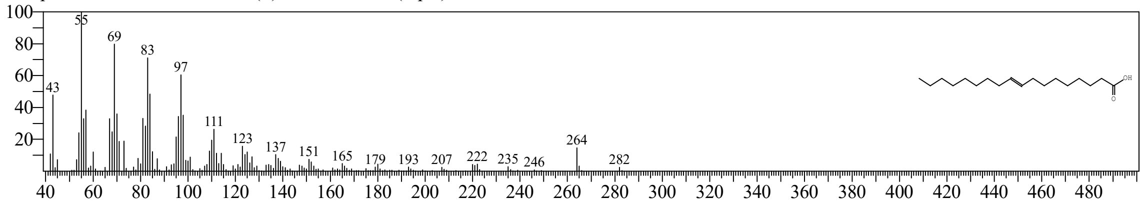

<< Target >>
Line#:34 R.Time:18.560(Scan#:4219) MassPeaks:245
RawMode:Averaged 18.557-18.563(4218-4220) BasePeak:235.20(1073178)
BG Mode:Calc. from Peak Group 1 - Event 1 Q3 Scan

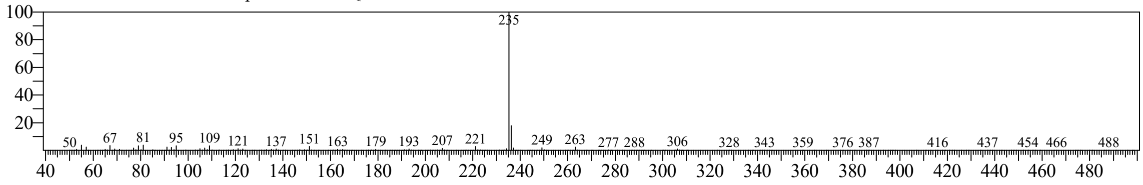

Hit#:1 Entry:255267 Library:NIST17-1.lib
SI:82 Formula:C28H38O7 CAS:5366-08-5 MolWeight:486 RetIndex:3603
CompName:Benzoic acid, 4-[(2,4-dimethoxy-6-pentylbenzoyl)oxy]-2-methoxy-6-pentyl-, methyl ester $$ o-Anisic acid, 4-hydroxy-6-pentyl-, methyl ester, 2

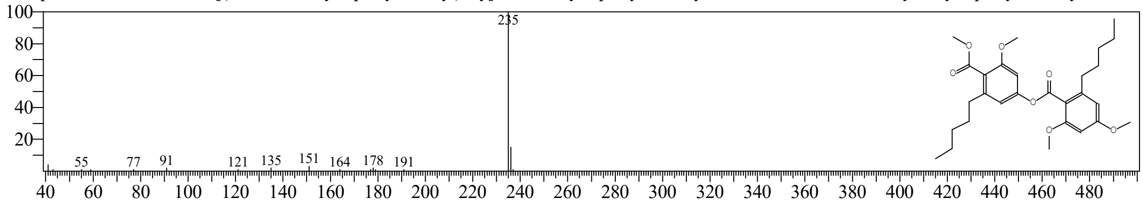

<< Target >>
Line#:35   R.Time:18.780(Scan#:4285) MassPeaks:321
RawMode:Averaged 18.777-18.783(4284-4286) BasePeak:193.15(696291)
BG Mode:Calc. from Peak Group 1 - Event 1 Q3 Scan

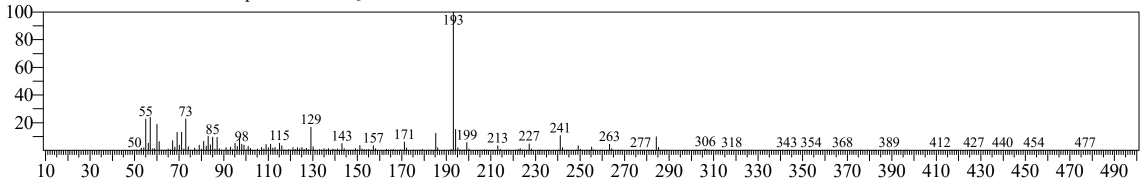

Hit#:1 Entry:31773 Library:NIST17s.lib
SI:66 Formula:C18H36O2 CAS:57-11-4 MolWeight:284 RetIndex:2167
CompName:Octadecanoic acid $$ Stearic acid $$ n-Octadecanoic acid $$ Humko Industrene R $$ Hydrofol Acid 150 $$ Hystrene S-97 $$ Hystrene T-70 $

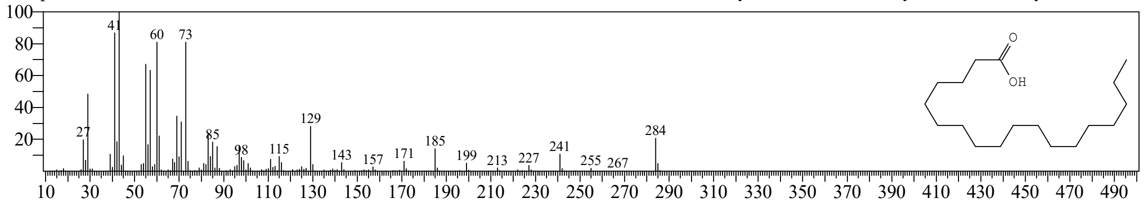

<< Target >>
Line#:36 R.Time:20.370(Scan#:4762) MassPeaks:309
RawMode:Averaged 20.367-20.373(4761-4763) BasePeak:57.10(588167)
BG Mode:Calc. from Peak Group 1 - Event 1 Q3 Scan

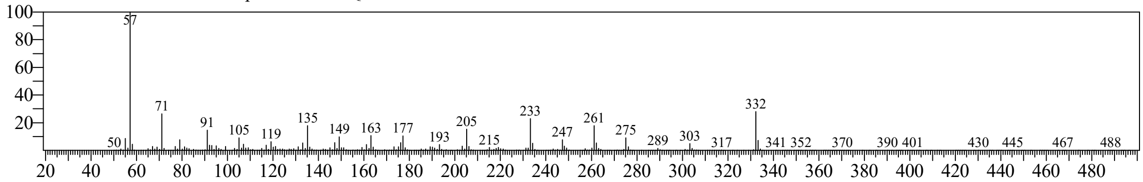

Hit#:1 Entry:176347 Library:NIST17-1.lib
SI:65 Formula:C22H36O2 CAS:0-00-0 MolWeight:332 RetIndex:2346
CompName:2H-1-benzopyran-6-ol, 3,4-dihydro-2,2-dimethyl-4-(1-methylethyl)-7-(1,1,3,3-tetramethylbutyl)- $$ 2,2-dimethyl-4-(propan-2-yl)-7-(2,4,4-trime

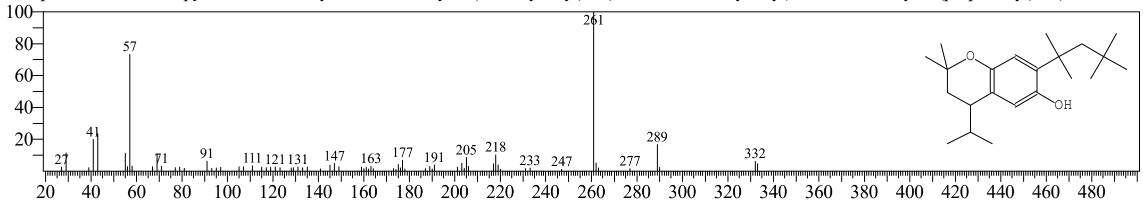

<< Target >>
Line#:37 R.Time:20.453(Scan#:4787) MassPeaks:312
RawMode:Averaged 20.450-20.457(4786-4788) BasePeak:57.05(343040)
BG Mode:Calc. from Peak Group 1 - Event 1 Q3 Scan

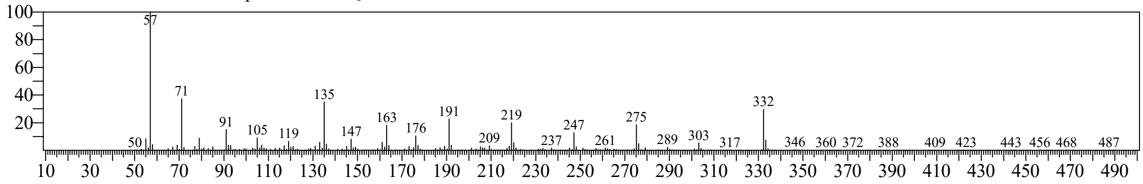

Hit#:1 Entry:65438 Library:NIST17-1.lib
SI:63 Formula:C14H21NO CAS:0-00-0 MolWeight:219 RetIndex:1847
CompName:2,4,5-Trimethylaniline, N-trimethylacetyl-

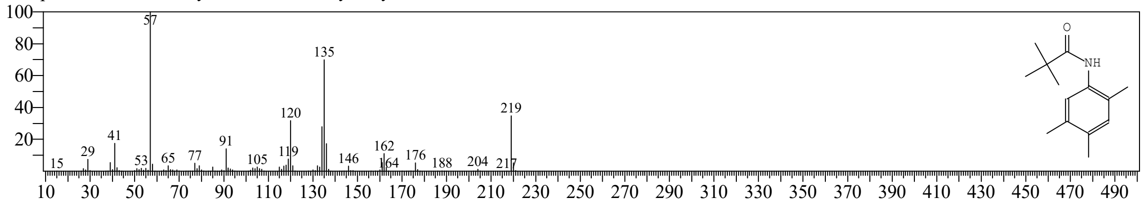

<< Target >>
Line#:38 R.Time:21.430(Scan#:5080) MassPeaks:344
RawMode:Averaged 21.427-21.433(5079-5081) BasePeak:235.20(249575)
BG Mode:Calc. from Peak Group 1 - Event 1 Q3 Scan

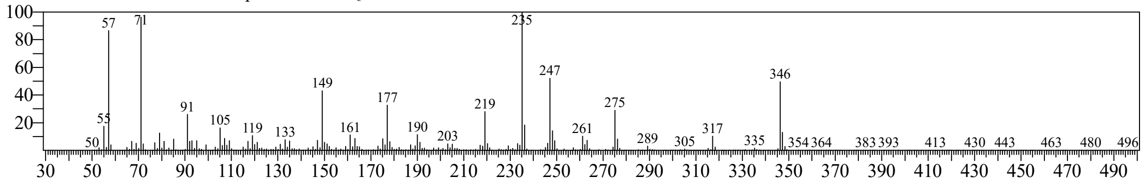

Hit#:1 Entry:188956 Library:NIST17-1.lib
SI:56 Formula:C16H27I CAS:132156-64-0 MolWeight:346 RetIndex:1666
CompName:9-t-Butyl-4-iodo-2,2-dimethyladamantane $$ 4-tert-Butyl-9-iodo-2,2-dimethyladamantane # $$

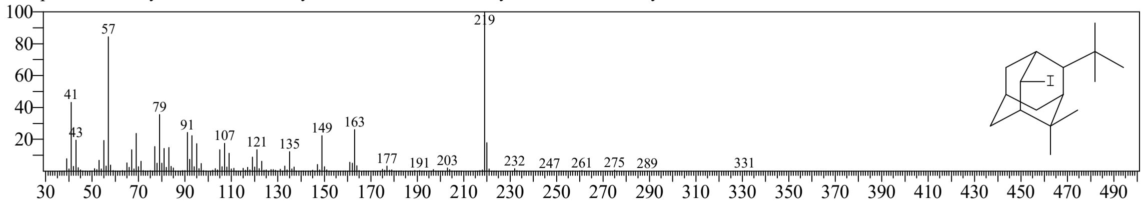

<< Target >>
Line#:39   R.Time:21.517(Scan#:5106) MassPeaks:277
RawMode:Averaged 21.513-21.520(5105-5107) BasePeak:71.10(252446)
BG Mode:Calc. from Peak Group 1 - Event 1 Q3 Scan

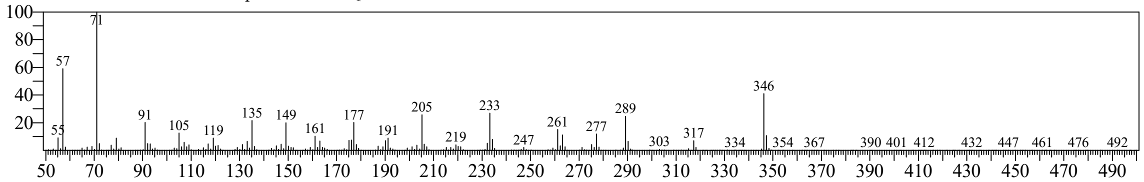

Hit#:1 Entry:119555 Library:NIST17-1.lib
SI:58 Formula:C17H24O3 CAS:82304-66-3 MolWeight:276 RetIndex:2081
CompName:7,9-Di-tert-butyl-1-oxaspiro(4,5)deca-6,9-diene-2,8-dione $$ 1-Oxa-spiro[4.5]deca-6,9-diene-2,8-dione, 7,9-di-tert-butyl- $$ 7,9-Di-tert-butyl-1

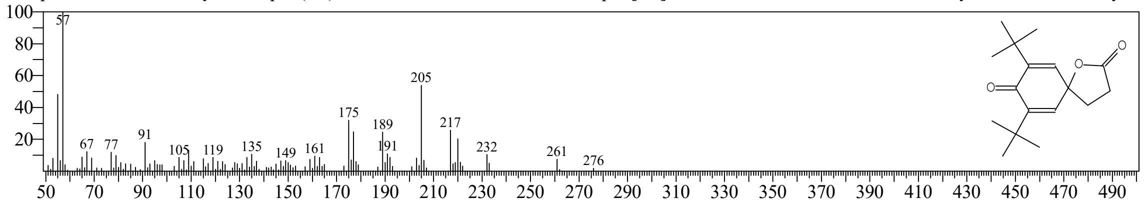

<< Target >>
Line#:40 R.Time:21.910(Scan#:5224) MassPeaks:261
RawMode:Averaged 21.907-21.913(5223-5225) BasePeak:235.20(852089)
BG Mode:Calc. from Peak Group 1 - Event 1 Q3 Scan

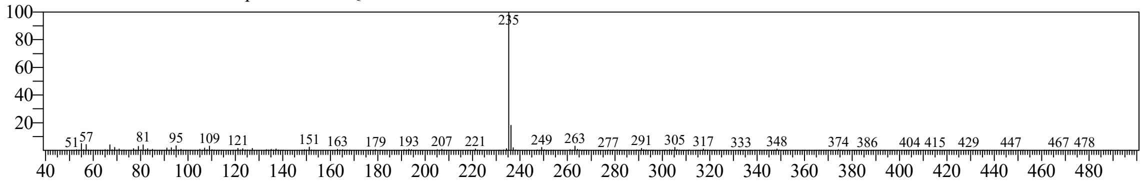

Hit#:1 Entry:255267 Library:NIST17-1.lib
SI:80 Formula:C28H38O7 CAS:5366-08-5 MolWeight:486 RetIndex:3603
CompName:Benzoic acid, 4-[(2,4-dimethoxy-6-pentylbenzoyl)oxy]-2-methoxy-6-pentyl-, methyl ester $$ o-Anisic acid, 4-hydroxy-6-pentyl-, methyl ester, 2

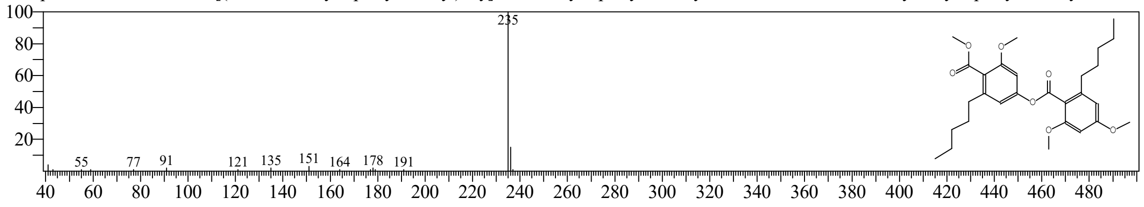

<< Target >>
Line#:41 R.Time:23.517(Scan#:5706) MassPeaks:347
RawMode:Averaged 23.513-23.520(5705-5707) BasePeak:57.10(278644)
BG Mode:Calc. from Peak Group 1 - Event 1 Q3 Scan

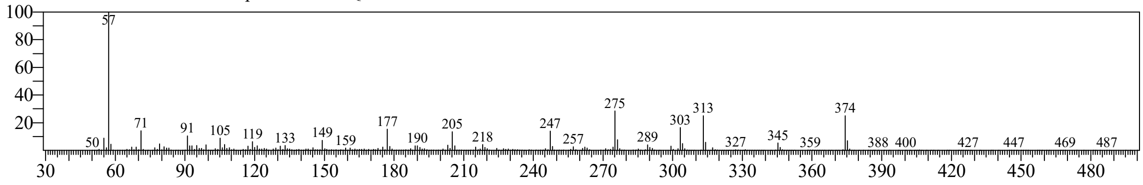

Hit#:1 Entry:31113 Library:NIST17s.lib
SI:56 Formula:C17H24O3 CAS:82304-66-3 MolWeight:276 RetIndex:2081
CompName:7,9-Di-tert-butyl-1-oxaspiro(4,5)deca-6,9-diene-2,8-dione $$ 1-Oxa-spiro[4.5]deca-6,9-diene-2,8-dione, 7,9-di-tert-butyl- $$ 7,9-Di-tert-butyl-1

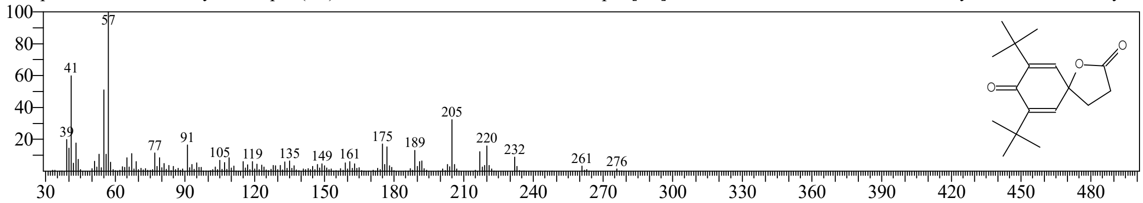

<< Target >>
Line#:42   R.Time:23.593(Scan#:5729) MassPeaks:341
RawMode:Averaged 23.590-23.597(5728-5730) BasePeak:57.10(450411)
BG Mode:Calc. from Peak Group 1 - Event 1 Q3 Scan

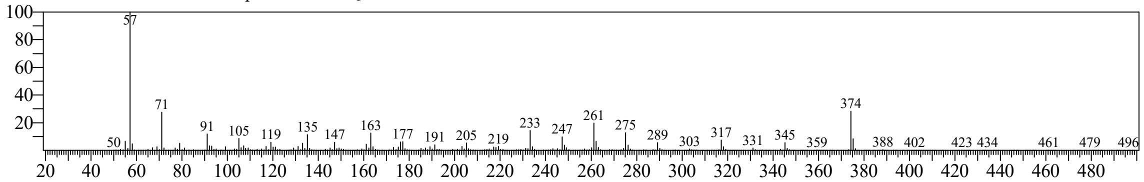

Hit#:1 Entry:176347 Library:NIST17-1.lib
SI:63 Formula:C22H36O2 CAS:0-00-0 MolWeight:332 RetIndex:2346
CompName:2H-1-benzopyran-6-ol, 3,4-dihydro-2,2-dimethyl-4-(1-methylethyl)-7-(1,1,3,3-tetramethylbutyl)- $$ 2,2-dimethyl-4-(propan-2-yl)-7-(2,4,4-trime

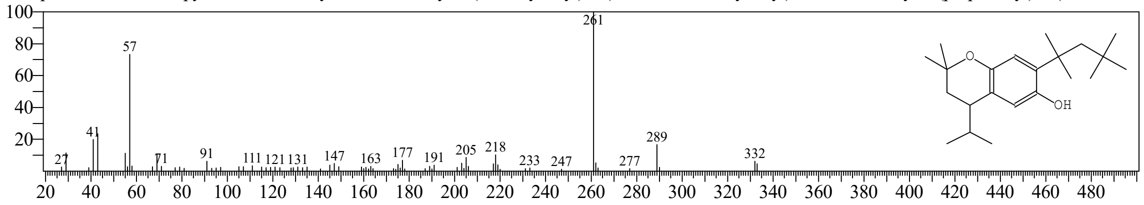

<< Target >>
Line#:43   R.Time:24.280(Scan#:5935) MassPeaks:325
RawMode:Averaged 24.277-24.283(5934-5936) BasePeak:277.20(139482)
BG Mode:Calc. from Peak Group 1 - Event 1 Q3 Scan

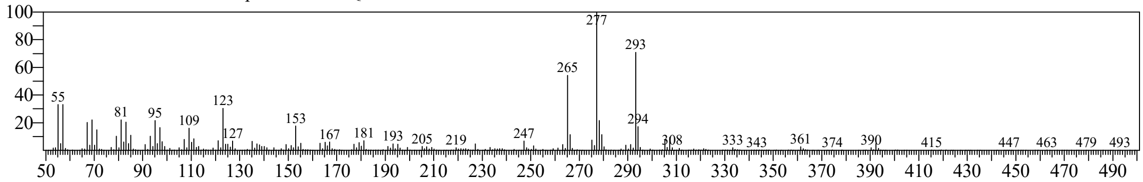

Hit#:1 Entry:181798 Library:NIST17-1.lib
SI:62 Formula:C22H42O2 CAS:0-00-0 MolWeight:338 RetIndex:2275
CompName:i-Propyl 11,12-methylene-octadecanoate

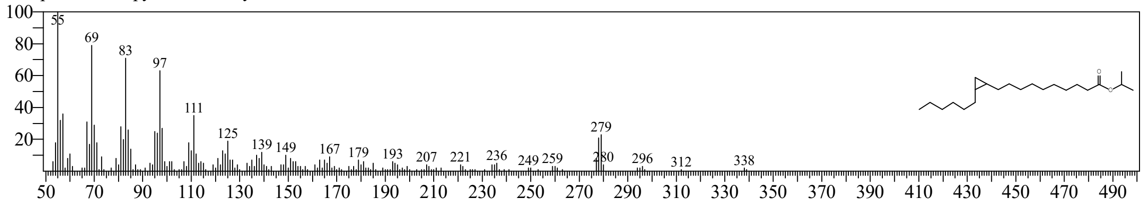

<< Target >>
Line#:44 R.Time:24.540(Scan#:6013) MassPeaks:348
RawMode:Averaged 24.537-24.543(6012-6014) BasePeak:71.10(198429)
BG Mode:Calc. from Peak Group 1 - Event 1 Q3 Scan

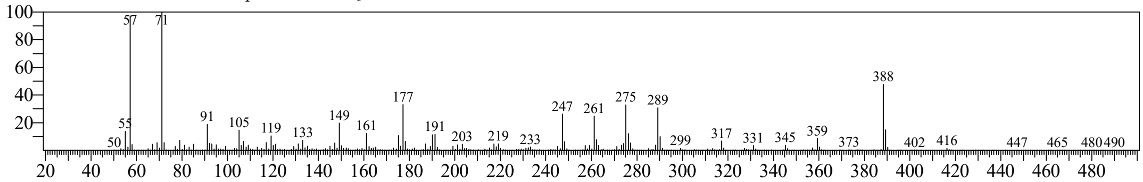

Hit#:1 Entry:176347 Library:NIST17-1.lib
SI:57 Formula:C22H36O2 CAS:0-00-0 MolWeight:332 RetIndex:2346
CompName:2H-1-benzopyran-6-ol, 3,4-dihydro-2,2-dimethyl-4-(1-methylethyl)-7-(1,1,3,3-tetramethylbutyl)- $$ 2,2-dimethyl-4-(propan-2-yl)-7-(2,4,4-trime

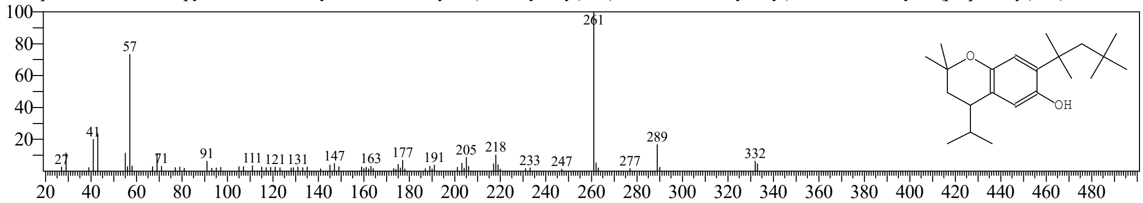

<< Target >>
Line#:45   R.Time:30.167(Scan#:7701) MassPeaks:360
RawMode:Averaged 30.163-30.170(7700-7702) BasePeak:400.30(65790)
BG Mode:Calc. from Peak Group 1 - Event 1 Q3 Scan

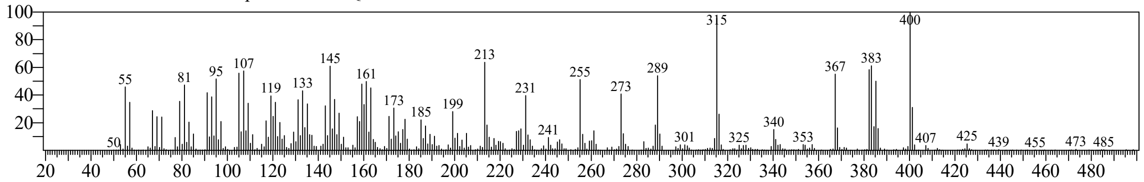

Hit#:1 Entry:37658 Library:NIST17s.lib
SI:88 Formula:C28H48O CAS:474-62-4 MolWeight:400 RetIndex:2632
CompName:Campesterol $$ Ergost-5-en-3-ol, (3.beta.,24R)- $$ Ergost-5-en-3.beta.-ol, (24R)- $$ (24R)-5-Ergosten-3.beta.-ol $$ Campesterin $$ 24.alpha.-

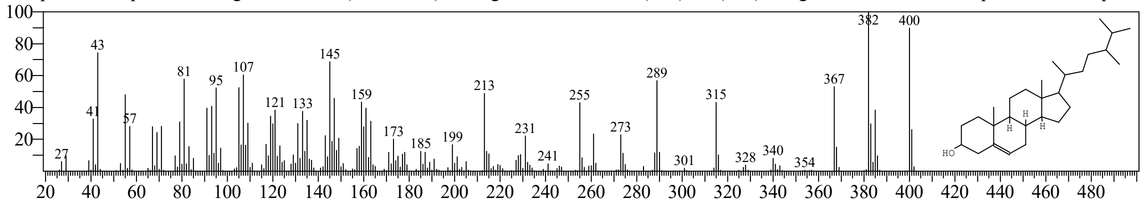

<< Target >>
Line#:46   R.Time:30.447(Scan#:7785) MassPeaks:382
RawMode:Averaged 30.443-30.450(7784-7786) BasePeak:55.05(63963)
BG Mode:Calc. from Peak Group 1 - Event 1 Q3 Scan

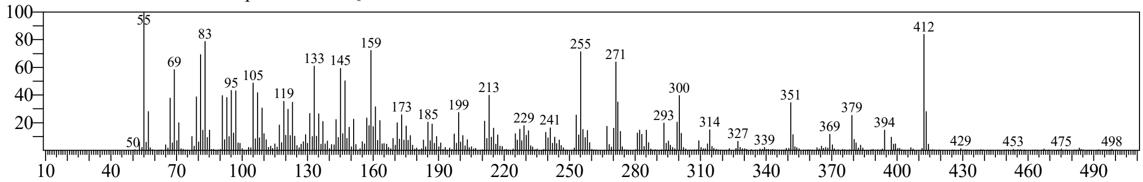

Hit#:1 Entry:37859 Library:NIST17s.lib
SI:83 Formula:C29H48O CAS:83-48-7 MolWeight:412 RetIndex:2739
CompName:Stigmasterol $$ Stigmasta-5,22-dien-3-ol, (3.beta.,22E)- $$ Stigmasta-5,22-dien-3.beta.-ol $$ .beta.-Stigmasterol $$ (24S)-5,22-Stigmastadien-

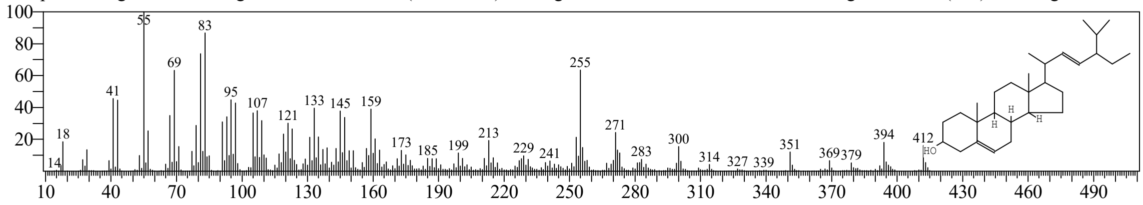

<< Target >>
Line#:47   R.Time:30.970(Scan#:7942) MassPeaks:406
RawMode:Averaged 30.967-30.973(7941-7943) BasePeak:414.35(637425)
BG Mode:Calc. from Peak Group 1 - Event 1 Q3 Scan

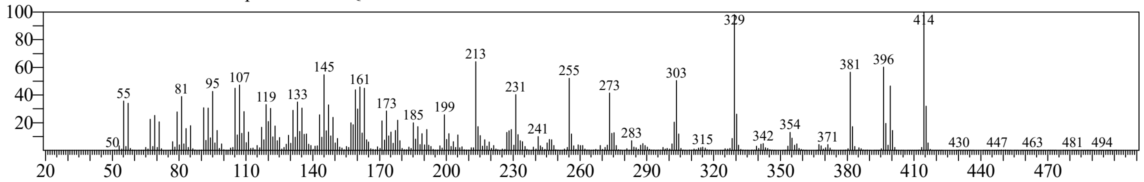

Hit#:1 Entry:37913 Library:NIST17s.lib
SI:89 Formula:C29H50O CAS:83-47-6 MolWeight:414 RetIndex:2731
CompName:.gamma.-Sitosterol $$ Stigmast-5-en-3-ol, (3.beta.,24S)- $$ Stigmast-5-en-3.beta.-ol, (24S)- $$ Clionasterol $$ Fucosterol, .beta.-dihydro- $$ 2[illegible]

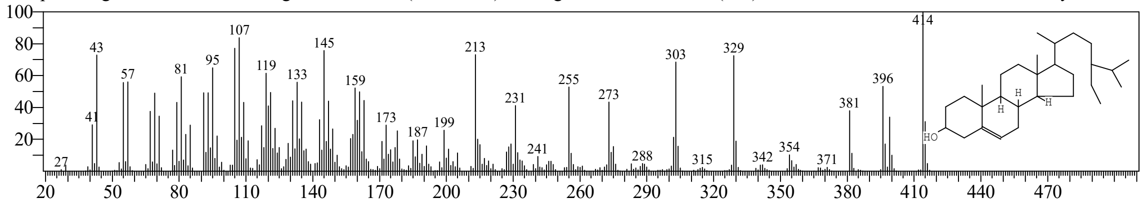

<< Target >>
Line#:48 R.Time:31.457(Scan#:8088) MassPeaks:353
RawMode:Averaged 31.453-31.460(8087-8089) BasePeak:414.35(143818)
BG Mode:Calc. from Peak Group 1 - Event 1 Q3 Scan

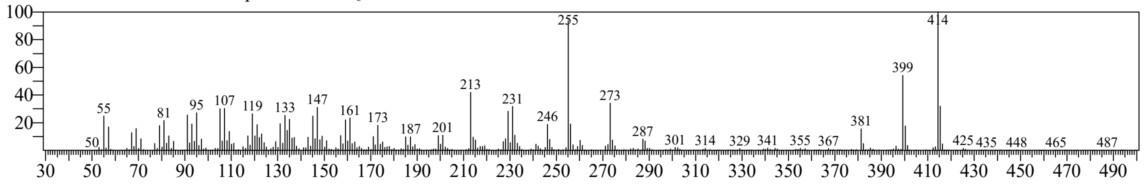

Hit#:1 Entry:234573 Library:NIST17-1.lib
SI:85 Formula:C29H50O CAS:18525-35-4 MolWeight:414 RetIndex:2731
CompName:Stigmast-7-en-3-ol, (3.beta.,5.alpha.,24S)- $$ 5.alpha.-Stigmast-7-en-3.beta.-ol, (24S)- $$ .delta.7-Chondrillastenol $$ Chondrillast-7-enol $$ 2

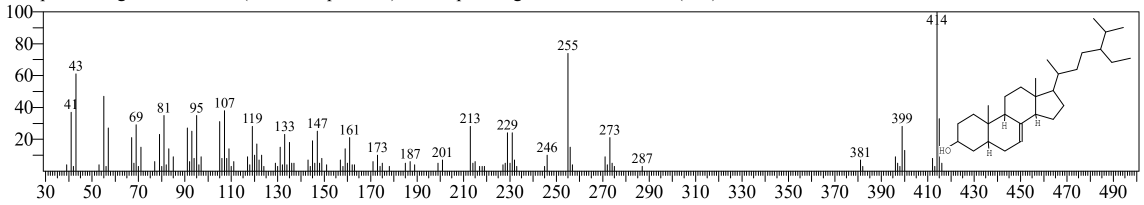

<< Target >>
Line#:49 R.Time:32.143(Scan#:8294) MassPeaks:380
RawMode:Averaged 32.140-32.147(8293-8295) BasePeak:95.05(41461)
BG Mode:Calc. from Peak Group 1 - Event 1 Q3 Scan

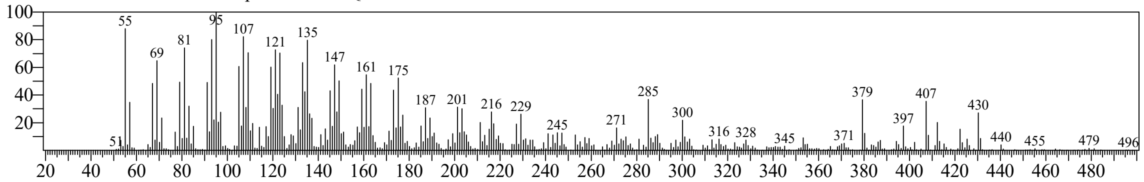

Hit#:1 Entry:244261 Library:NIST17-1.lib
SI:86 Formula:C31H52O CAS:1449-09-8 MolWeight:440 RetIndex:2834
CompName:9,19-Cyclolanostan-3-ol, 24-methylene-, (3.beta.)- $$ 9,19-Cyclo-9.beta.-lanostan-3.beta.-ol, 24-methylene- $$ 24-Methylenecycloartanol $$ 1-

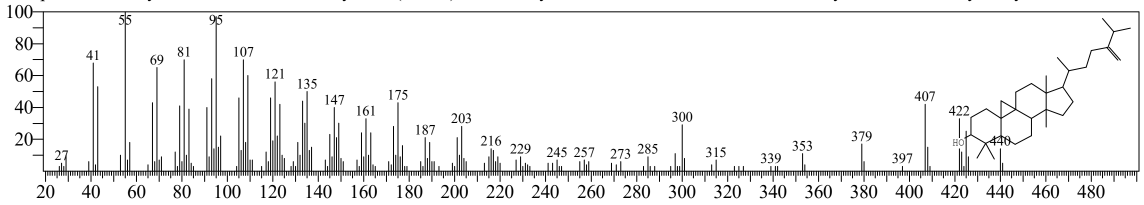

<< Target >>
Line#:50 R.Time:32.327(Scan#:8349) MassPeaks:394
RawMode:Averaged 32.323-32.330(8348-8350) BasePeak:95.05(34803)
BG Mode:Calc. from Peak Group 1 - Event 1 Q3 Scan

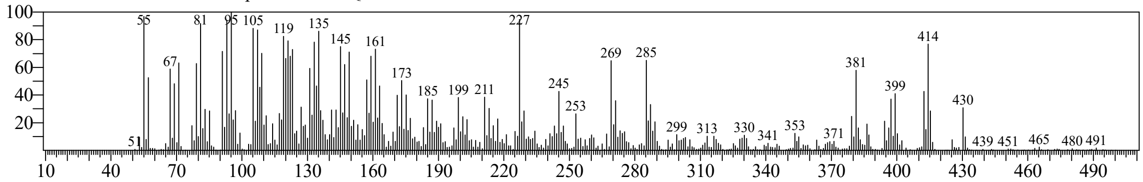

Hit#:1 Entry:150126 Library:NIST17-1.lib
SI:68 Formula:C20H34O2 CAS:4349-94-4 MolWeight:306 RetIndex:2206
CompName:1.alpha.-Methyl-5.alpha.-androstan-3.alpha.,17.beta.-diol

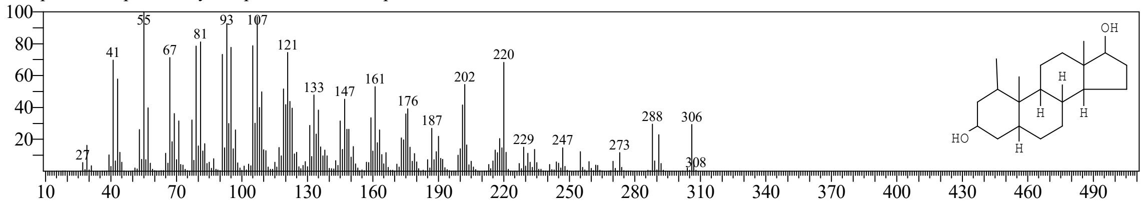

Supplement: Supplementary file 1 [file ijms-26-09156-s001.zip › MS OSO 480min.pdf]
