# Supplementary material for: Ozonized Sunflower Oil: Standardization and Mechanisms of the Antimicrobial Effect
Source: Int J Mol Sci. 2025 Sep 19;26(18):9156. doi: 10.3390/ijms26189156 (PMC12470628; doi:10.3390/ijms26189156)

Centro Integrado de Análises - CIA-FURG
Laboratório de Cromatografia Gasosa - GC-MS/MS
Visite o nosso site - www.cia.furg.br

Relatório de análise - GC-MS/MS

Sample Information

Analyzed by	: Jean Arias
Analyzed	: 01/03/2023 18:47:53
Sample Type	: Unknown
Level #	: 1
Sample Name	: 01032023_Óleo_girassol_PG
Sample ID	:
Tray	: Rack 1
IS Amount	: [1]=1
Sample Amount	: 1
Dilution Factor	: 1
Vial #	: 2
Injection Volume	: 1.00
Data File	: C:\GCMSsolution\Data\Prestações de serviço\01032023_Melk_Cosméticos\01032023_Óleo_girassol_PG.qgd
Org Data File	: C:\GCMSsolution\Data\Prestações de serviço\01032023_Melk_Cosméticos\01032023_Óleo_girassol_PG.qgd
Method File	: C:\GCMSsolution\Data\Prestações de serviço\01032023_Melk_Cosméticos\Ácidos graxos - scan.qgm
Org Method File	: C:\GCMSsolution\Data\Prestações de serviço\01032023_Melk_Cosméticos\Ácidos graxos - scan.qgm
Report File	:
Tuning File	: C:\GCMSsolution\System\Tune1\01032023_tuning.qgt

[Comment]
Perfil Graxo de óleo de girassol

Modified by	: Jean Arias
Modified	: 02/03/2023 11:06:13

Method

[Comment]

===== Analytical Line 1 =====

[GC-2010]

Column Oven Temp.	:80.0 °C
Injection Temp.	:250.00 °C
Injection Mode	:Split
Flow Control Mode	:Linear Velocity
Pressure	:88.5 kPa
Total Flow	:134.2 mL/min
Column Flow	:1.30 mL/min
Linear Velocity	:42.0 cm/sec
Purge Flow	:3.0 mL/min
Split Ratio	:100.0
High Pressure Injection	:ON
High Press. Inj. Pressure	:300.0 kPa
High Press. Inj. Time	:2.30 min
Carrier Gas Saver	:ON
Carrier Gas Saver Split Ratio	:5.0
Carrier Gas Saver Time	:1.00 min
Splitter Hold	:OFF

Oven Temp. Program

Rate	Temperature(°C)	Hold Time(min)
-	80.0	1.00
10.00	180.0	0.00
7.00	330.0	0.00

< Ready Check Heat Unit >
Column Oven : Yes
SPL1 : Yes
MS : Yes
< Ready Check Detector(FTD/BID) >
< Ready Check Baseline Drift >
< Ready Check Injection Flow >
SPL1 Carrier : Yes
SPL1 Purge : Yes
< Ready Check APC Flow >
< Ready Check Detector APC Flow >
External Wait :No
Equilibrium Time :3.0 min

[GC Program]

[GCMS-TQ8050]

IonSourceTemp	:230.00 °C
Interface Temp.	:280.00 °C
Solvent Cut Time	:4.00 min
Detector Gain Mode	:Relative to the Tuning Result
Detector Gain	:1.01 kV +0.00 kV
Threshold	:0
Acquire Data without Using CID Gas(Q3Scan)	:ON

[MS Table]

--Group 1 - Event 1--

Compound Name	:
Start Time	:4.50min
End Time	:32.40min
Acq. Mode	:Q3 Scan
Event Time	:0.200sec
Scan Speed	:2500
Start m/z	:50.00
End m/z	:500.00
Q1 Resolution	: -
Q3 Resolution	: -

Sample Inlet Unit :GC

[MS Program]

Use MS Program :OFF

Peak Report TIC

Peak#	R.Time	Area	Area%	Name
1	12.714	160585	0.06	Myristic acid - C14:0
2	14.946	189548	0.07	Palmitoleic acid - C16:1
3	15.200	29941651	10.82	Palmitic acid - C16:0
4	16.425	186254	0.07	Margaric acid - C17:0
5	17.359	130402812	47.13	Linoleic acid - C18:2
6	17.422	91571841	33.10	Oleic acid - C18:1
7	17.557	1132627	0.41	Linolenic acid - C18:3
8	17.658	16927881	6.12	Stearic acid - C18:0
9	19.735	724453	0.26	Eicosenoic acid - C20:1
10	20.017	1627486	0.59	Arachidic acid - C20:0
11	22.252	2924239	1.06	Behenic acid - C22:0
12	24.344	887708	0.32	Lignoceric acid - C24:0
		276677085	100.00	

Chromatogram 01032023_Óleo_girassol_PG C:\GCMSsolution\Data\Prestações de serviço\01032023_Melk_Cosméticos\01032023_Óleo_girassol_PG.qgd

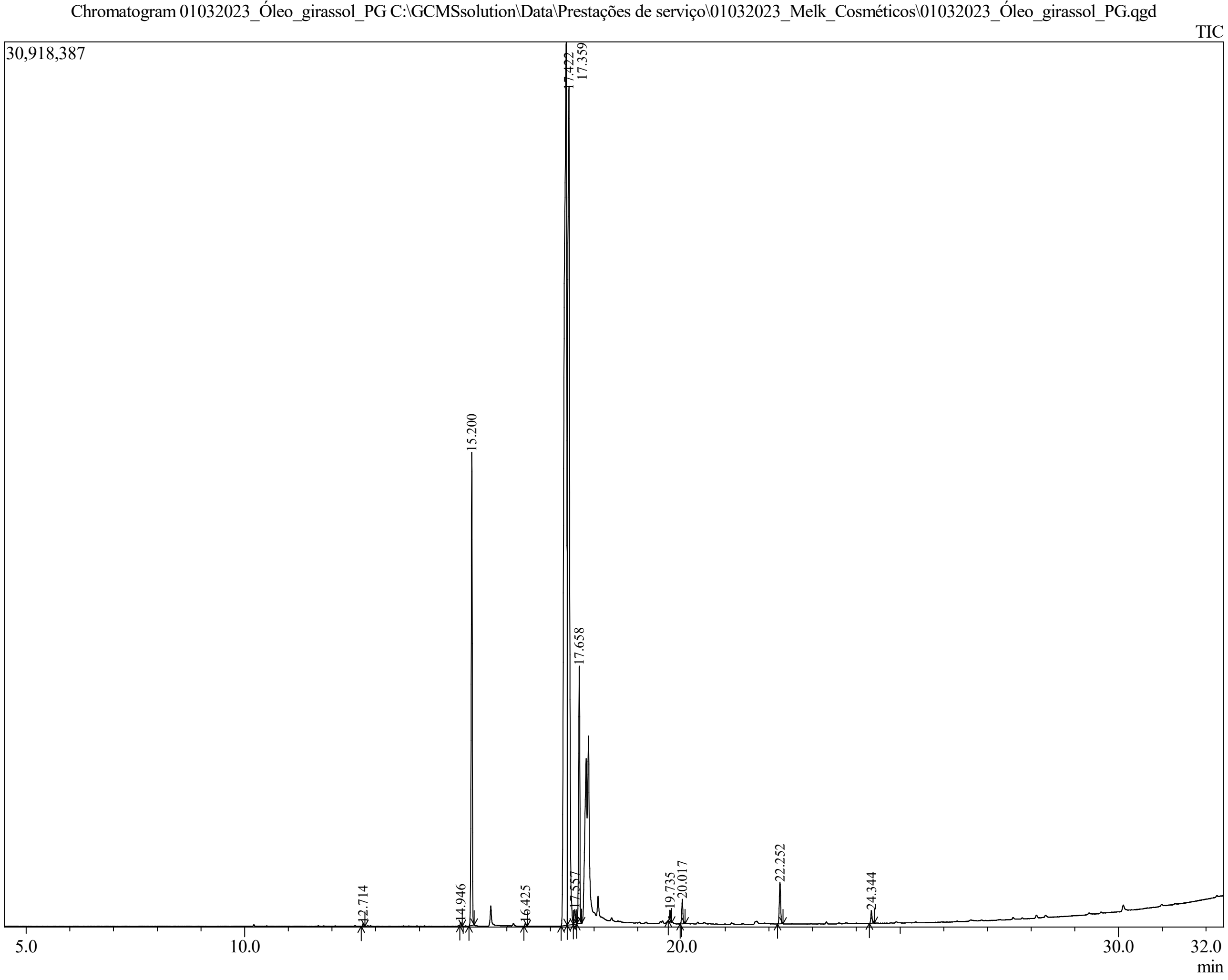

Library

<< Target >>
Line#:1 R.Time:12.713(Scan#:2465) MassPeaks:293
RawMode:Averaged 12.710-12.717(2464-2466) BasePeak:74.05(21515)
BG Mode:Calc. from Peak Group 1 - Event 1 Q3 Scan

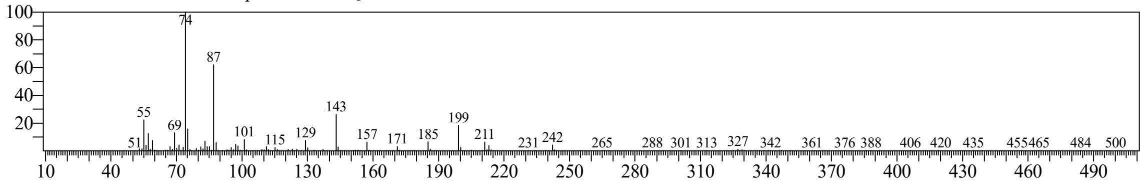

Hit#:1 Entry:86786 Library:NIST17-1.lib
SI:95 Formula:C15H30O2 CAS:124-10-7 MolWeight:242 RetIndex:1680
CompName:Methyl tetradecanoate $$ Tetradecanoic acid, methyl ester $$ Myristic acid, methyl ester $$ Metholeneat 2495 $$ Methyl myristate $$ Methyl m

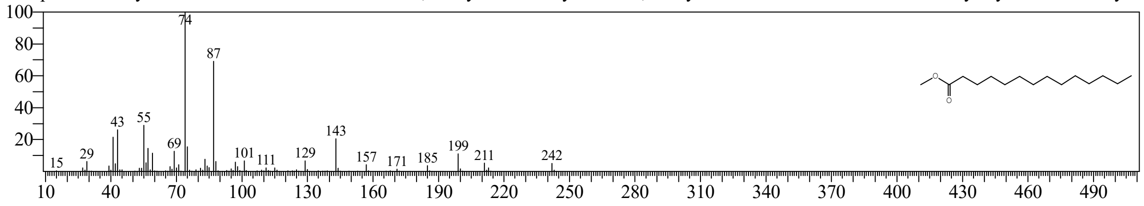

<< Target >>
Line#:2 R.Time:14.947(Scan#:3135) MassPeaks:309
RawMode:Averaged 14.943-14.950(3134-3136) BasePeak:55.05(9085)
BG Mode:Calc. from Peak Group 1 - Event 1 Q3 Scan

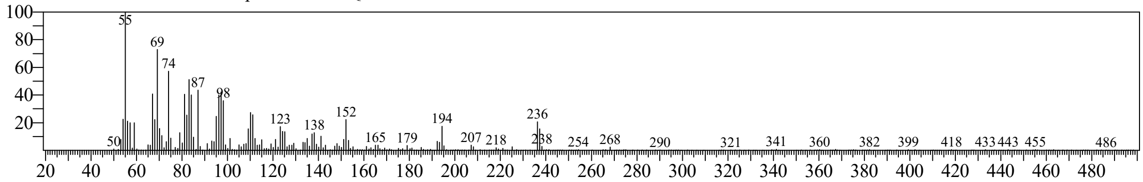

Hit#:1 Entry:30415 Library:NIST17s.lib
SI:95 Formula:C17H32O2 CAS:1120-25-8 MolWeight:268 RetIndex:1886
CompName:9-Hexadecenoic acid, methyl ester, (Z)- $$ Methyl palmitoleate $$ Methyl palmitoleinate $$ Palmitoleic acid, methyl ester $$ Methyl (9Z)-9-he:

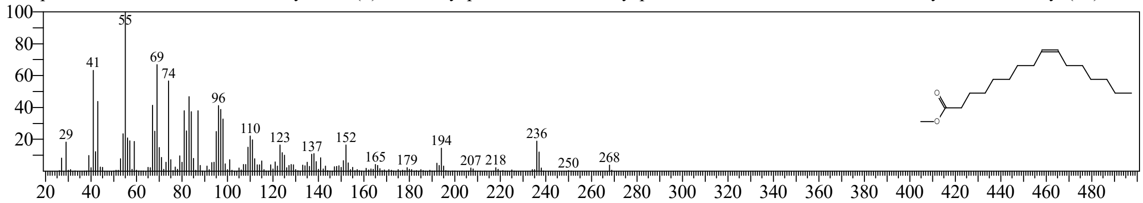

<< Target >>
Line#:3   R.Time:15.200(Scan#:3211) MassPeaks:338
RawMode:Averaged 15.197-15.203(3210-3212) BasePeak:74.05(3172823)
BG Mode:Calc. from Peak Group 1 - Event 1 Q3 Scan

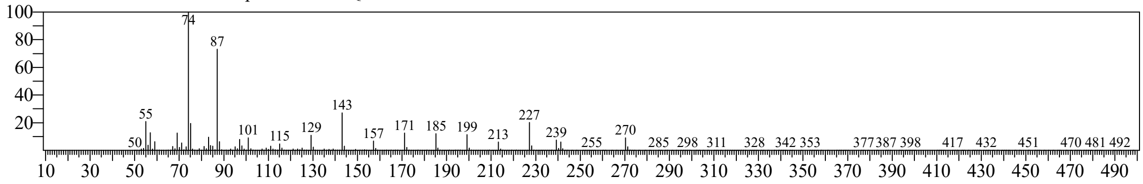

Hit#:1 Entry:113663 Library:NIST17-1.lib
SI:94 Formula:C17H34O2 CAS:112-39-0 MolWeight:270 RetIndex:1878
CompName:Hexadecanoic acid, methyl ester $$ Palmitic acid, methyl ester $$ n-Hexadecanoic acid methyl ester $$ Metholene 2216 $$ Methyl hexadecanoa

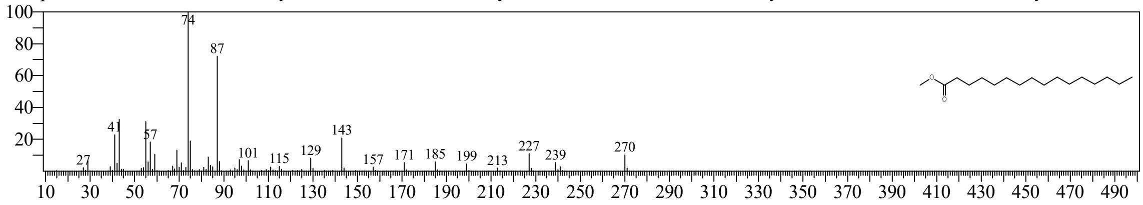

<< Target >>
Line#:4   R.Time:16.427(Scan#:3579) MassPeaks:267
RawMode:Averaged 16.423-16.430(3578-3580) BasePeak:74.05(18467)
BG Mode:Calc. from Peak Group 1 - Event 1 Q3 Scan

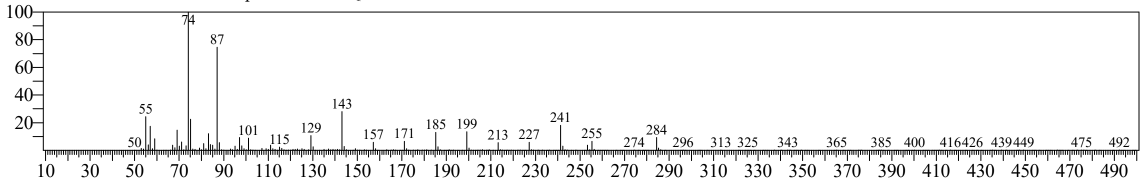

Hit#:2 Entry:31787 Library:NIST17s.lib
SI:94 Formula:C18H36O2 CAS:1731-92-6 MolWeight:284 RetIndex:1978
CompName:Heptadecanoic acid, methyl ester $$ Margaric acid methyl ester $$ Methyl heptadecanoate $$ Methyl margarate $$ n-Heptadecanoic acid methy

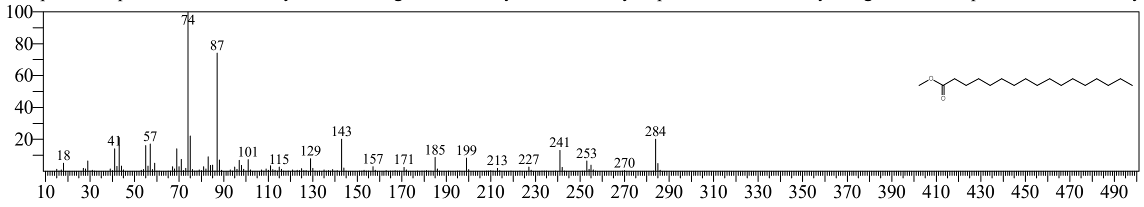

<< Target >>
Line#:5 R.Time:17.360(Scan#:3859) MassPeaks:296
RawMode:Averaged 17.357-17.363(3858-3860) BasePeak:81.10(1677138)
BG Mode:Calc. from Peak Group 1 - Event 1 Q3 Scan

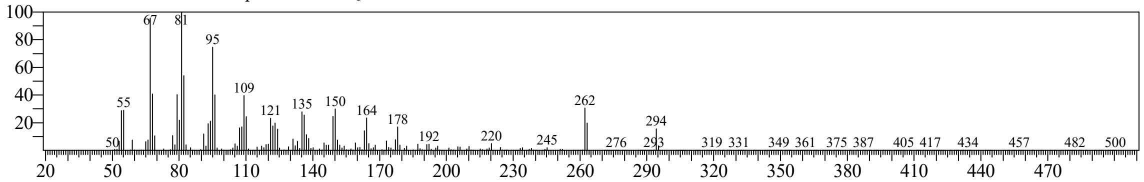

Hit#:2 Entry:33538 Library:NIST17s.lib
SI:87 Formula:C20H36O2 CAS:544-35-4 MolWeight:308 RetIndex:2193
CompName:Linoleic acid ethyl ester $$ Ethyl linoleate $$ 9,12-Octadecadienoic acid (Z,Z)-, ethyl ester $$ Ethyl cis,cis-9,12-octadecadienoate $$ Ethyl linol

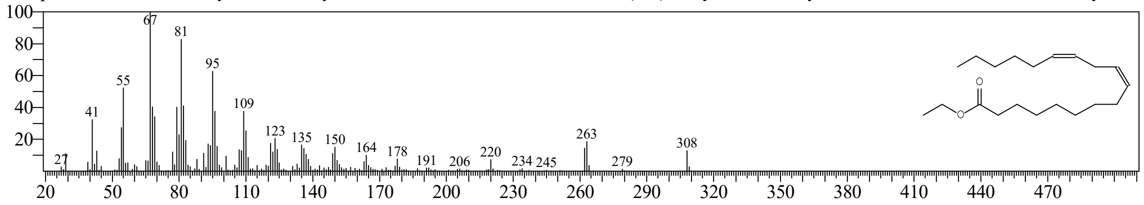

<< Target >>
Line#:6 R.Time:17.420(Scan#:3877) MassPeaks:332
RawMode:Averaged 17.417-17.423(3876-3878) BasePeak:55.05(893049)
BG Mode:Calc. from Peak Group 1 - Event 1 Q3 Scan

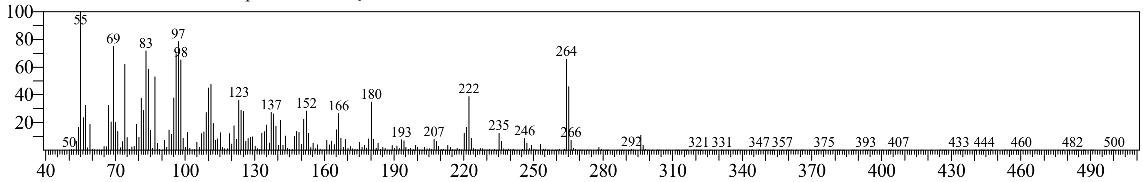

Hit#:3 Entry:32750 Library:NIST17s.lib
SI:85 Formula:C19H36O2 CAS:112-62-9 MolWeight:296 RetIndex:2085
CompName:9-Octadecenoic acid (Z)-, methyl ester $$ Oleic acid, methyl ester $$ Emery oleic acid ester 2301 $$ Methyl cis-9-octadecenoate $$ Methyl ole

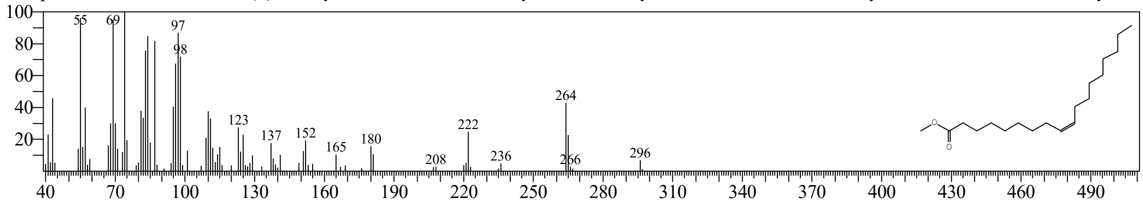

<< Target >>
Line#:7 R.Time:17.557(Scan#:3918) MassPeaks:326
RawMode:Averaged 17.553-17.560(3917-3919) BasePeak:79.05(38940)
BG Mode:Calc. from Peak Group 1 - Event 1 Q3 Scan

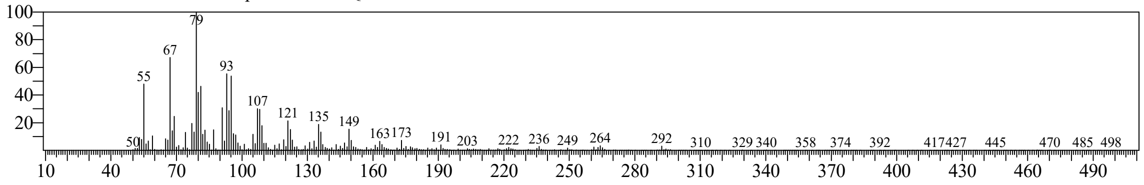

Hit#:1 Entry:135724 Library:NIST17-1.lib
SI:94 Formula:C19H32O2 CAS:301-00-8 MolWeight:292 RetIndex:2101
CompName:9,12,15-Octadecatrienoic acid, methyl ester, (Z,Z,Z)- $$ Linolenic acid, methyl ester $$ Methyl all-cis-9,12,15-octadecatrienoate $$ Methyl lino

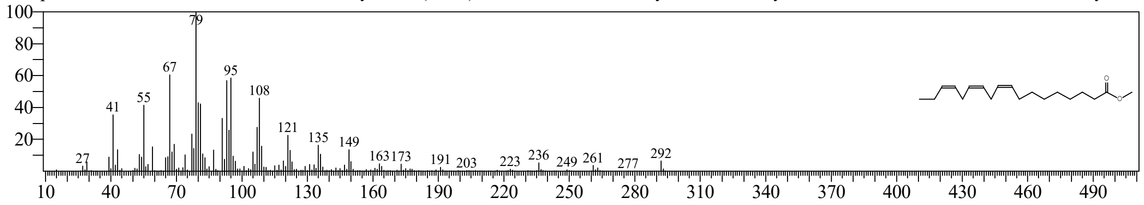

<< Target >>
Line#:8   R.Time:17.660(Scan#:3949) MassPeaks:365
RawMode:Averaged 17.657-17.663(3948-3950) BasePeak:74.05(1636551)
BG Mode:Calc. from Peak Group 1 - Event 1 Q3 Scan

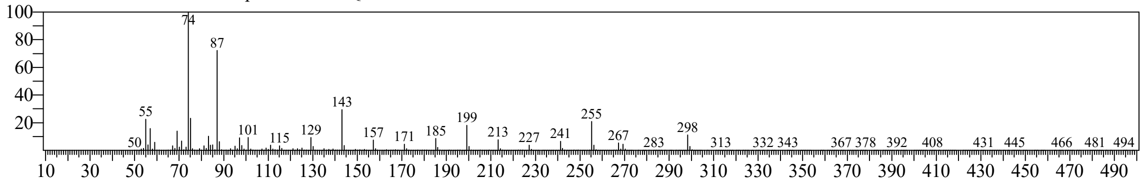

Hit#:1 Entry:32878 Library:NIST17s.lib
SI:95 Formula:C19H38O2 CAS:112-61-8 MolWeight:298 RetIndex:2077
CompName:Methyl stearate $$ Octadecanoic acid, methyl ester $$ Stearic acid, methyl ester $$ n-Octadecanoic acid, methyl ester $$ Kemester 9718 $$ Me

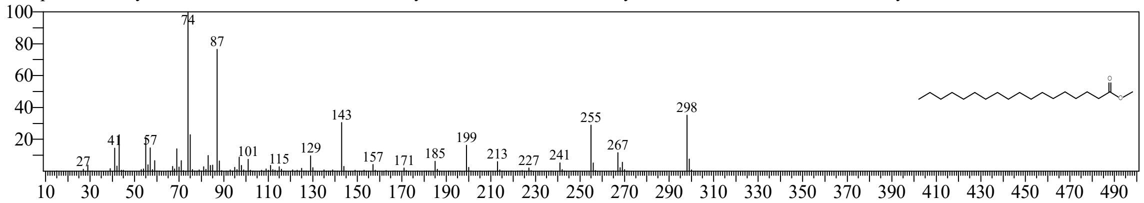

<< Target >>
Line#:9   R.Time:19.737(Scan#:4572) MassPeaks:316
RawMode:Averaged 19.733-19.740(4571-4573) BasePeak:55.05(26277)
BG Mode:Calc. from Peak Group 1 - Event 1 Q3 Scan

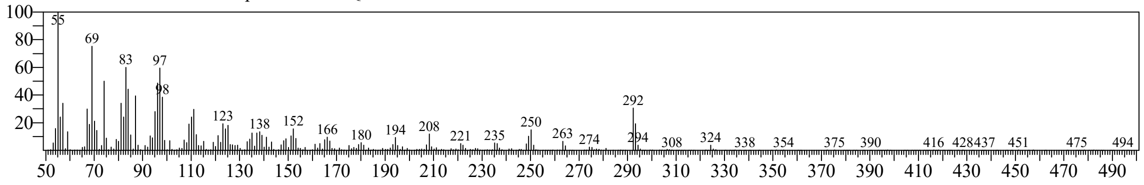

Hit#:1 Entry:34618 Library:NIST17s.lib
SI:95 Formula:C21H40O2 CAS:3946-08-5 MolWeight:324 RetIndex:2284
CompName:11-Eicosenoic acid, methyl ester $$ Methyl (11E)-11-icosenoate # $$

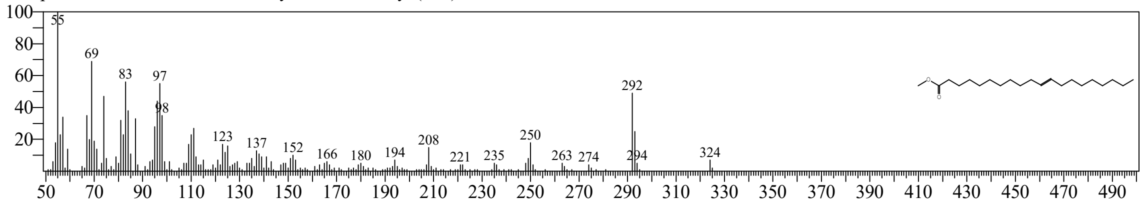

<< Target >>
Line#:10   R.Time:20.017(Scan#:4656) MassPeaks:322
RawMode:Averaged 20.013-20.020(4655-4657) BasePeak:74.05(137381)
BG Mode:Calc. from Peak Group 1 - Event 1 Q3 Scan

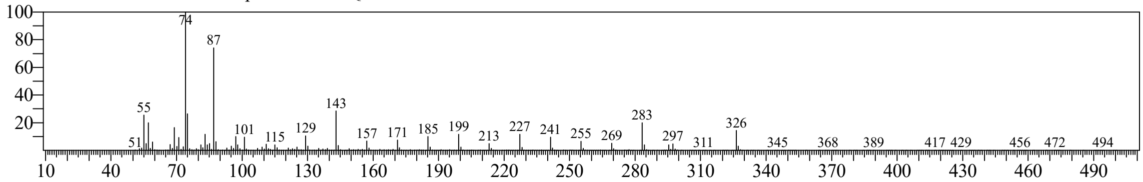

Hit#:2 Entry:34726 Library:NIST17s.lib
SI:91 Formula:C21H42O2 CAS:1120-28-1 MolWeight:326 RetIndex:2276
CompName:Eicosanoic acid, methyl ester $$ Methyl arachisate $$ Methyl eicosanoate $$ Arachidic acid methyl ester $$ Kemester 2050 $$ Methyl aracidat

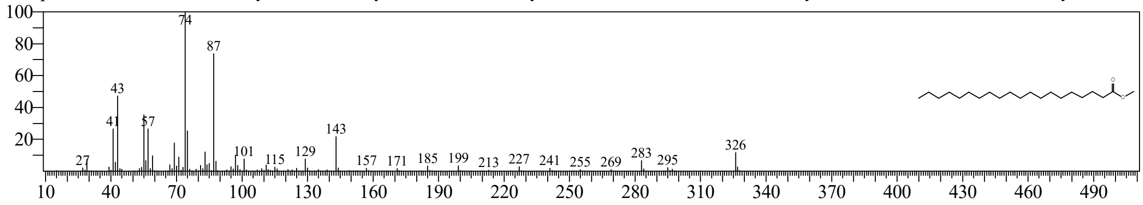

<< Target >>
Line#:11   R.Time:22.253(Scan#:5327) MassPeaks:341
RawMode:Averaged 22.250-22.257(5326-5328) BasePeak:74.05(211836)
BG Mode:Calc. from Peak Group 1 - Event 1 Q3 Scan

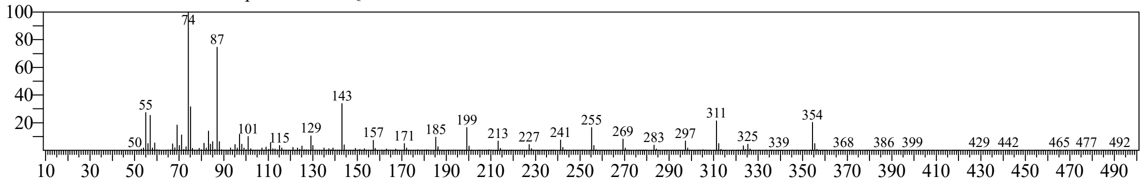

Hit#:2 Entry:196443 Library:NIST17-1.lib
SI:90 Formula:C23H46O2 CAS:929-77-1 MolWeight:354 RetIndex:2475
CompName:Docosanoic acid, methyl ester $$ Behenic acid, methyl ester $$ Methyl behenate $$ Methyl docosanoate $$ n-Docosanoic acid methyl ester $$

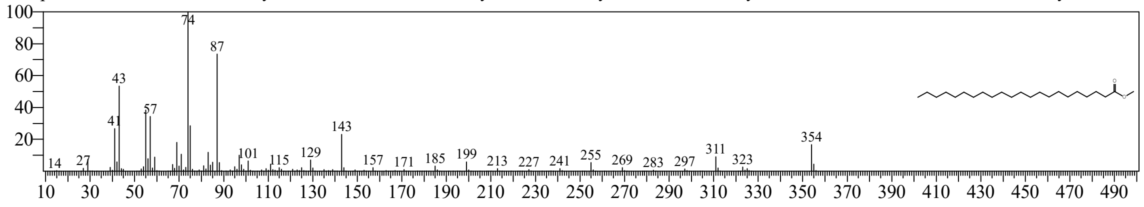

<< Target >>
Line#:12   R.Time:24.343(Scan#:5954) MassPeaks:316
RawMode:Averaged 24.340-24.347(5953-5955) BasePeak:74.05(57495)
BG Mode:Calc. from Peak Group 1 - Event 1 Q3 Scan

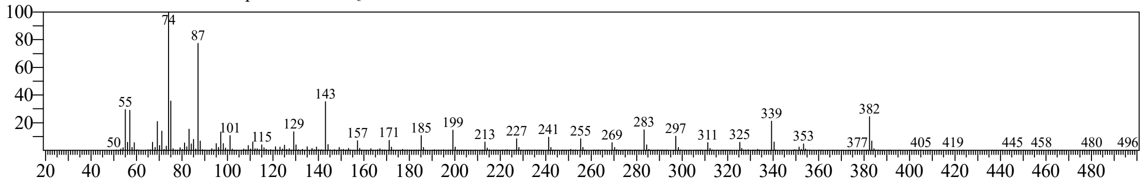

Hit#:1 Entry:37107 Library:NIST17s.lib
SI:92 Formula:C25H50O2 CAS:2442-49-1 MolWeight:382 RetIndex:2674
CompName:Tetracosanoic acid, methyl ester $$ Methyl lignocerate $$ Methyl tetracosanoate $$ Lignoceric acid methyl ester $$

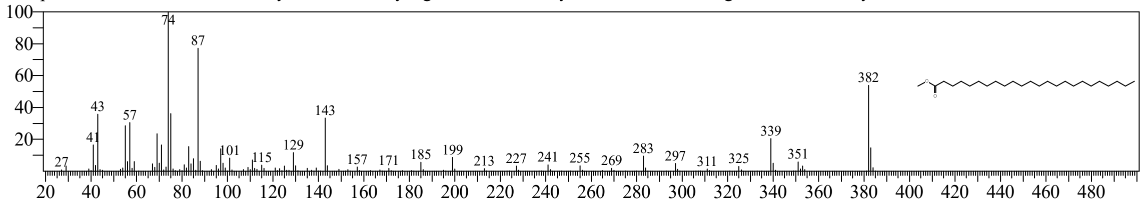

Supplement: Supplementary file 1 [file ijms-26-09156-s001.zip › Pure SO.pdf]
